# Synergetic iridium and amine catalysis enables asymmetric [4+2] cycloadditions of vinyl aminoalcohols with carbonyls

**DOI:** 10.1038/s41467-019-10674-3

**Published:** 2019-06-20

**Authors:** Mao-Mao Zhang, Ya-Ni Wang, Bao-Cheng Wang, Xiao-Wang Chen, Liang-Qiu Lu, Wen-Jing Xiao

**Affiliations:** 1 CCNU-uOttawa Joint Research Centre, Key Laboratory of Pesticide and Chemical Biology, Ministry of Education; College of Chemistry, Central China Normal University, 152 Luoyu Road, Wuhan, 430079 China; 2 State Key Laboratory for Oxo Synthesis and Selective Oxidation, Lanzhou Institute of Chemical Physics (LICP), Chinese Academy of Sciences, Lanzhou, 730000 China; 3 State Key Laboratory of Applied Organic Chemistry, College of Chemistry and Chemical Engineering, Lanzhou University, Lanzhou, 730000 China

**Keywords:** Asymmetric synthesis, Synthetic chemistry methodology, Stereochemistry

## Abstract

Catalytic asymmetric cycloadditions via transition-metal-containing dipolar intermediates are a powerful tool for synthesizing chiral heterocycles. However, within the field of palladium catalysis, compared with the well-developed normal electron-demand cycloadditions with electrophilic dipolarophiles, a general strategy for inverse electron-demand ones with nucleophilic dipolarophiles remains elusive, due to the inherent linear selectivity in the key palladium-catalyzed intermolecular allylations. Herein, based on the switched regioselectivity of iridium-catalyzed allylations, we achieved two asymmetric [4+2] cycloadditions of vinyl aminoalcohols with aldehydes and β,γ-unsaturated ketones through synergetic iridium and amine catalysis. The activation of vinyl aminoalcohols by iridium catalysts and carbonyls by amine catalysts provide a foundation for the subsequent asymmetric [4+2] cycloadditions of the resulting iridium-containing 1,4-dipoles and (di)enamine dipolarophiles. The former provides a straightforward route to a diverse set of enantio-enriched hydroquinolines bearing chiral quaternary stereocenters, and the later represent an enantio- and diastereodivergent synthesis of chiral hydroquinolines.

## Abstract (web summary)

Inverse electron-demand cycloadditions in palladium catalysis are inherently limited by the linear selectivity in the allylic substitution step. To overcome this issue, the authors report a synergetic iridium (branched-selective) and amine catalysis in [4+2] cycloadditions of vinyl aminoalcohols with carbonyls.

## Introduction

**Fig. 1:**
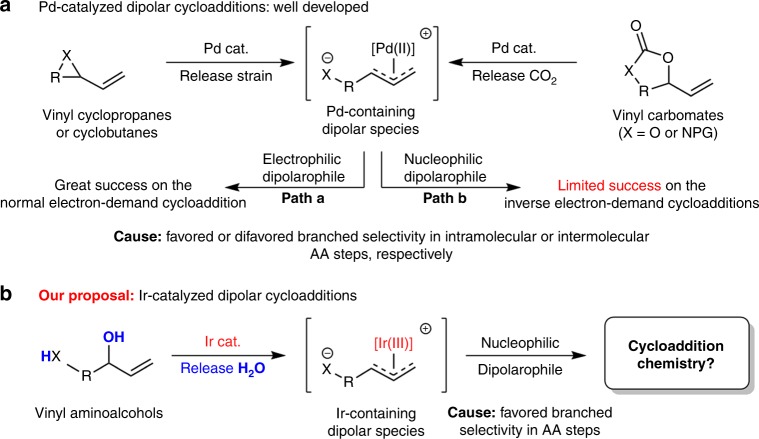
Research proposal on the Ir-catalyzed cycloadditions of vinyl aminoalcohols. **a** Well-developed dipolar cycloadditions through asymmetric palladium catalysis and remained problem. **b** Dipolar cycloadditions through asymmetric iridium catalysis

Heterocycles are privileged motifs in fields ranging from pharmaceutical chemistry and agrochemistry to materials chemistry and life sciences. The development of efficient synthetic methods for preparing heterocyclic compounds has been a major focus of the synthetic community^1,2^. Among the continuing and productive efforts, catalytic asymmetric cycloadditions via transition metal (TM)-containing dipolar intermediates have been determined to be powerful tools for this purpose^3–5^. Within this realm of palladium catalysis, although many impressive advancements have been made regarding cycloadditions of Pd-containing dipolar intermediates with electrophilic dipolarophiles (Fig. 1a, path a: normal electron-demand cycloadditions)^6–12^, the inherent linear selectivity of Pd-catalyzed intermolecular allylic alkylation (AA) reactions renders the development of a general strategy for the coupling of such intermediates with nucleophilic dipolarophiles a formidable task (Fig. 1a, path b: inverse electron-demand cycloaddition)^13,14^. In the few known examples, some additional interaction (i.e., electrostatic interactions or hydrogen bonding interactions) between the Pd-containing dipole and nucleophilic dipolarophile was required to induce branched selectivity in the Pd-catalyzed intermolecular AA processes^15–19^. For this reason, the exploitation of alternative catalyst systems is required to develop general dipolar cycloadditions and efficiently utilize the synthetic potential of TM catalysis in the synthesis of heterocycles.

Based on the pioneering works of Takeuchi^20,21^ and Helmchen^22^, as well as the substantial contribution from Helmchen^23^, Hartwig^24^, Carreira^25^, You^26^ and many other excellent scientists^27–29^, iridium-catalyzed AA reactions show excellent branched selectivity, which distinguishes them from Pd-catalyzed processes^30^. Recent studies have indicated that iridium catalysis is compatible with many other catalysis modes (i.e., phase catalysis^31^, amine catalysis^32–34^, Lewis base catalysis^35^, Brønsted acid catalysis^36^, and Lewis acid catalysis^37–40^), significantly expanding the scope of Ir-catalyzed asymmetric allylic alkylation (AAA) reactions^41^. Inspired by these impressive achievements, we question whether a synergetic catalysis strategy involving iridium catalysis can be adopted to resolve the remaining problem associated with Pd-catalyzed inverse electron-demand cycloadditions. More interestingly, readily available vinyl aminoalcohols can be directly utilized as coupling partners in TM-catalyzed cycloadditions using Ir-containing dipoles (Fig. 1b), avoiding the use of vinyl-substituted strained rings and carbonates like those required in Pd-catalyzed cycloaddition (Fig. 1a). Here, through the combination of iridium and amine catalysis, we accomplished the [4+2] cycloadditions of vinyl aminoalcohols with

aldehydes and β,γ-unsaturated ketones. Optically active quinolinones and tetrahydroquinolines were produced in good yields and with high diastereoselectivity and enantioselectivity.

## Results

### Design plan

**Fig. 2:**
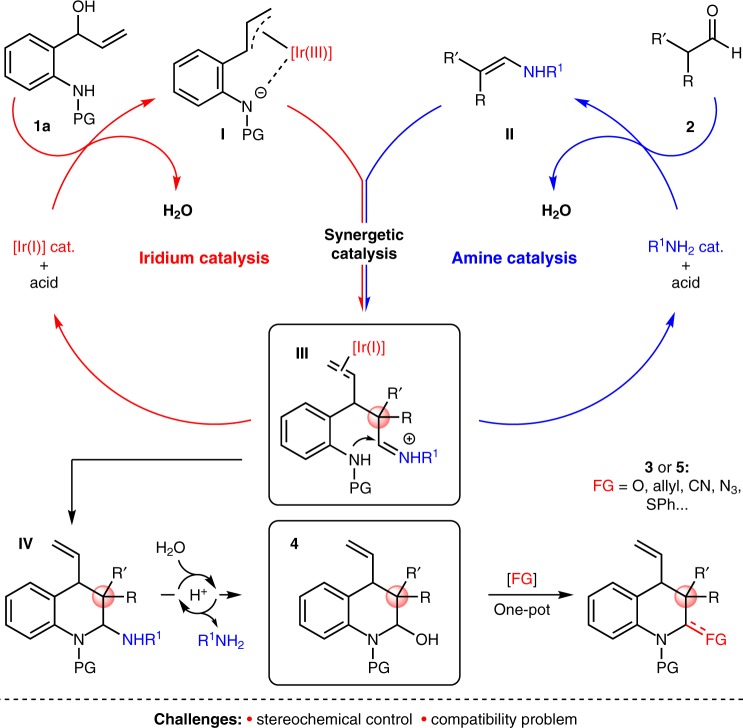
Proposed mechanism. Synergetic iridium/amine catalysis for the enantioconvergent syntheses of hydroquinolines

Hydroquinolines are a class of important aza-heterocycles that are ubiquitous in natural alkaloids and functional molecules (i.e., pharmaceuticals, agrochemicals, and chiral ligands)^42–44^. Among existing methods for synthesizing chiral hydroquinolines, catalytic asymmetric [4+2] cycloadditions stand out as one of the most streamlined approaches^45–49^. As part of our ongoing studies on the synthesis of heterocycles via TM-catalyzed cycloadditions^15,50–55^, in this work, we plan to develop reaction methodologies that utilize readily available reagents and feature good selectivity and high synthetic efficiency. A detailed description of a possible mechanism is outlined in Fig. 2. We envision that two catalytic cycles could act in concert to realize this cycloaddition via the following steps: (1) the Ir catalyst reacts with vinyl aminoalcohol **1a** to form Ir-containing dipole int. **I**^56,57^; (2) the amine catalyst condenses with the aldehyde to generate enamine species **II**; (3) these two transient species, **I** and **II**, react with each other to produce int. **III**, which can be further converted to hemiacetal **4** through an intramolecular cyclization (**III** → **IV**) and acid-promoted hydrolysis process (**IV** → **4**). Based on this mechanism, a diverse set of hydroquinolines can be generated in a facile manner by in situ conversion of the OH group to a variety of other functional groups (**4** → **3**/**5**). While attractive in theory, the construction of contiguous stereocenters and chiral all-carbon quaternary stereocenters^58–62^, as well as the compatibility between the catalysts, substrates, and additives are challenges that must be addressed.

### Condition optimization

First, we investigated the Ir/amine-catalyzed cycloaddition/oxidation sequence using vinyl aminoalcohol **1a** and aldehyde **2a**. The use of 4 mol% of chiral Ir catalyst (2 mol% of [Ir(cod)Cl]_2_ and 8 mol% of Carreira ligand **L1**) and 20 mol% of amine catalyst **A1**, together with 0.5 eq. of CCl_3_CO_2_H and 1.0 eq. of H_2_O as additives, gave hemiaminal **4aa** as a white solid in 92% yield; **4aa** can be further oxidized to desired hydroquinone **3aa** by PCC in 67% yield with 99% ee and >19:1 dr. The absolute configuration of product **3aa** was determined by comparing the chiral HPLC spectra with a previous literature^55^. To circumvent the isolation of intermediate **4aa**, we adopted an improved procedure that simply combined these two processes in one pot. To our delight, quinolinone product **3aa** was successfully generated with good reaction efficiency and excellent enantiocontrol under the standard conditions (Table 1, entry 1: 64% yield, >99% ee and >19:1 dr). We then found that the protecting group on the nitrogen atom of the vinyl aminoalcohol had a substantial effect on the reaction selectivity. For example, when Boc-protected substrate **1a**′ was used instead of **1a**, we only identified the byproduct from the allylation of CCl_3_CO_2_H (Table 1, entry 2). It is worthy to note that, we have proven that the Ts group on product **3aa** could be facilely removed by treatment with Mg powder in methanol (See Supplementary Procedure K). When vinyl aminoalcohol **1a**′′ was tested, there was almost no conversion of starting materials (Table 1, entry 3). Furthermore, the effects of the acid and water as additives were investigated (Table 1, entries 4–7). CCl_3_CO_2_H provided superior chemo- and enantioselectivities, and the addition of 1 eq. of water increased the reaction efficiency. Considering that the amine catalyst may play a key role in this reaction, various amine catalysts were examined. As shown in Table 1, a bulkier amine, α,α-dimethyl benzylamine (Table 1, entry 8, **A2**), gave only 22% yield, albeit with >19:1 dr and >99% ee. In addition, when chiral amine catalysts **(*R*)-A3** and **(*S*)-A3** were applied, we did not observe stereodivergent products, but the different amines did result in different reaction efficiencies (Table 1 entries 9 and 10).

**Table 1:** Optimization of the reaction conditions for the [4+2] cycloaddition of vinyl aminoalcohols and aldehydes^a^


Entry	Variation from the standard conditions	Yield (%)^b^	ee (%′)^c^
1	none	64	99
2	replace **1a** with **1a′**	0	/
3	replace **1a** with **1a′′**	0	/
4	replace CCl_3_CO_2_H with CF_3_CO_2_H	63	82
5	replace CCl_3_CO_2_H with (PhO)_2_PO_2_H	58	98
6	replace CCl_3_CO_2_H with PhCO_2_H	0	/
7	no H_2_O	56	99
8	replace **A1** with **A2**	22	95
9	replace **A1** with **(*R*)-A3**	62	93
10	replace **A1** with **(*S*)-A3**	26	94


*Ts* *p*-toluene sulfonyl, *Boc* *t*-butyloxycarbonyl, *PCC* pyridinium chlorochromate, *Am* amine catalyst

^a^Standard conditions: **1a** (0.25 mmol), **2a** (0.5 mmol), [Ir(cod)Cl]_2_ (2 mol%), **(*R*)-L1** (8 mol%), amine **A1** (20 mol%), CCl_3_CO_2_H (0.5 eq.) and H_2_O (1.0 eq.) in 1,2-dichloroethane (0.5 mL) for 48 h at room temperature; then, PCC (5.0 eq.), silica gel (100 mg) and CH_2_Cl_2_ (5 mL) were added, and the mixture was stirred at 40 °C for 10 h

^b^Isolated yield

^c^Determined by chiral HPLC analysis; dr > 19:1

### Substrate scope

Having established the optimal conditions, we started to explore the generality of the vinyl aminoalcohol substrate. As summarized in Table 2, a wide range of vinyl aminoalcohols with electron-donating and electron-withdrawing groups, such as Me, F, and CF_3_, at the 5-position of the phenyl ring can readily react with aldehyde **2a** to afford the desired dihydro-quinolinone products in moderate yields over two steps with high diastereo- and enantioselectivities (Table 2, entries 1–4, **3aa**–**3da**: 59–66% yields, 6:1→ 19:1 dr and 94–99% ee). Furthermore, substrates with different substitution patterns, for example, 4-MeO, 4-Cl, and 6-F, were compatible with this asymmetric cycloaddition. The corresponding cycloadducts were achieved in satisfactory reaction efficiencies and stereoselectivities (Table 2, entries 5–7, **3ea**–**3ga**: 61–73% yields, 12:1→19:1 dr and 98–99% ee). An allyl alcohol bearing a naphthyl moiety was also suitable for this reaction, and it was converted to product **3ha** in 63% yield, 12:1 dr and 98% ee (Table 2, entry 8).

**Table 2:** Synthesis of Dihydroquinolinones form Various Vinyl Aminoalcohols^a^


Entry	1: R^1^	3	Yield (%)^b^	dr^c^	ee (%)^c^
1	**1a**: H	**3aa**	64	>19:1	99
2	**1b**: 5-Me	**3ba**	59	16:1	96
3	**1c**: 5-F	**3ca**	59	8:1	94
4	**1d**: 5-CF_3_	**3da**	66	6:1	98
5	**1e**: 4-MeO	**3ea**	61	19:1	99
6	**1f**: 4-Cl	**3fa**	60	12:1	99
7	**1g**: 6-F	**3ga**	73	>19:1	99
8			63	12:1	98

^a^Standard conditions as indicated in entry 1 of Table 1

^b^Isolated yield

^c^Determined by chiral HPLC analysis

**Fig. 3:**
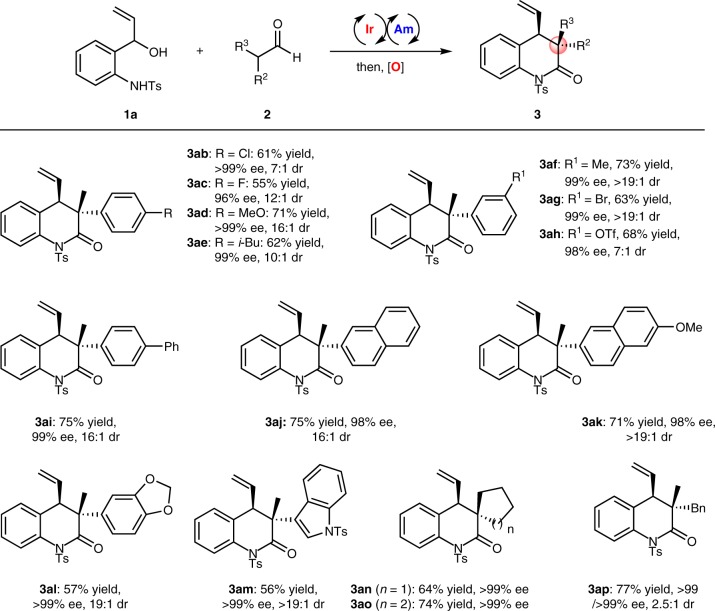
Synthesis of dihydroquinolinones from various aldehydes. Reaction conditions: as indicated in entry 1 in Table 1; isolated yield; the ee and dr values were determined by chiral HPLC analysis

Subsequently, we explored an array of α-disubstituted aldehydes in this Ir- and amine-catalyzed cycloaddition/oxidation procedure. As summarized in Fig. 3, the introduction of various groups at the *para*- or *meta*-positions of the 2-phenylpropanal was well tolerated in this reaction, and corresponding products **3ab**–**3ai** were obtained in good yields (55%−75% yields) and with high stereoselectivities (up to >19:1 dr and 96 → 99% ee). Moreover, aldehydes with naphthalene and other polycyclic systems can be converted to corresponding dihydro-quinolinones **3aj**–**3am** in good yields and excellent enantioselectivities (56–75% yields and up to >99% ee). Notably, an indole-derived substrate was also well tolerated in this reaction and provided a good result (**3am**, 56% yield, >19:1 dr and >99% ee). In addition, cyclic aldehydes with various ring sizes (i.e., five- or six-membered rings) could readily participate in this reaction (**3an**, 64% yield, >99% ee; **3ao**, 74% yield, >99% ee). A dialkyl-substituted acetaldehyde, 2-methyl-3-phenylpropanal, could also be reacted under these conditions to give the desired product **3ap** in a good yield and with an excellent enantioselectivity. A linear aldehyde, *n*-butanal, was also proven suitable for this transformation, affording dihydroquinolinone product **3ar** in 31% yield, >99% ee and >20:1 dr (See Supplementary Procedure M).

### Demonstrations of synthetic utility

**Fig. 4:**
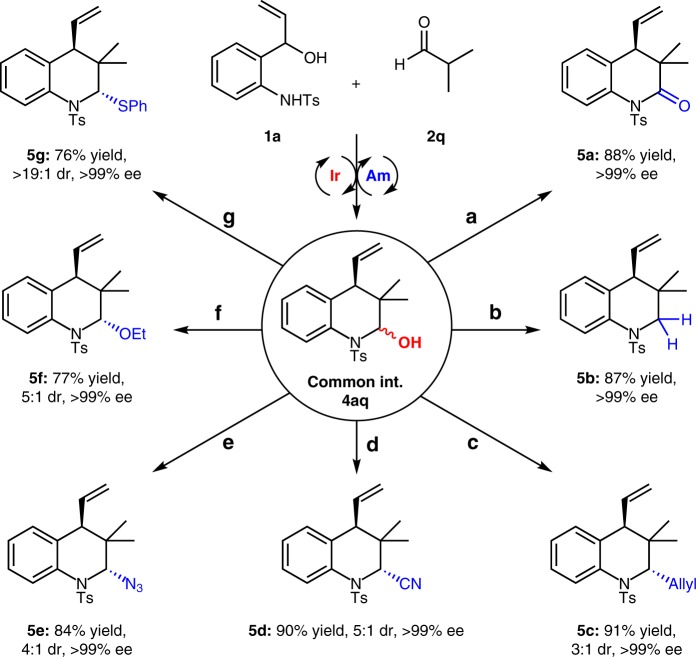
Divergent synthetic transformations of chiral hemiaminal **4aq**. Reaction conditions: **a**) PCC (5.0 eq.), silica (100 mg), CH_2_Cl_2_ (5 mL), 40 °C, 10 h; **b**) Et_3_SiH (3.0 eq.), Et_2_O·BF_3_ (3.0 eq.), CH_2_Cl_2_ (3 mL), 0 °C, 5 min; **c**) allyltrimethylsilane (3.0 eq.), Et_2_O·BF_3_ (3.0 eq.), CH_2_Cl_2_ (3 mL), 0 °C, 5 min; **d**) TMSCN (2.0 eq.), Et_2_O·BF_3_ (3.0 eq.), CH_2_Cl_2_ (3 mL), 0 °C, 5 min; **e**) TMSN_3_ (2.0 eq.), Et_2_O·BF_3_ (3.0 eq.), CH_2_Cl_2_ (3 mL), 0 °C, 5 min; **f**) *p*-TSA (20 mol%), EtOH (2.5 mL), 30 °C, 10 h. **g**) PhSH (1.5 eq.), Et_2_O·BF_3_ (3.0 eq.), CH_2_Cl_2_ (3 mL), 0 °C, 5 min; isolated yield; the ee and dr values were determined by chiral HPLC analysis of purified products and ^1^H NMR analysis of reaction mixtures, respectively. *p*-TSA: *p*-toluenesulfonic acid

To illustrate the versatility of this cycloaddition, we explored various derivatizations of the hemiaminal intermediate. As highlighted in Fig. 4, taking key hemiaminal **4aq** (>99% ee and 5:1 dr), which was generated from the asymmetric cycloaddition of vinyl aminoalcohol **1a** and isobutyraldehyde **2q**, as an example, treatment with various organic reagents in one-pot reactions indeed provided a diverse set of functionalized hydroquinolines in generally high yields and excellent enantioselectivities. Except in the oxidation with PCC, the OH group of hemiaminal **4aq** could be removed by treating the compound with Et_3_SiH and Et_2_O·BF_3_ in DCM, giving tetrahydroquinoline **5b** in 87% yield and >99% ee. Similarly, replacement of the hydroxyl group with other organosilicon reagents could deliver 2-allyl-, 2-CN- and 2-N_3_-substituted tetrahydroquinolines **5c**–**5e** in 84–91% yields and >99% ee, albeit with modest dr values. In addition, the OH group can be successfully converted to ether or sulfide moieties in good yields and excellent enantioselectivities (**5f**, 77% yield, 5:1 dr and >99% ee; **5g**, 76% yield, >19:1 dr and >99% ee. The high diastereoselectivity of product **5g** may be result of the steric effect (See Supplementary Procedures I and J), and its absolute configuration was established through the X-ray diffraction analysis (CCDC 1821791)^63^.

### Stereodivergent [4+2] cycloadditions

**Fig. 5:**
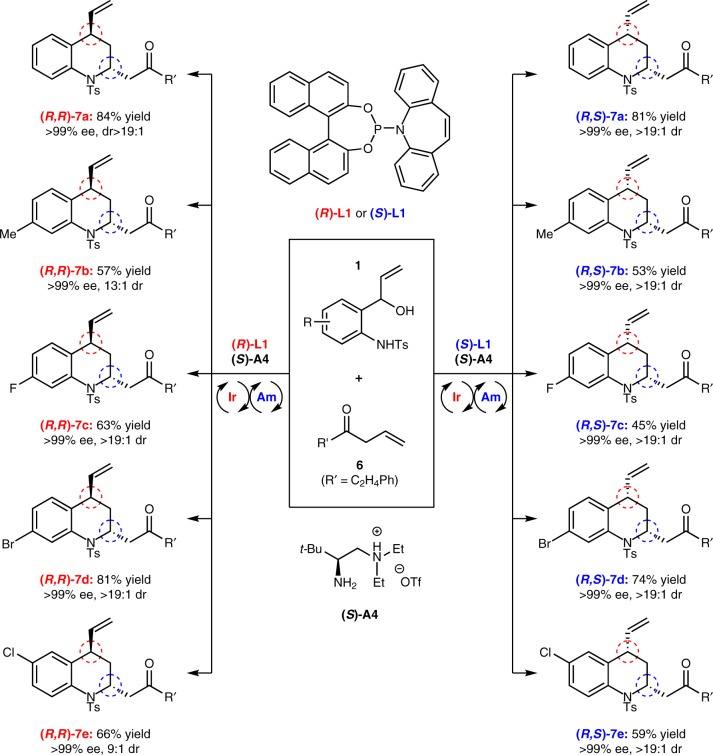
Enantioselective and diastereodivergent [4+2] cycloadditions of vinyl aminoalcohols and β,γ-unsaturated ketones. Reaction conditions: **1** (0.25 mmol), **6** (1.25 mmol), [Ir(cod)Cl]_2_ (2 mol%), **(*R*)-L1** or **(*S*)-L1** (8 mol%), amine **(*S*)-A4** (20 mol%), in 1,2-dichloroethane (0.3 mL) for 48 h at 50 ^o^C; isolated yield; the values of ee and dr were determined by chiral HPLC analysis

In addition to activating the α-position of the aldehyde via an enamine mechanism^64^, amine catalysis can also be used to efficiently activate the γ-position of the β,γ-unsaturated ketone through the formation of an dienamine species^65–69^. Based on this information and our above impressive results, we further probed the asymmetric [4 + 2] cycloadditions of vinyl aminoalcohols with β,γ-unsaturated ketones by merging iridium catalysis with dienamine catalysis. With the cycloaddition of vinyl aminoalcohol **1a** and ketone **6** as model substrates, a simple optimization of the reaction parameters including chiral amine catalyst, solvent, and acid defined the optimal reaction conditions (See Supplementary Table 3 for the details of condition optimization). To our delight, unlike the cycloaddition of vinyl aminoalcohols **1** with aldehyde **2**, which provides a pair of diastereomers, by reasonably using Carreira’s chiral phosphoramidite ligands, **(*R*)-L1** or **(*S*)-L1**, and Luo’s primary amine catalyst **(*S*)-A4**^70^, this reaction can provide both members of either of two pairs of diastereomers in good yields and with excellent enantio- and diastereoselectivities (Fig. 5, (***R***,***R***)-**7a**, 84% yield, >99% ee and >19:1 dr; **(*R***,***S*)-7a**, 81% yield, >99% ee and >19:1 dr). Worthy to note that, other two stereoisomers of **7a** have been synthesized by using the enantiomer of Luo’s catalyst **(*S*)-A4**, too (See Supplementary Table 3 for details). The absolute configuration of product **(*R***, ***S*)**-**7a** were established by analyzing its derivative through the X-ray diffraction analysis (CCDC 1869868, See Supplementary Note 1 for details)^25^. Encouraged by these results, experiments to preliminarily examine the scope of vinyl aminoalcohols for this stereodivergent cycloaddition were performed. As highlighted in Fig. 4, moderate to good yields together with high levels of enantio- and diastereocontrol were generally observed for both pairs of diastereomers. In a word, this is an interesting but challenging work that high diastereo- and enantioselectivities have been simultaneously accomplished in a stereodivergent manner, demonstrating the power and potential of synergetic catalysis strategies in the field of TM-catalyzed dipolar cycloaddition.

## Discussion

Overall, we have successfully developed two enantioselective [4+2] cycloadditions of vinyl aminoalcohols with carbonyls, namely, aldehydes and β,γ-unsaturated ketones, through synergetic iridium and amine catalysis. The cycloaddition with aldehydes provides a diverse set of hydroquinolines bearing chiral quaternary stereocenters with a high level of enantiocontrol. The reaction with β,γ-unsaturated ketones allows the enantio- and diastereodivergent synthesis of hydroquinoline products using reasonable chiral iridium catalysts and chiral amine catalysts. Obviously, the formation of Ir-containing dipoles from vinyl aminoalcohols and chiral iridium catalysts and (di)enamine dipolarophiles from carbonyls and amine catalysts provides a foundation for subsequent asymmetric [4+2] dipolar cycloadditions. As demonstrated in this work, the application of synergetic catalysis makes transition metal-catalyzed cycloadditions more practical and powerful for the synthesis of chiral heterocycles.

## Methods

### Preparation of product 3

In a dried Schlenk tube under N_2_, [Ir(cod)Cl]_2_ (0.005 mmol, 3.35 mg) and chiral ligand **(*R*)**-**L1** (0.02 mmol, 10.15 mg) were mixed in 0.5 mL DCE and stirred at ambient temperature for 20 min under argon atmosphere. Then allylic alcohol **1** (0.25 mmol, 1.0 eq), aldehyde **2** (0.5 mmol, 2.0 eq), Cl_3_CCOOH (0.125 mmol, 20.4 mg), amine **A1** (0.05 mmol, 9.2 mg, 8.7 μL), 4.5 μL H_2_O (1.0 eq.) were added to the mixture successively. The reaction mixture was stirred at ambient temperature until substrate **1** disappeared on TLC. Then the mixture was diluted with 5 mL DCM, PCC (275 mg, 5.0 eq.) and 100 mg silica gel were added to the mixture. The mixture was stirred at 40 ^o^C for 12 h and the final product was achieved by flash column chromatography.

### Preparation of product 7

In a dried Schlenk tube under N_2_, [Ir(cod)Cl]_2_ (0.005 mmol, 3.35 mg) and chiral ligand **(*R*)-L** or **(*S*)-L1** (0.02 mmol, 10.15 mg) were mixed in 0.3 mL DCE and stirred at ambient temperature for 20 min under argon atmosphere. Then allylic alcohol **1** (0.25 mmol, 1.0 eq), ketone **2** (0.5 mmol, 2.0 eq) and chiral amine catalyst **(*S*)-A4** (0.05 mmol, 16.1 mg) were added to the mixture successively. After 8 h, the other 3.0 eq. ketone **2** was added in portions. Stirring at 50 ^o^C for 48 h. The residue was directly purified by flash silica gel chromatography to afford the title compound.

## Supplementary information

Supplementary Information

## Data Availability

The authors declare that the data supporting the findings of this study are available within the article and Supplementary Information file, or from the corresponding author upon reasonable request. The X-ray crystallographic coordinates for structures reported in this study have been deposited at the Cambridge Crystallographic Data Centre (CCDC), under deposition numbers CCDC 1821791 and CCDC 1869868. These data can be obtained free of charge from The Cambridge Crystallographic Data Centre via www.ccdc.cam.ac.uk/data_request/cif.
