# Supplementary material for: Synergetic iridium and amine catalysis enables asymmetric [4+2] cycloadditions of vinyl aminoalcohols with carbonyls
Source: Nat Commun. 2019 Jun 20;10:2716. doi: 10.1038/s41467-019-10674-3 (PMC6586609; doi:10.1038/s41467-019-10674-3)
Supplement: Supplementary file 1 — Supplementary Information [file 41467_2019_10674_MOESM1_ESM.pdf]

Supplementary Information

Synergetic iridium and amine catalysis enables asymmetric [4+2] cycloadditions of vinyl aminoalcohols with carbonyls

Zhang et al

Supplementary Figures

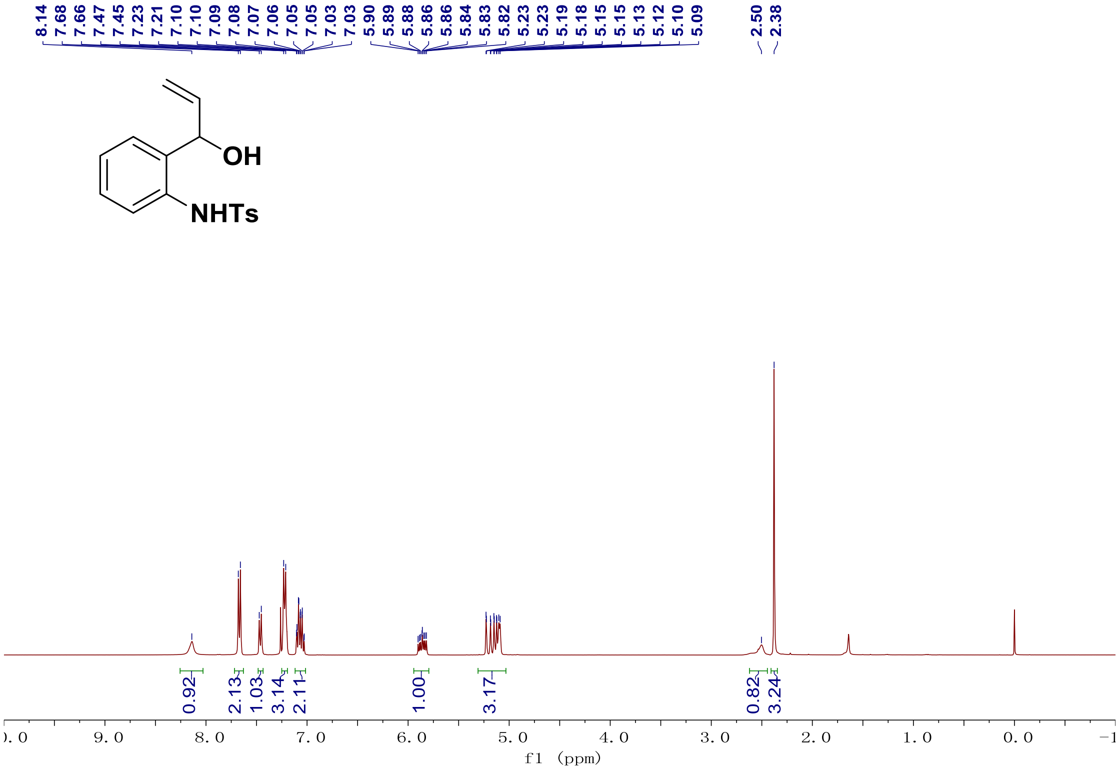

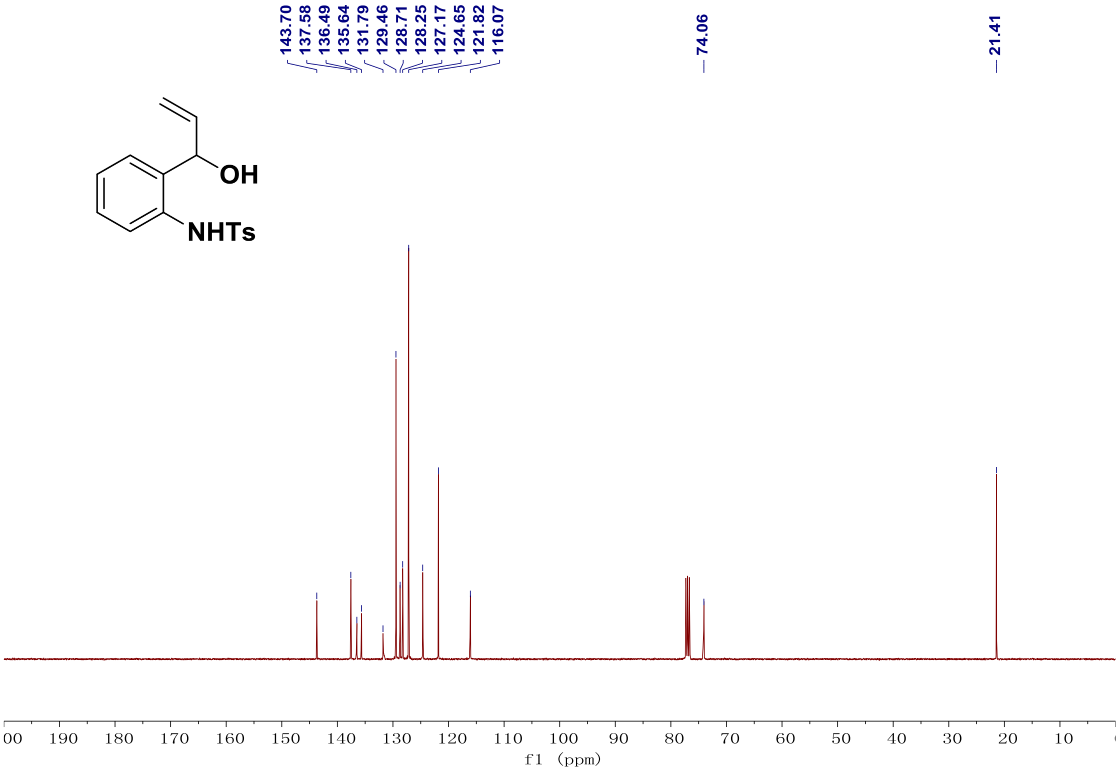

Supplementary Figure 1. ^{1}H and ^{13}C NMR of compound **1a**.

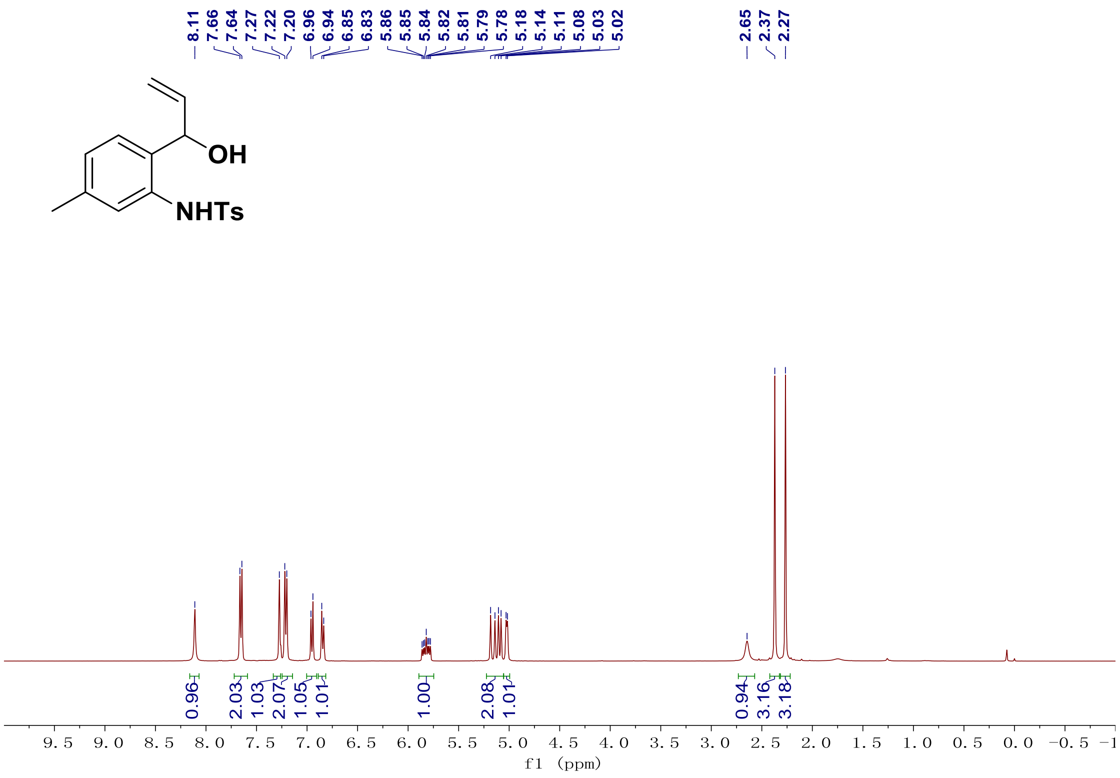

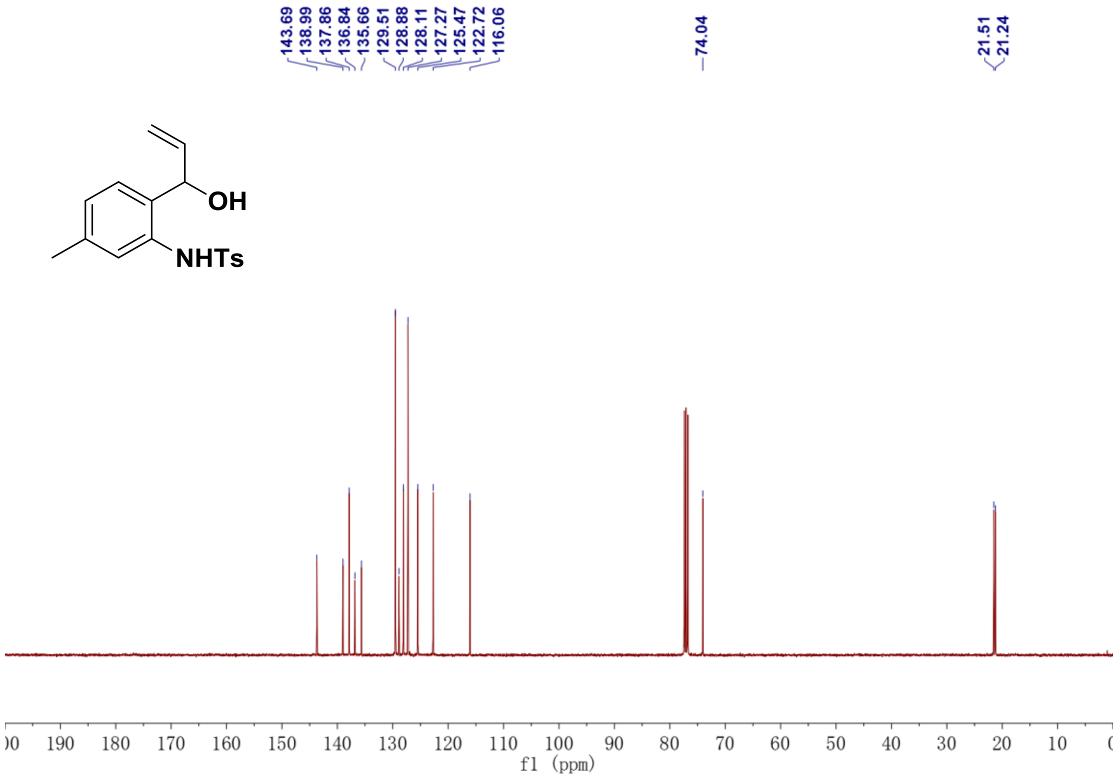

Supplementary Figure 2. ^{1}H and ^{13}C NMR of compound **1b**.

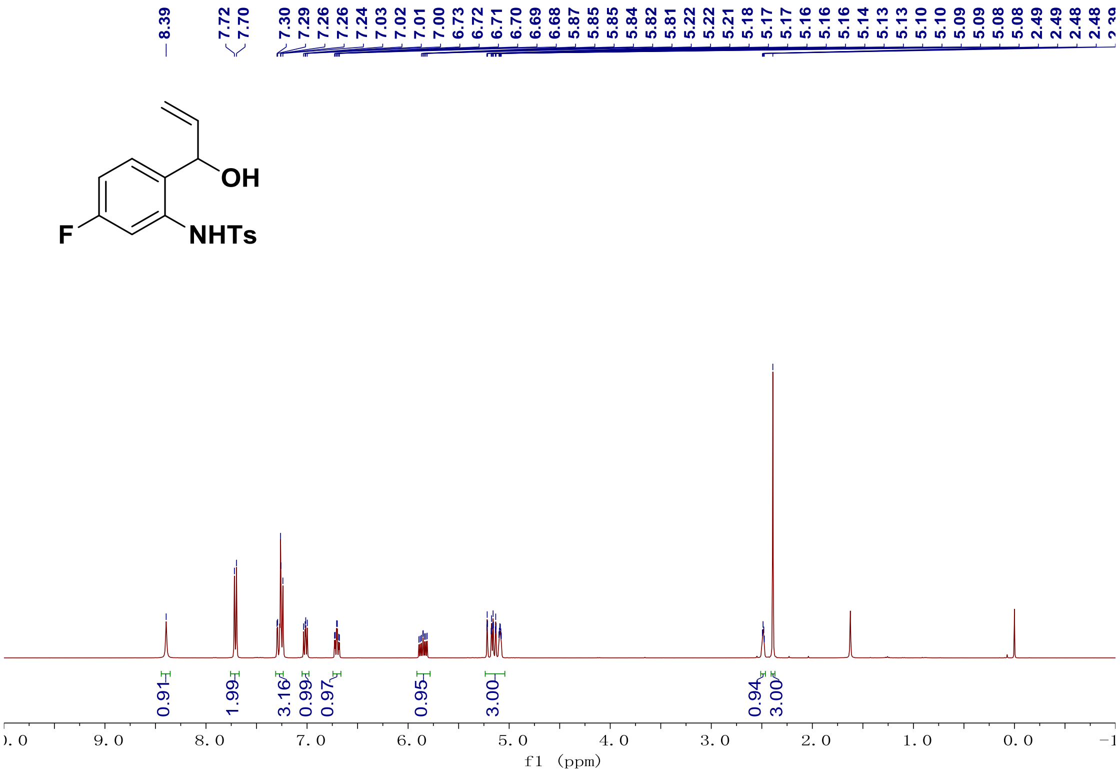

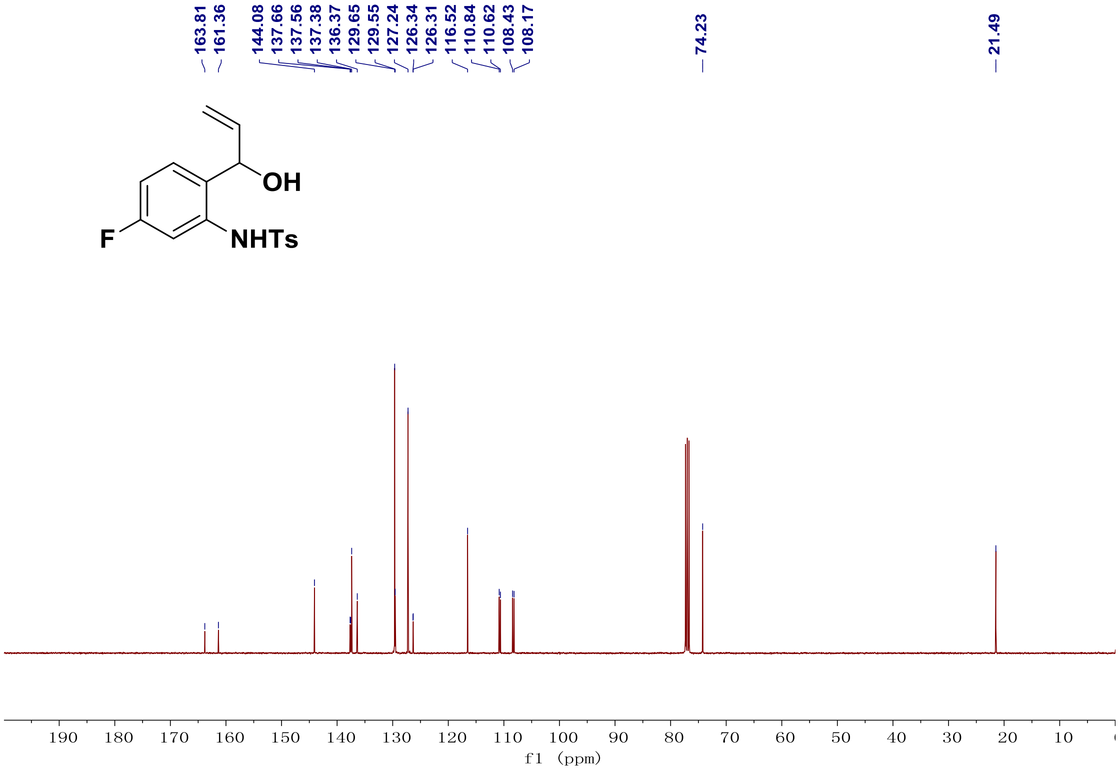

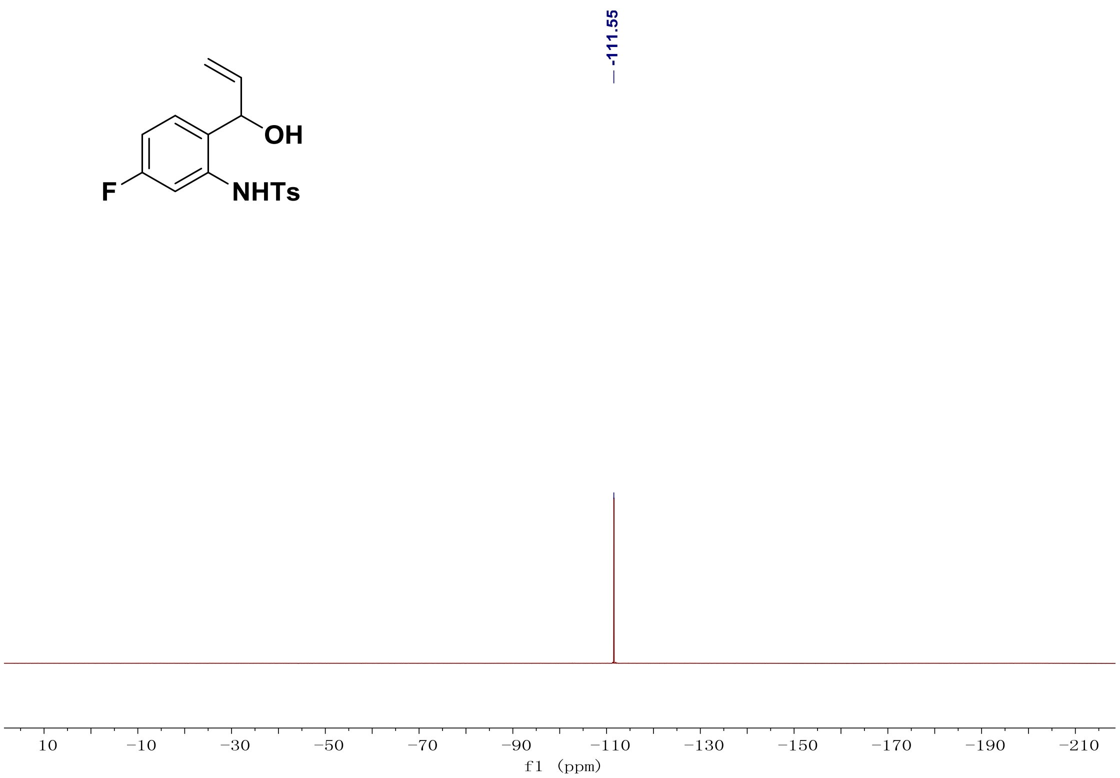

Supplementary Figure 3. ^{1}H and ^{13}C NMR ^{19}F NMR of compound **1c**.

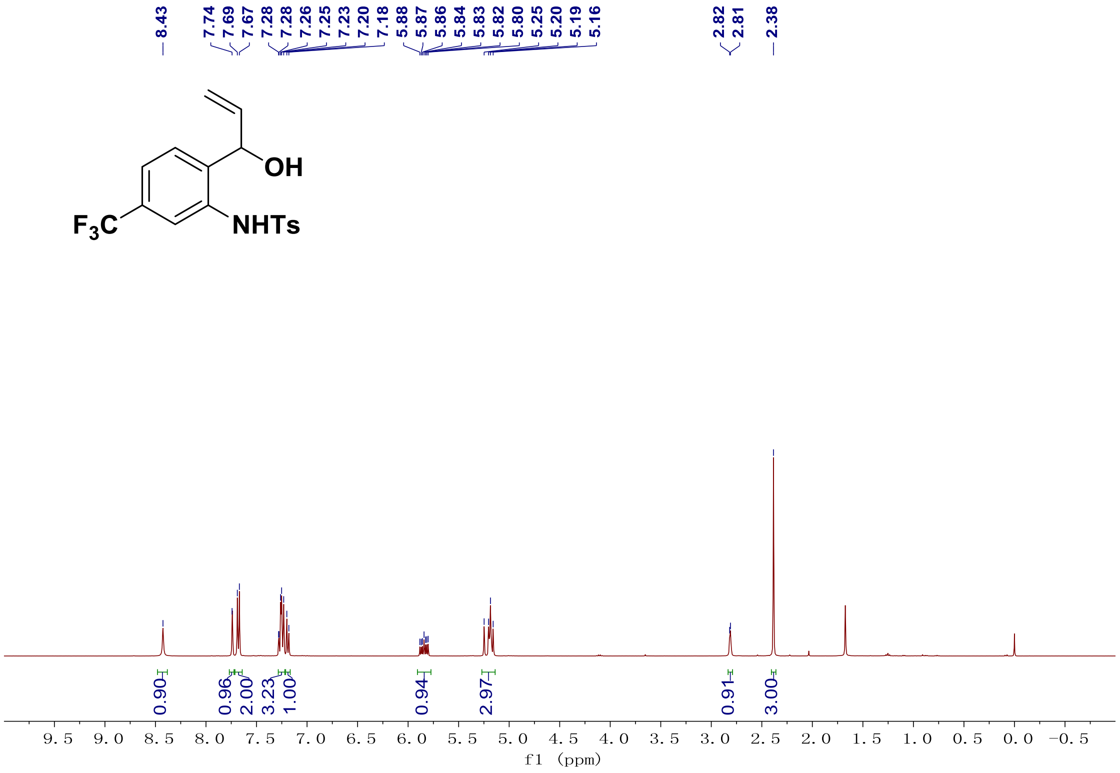

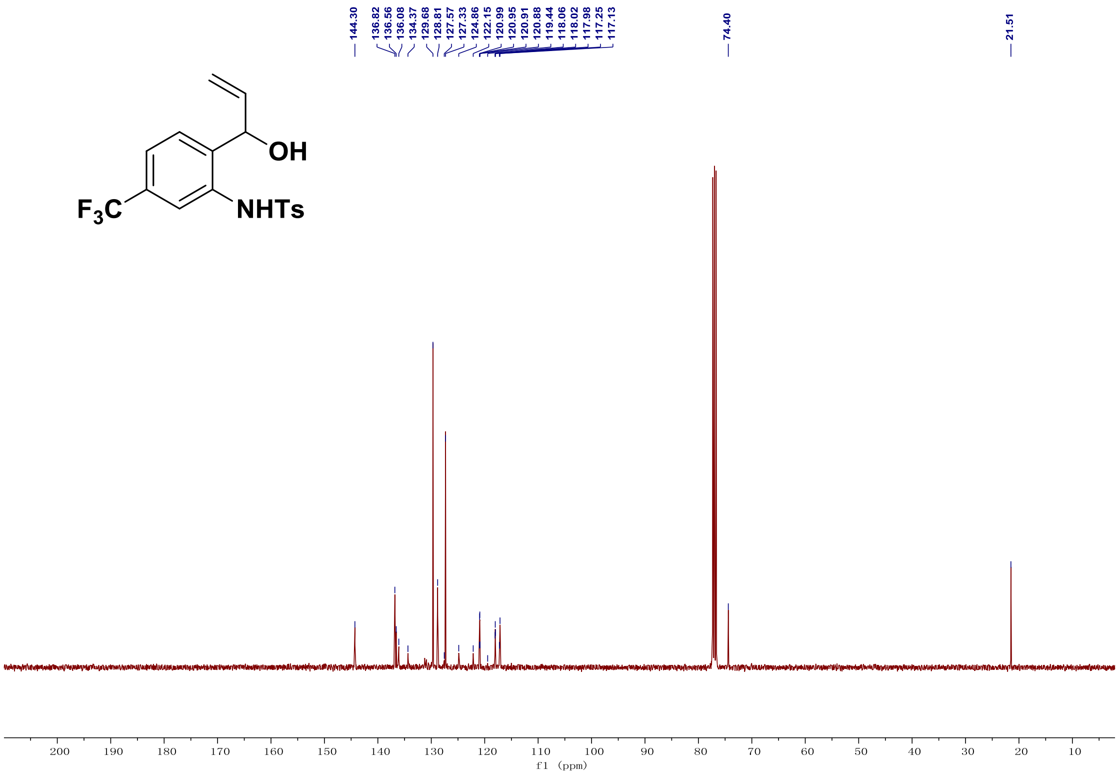

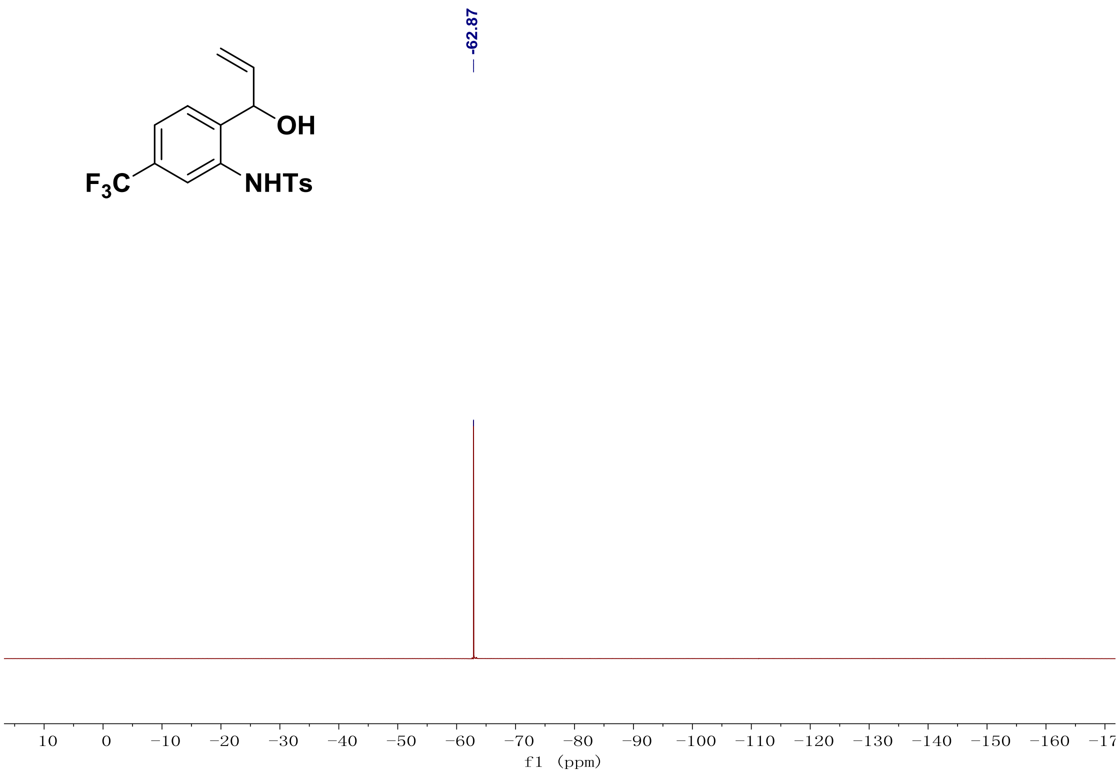

Supplementary Figure 4. ^{1}H and ^{13}C NMR ^{19}F NMR of compound **1d**.

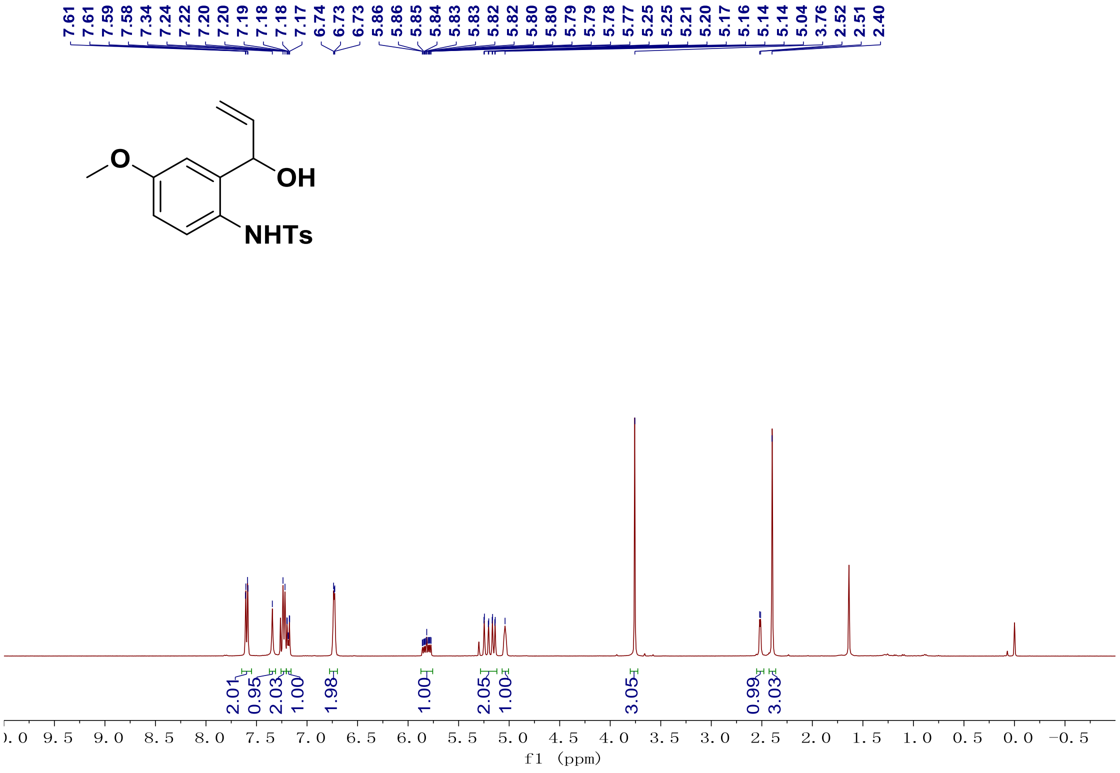

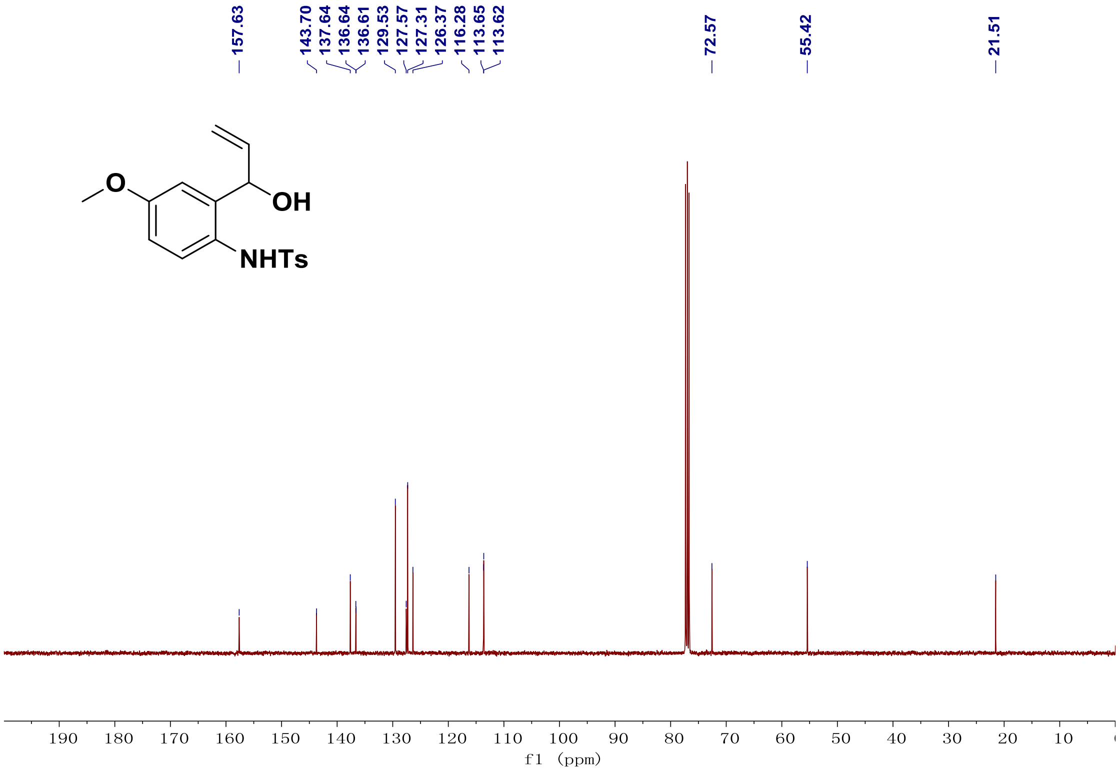

Supplementary Figure 5. ^{1}H and ^{13}C NMR of compound **1e**.

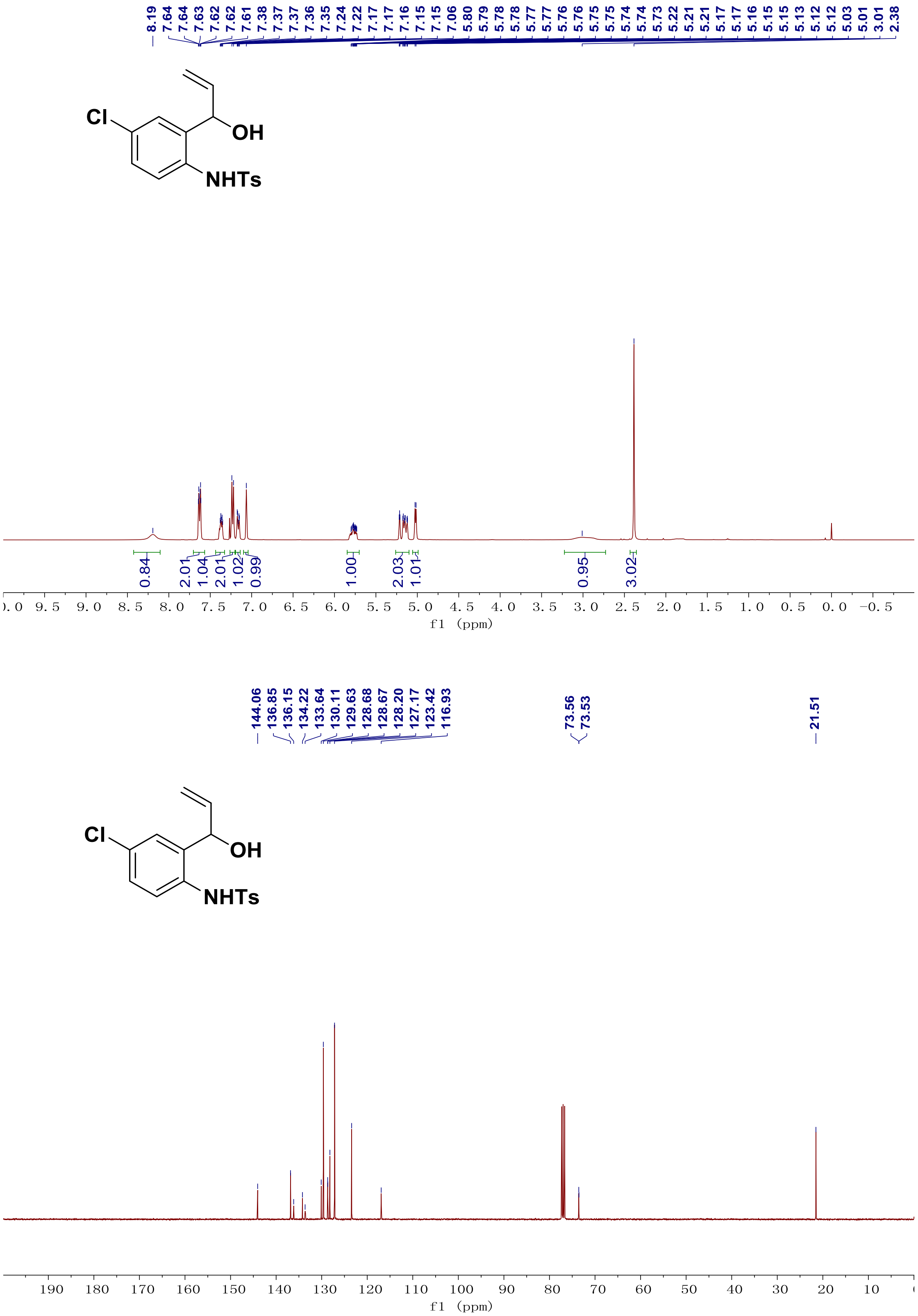

Supplementary Figure6. ^{1}H and ^{13}C NMR of compound **1f**.

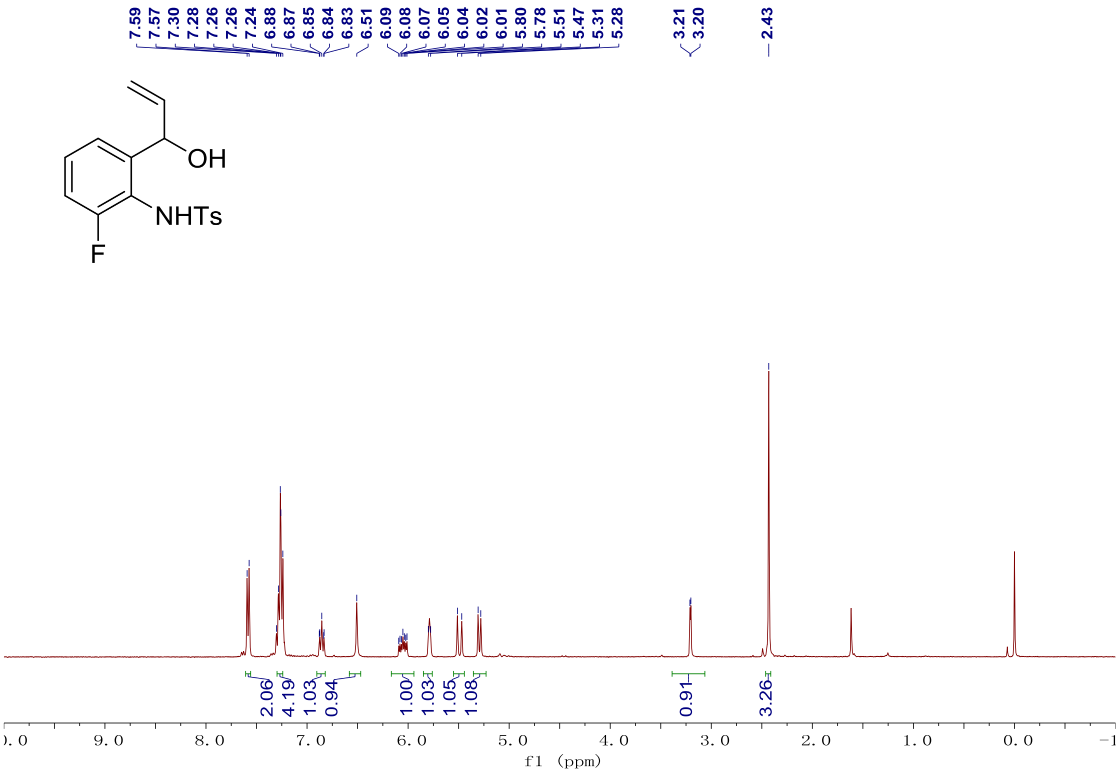
7.59 7.57 7.30 7.28 7.26 7.26 7.24 6.88 6.87 6.85 6.84 6.83 6.51 6.09 6.08 6.07 6.05 6.04 6.02 6.01 5.80 5.78 5.51 5.47 5.31 5.28
3.21 3.20
2.43
OH
NHTs
F
2.06 4.19 1.03 0.94 1.00 1.03 1.05 1.08 0.91 3.26
f1 (ppm)

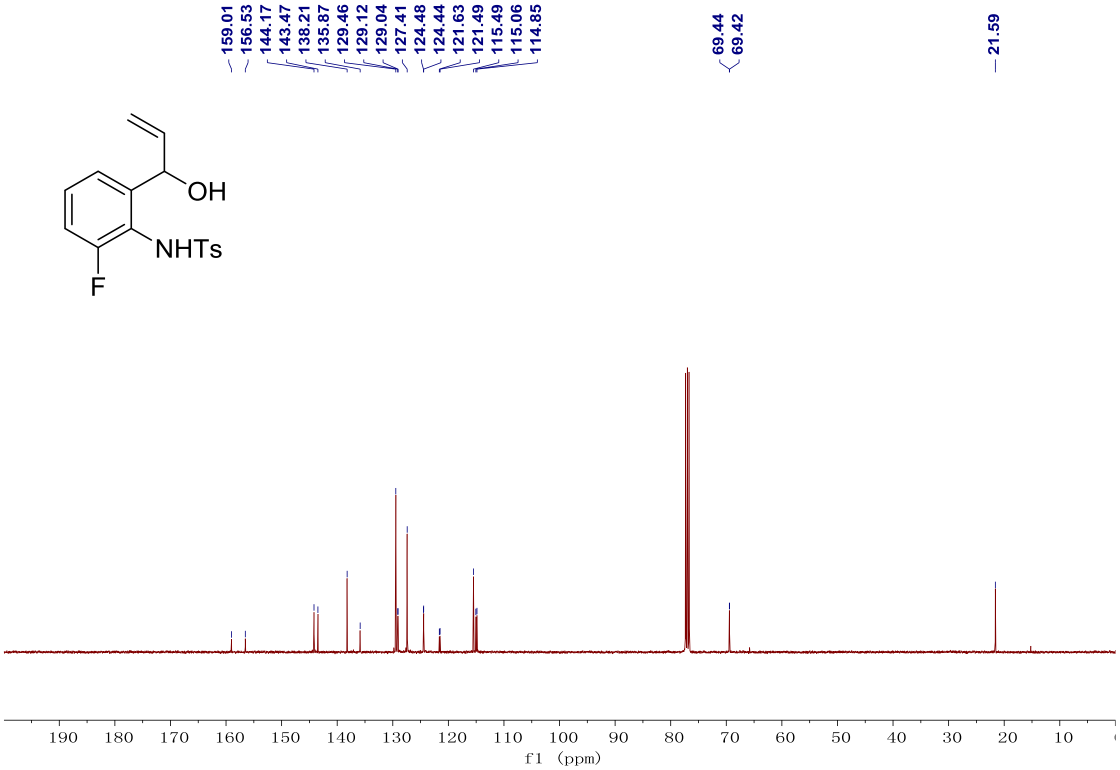
159.01 156.53 144.17 143.47 138.21 135.87 129.46 129.12 129.04 127.41 124.48 124.44 121.63 121.49 115.49 115.06 114.85
69.44 69.42
21.59
OH
NHTs
F
f1 (ppm)

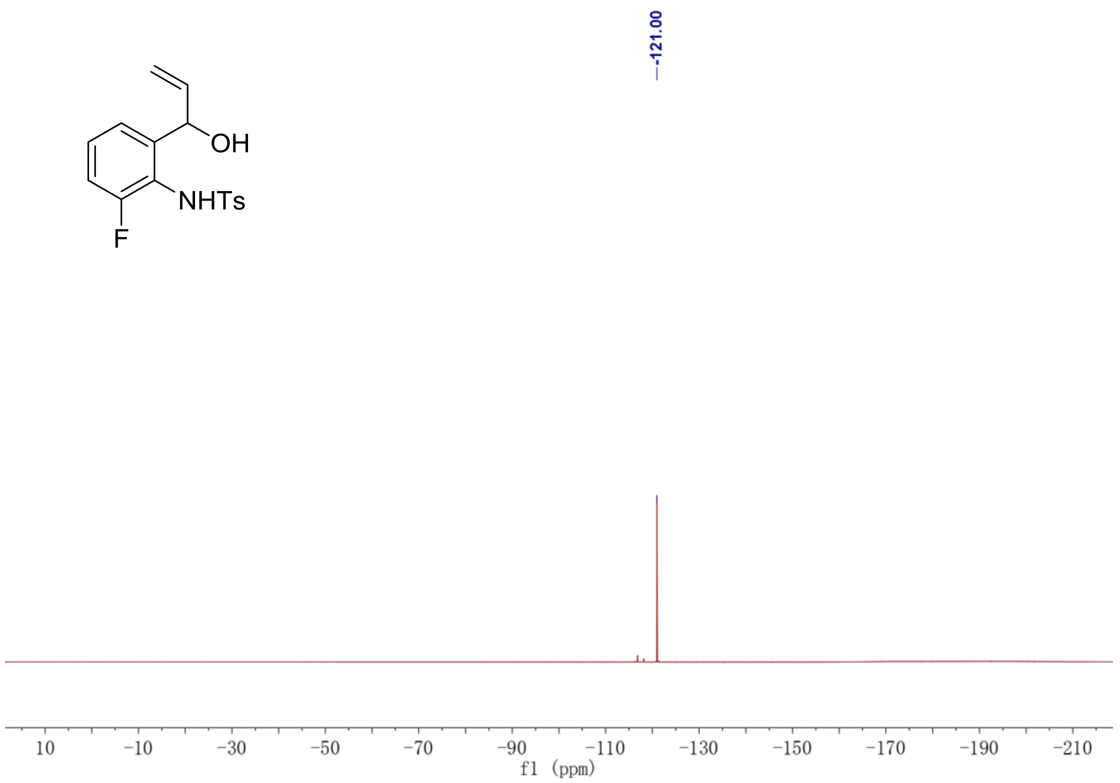

Supplementary Figure 7. ^{1}H and ^{13}C NMR ^{19}F NMR of compound **1g**.

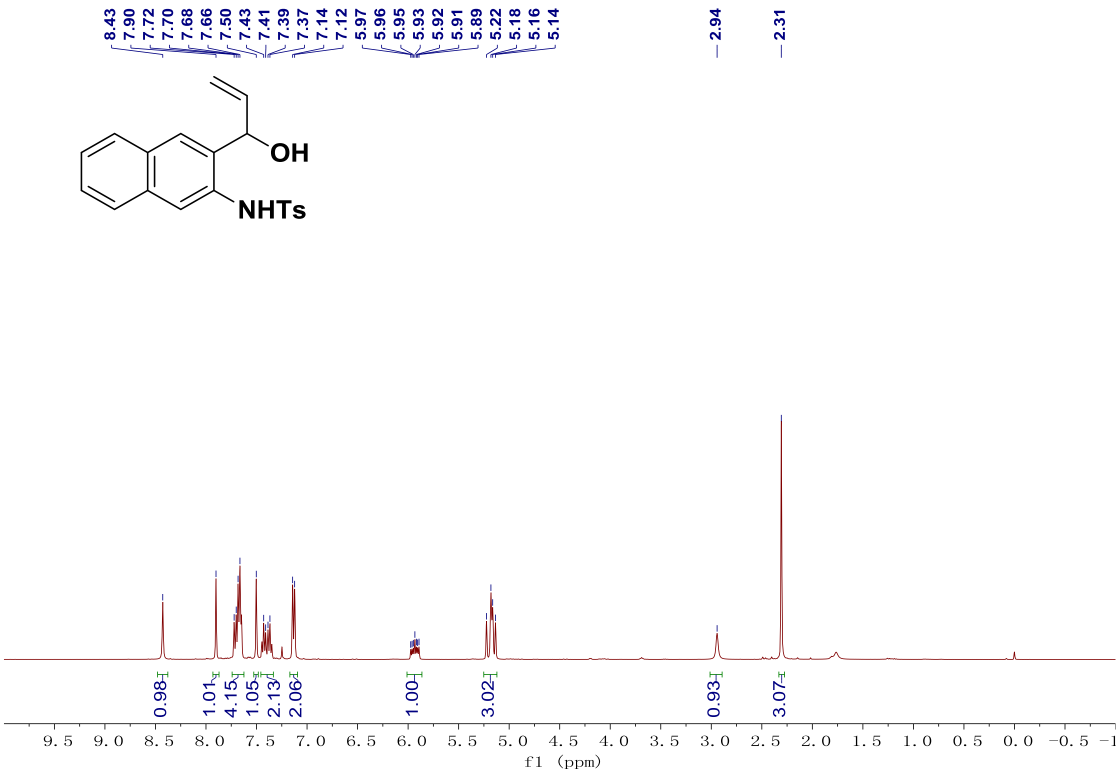

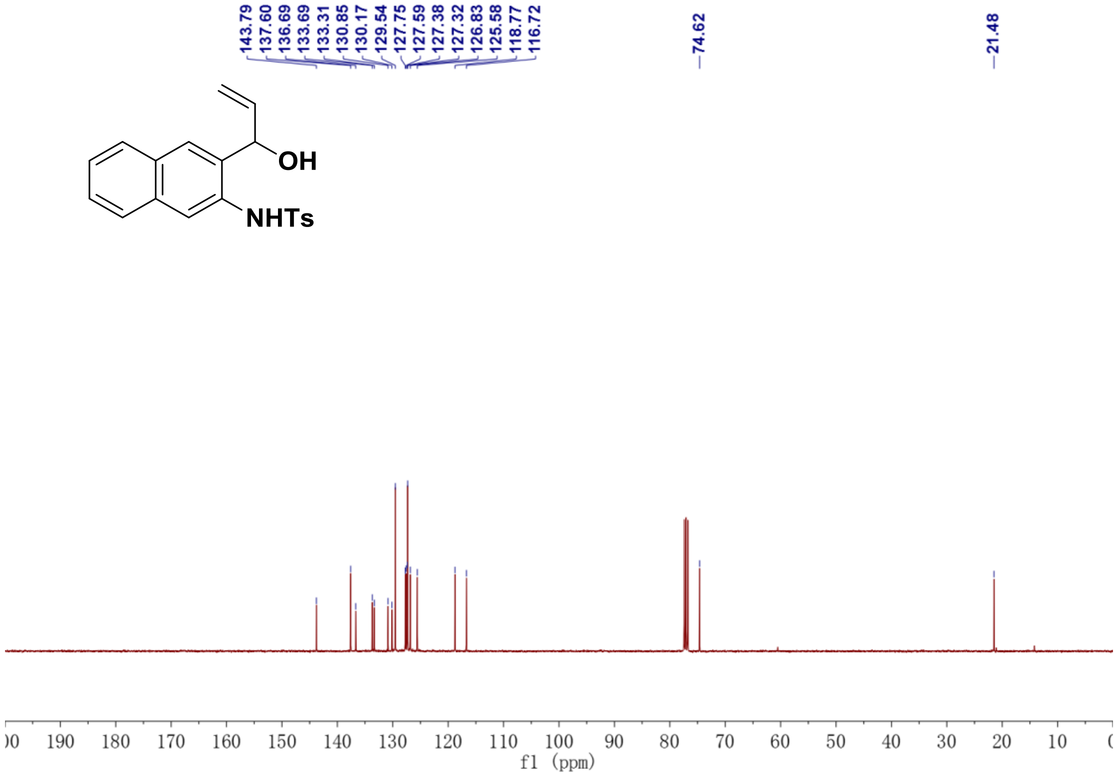

Supplementary Figure 8. ^{1}H and ^{13}C NMR of compound **1h**.

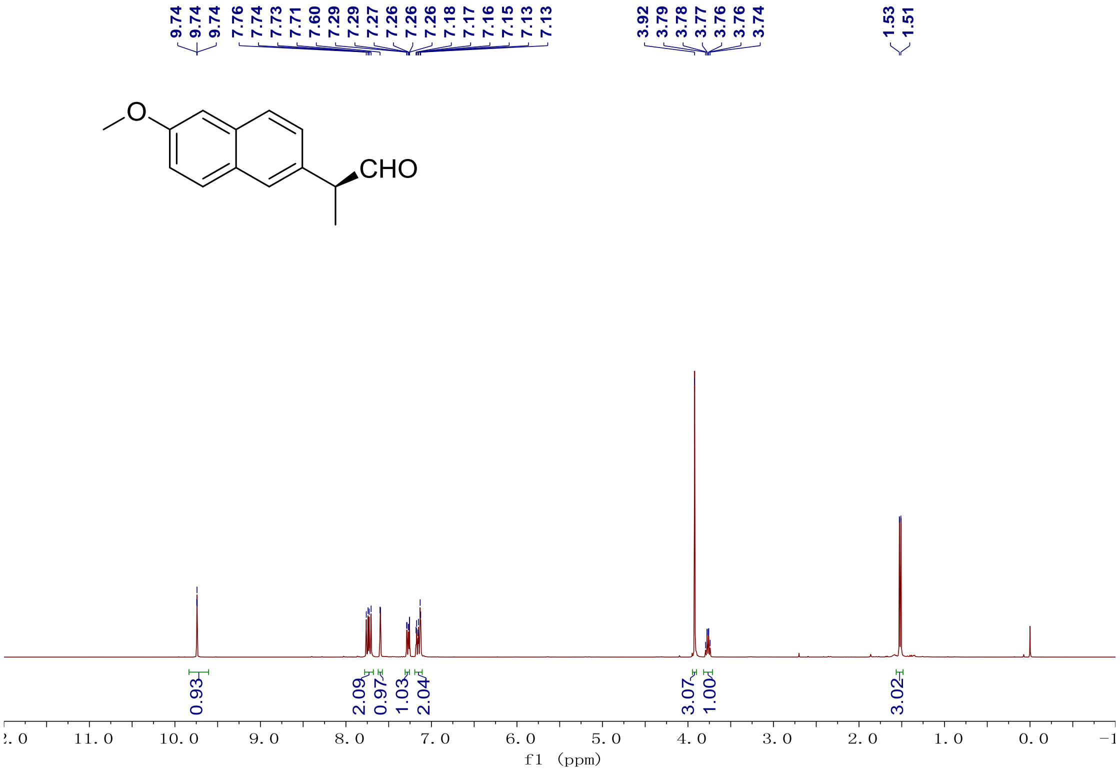

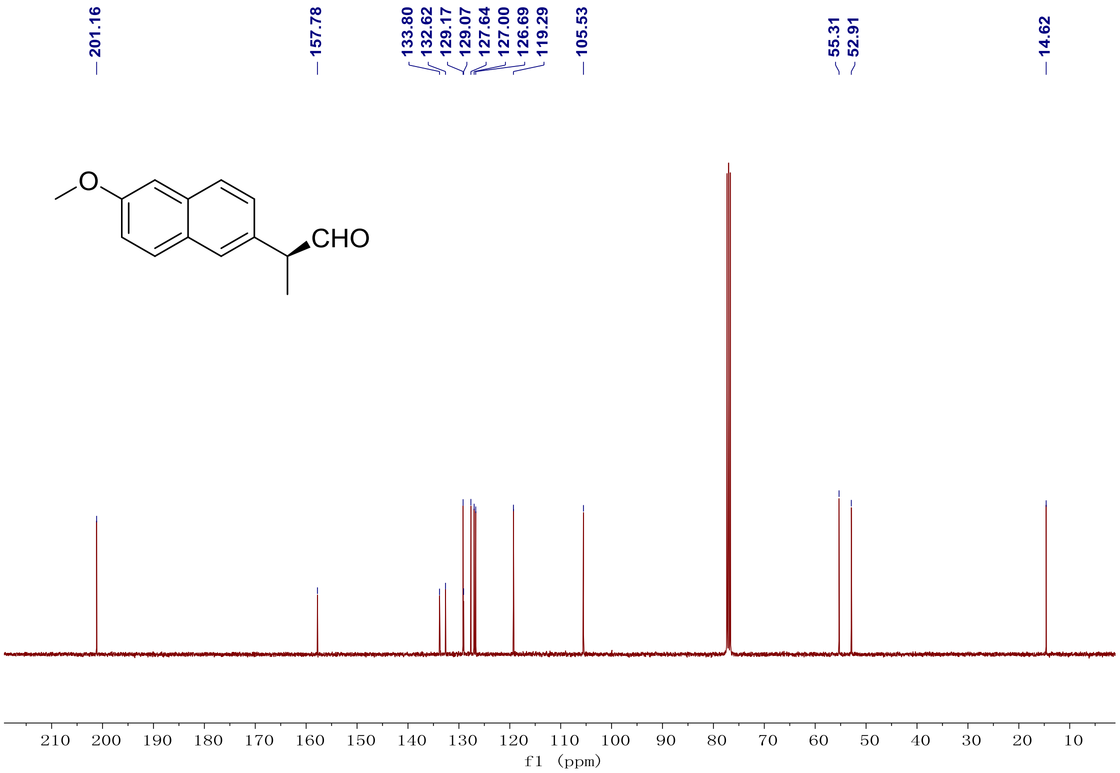

Supplementary Figure 9. ¹H and ¹³C NMR of compound **2k**.

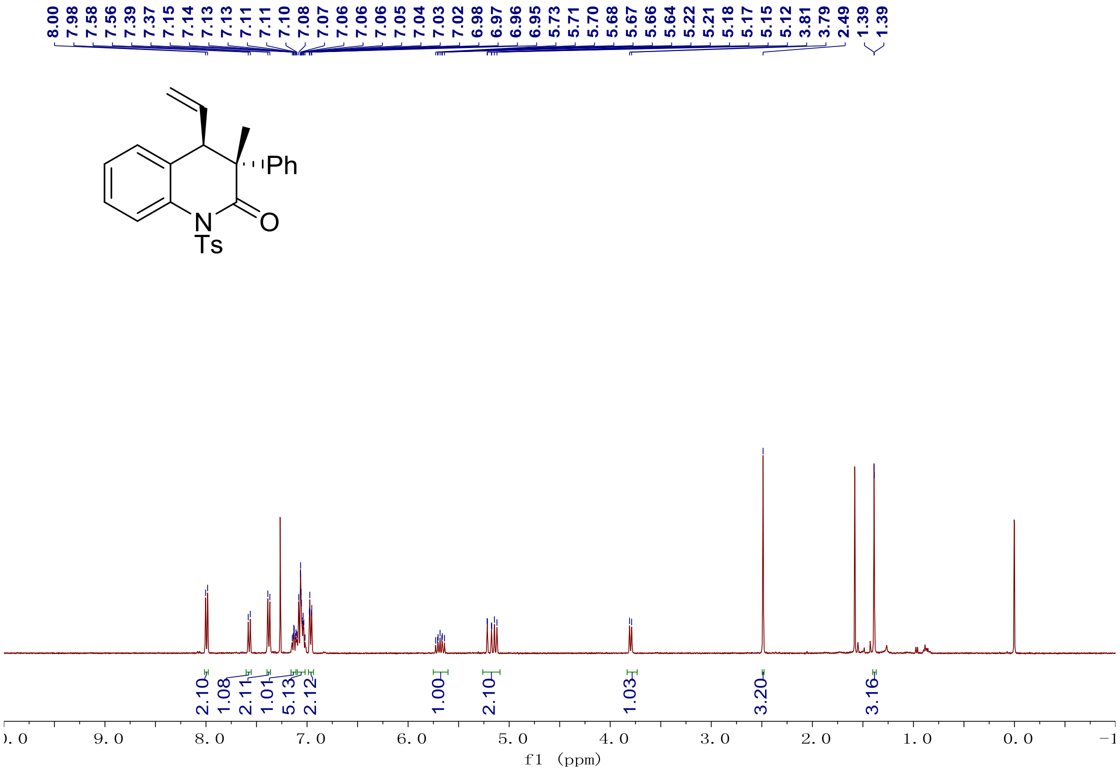

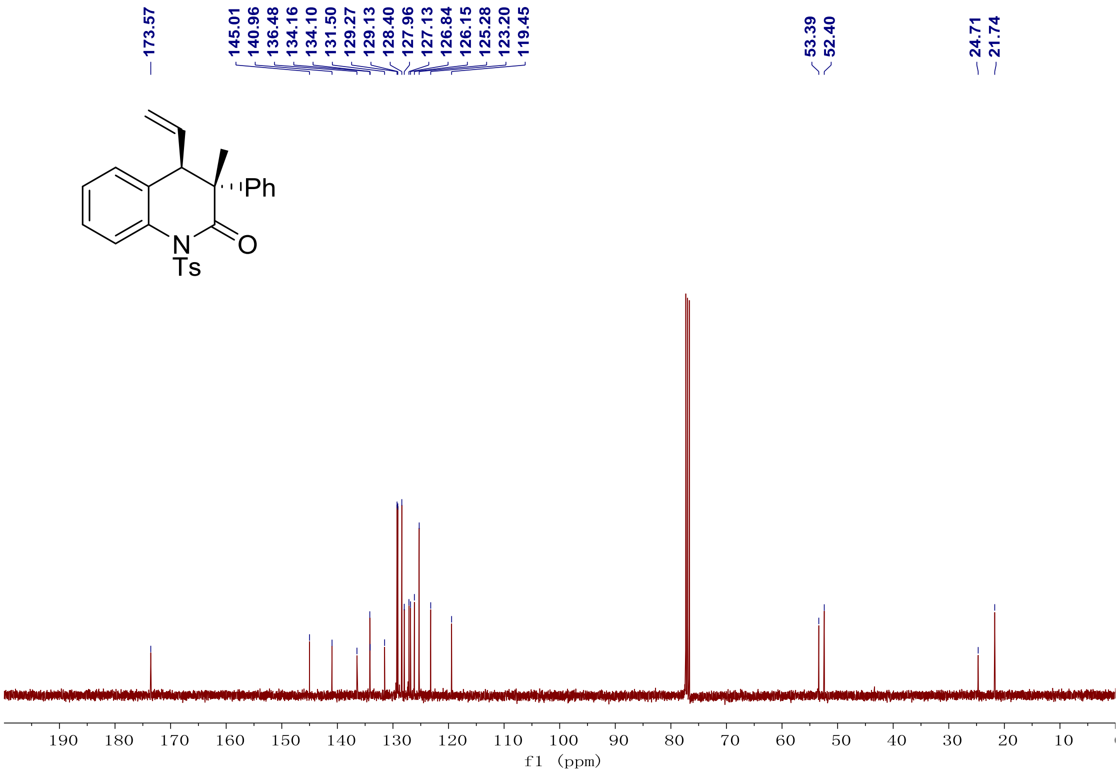

Supplementary Figure 10. ^{1}H and ^{13}C NMR of compound **3aa**

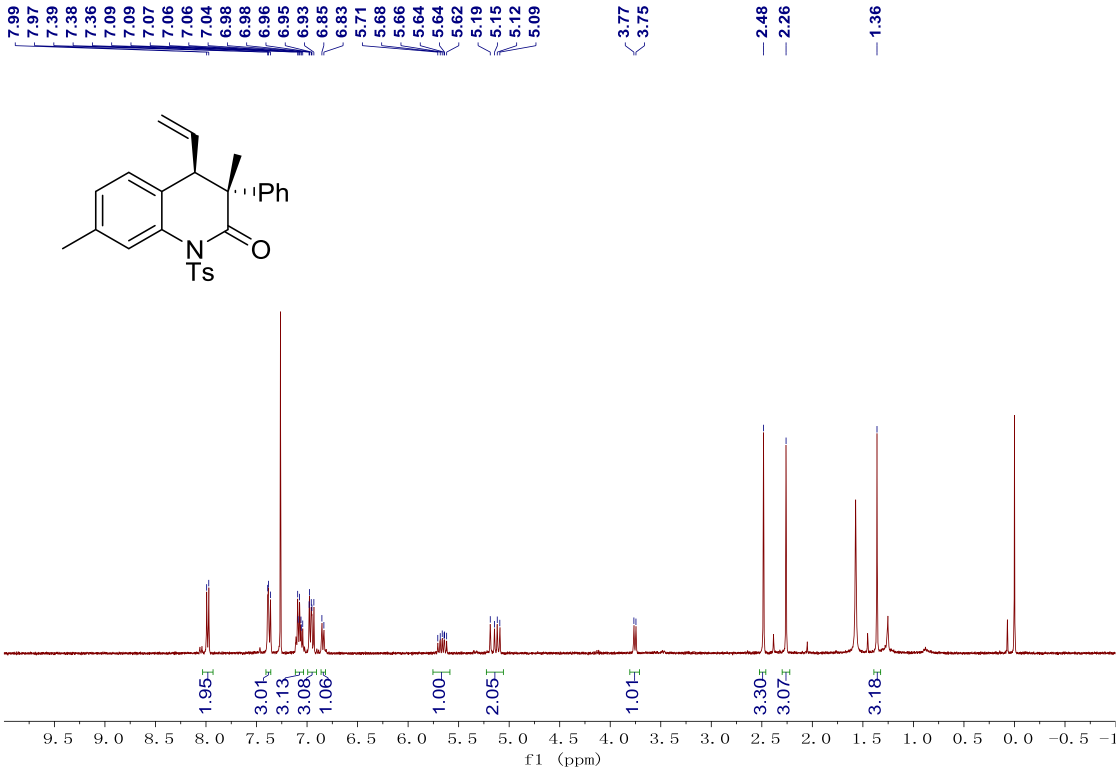

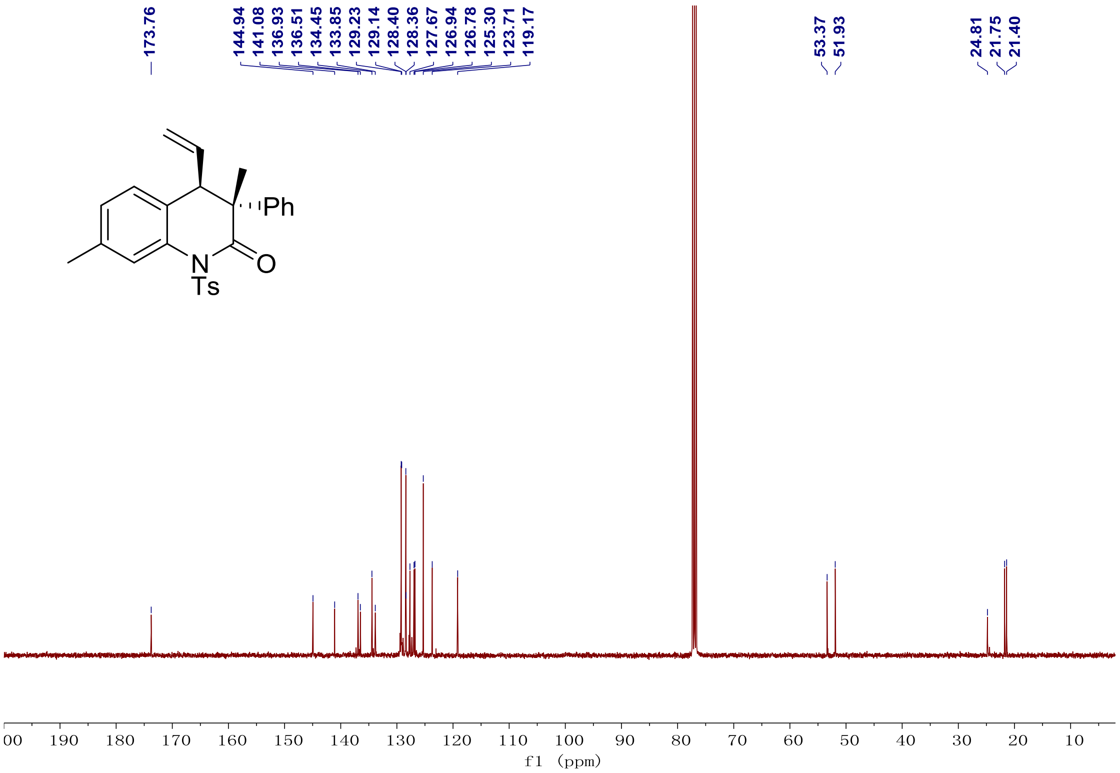

Supplementary Figure 11. ^{1}H and ^{13}C NMR of compound **3ba**

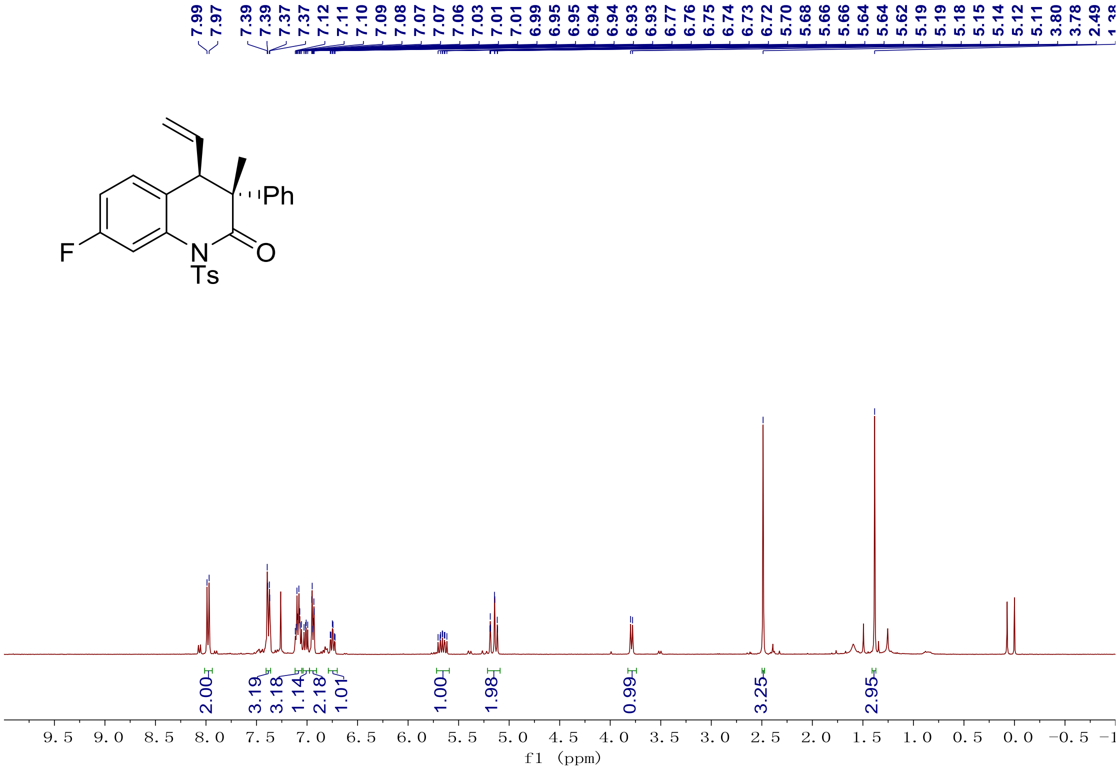

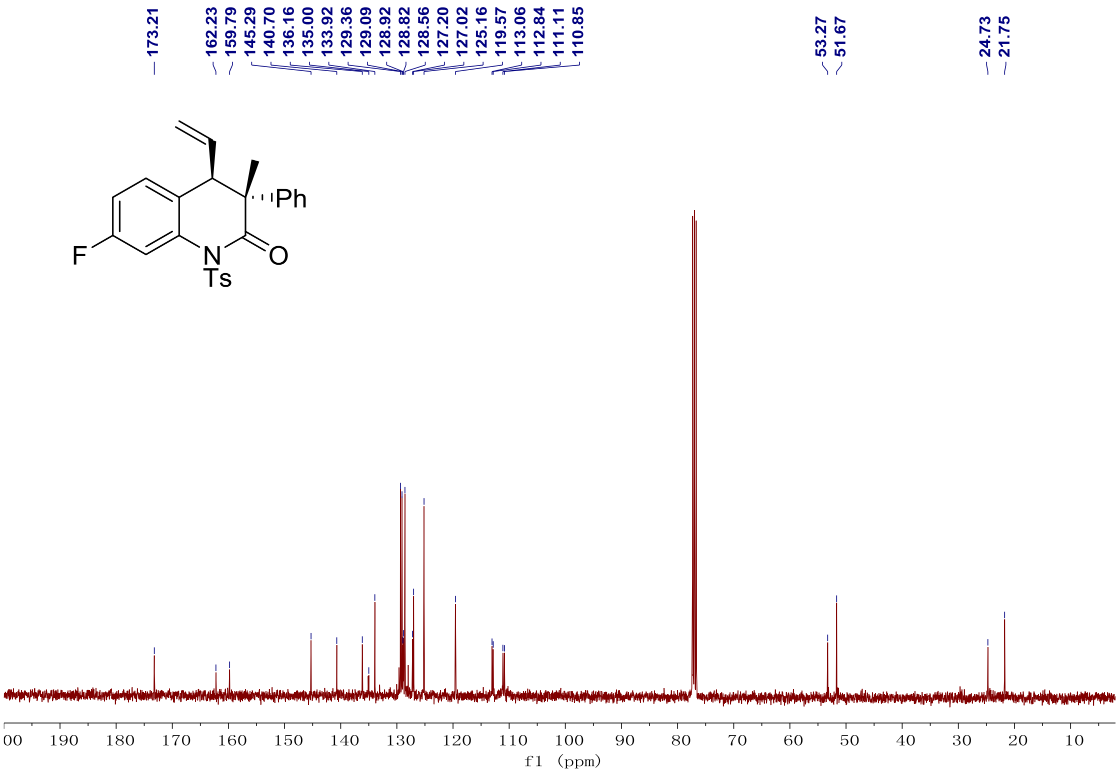

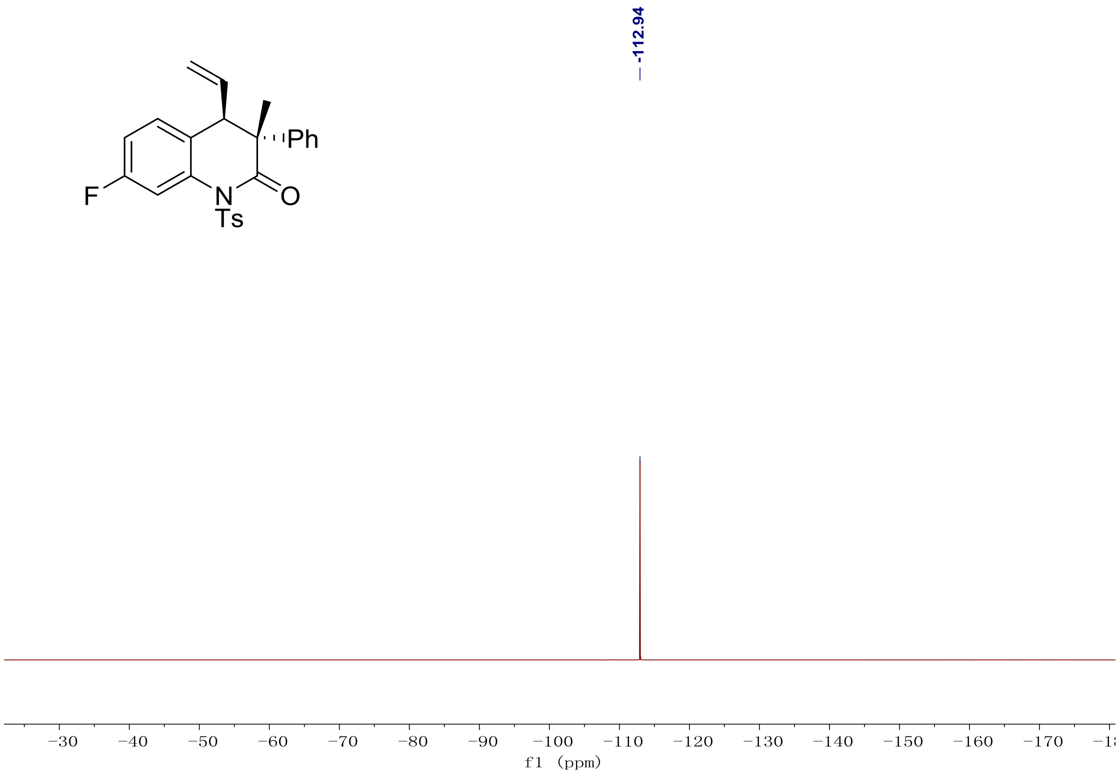

Supplementary Figure 12. ^{1}H and ^{13}C NMR ^{19}F NMR of compound **3ca**

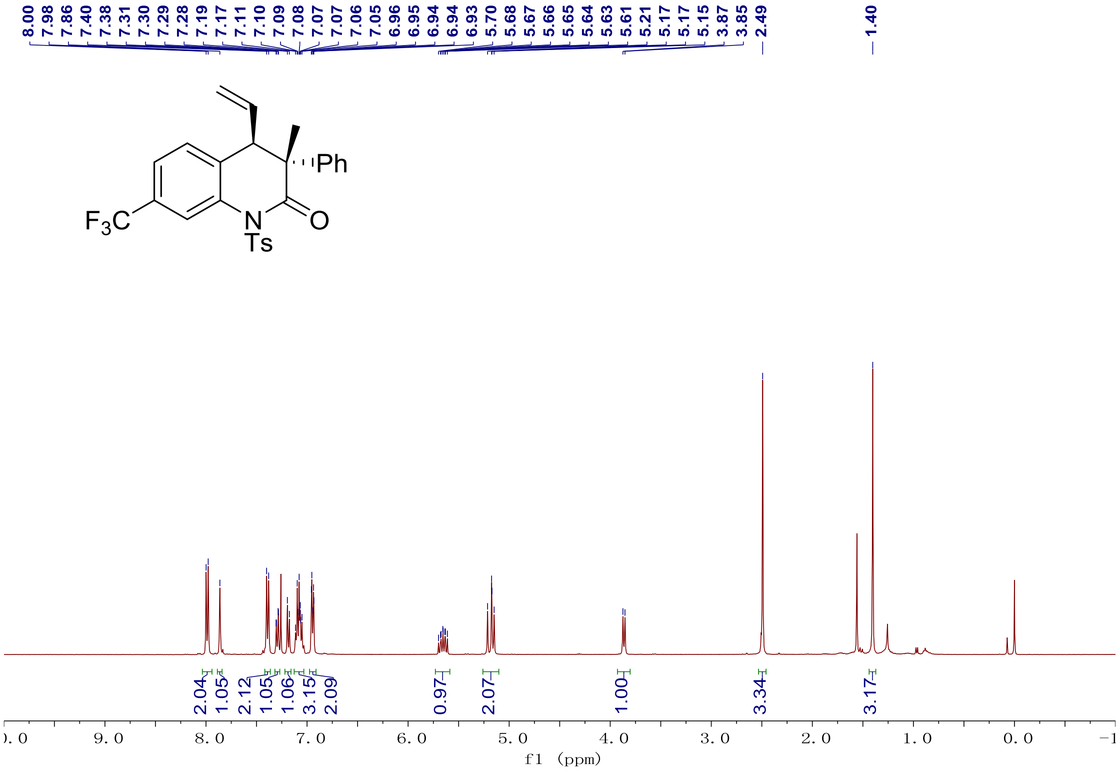

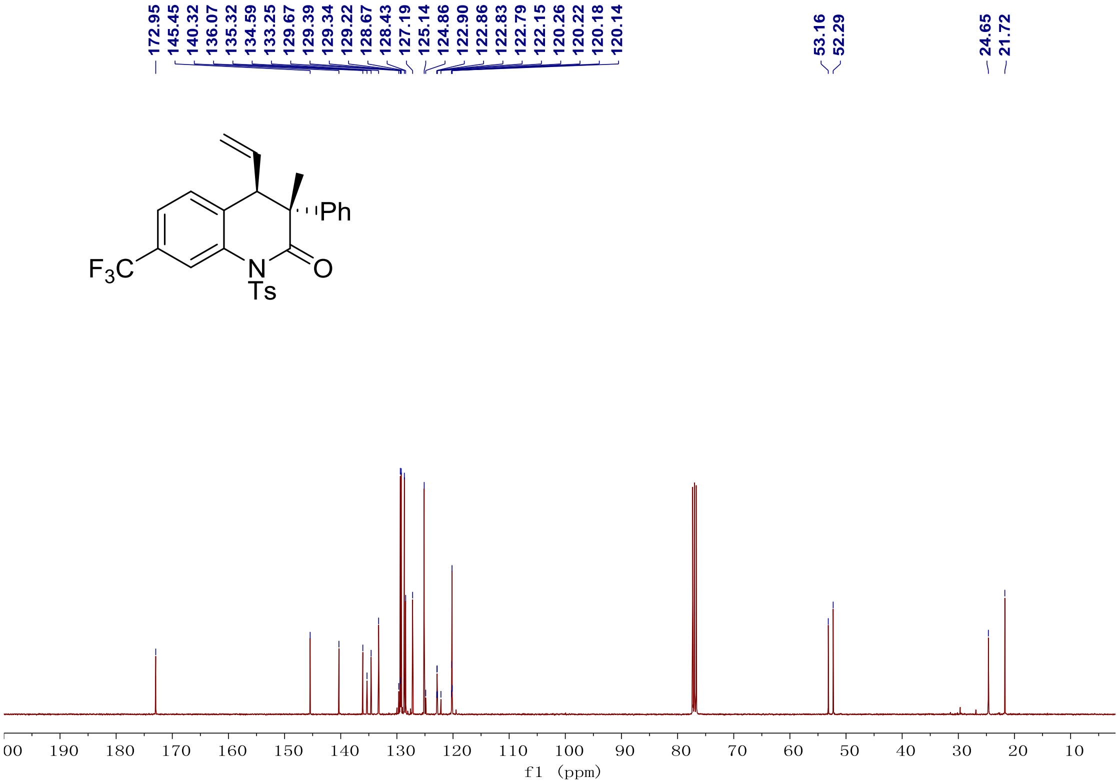

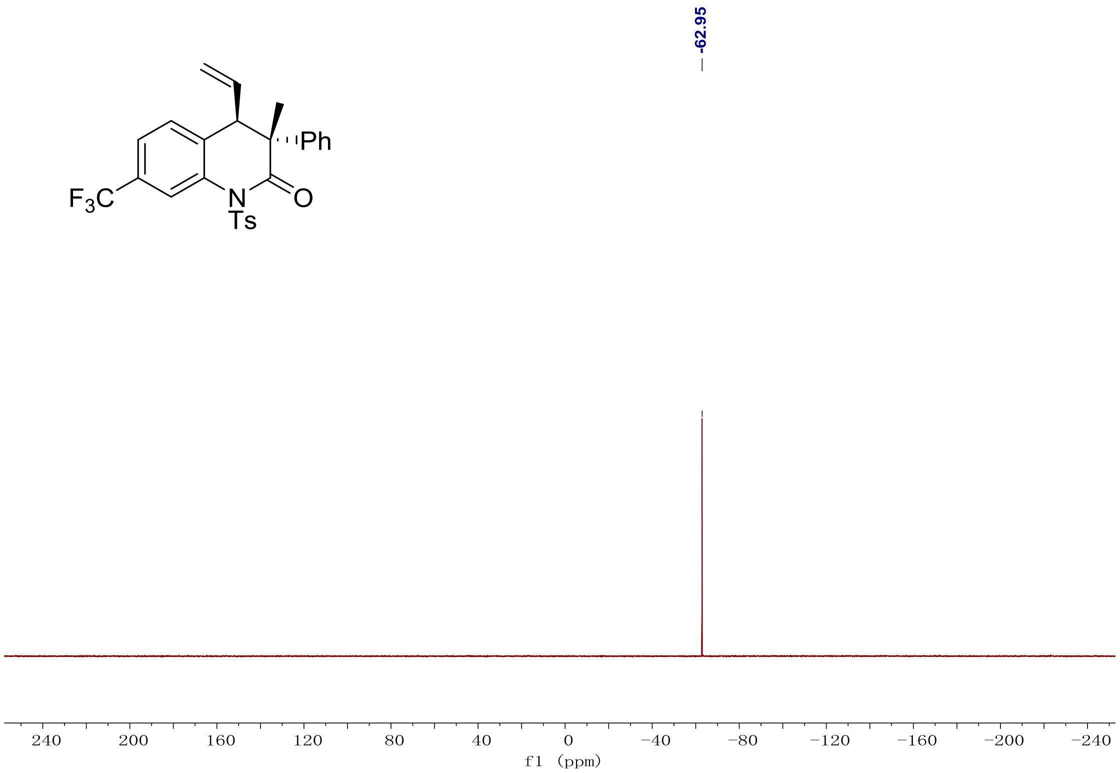

Supplementary Figure 13. ^{1}H and ^{13}C NMR ^{19}F NMR of compound **3da**

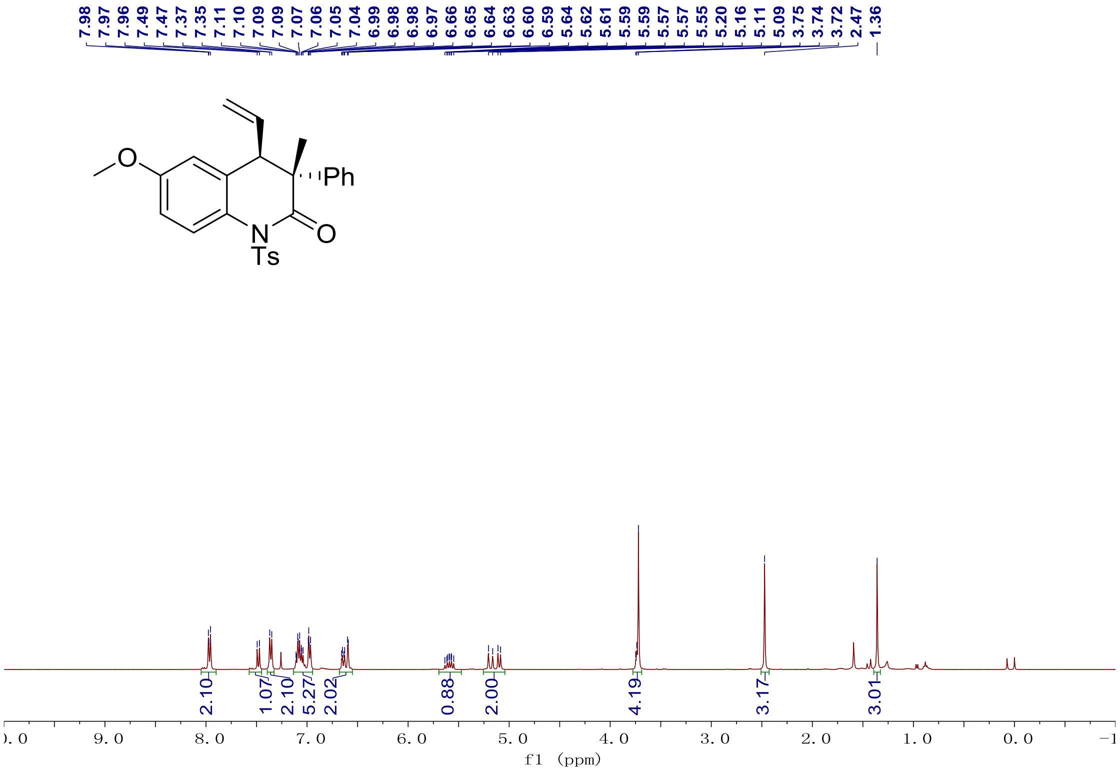

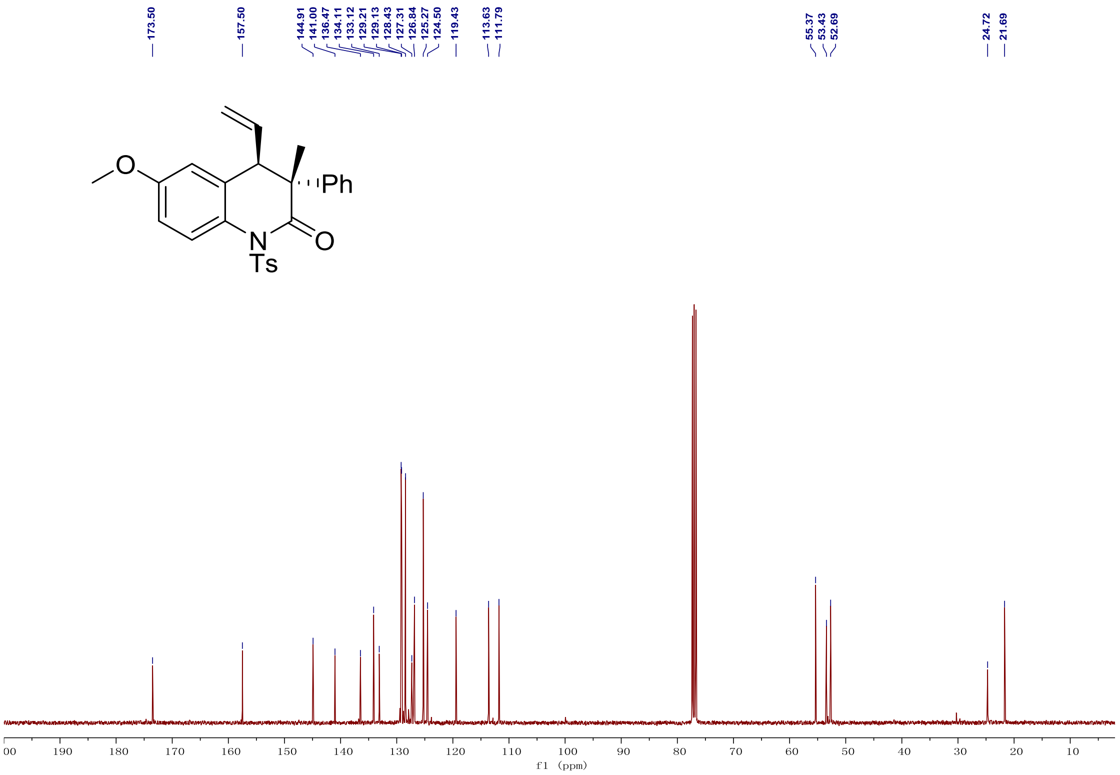

Supplementary Figure 14. ^{1}H and ^{13}C NMR of compound **3ea**

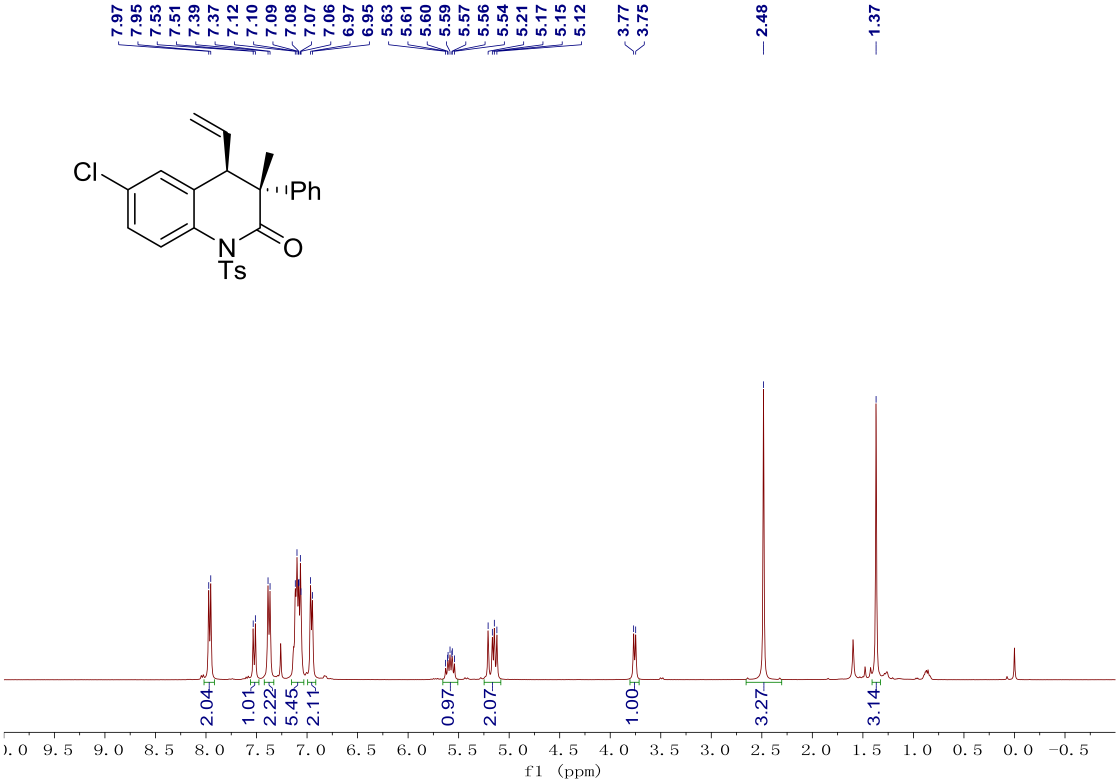

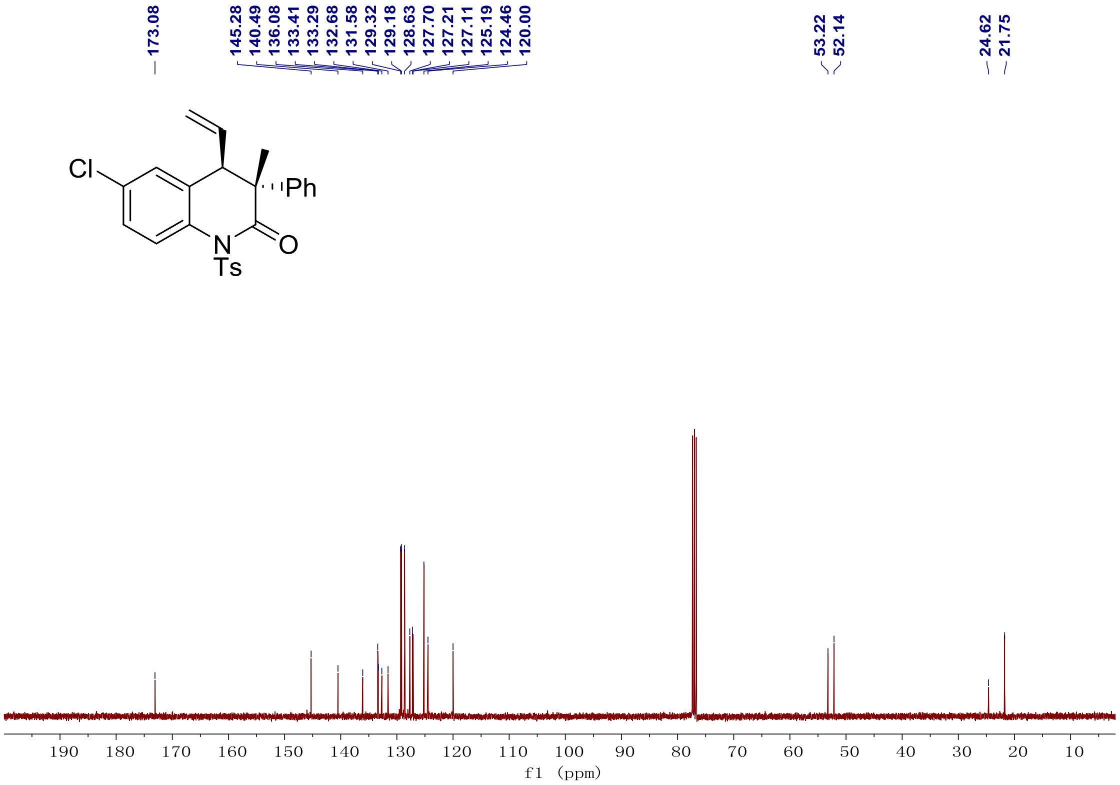

Supplementary Figure 15. ^{1}H and ^{13}C NMR of compound **3fa**

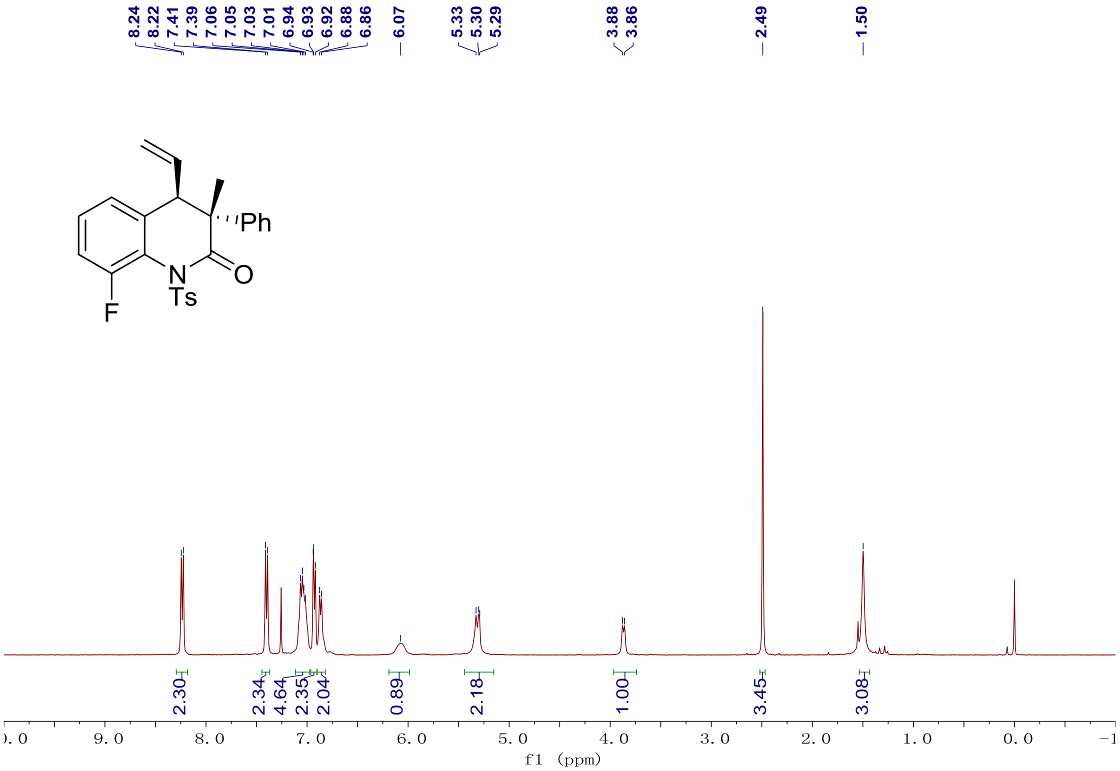

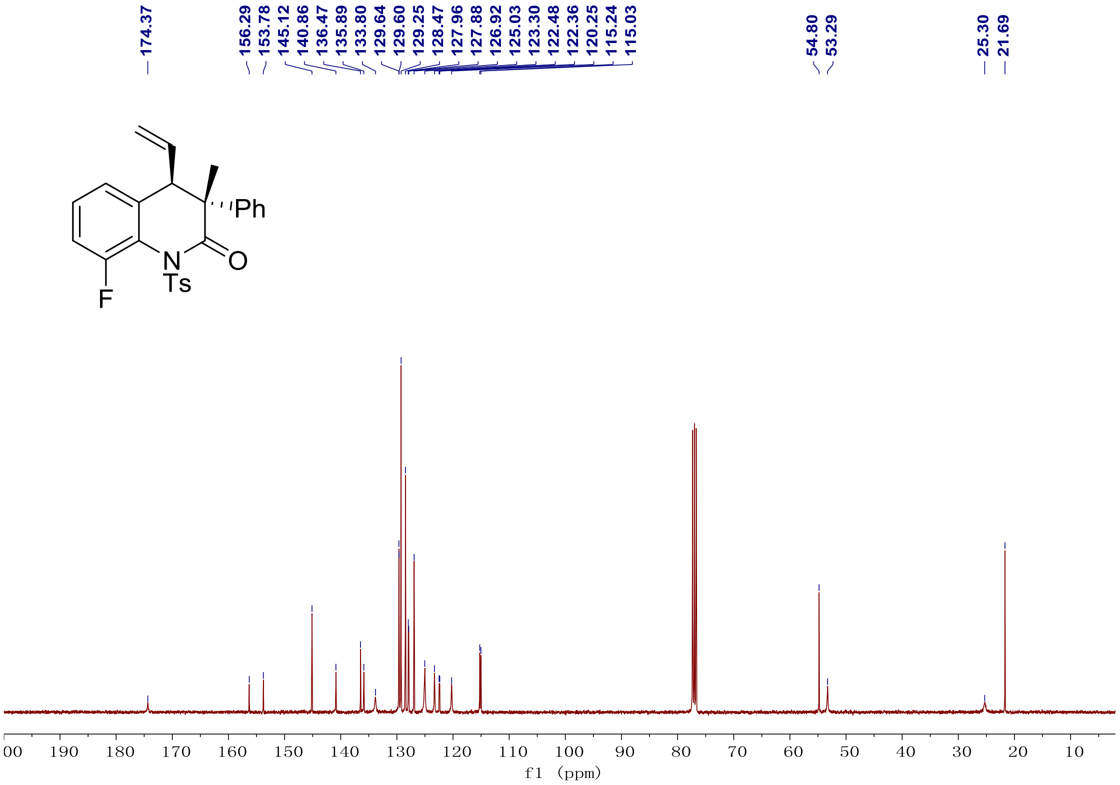

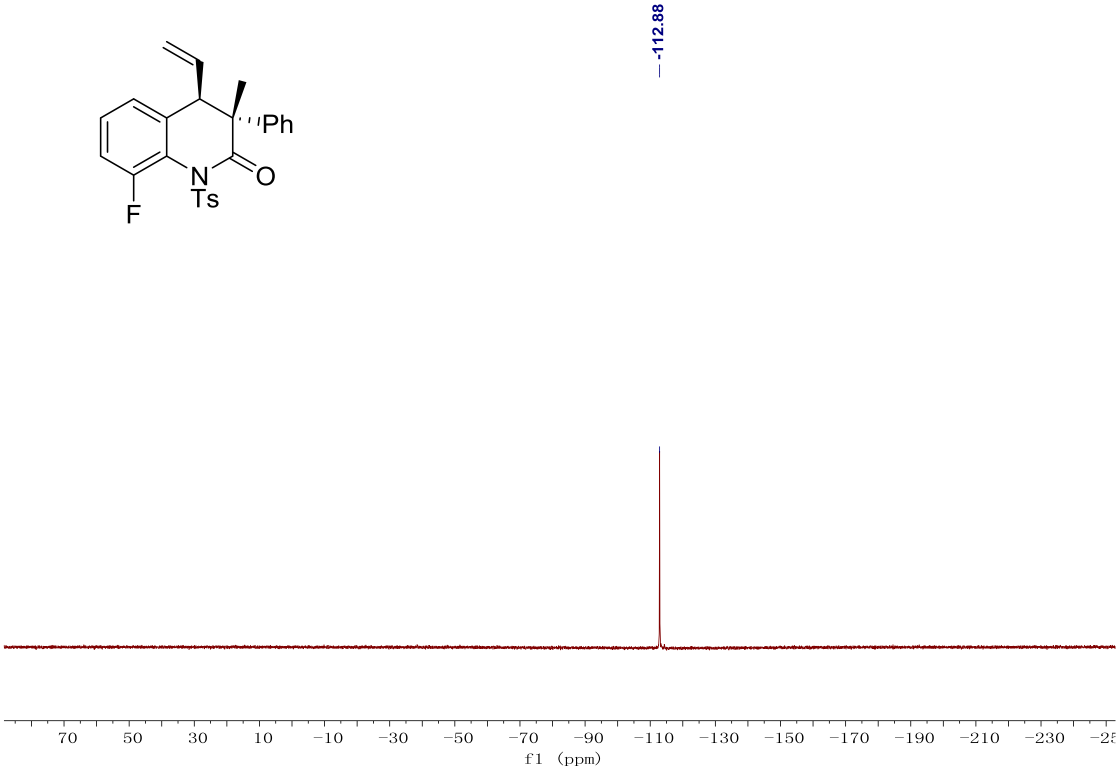

Supplementary Figure 16. ^{1}H and ^{13}C NMR ^{19}F NMRof compound **3ga**

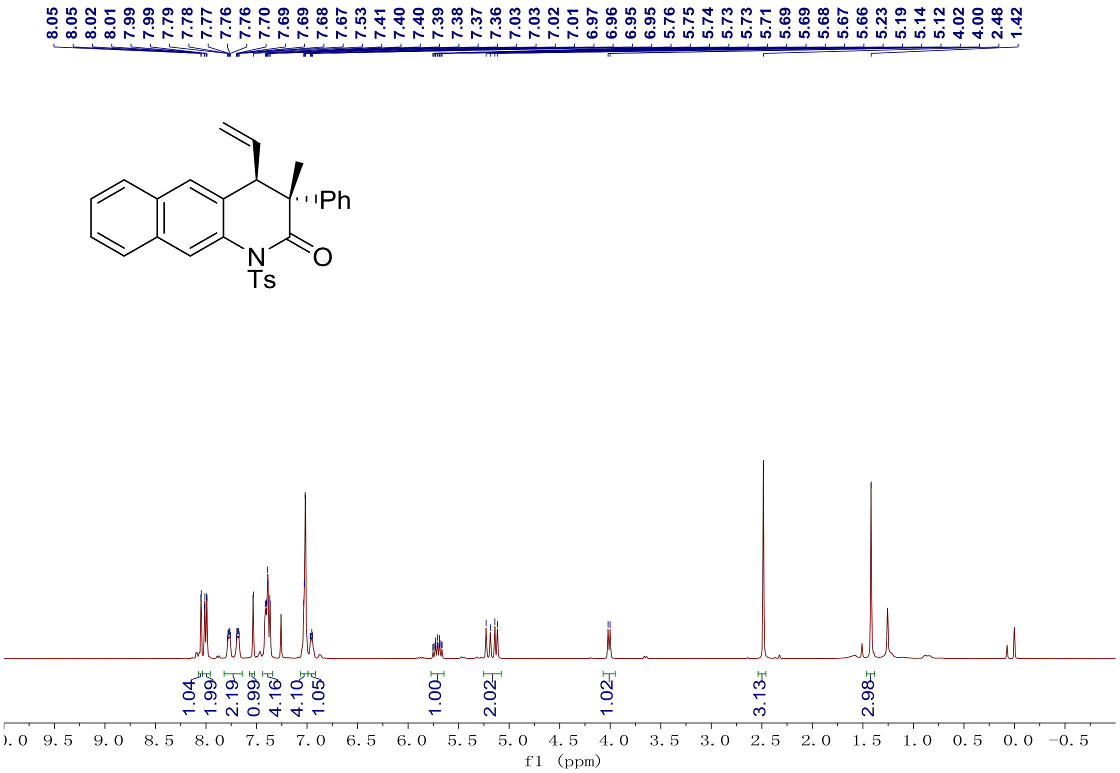

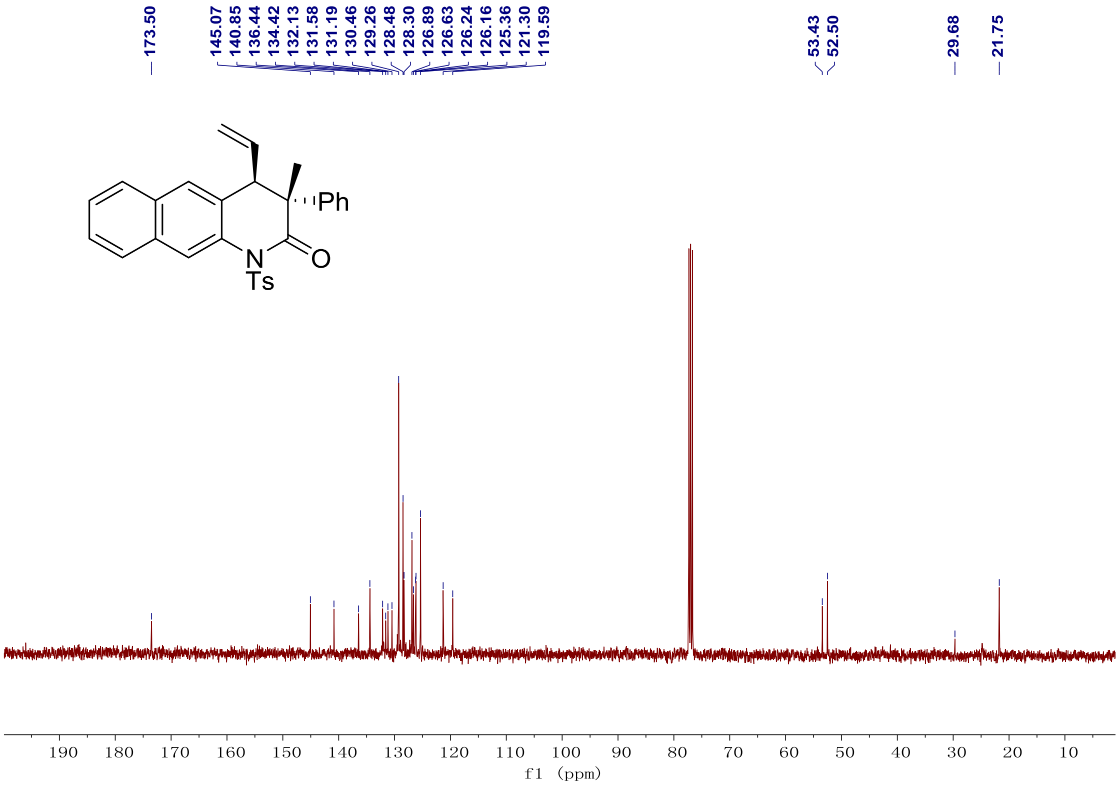

Supplementary Figure 17. ^{1}H and ^{13}C NMR of compound **3ha**

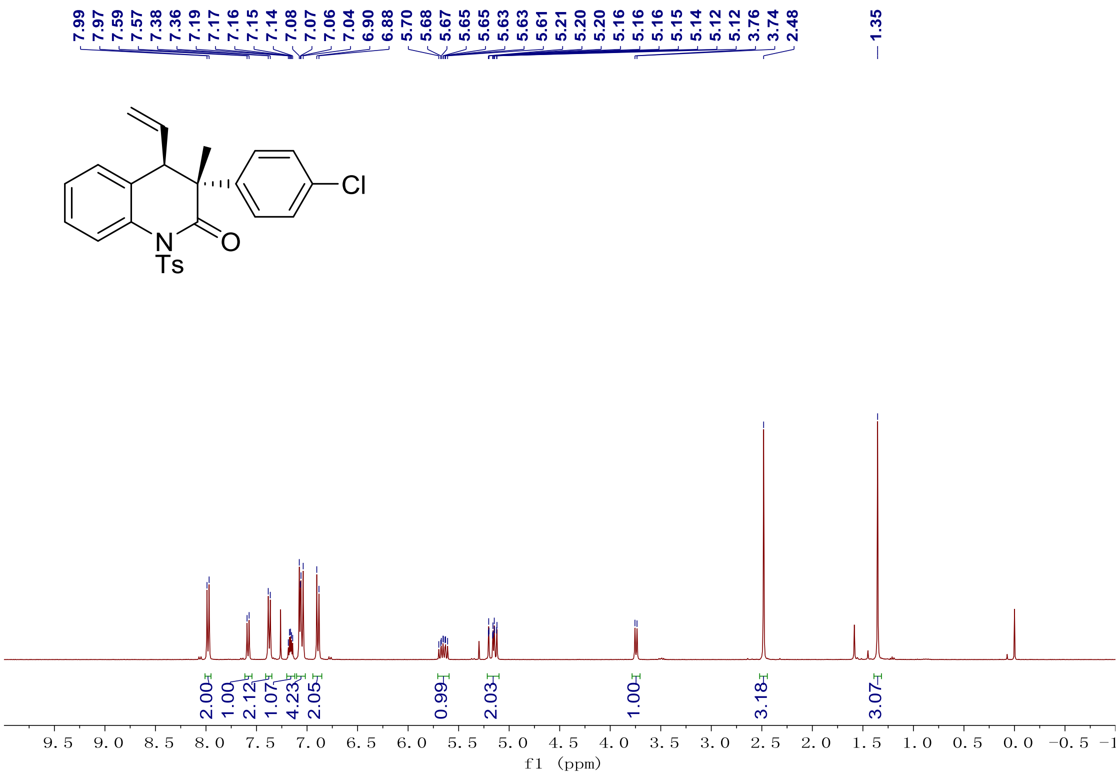

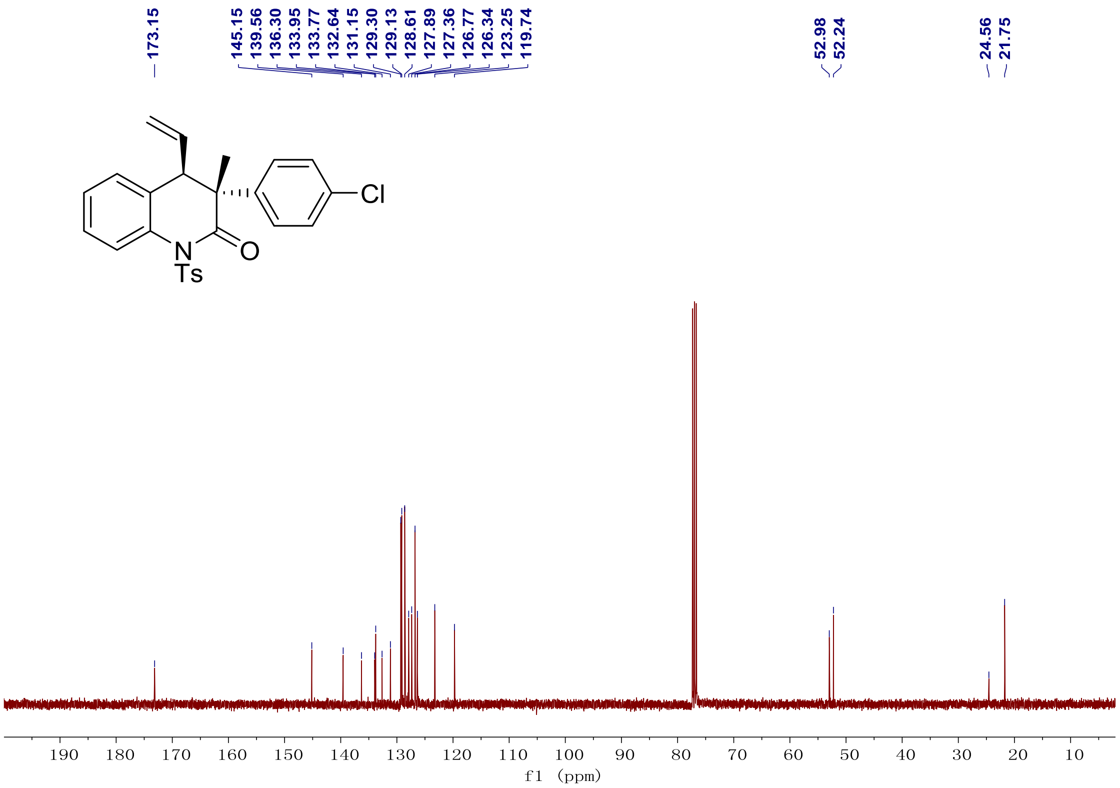

Supplementary Figure 18. ^{1}H and ^{13}C NMR of compound **3ab**

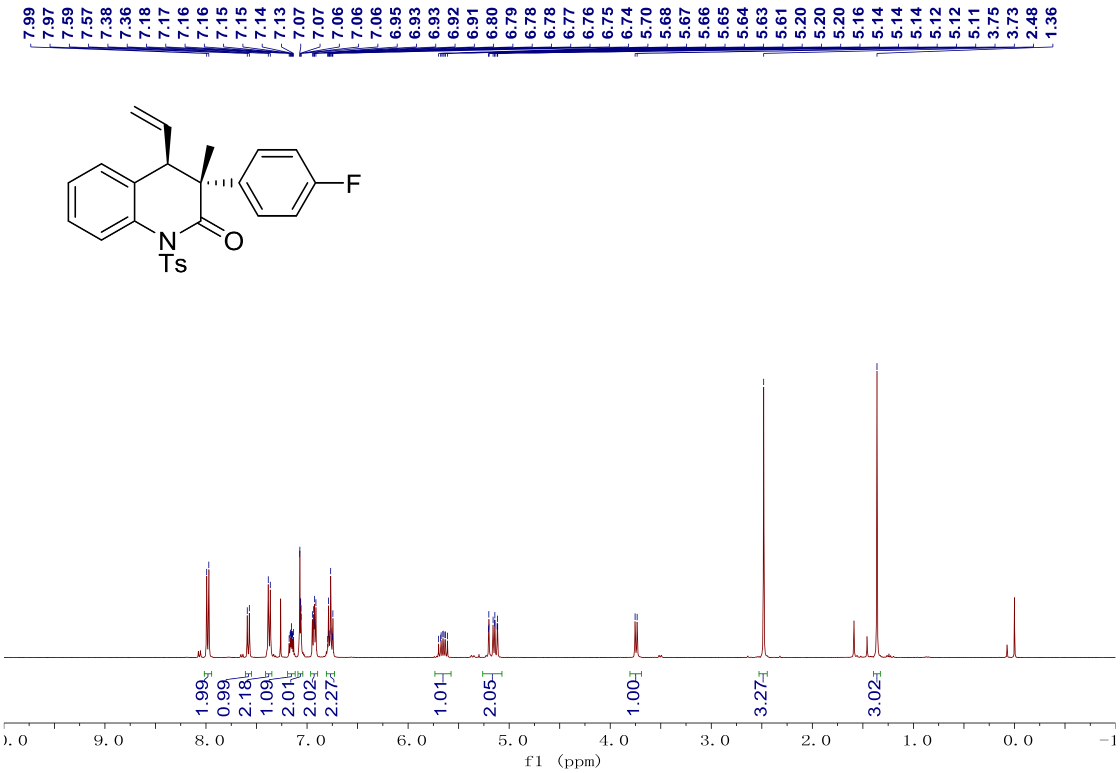

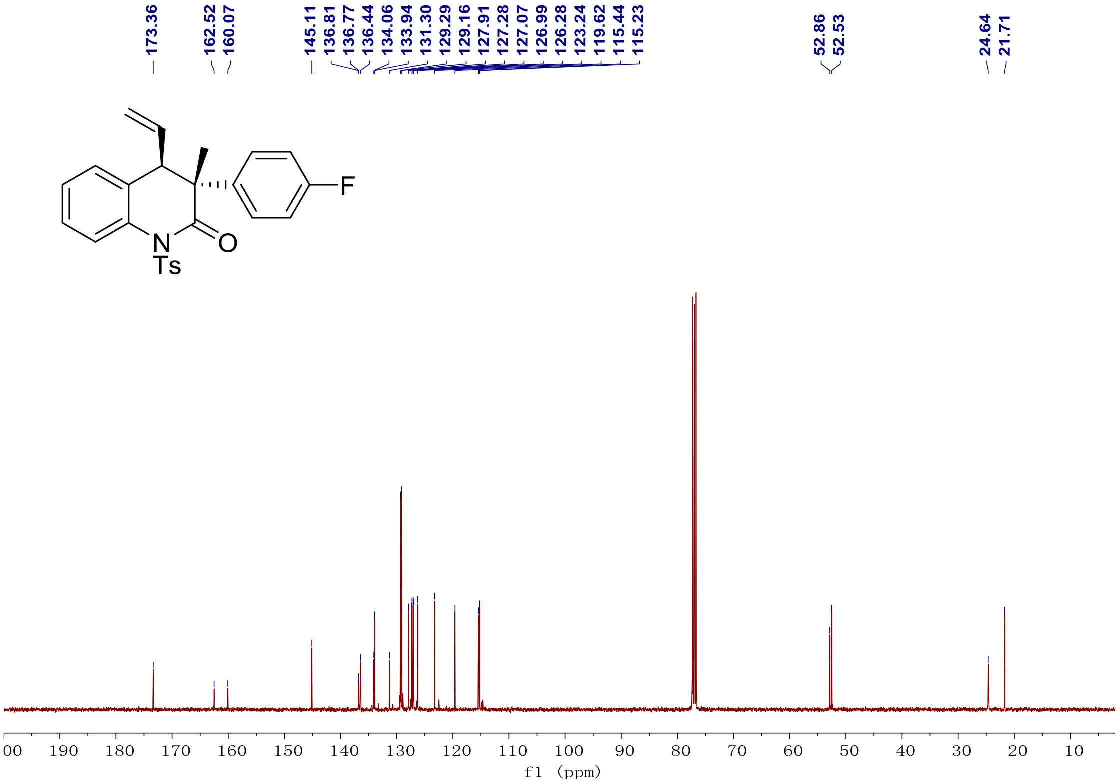

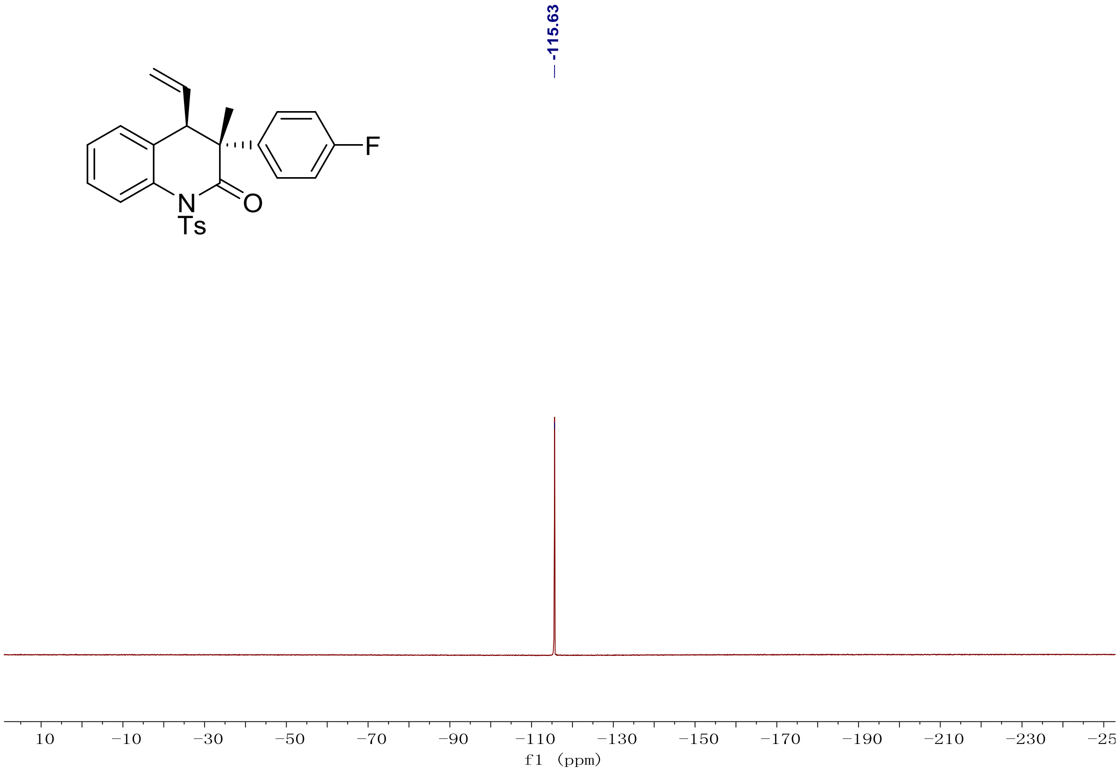

Supplementary Figure 19. ^{1}H and ^{13}C NMR ^{19}F NMRof compound **3ac**

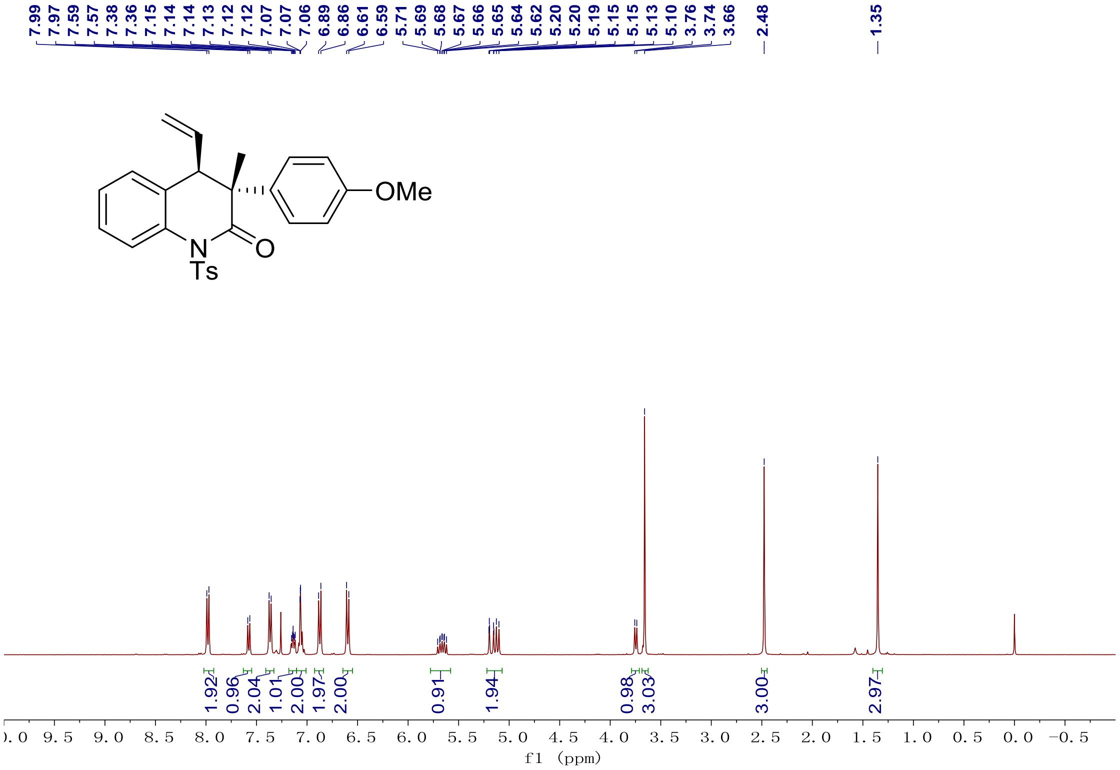

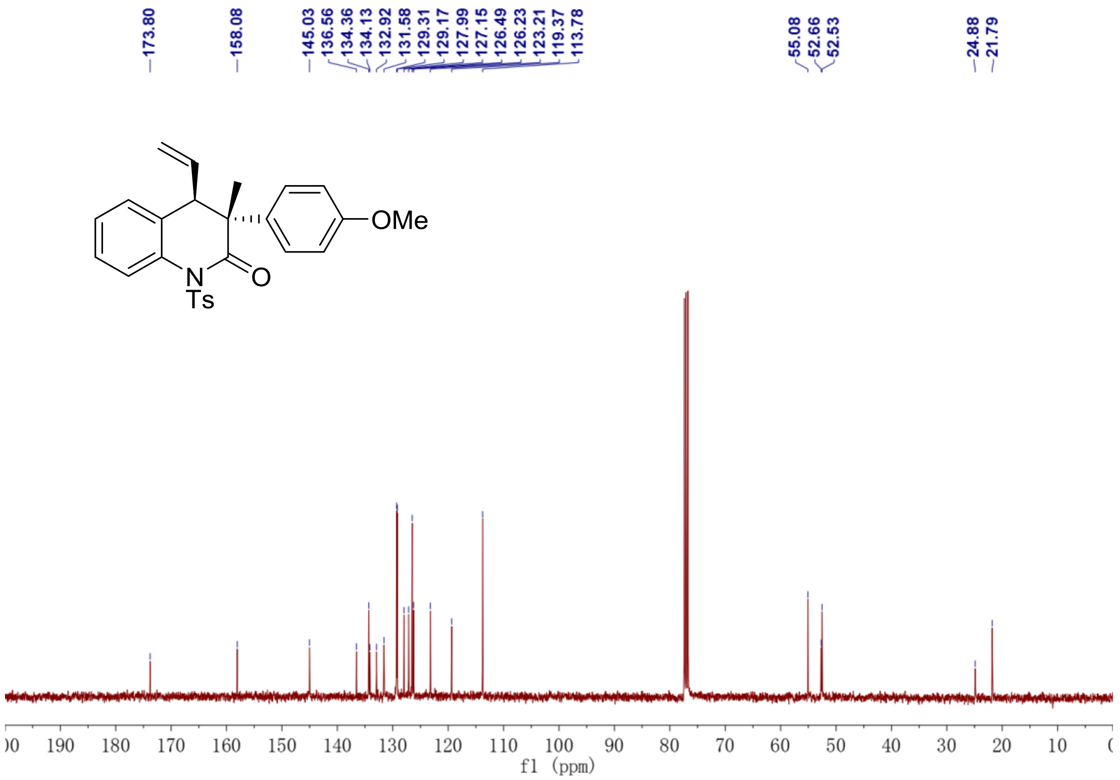

Supplementary Figure 20. ^{1}H and ^{13}C NMR of compound **3ad**

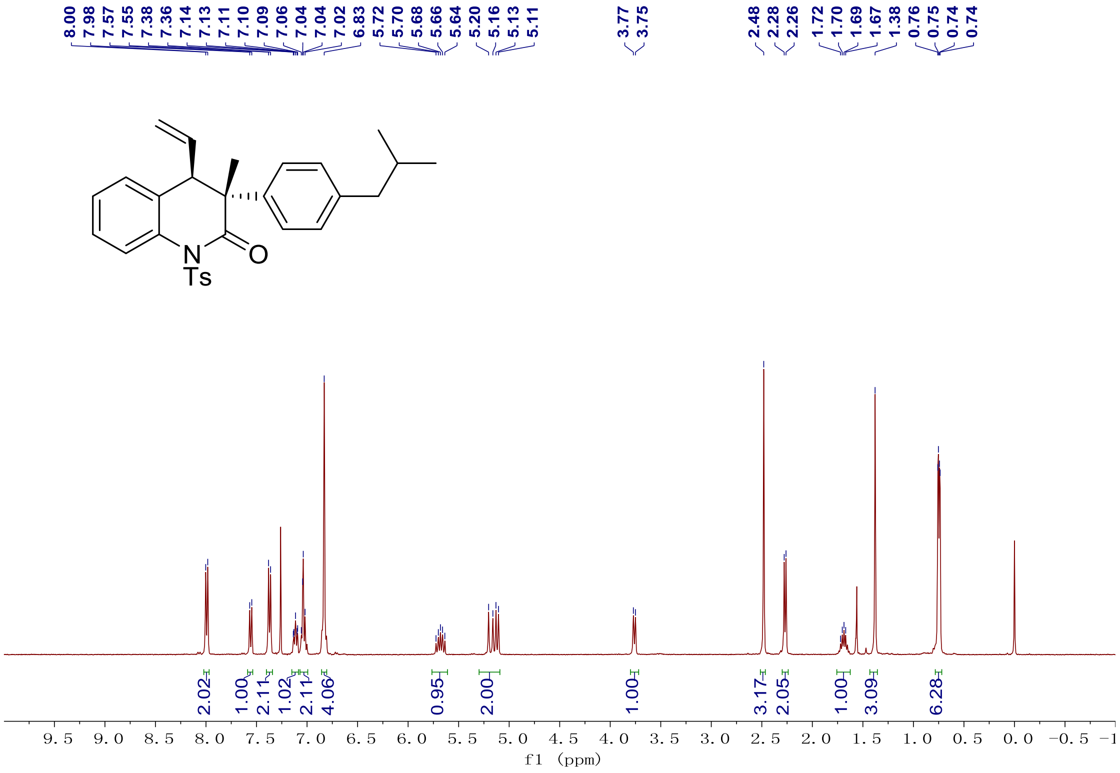

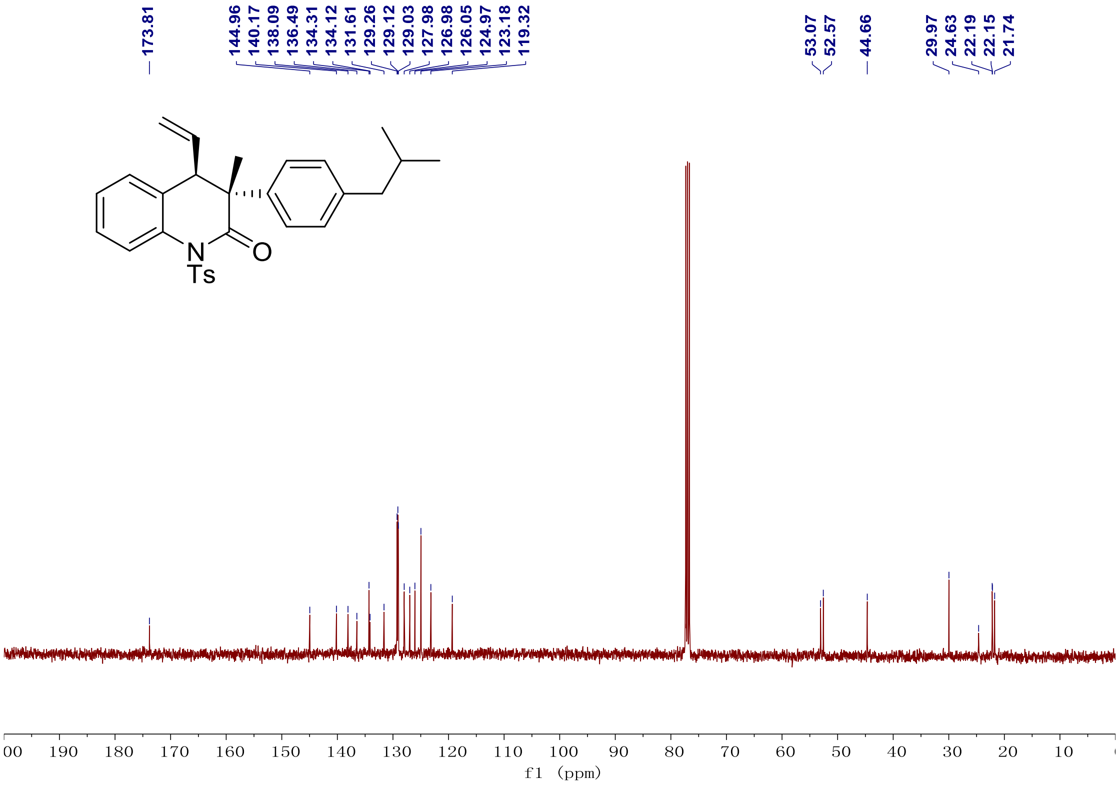

Supplementary Figure 21. ^{1}H and ^{13}C NMR of compound **3ae**

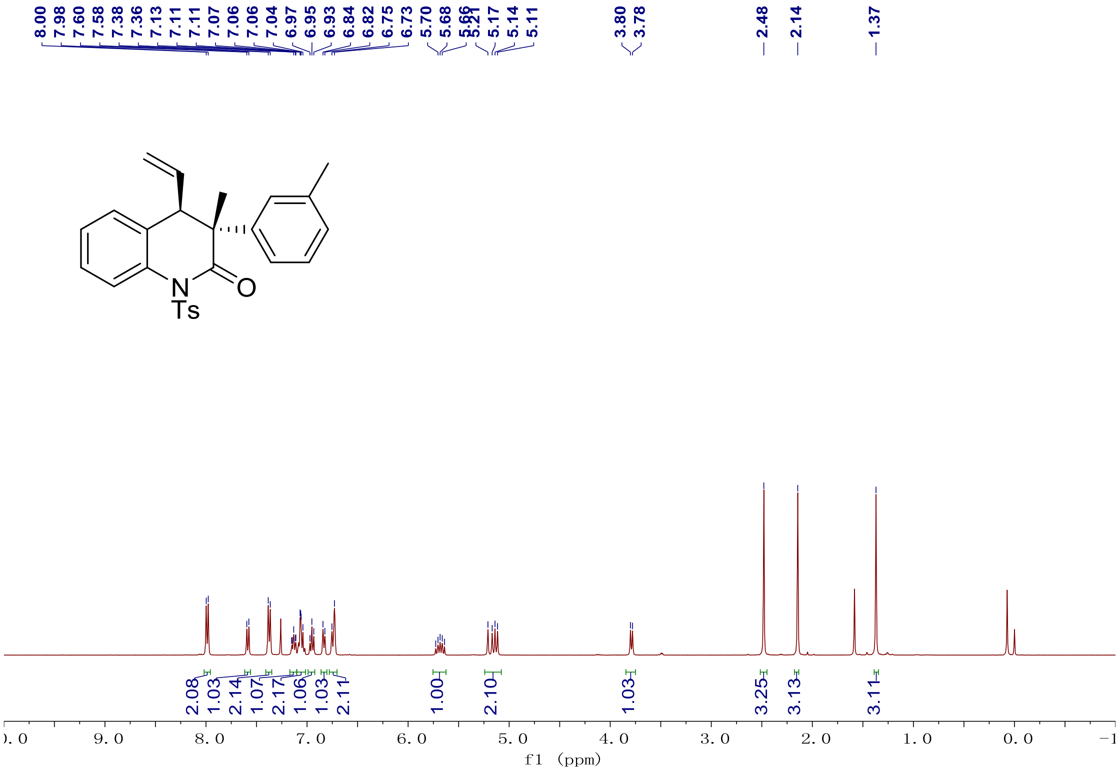

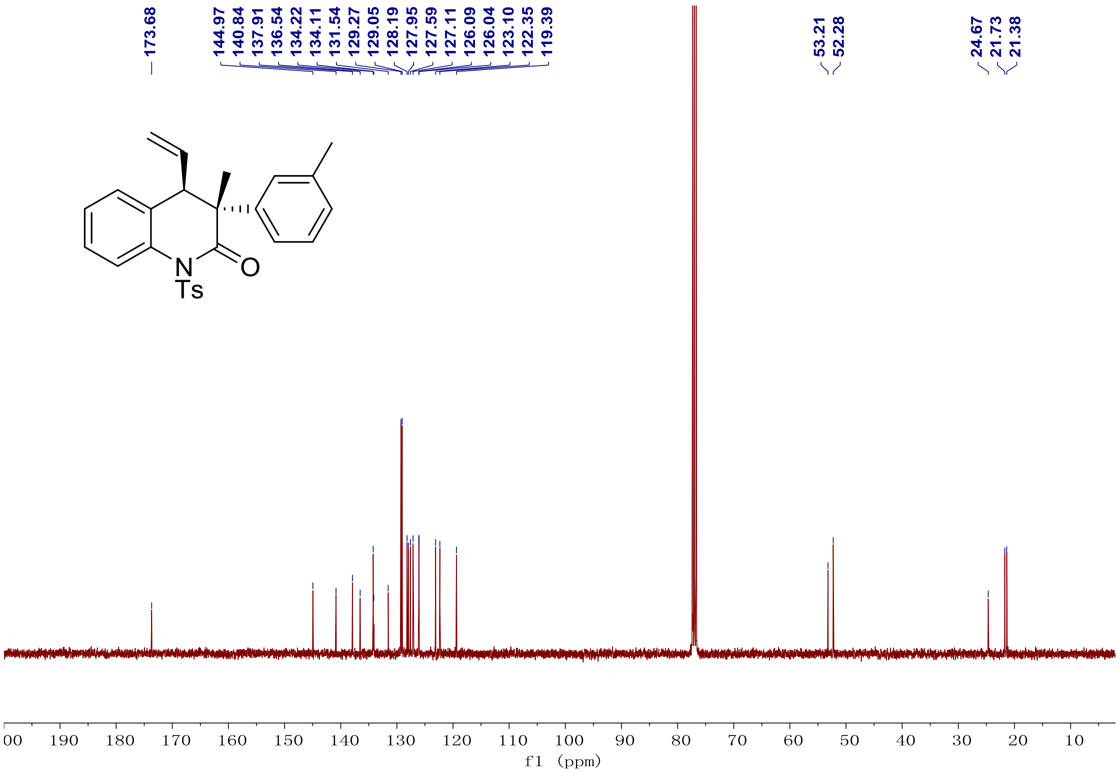

Supplementary Figure 22. ^{1}H and ^{13}C NMR of compound **3af**

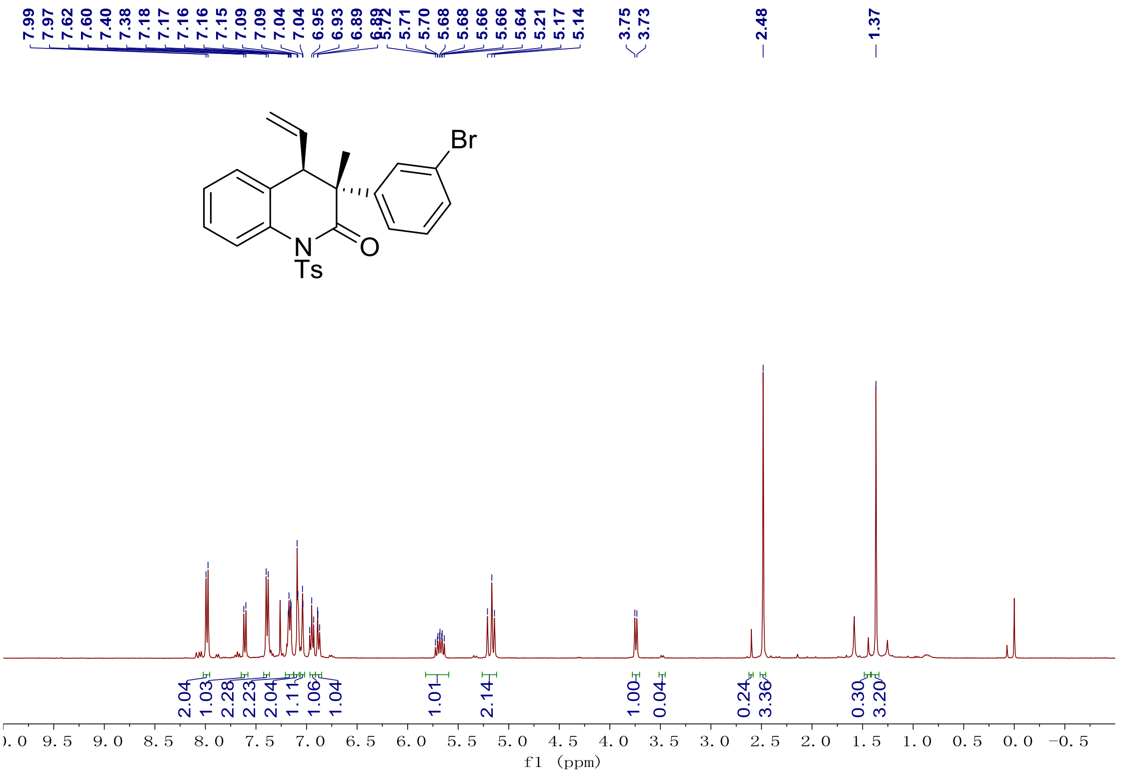

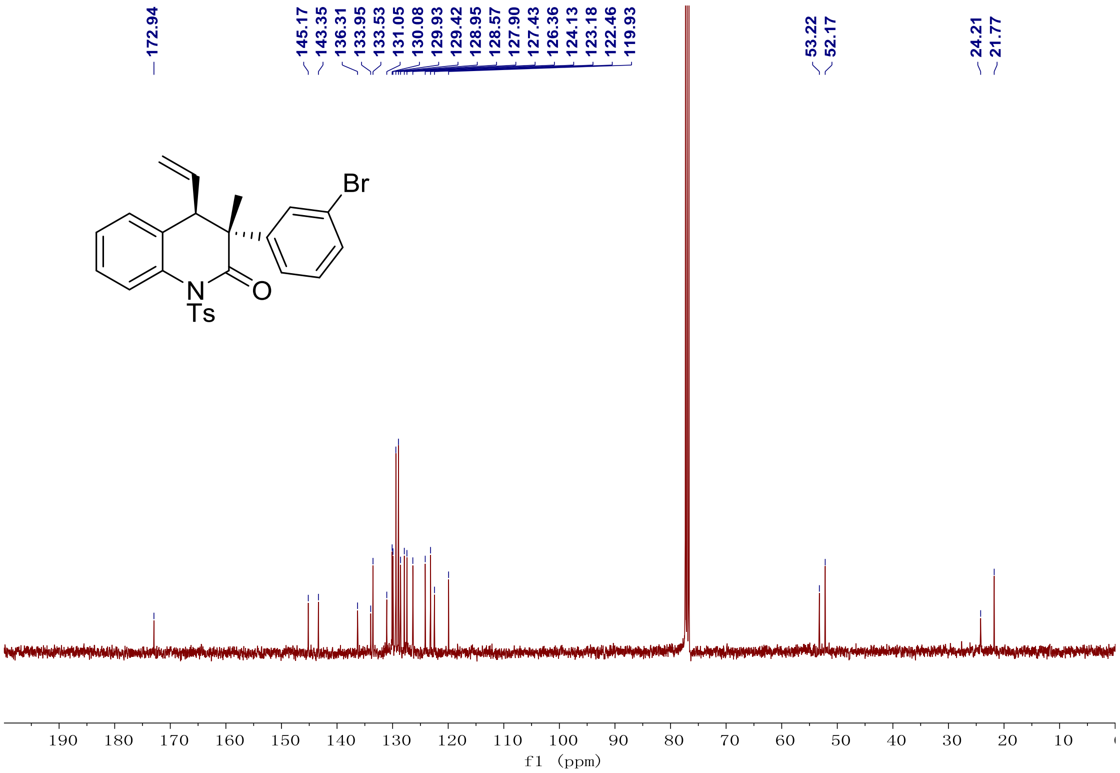

Supplementary Figure 23. ^{1}H and ^{13}C NMR of compound **3ag**

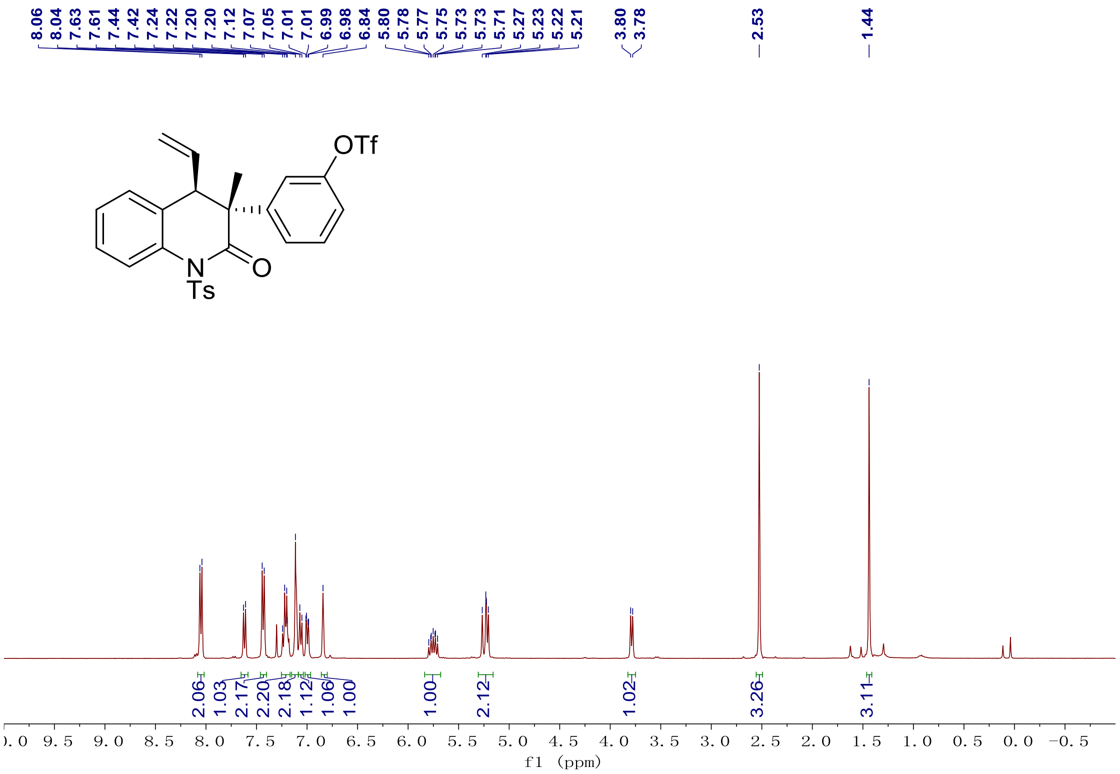

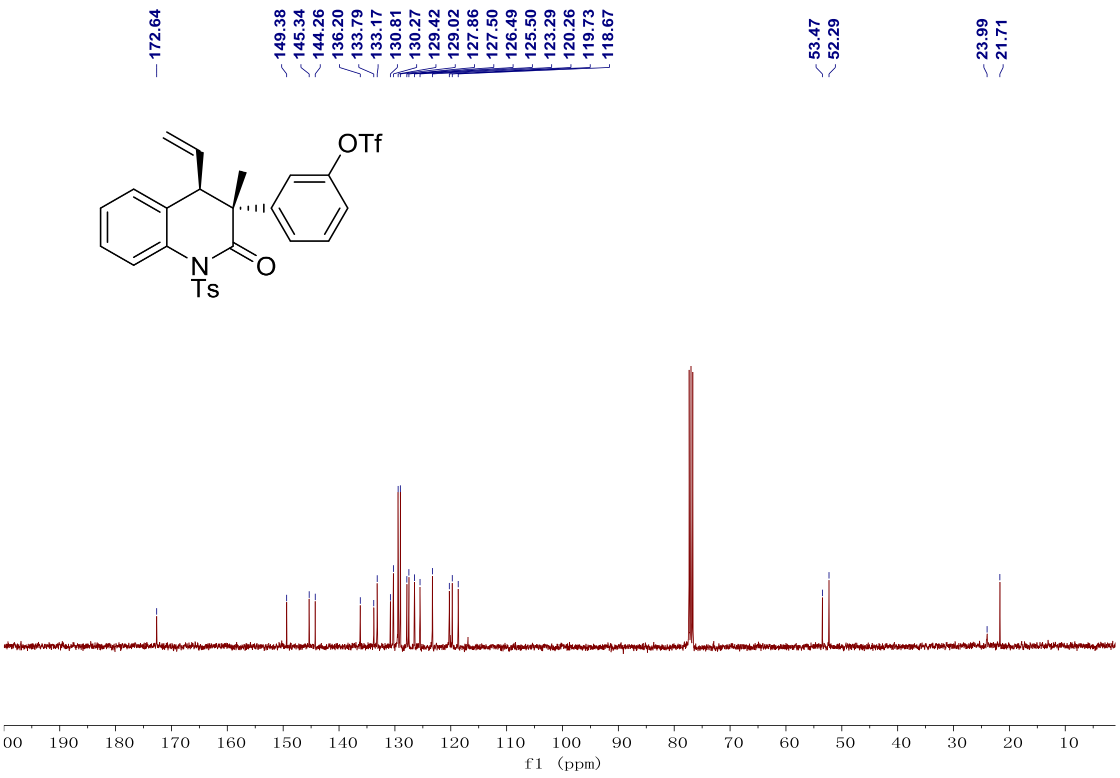

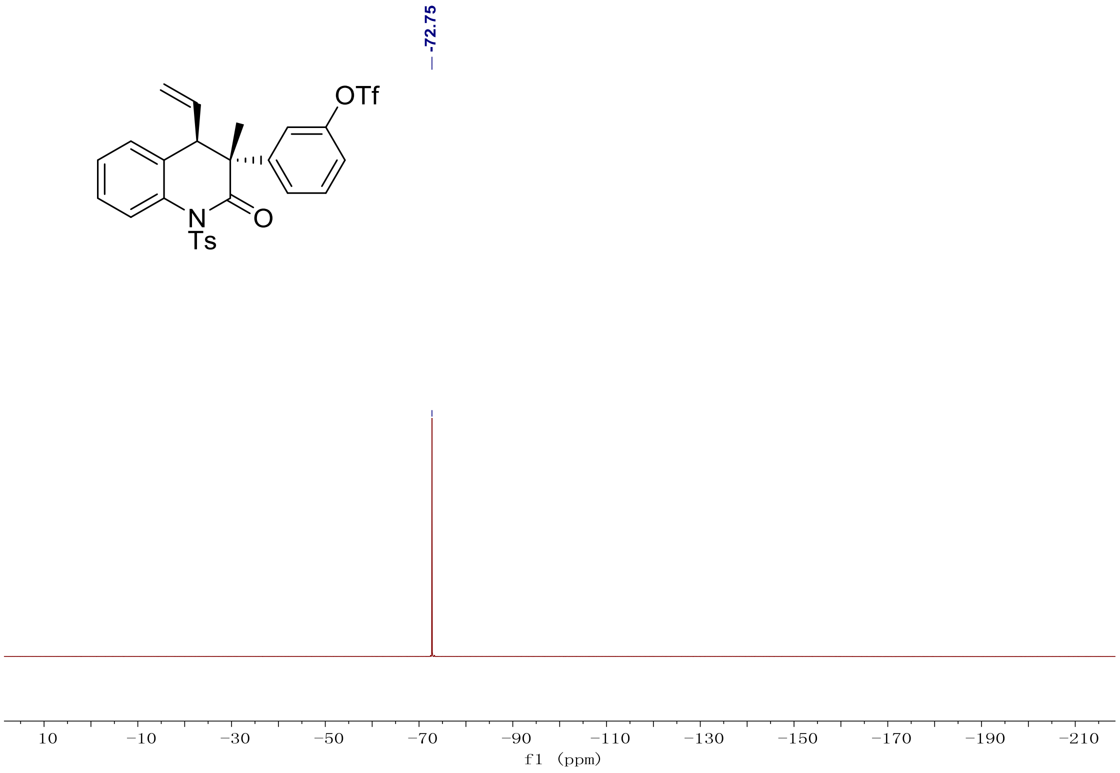

Supplementary Figure 24. ^{1}H and ^{13}C NMR ^{19}F NMR of compound **3ah**

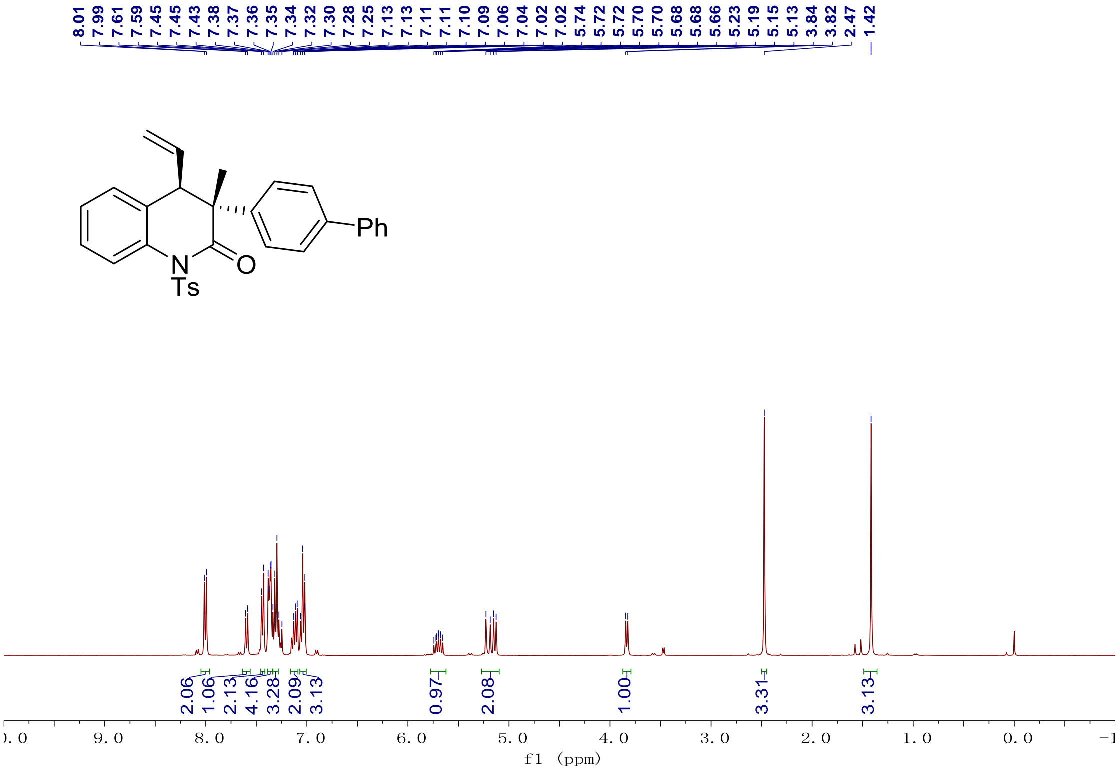

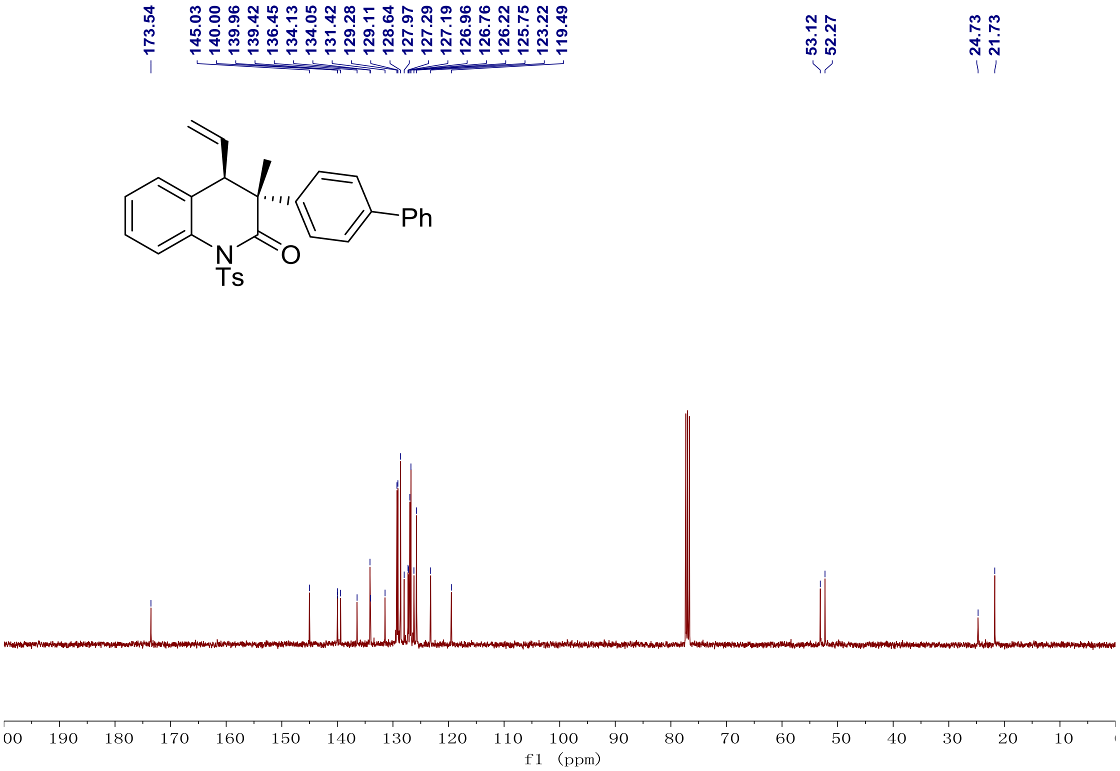

Supplementary Figure 25. ^{1}H and ^{13}C NMR of compound **3ai**

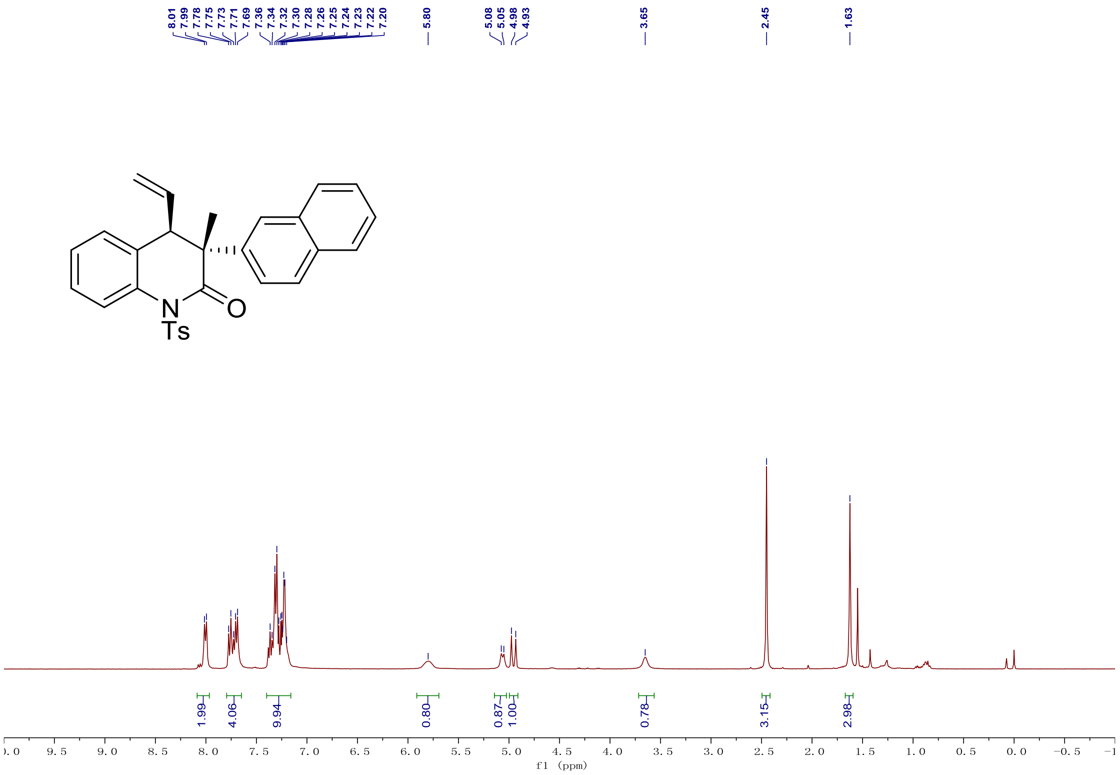

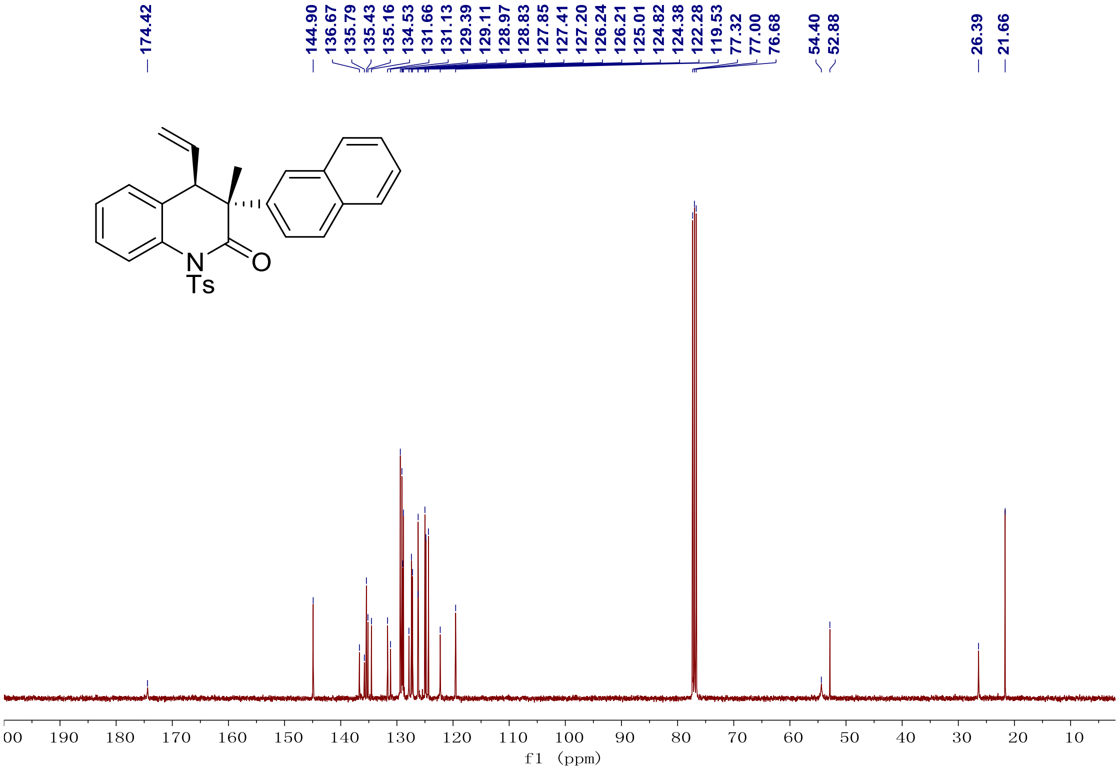

Supplementary Figure 26. ^{1}H and ^{13}C NMR of compound **3aj**

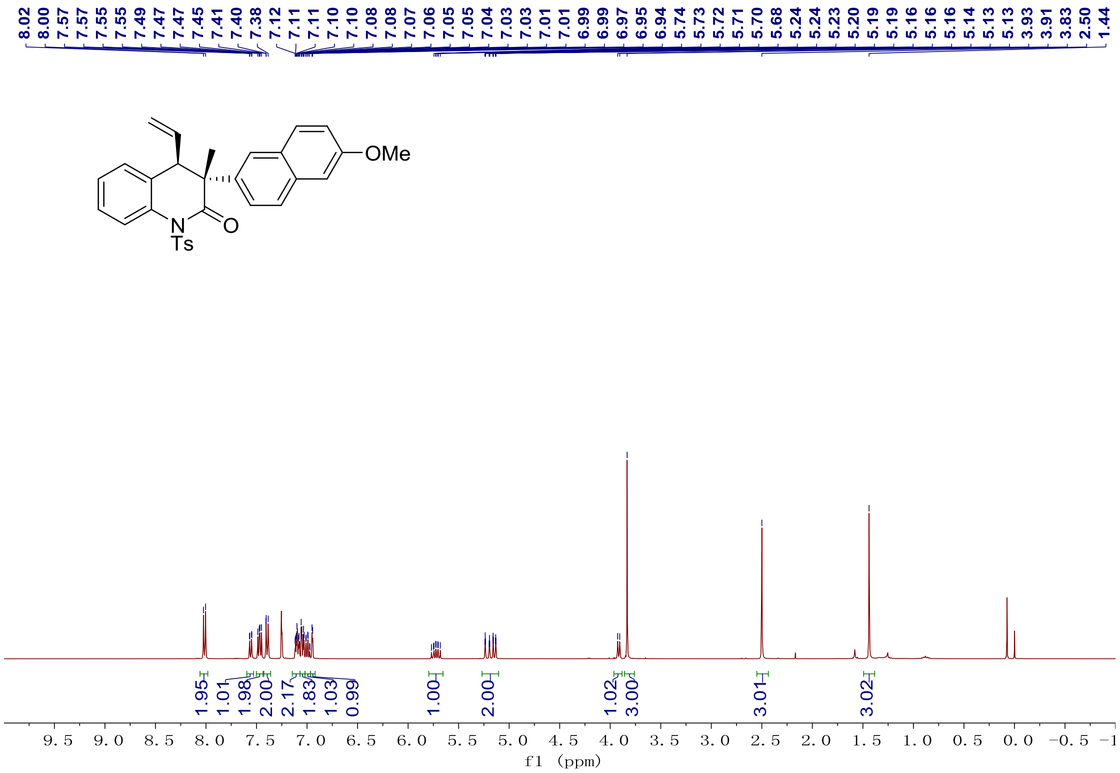

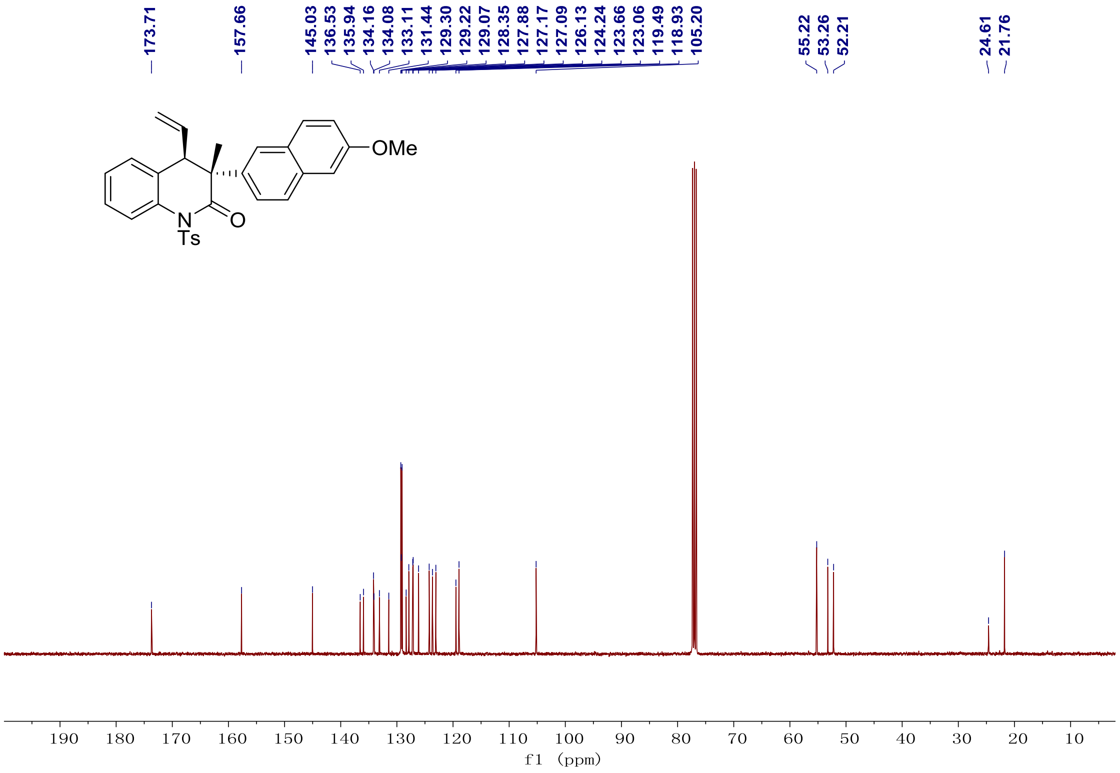

Supplementary Figure 27. ^{1}H and ^{13}C NMR of compound **3ak**

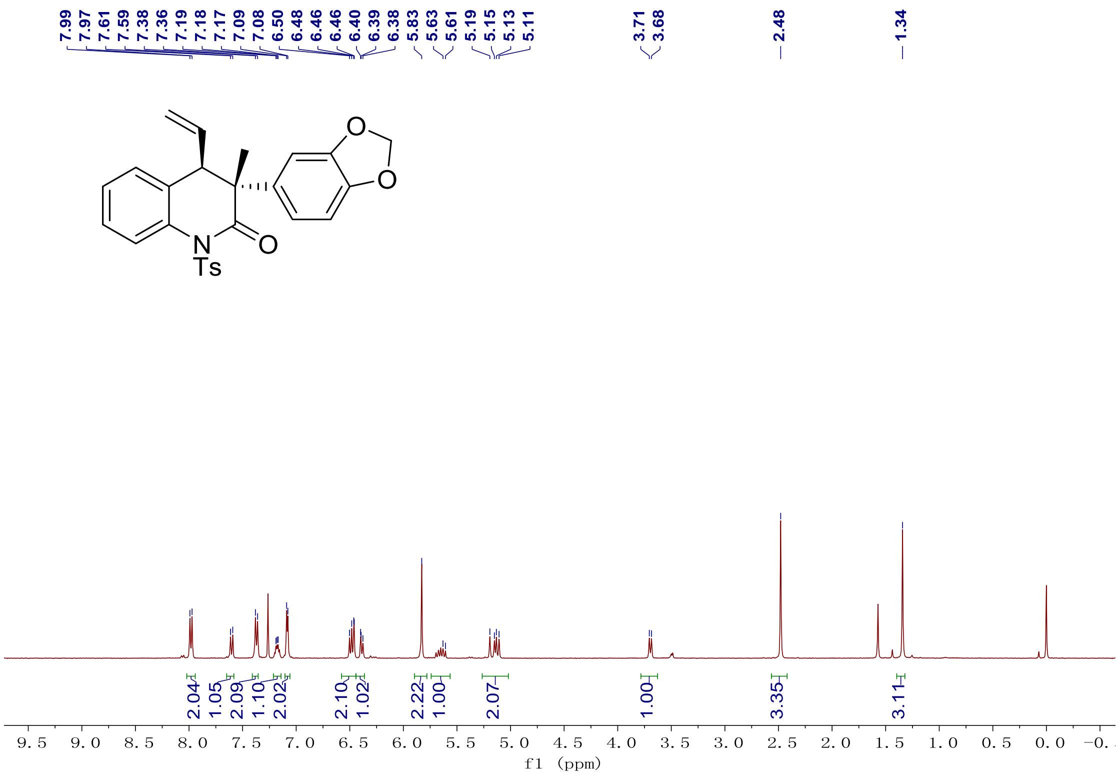

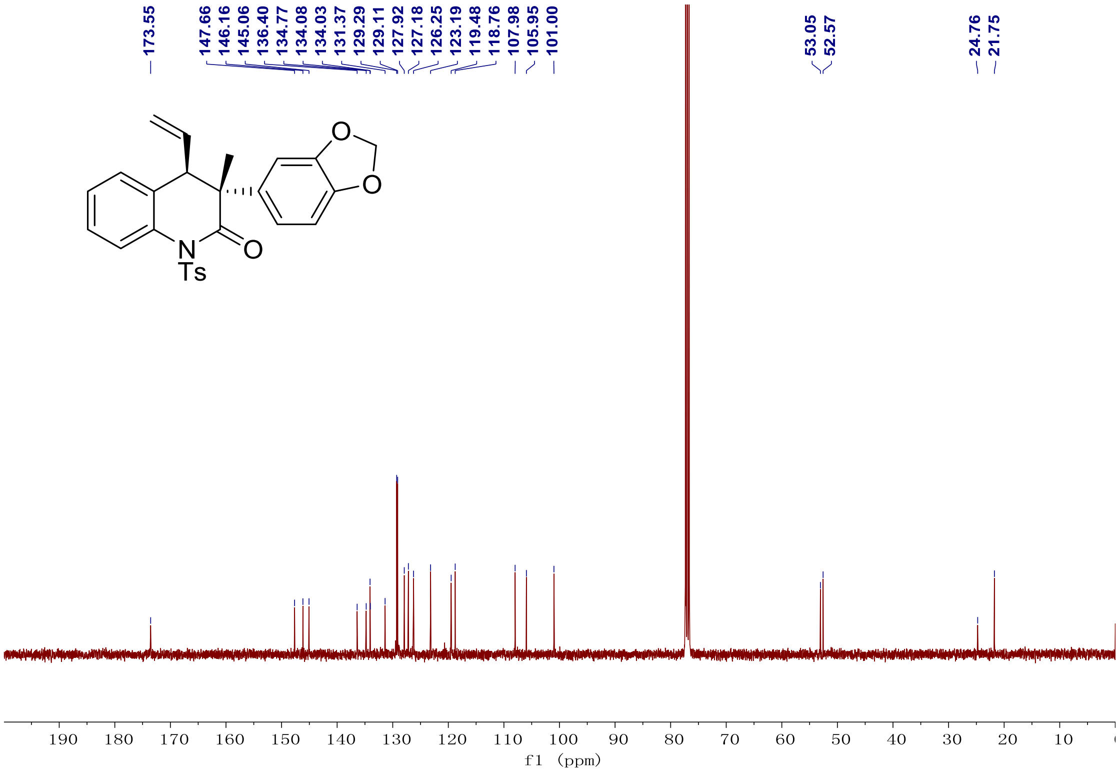

Supplementary Figure 28. ^{1}H and ^{13}C NMR of compound **3al**

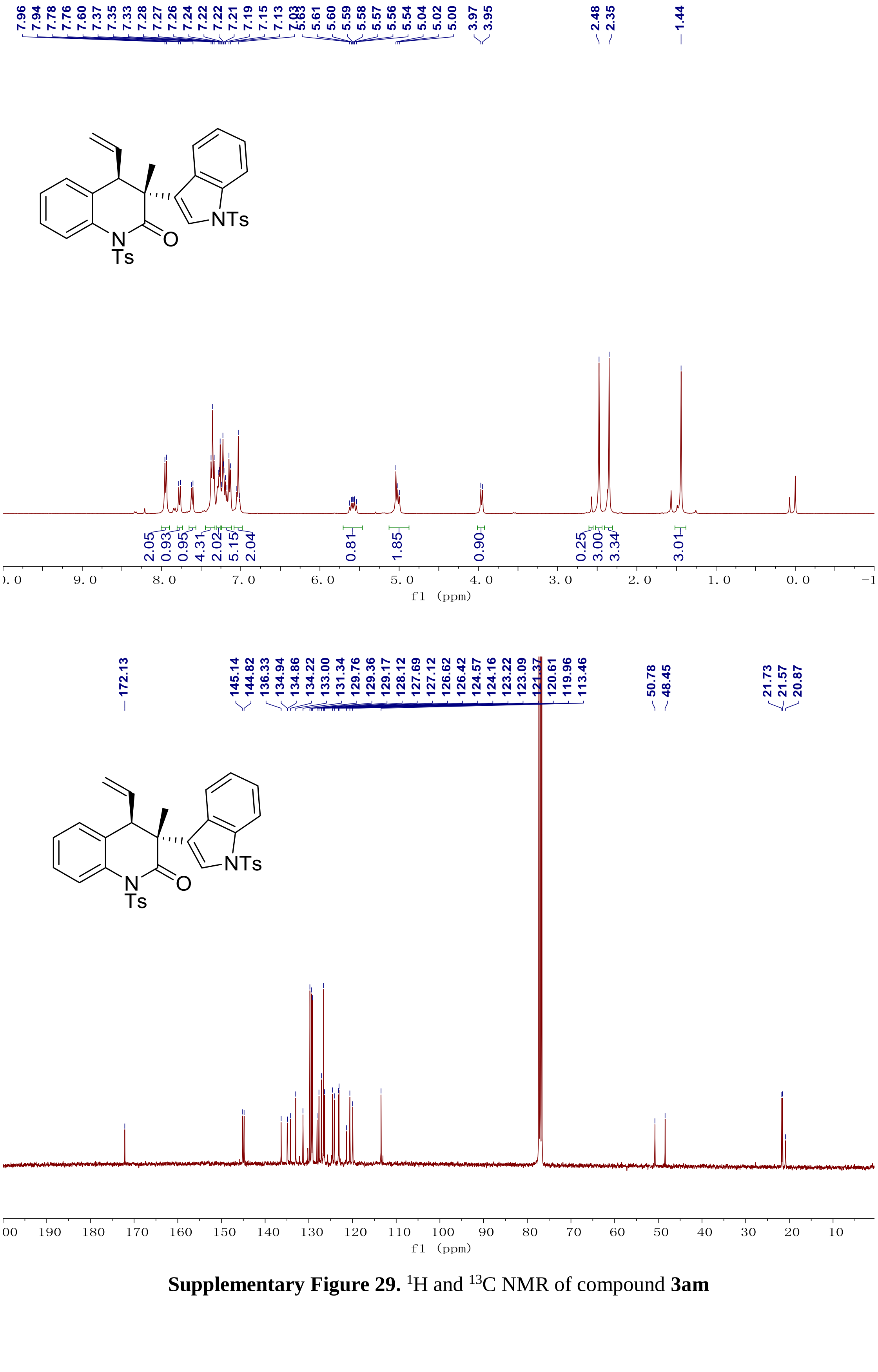

Supplementary Figure 29. ^{1}H and ^{13}C NMR of compound **3am**

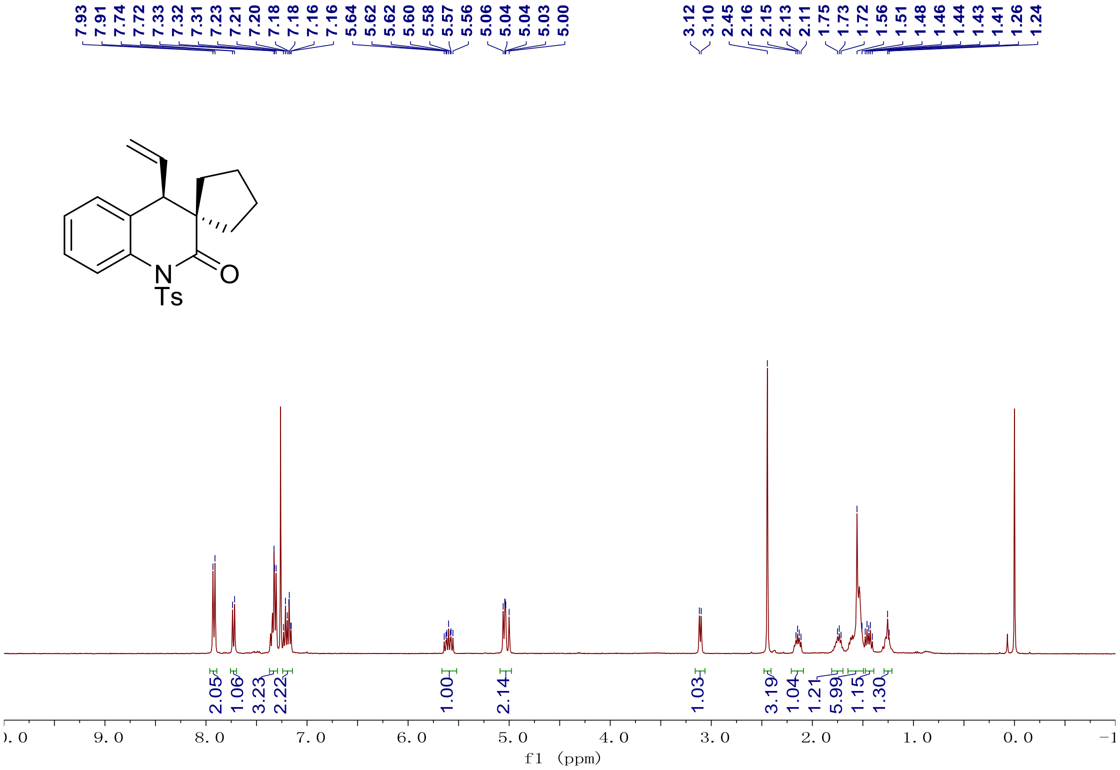

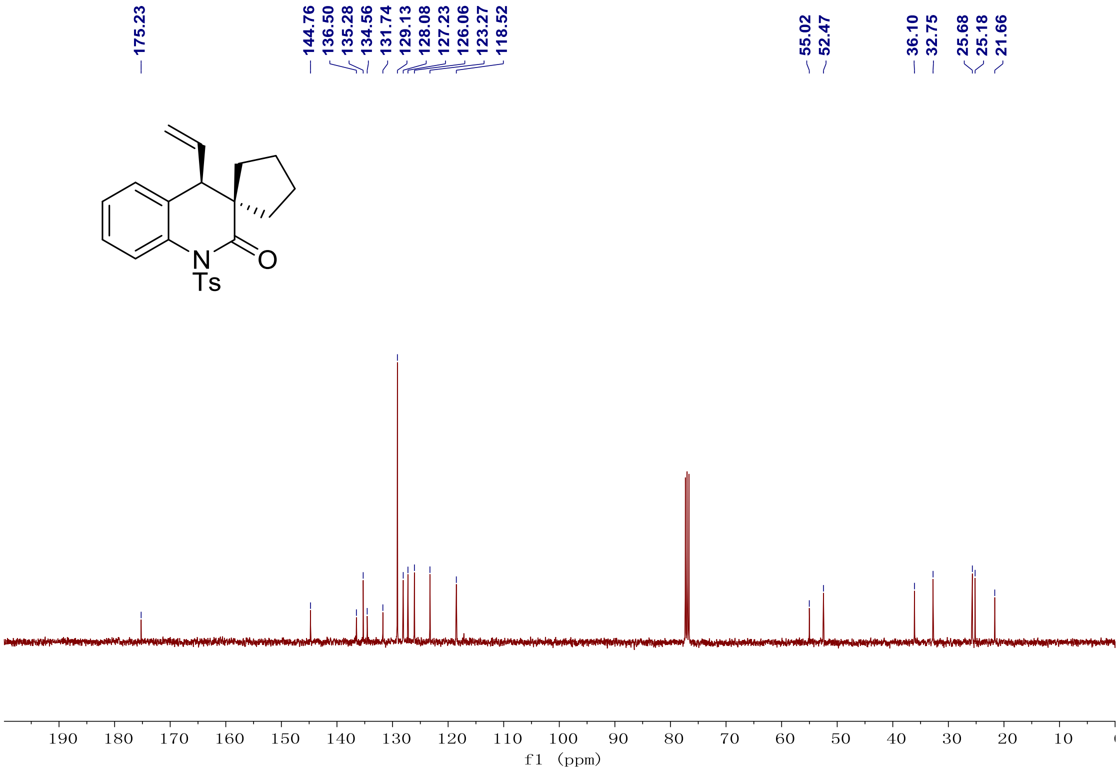

Supplementary Figure30. ^{1}H and ^{13}C NMR ^{19}F NMR of compound **3an**

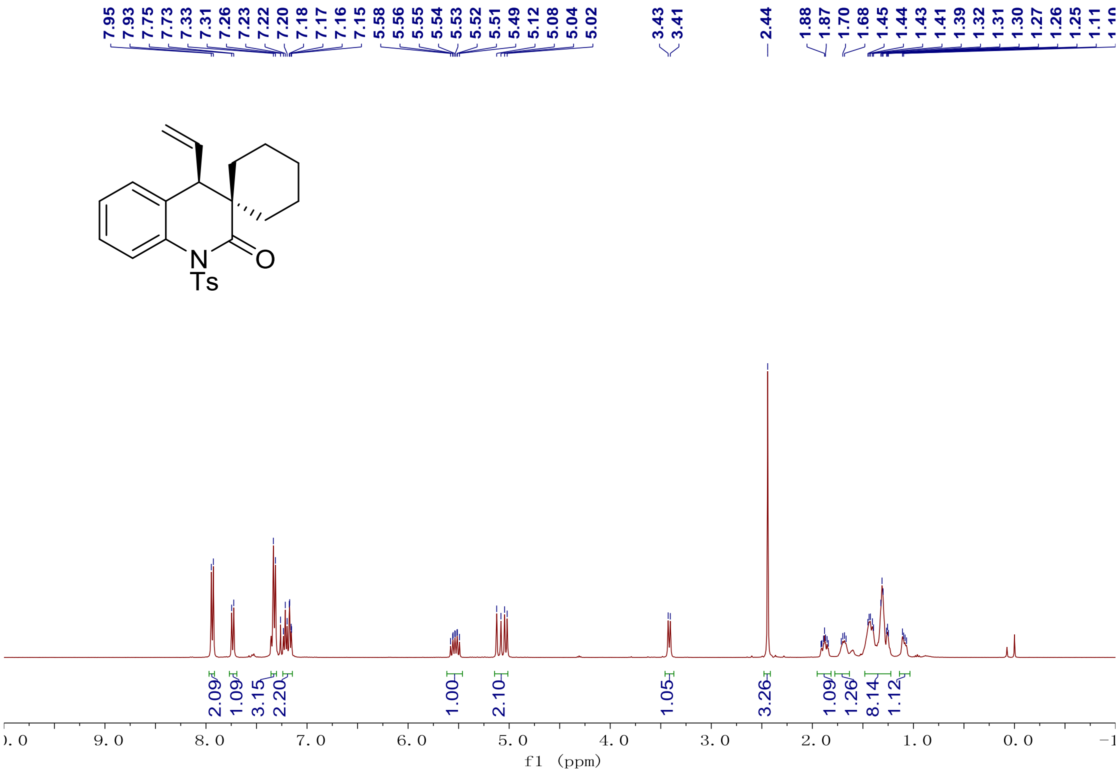

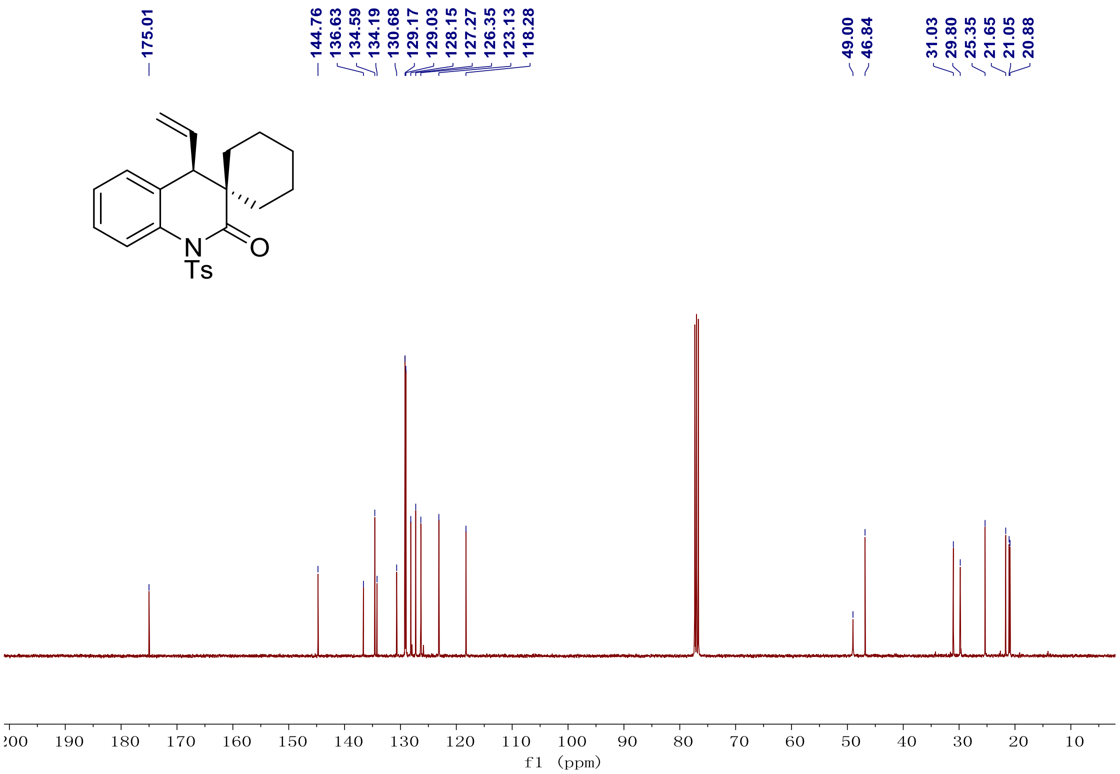

Supplementary Figure 31. ^{1}H and ^{13}C NMR of compound **3ao**

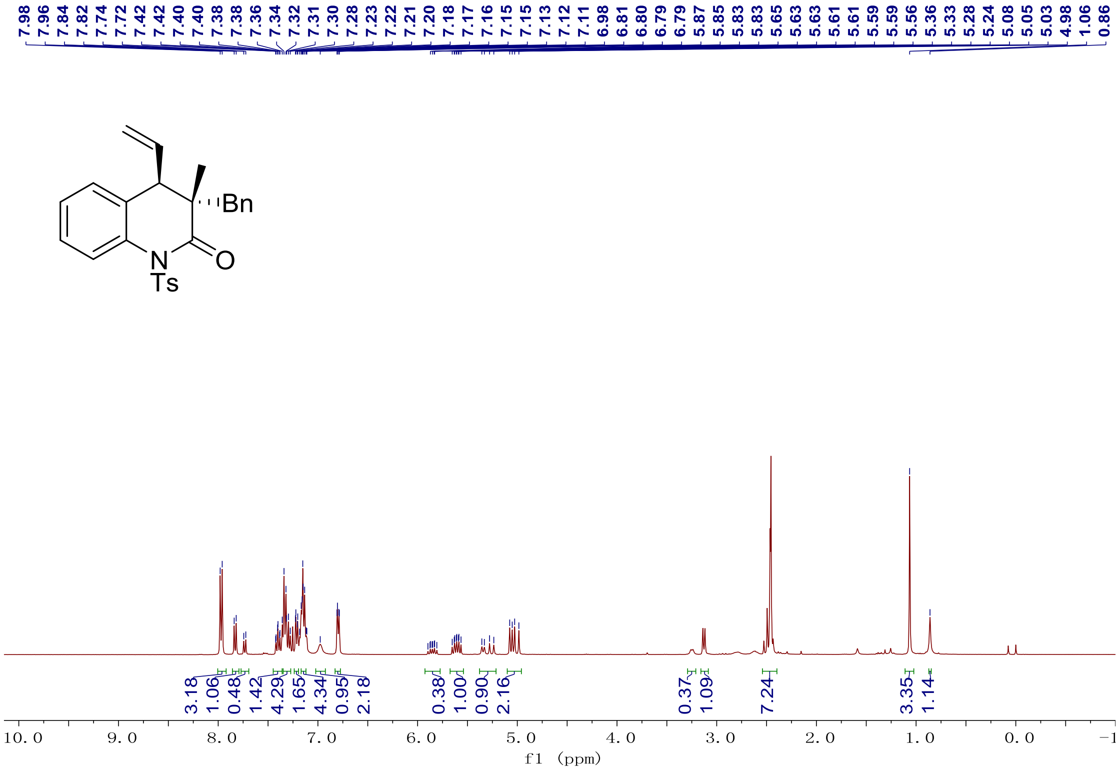

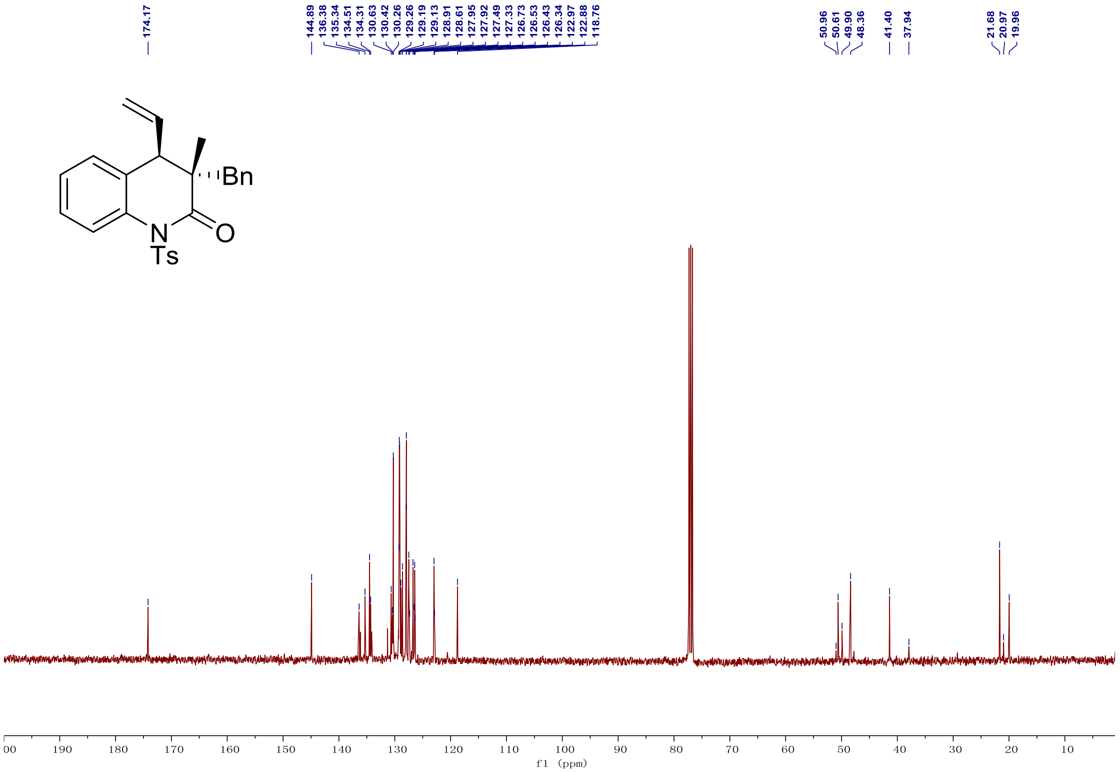

Supplementary Figure 32. ^{1}H and ^{13}C NMR of compound **3ap**

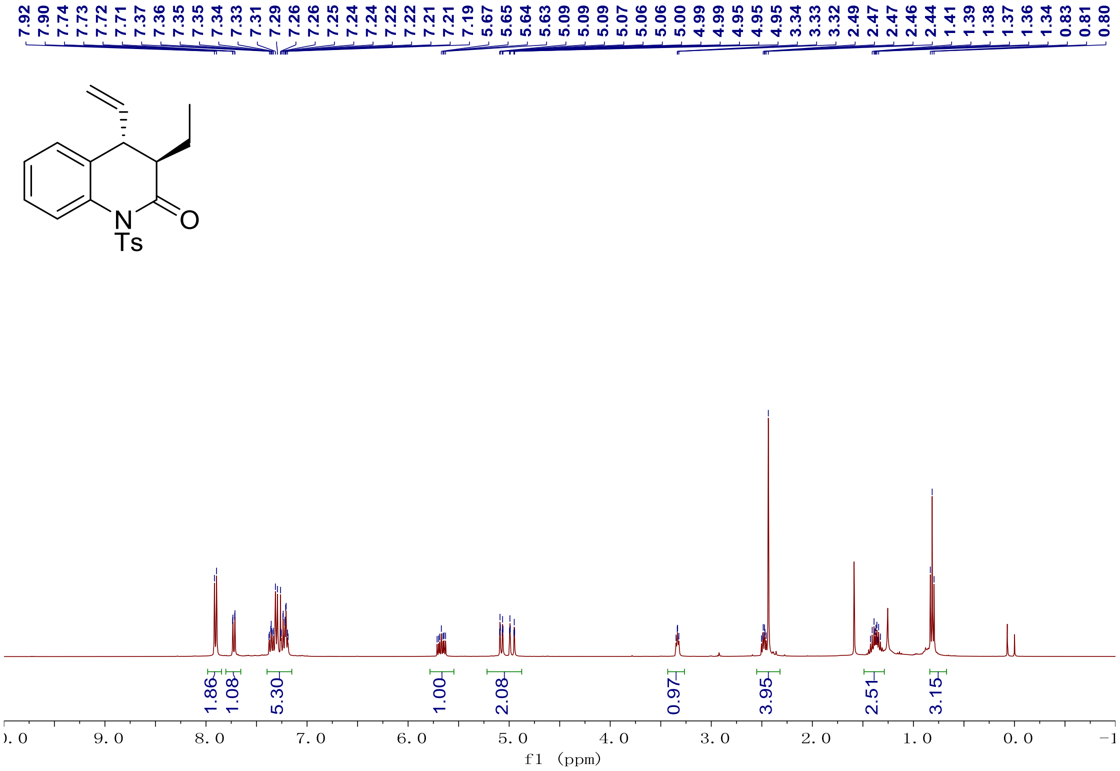

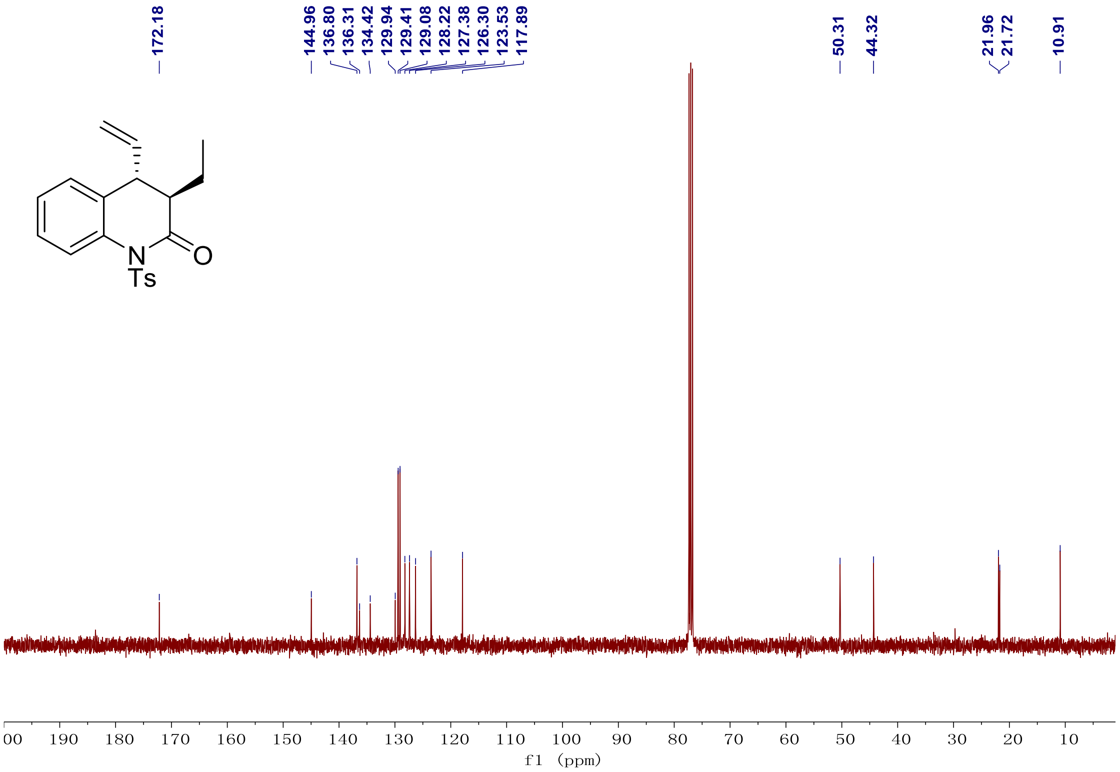

Supplementary Figure 33. ^{1}H and ^{13}C NMR of compound **3ar**

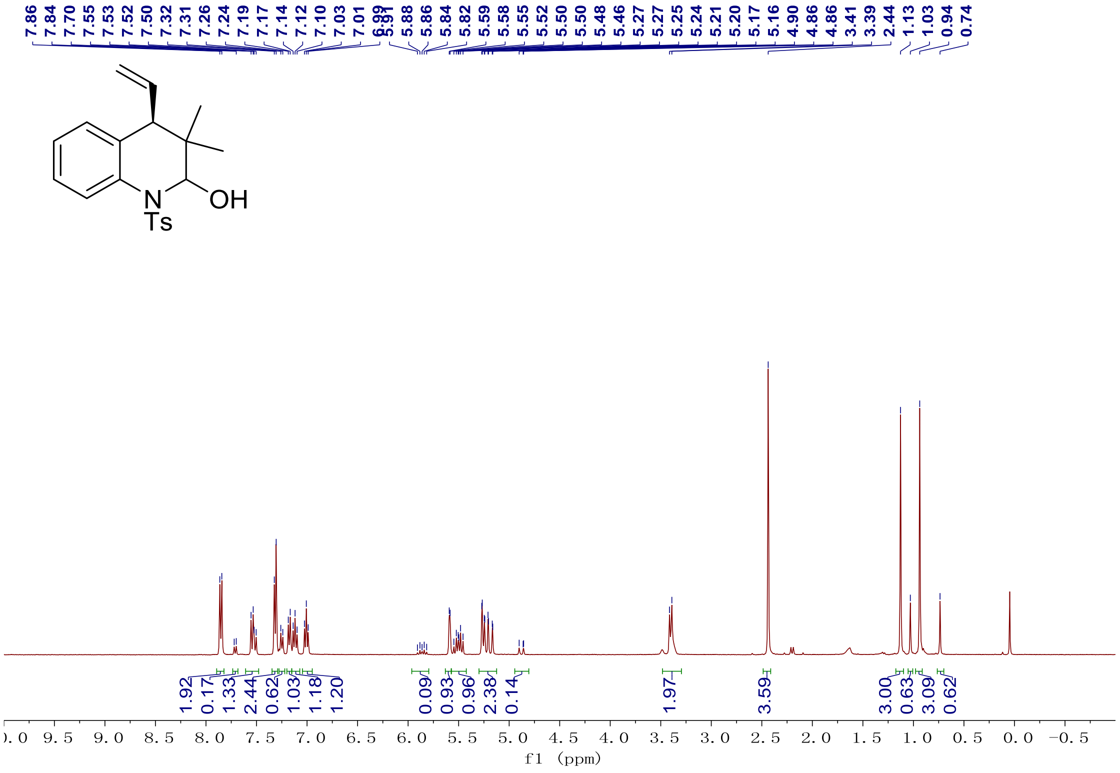

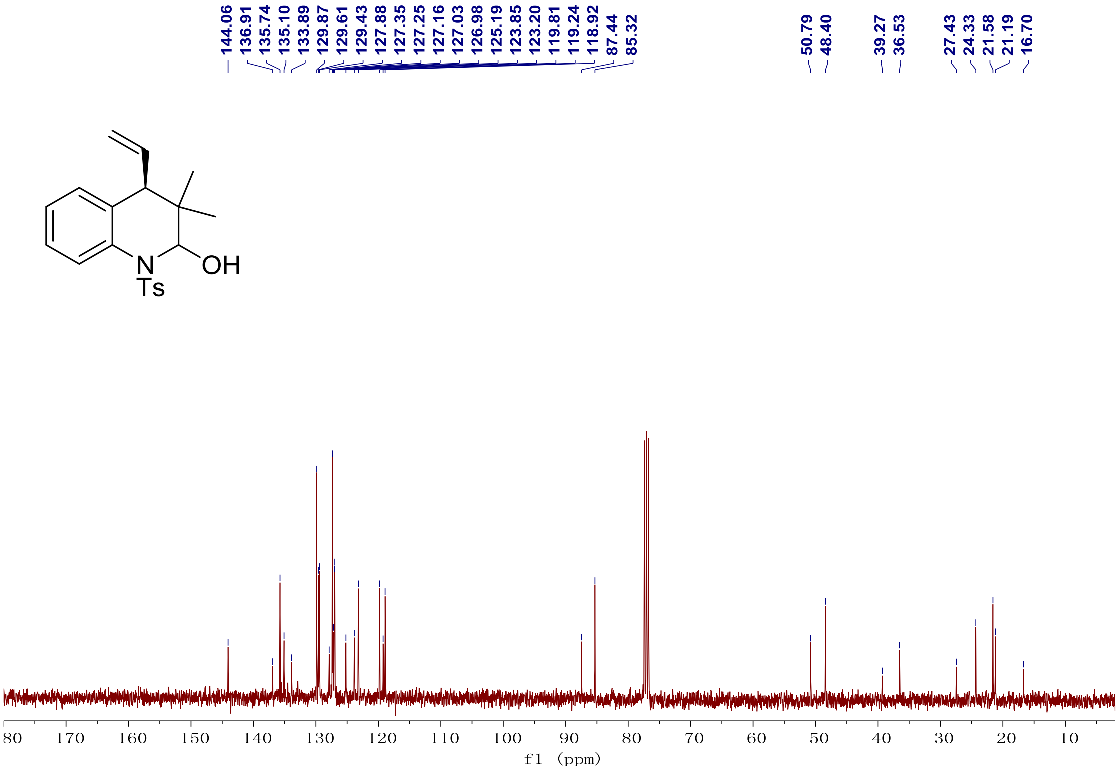

Supplementary Figure 34. ^{1}H and ^{13}C NMR of compound **4aq**

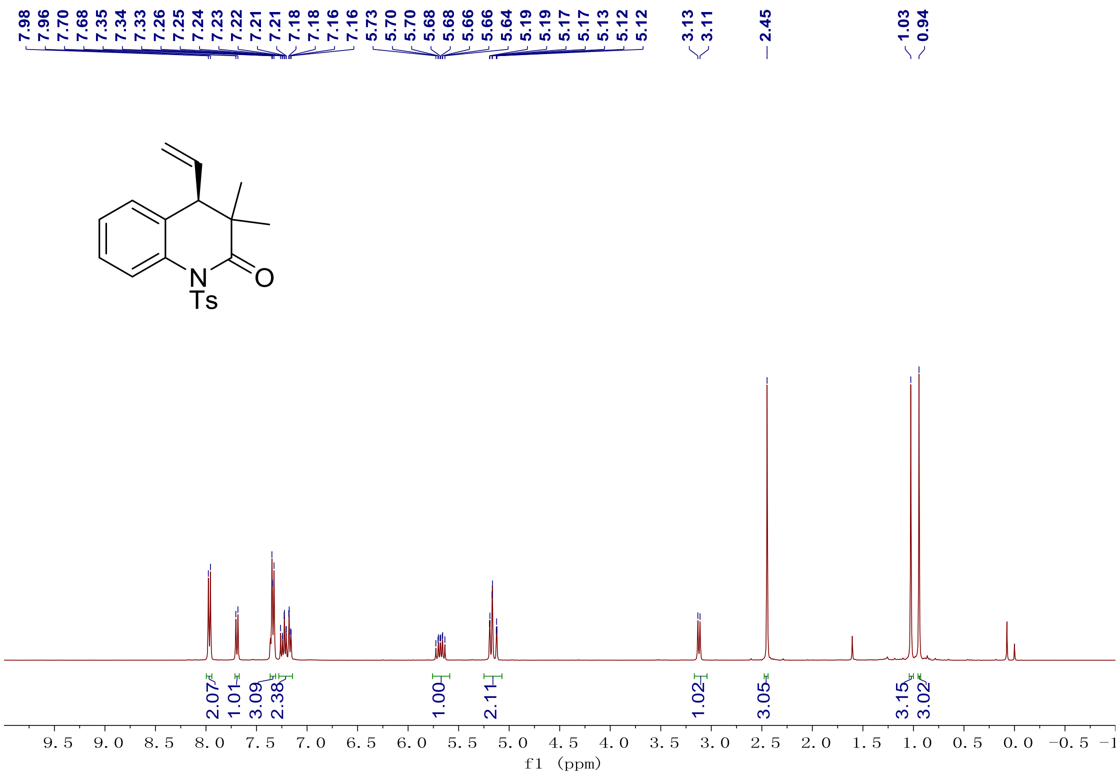

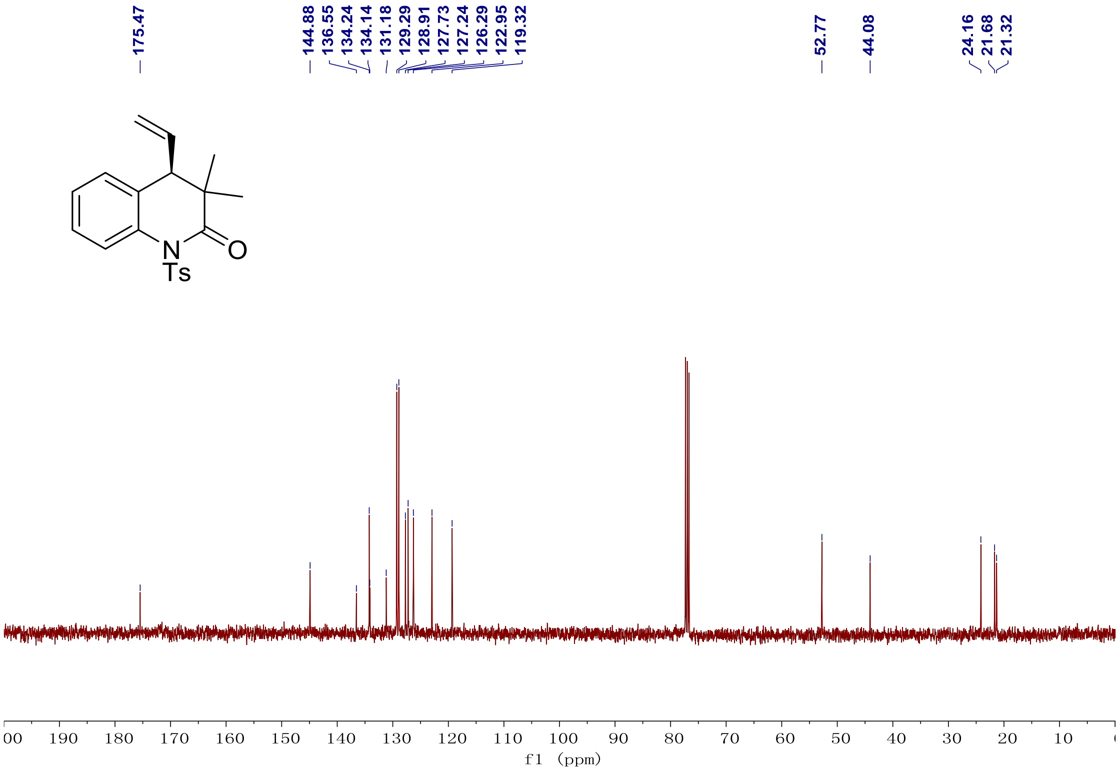

Supplementary Figure 35. ^{1}H and ^{13}C NMR of compound **5a**

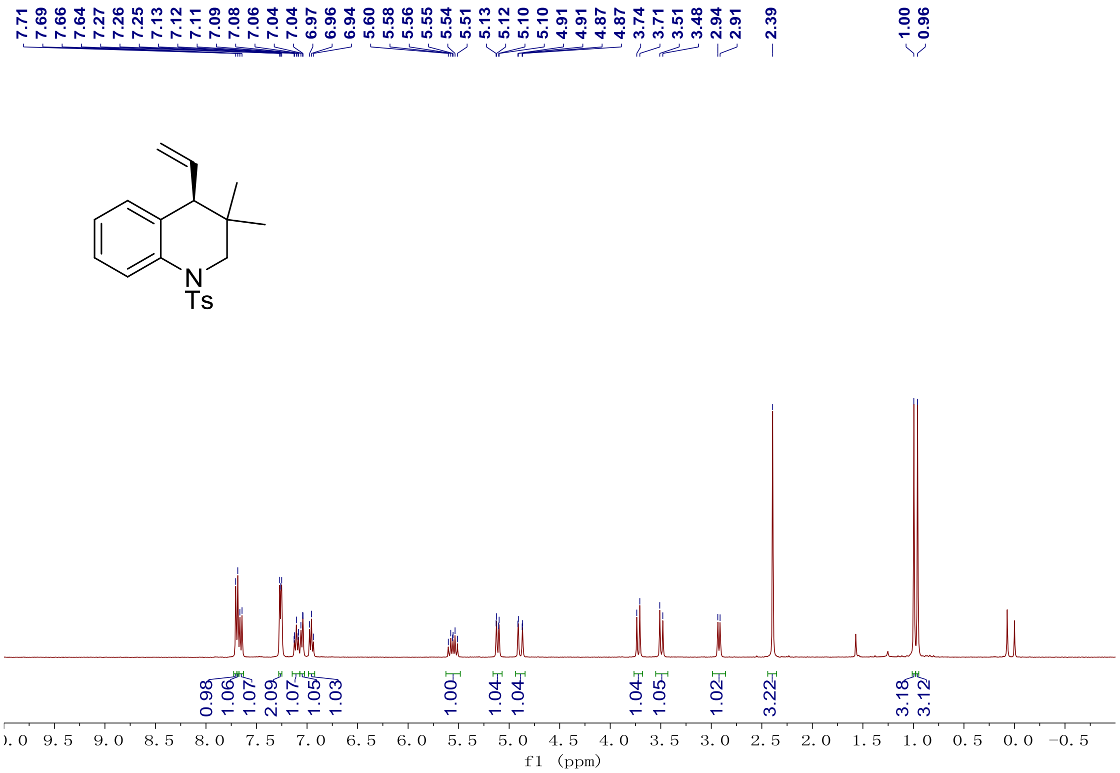

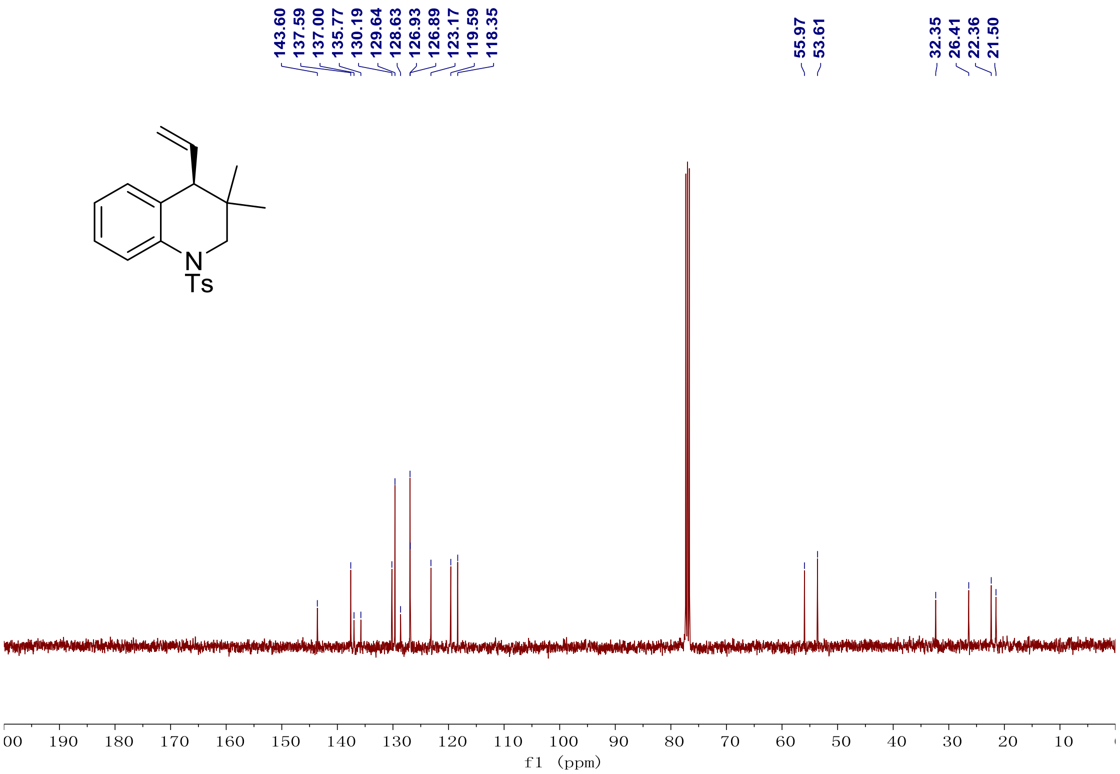

Supplementary Figure 36. ^{1}H and ^{13}C NMR of compound **5b**

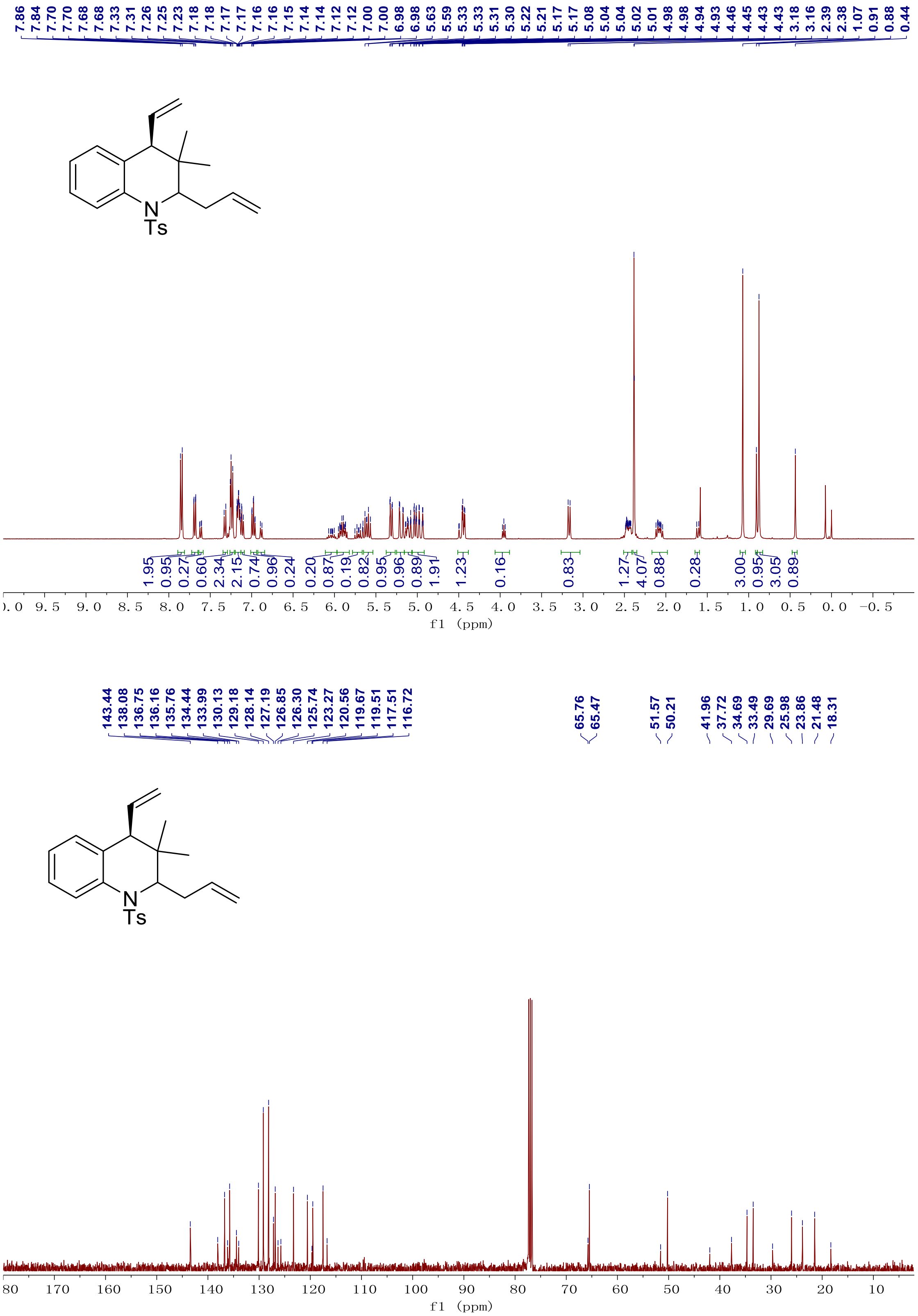

Supplementary Figure 37. ^{1}H and ^{13}C NMR of compound **5c**

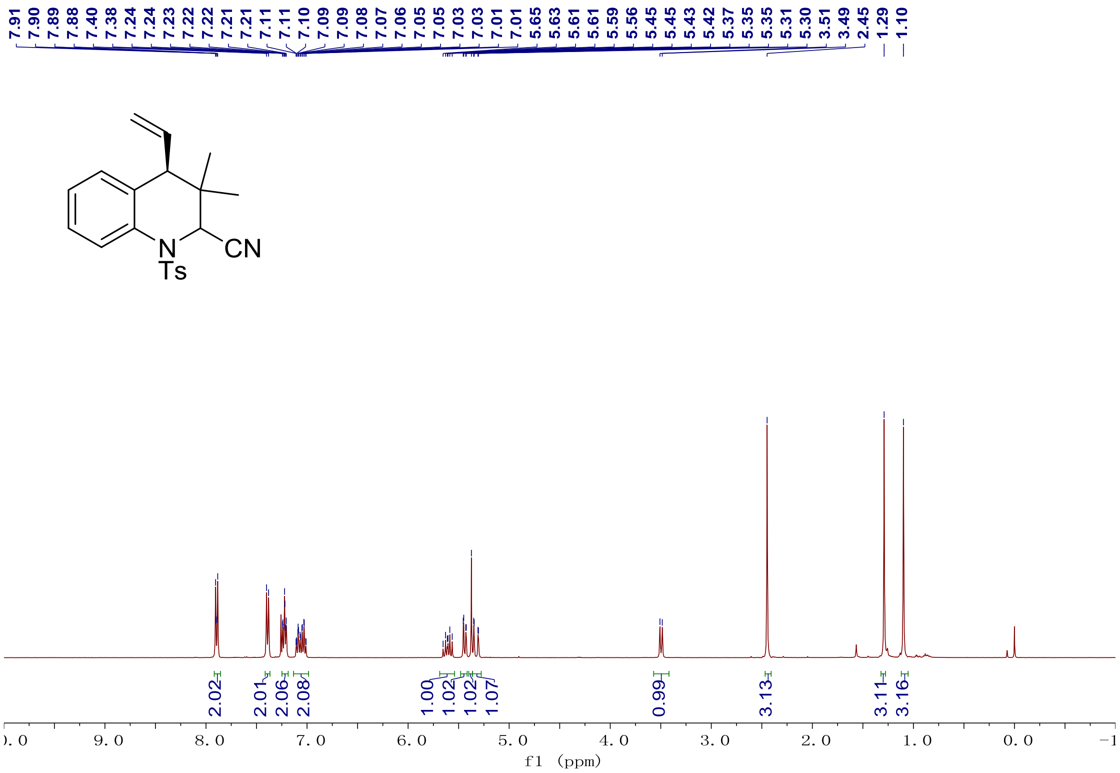

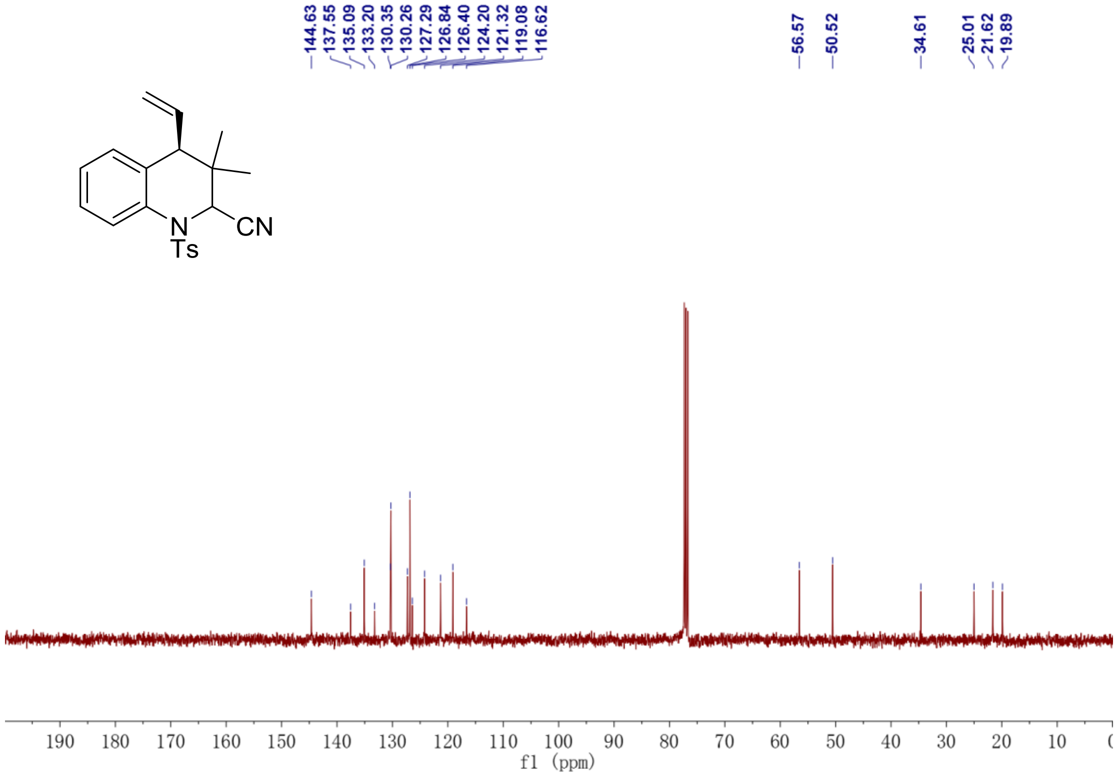

Supplementary Figure 38. ^{1}H and ^{13}C NMR of compound **5d**

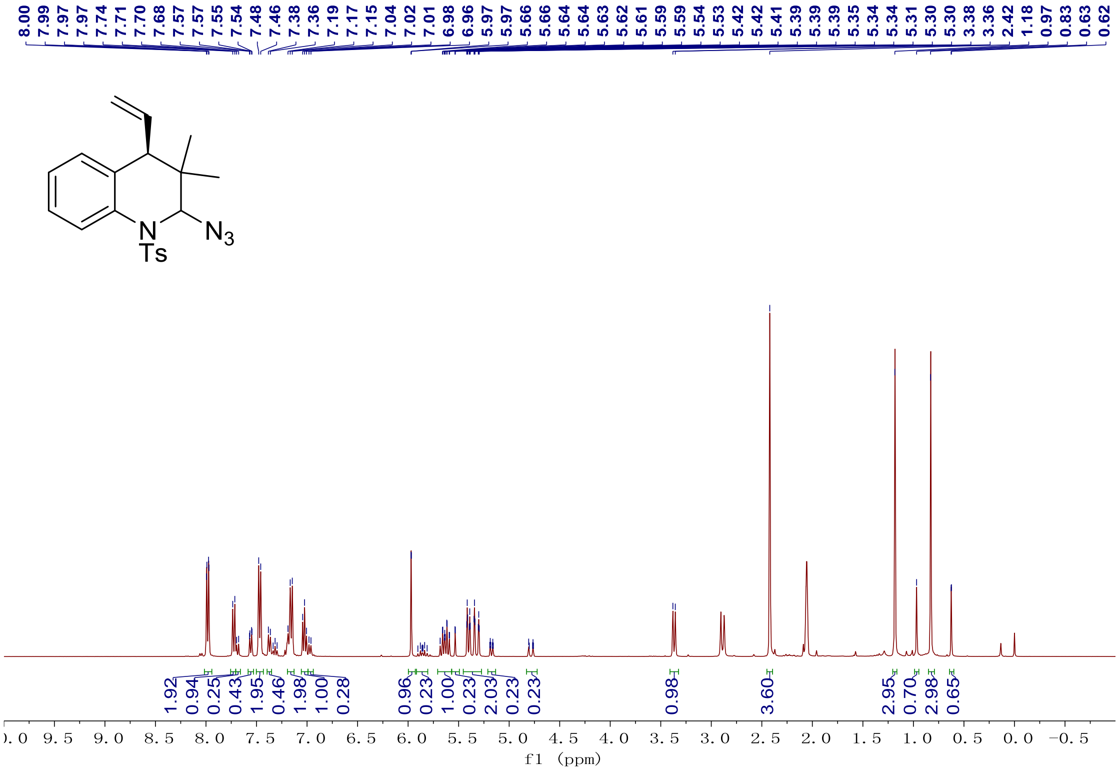

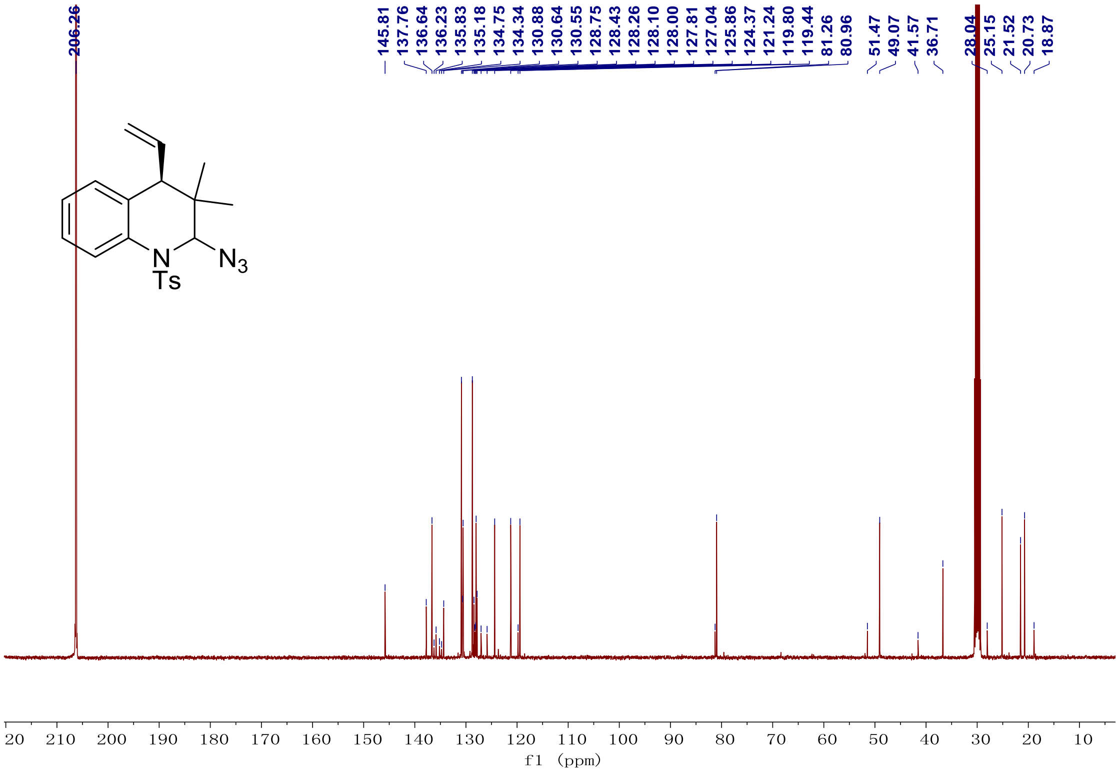

Supplementary Figure 39. ^{1}H and ^{13}C NMR of compound **5e** (in Acetone-*d*6)

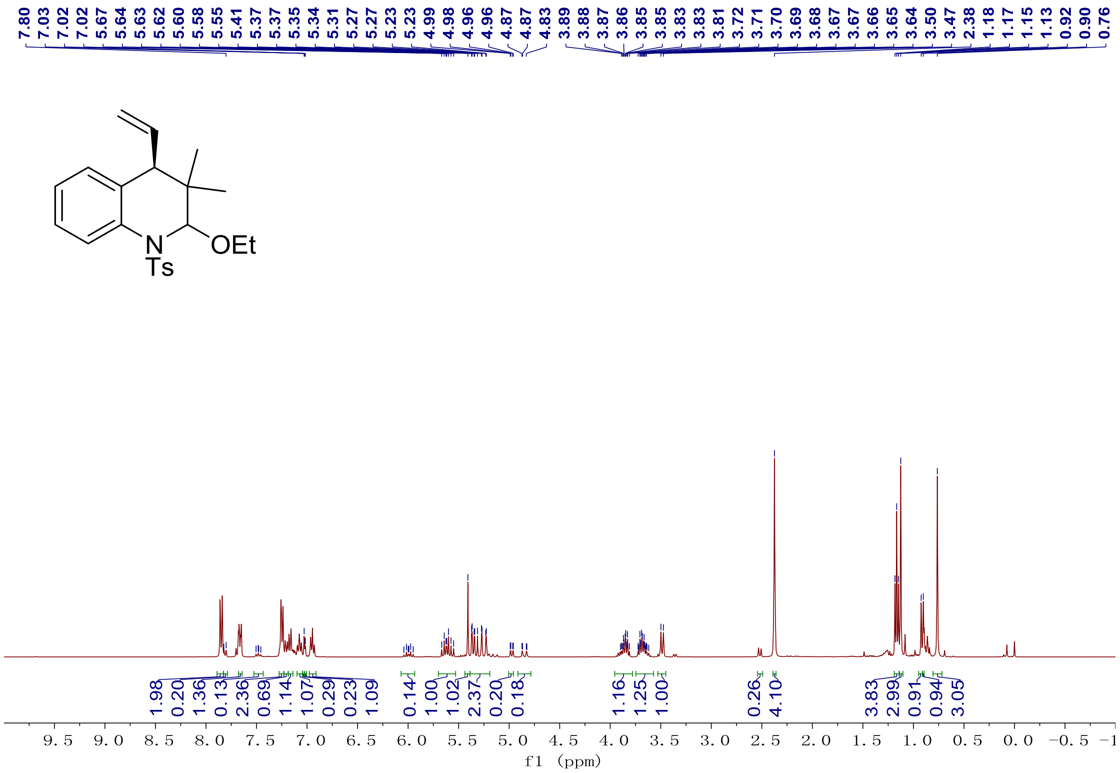

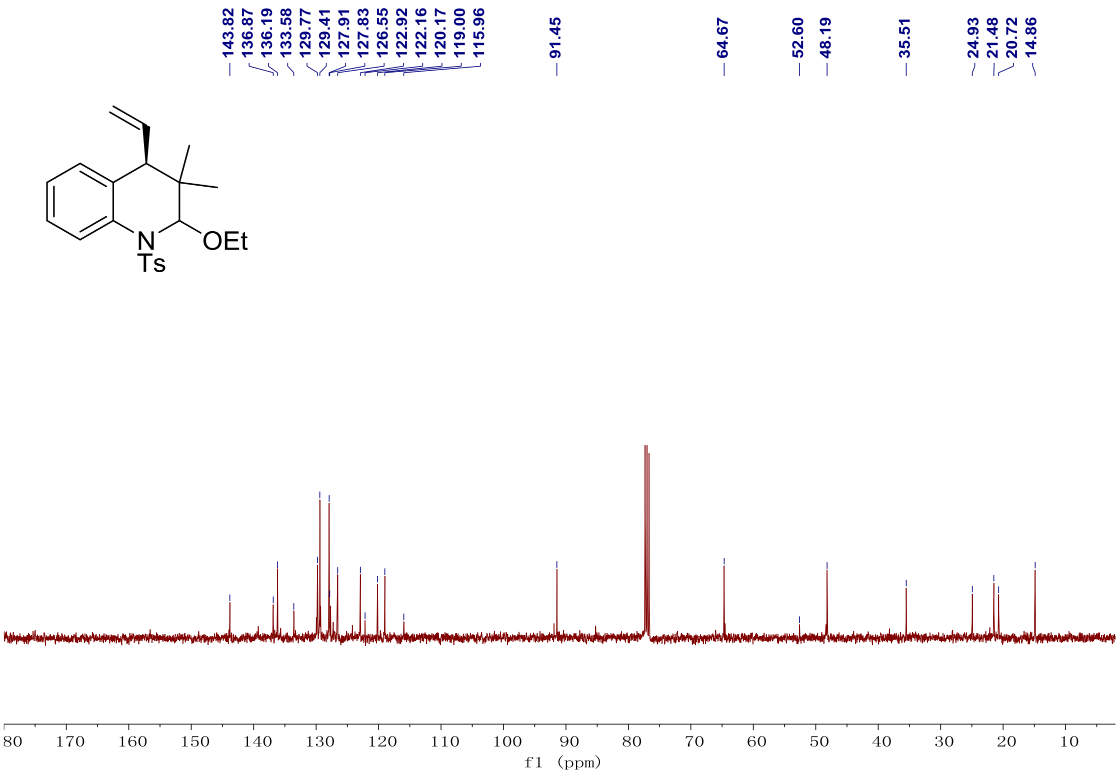

Supplementary Figure 40. ^{1}H and ^{13}C NMR of compound **5f**

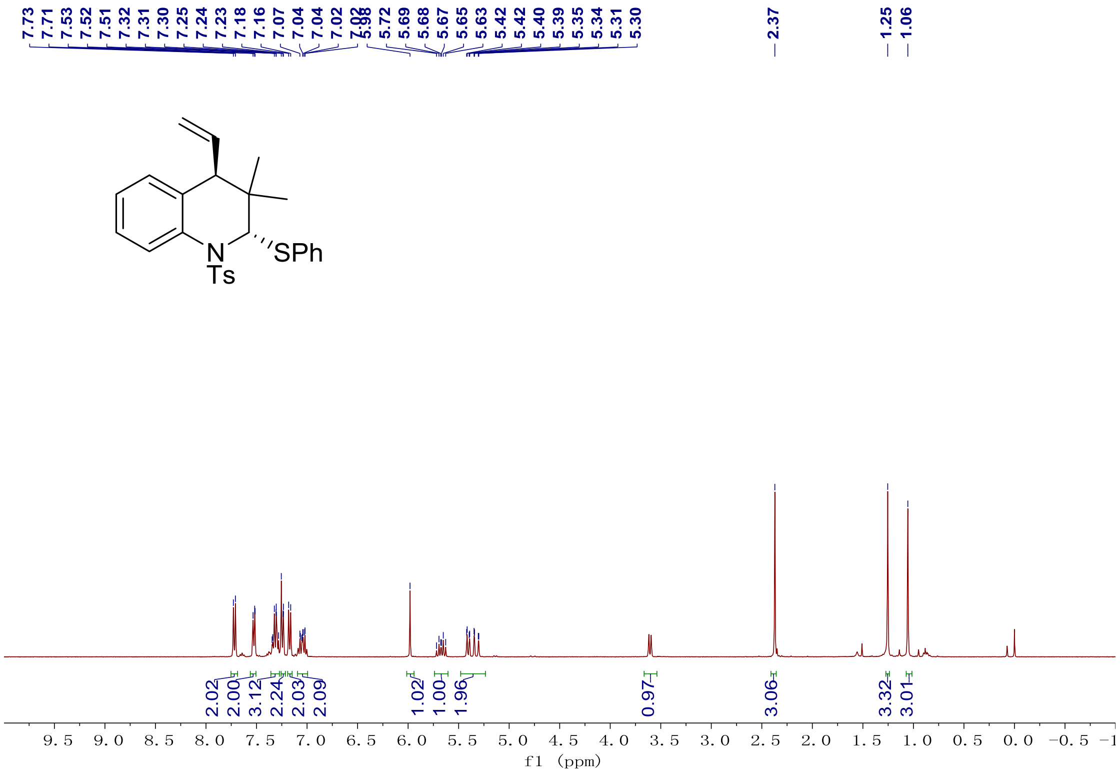

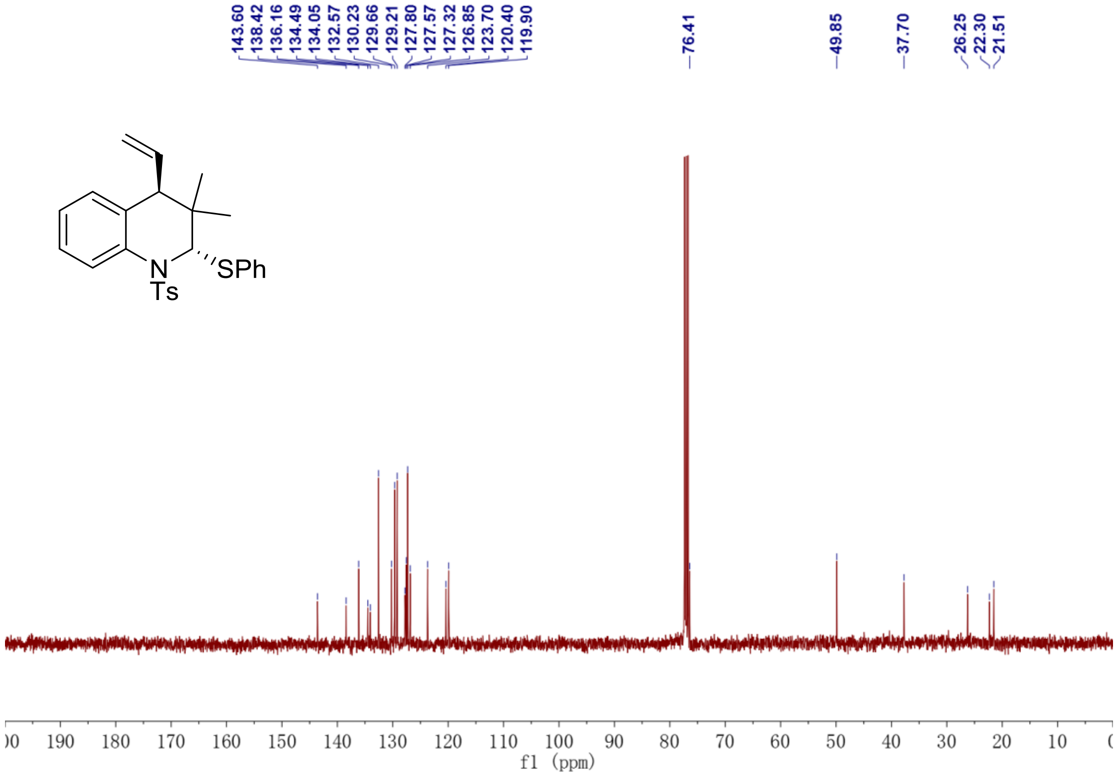

Supplementary Figure 41. ^{1}H and ^{13}C NMR of compound **5g**

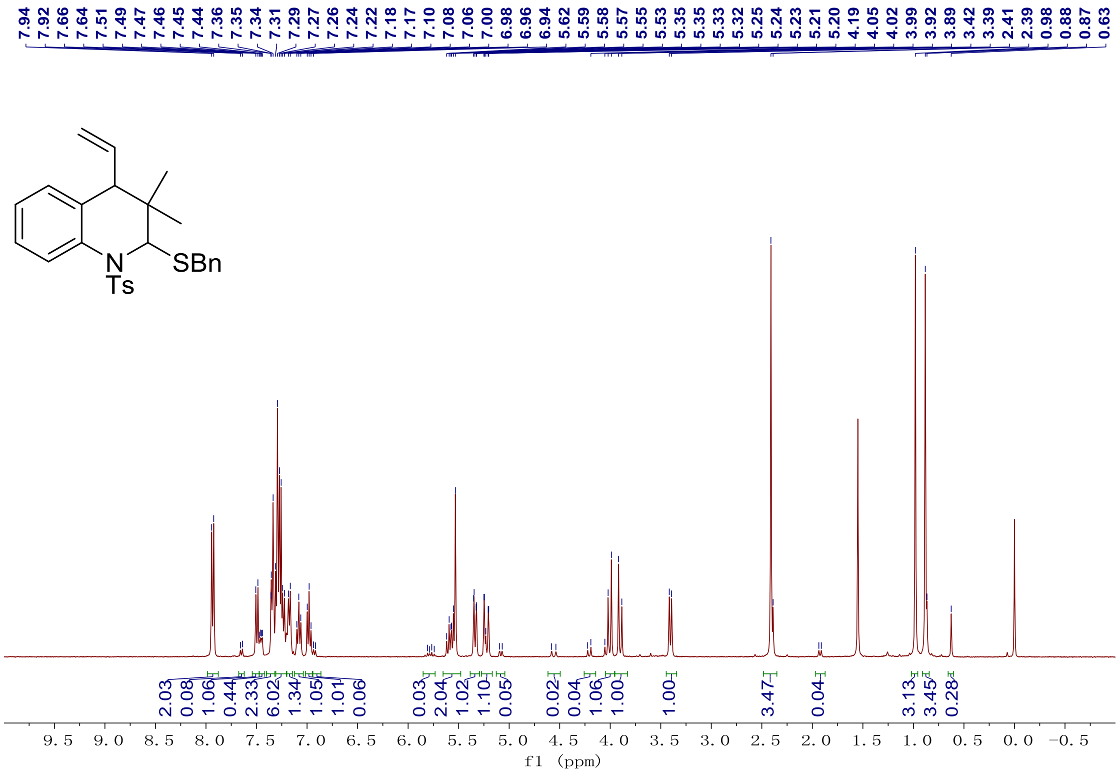

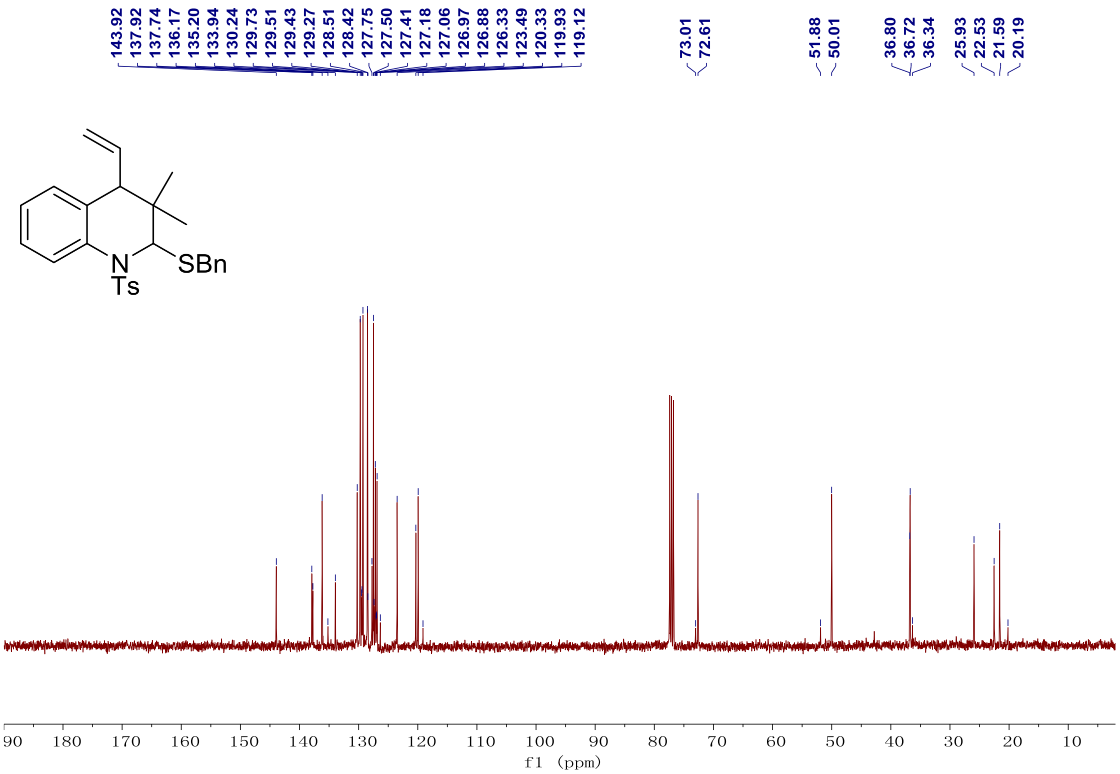

Supplementary Figure 42. ^{1}H and ^{13}C NMR of compound **5h**

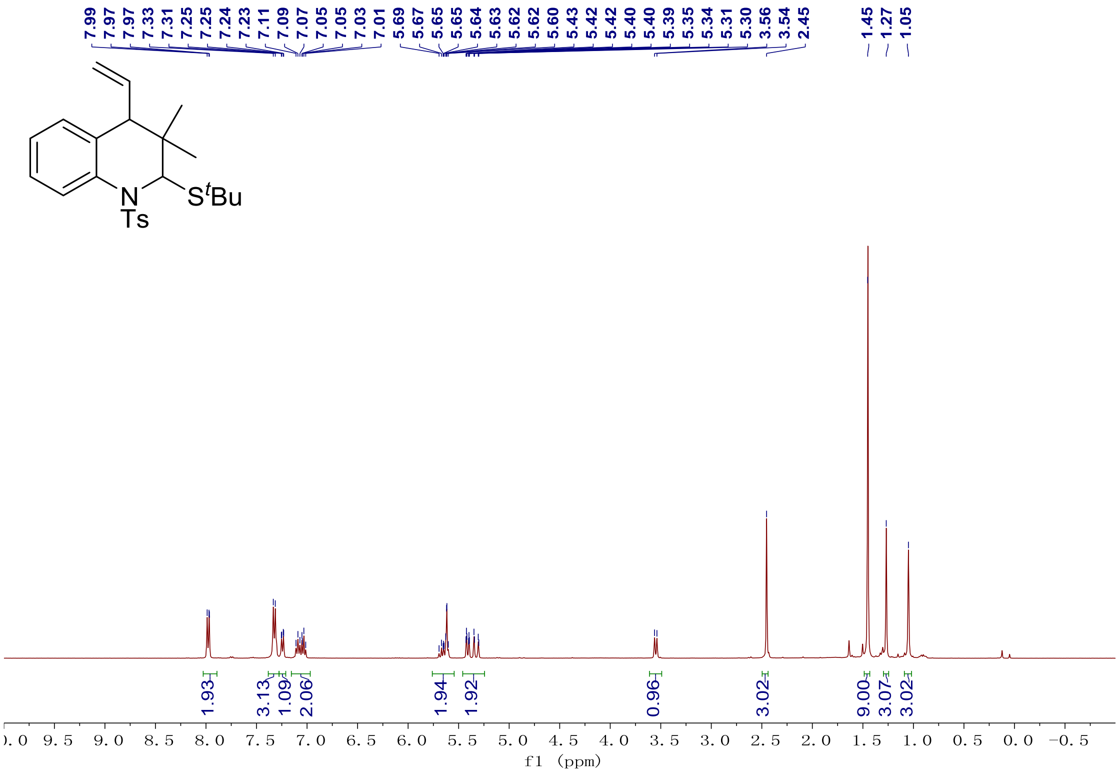

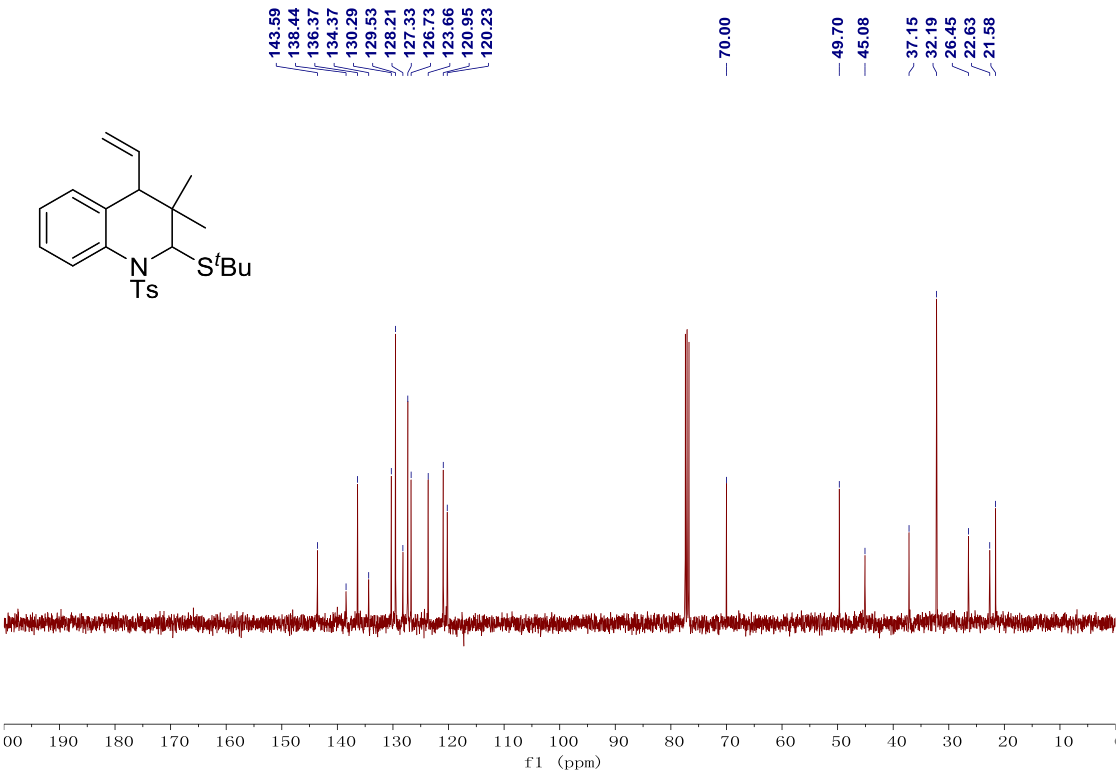

Supplementary Figure 43. ^{1}H and ^{13}C NMR of compound **5i**

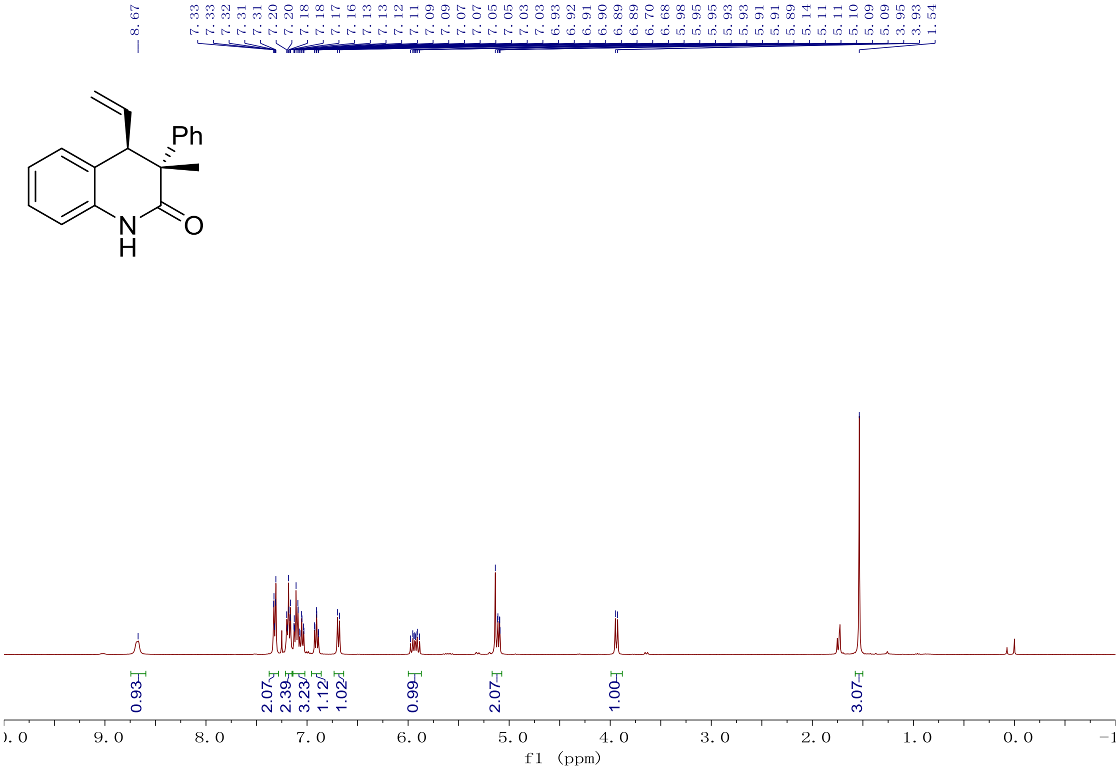

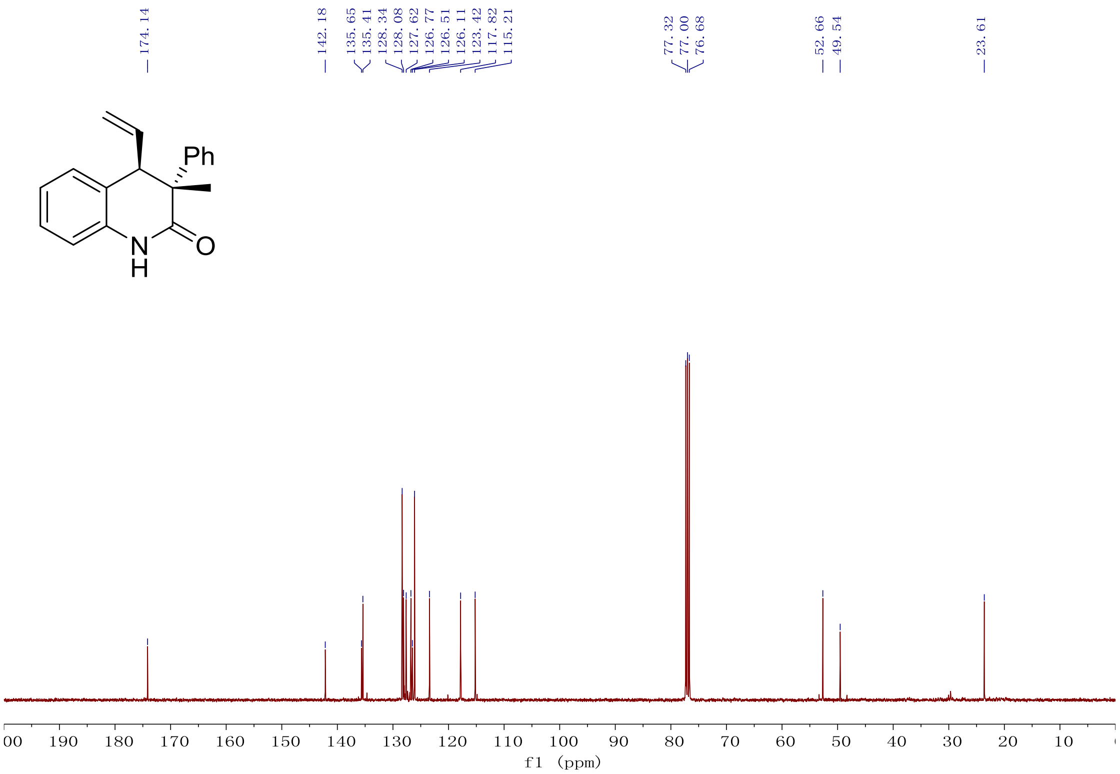

Supplementary Figure 43. ^{1}H and ^{13}C NMR of compound **8aa**

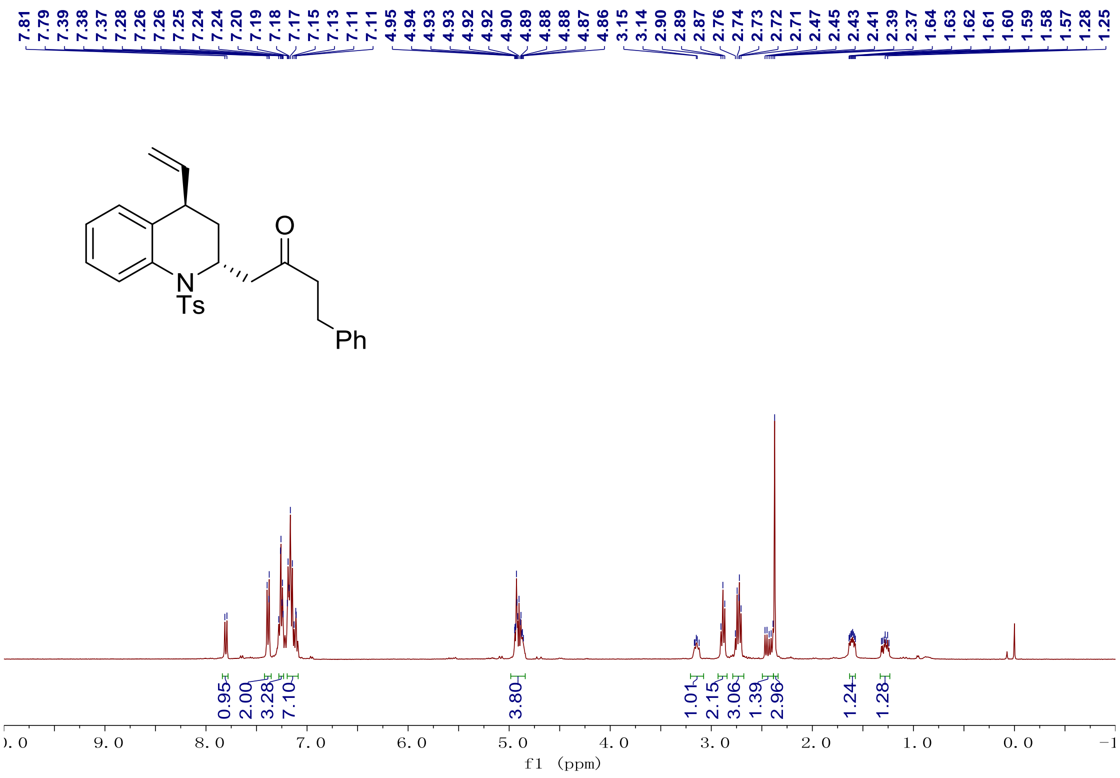

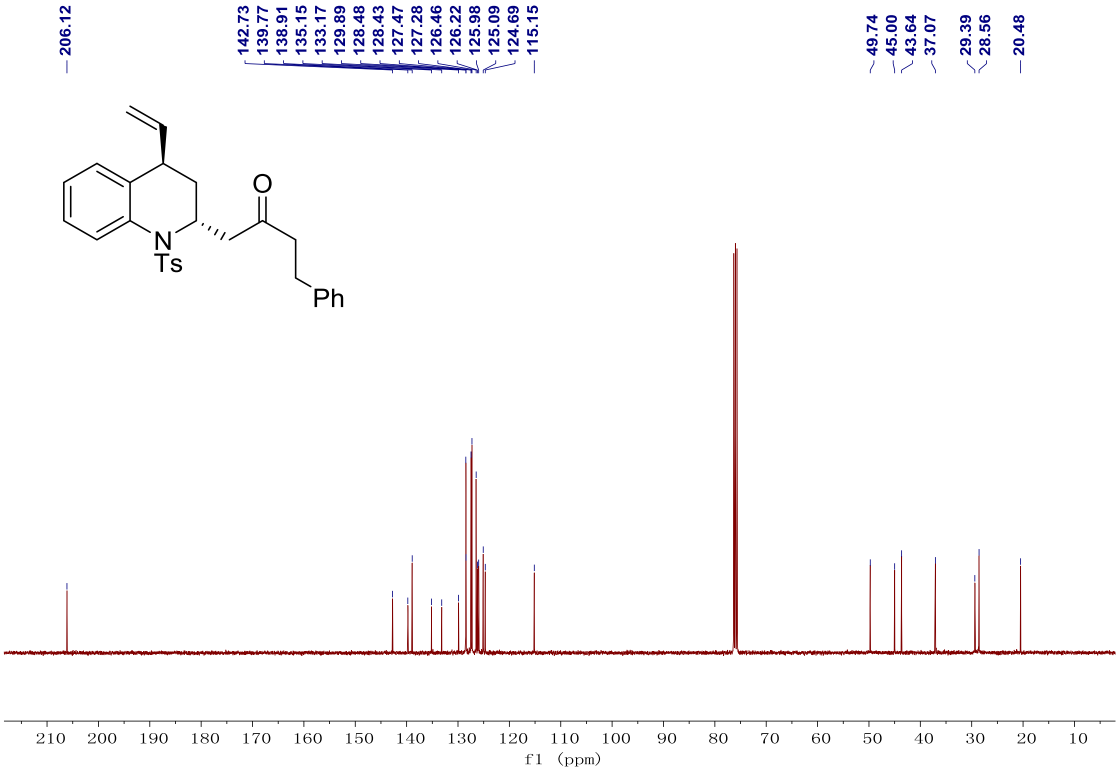

Supplementary Figure 44. ^{1}H and ^{13}C NMR of compound ***(R,R)*-7a**

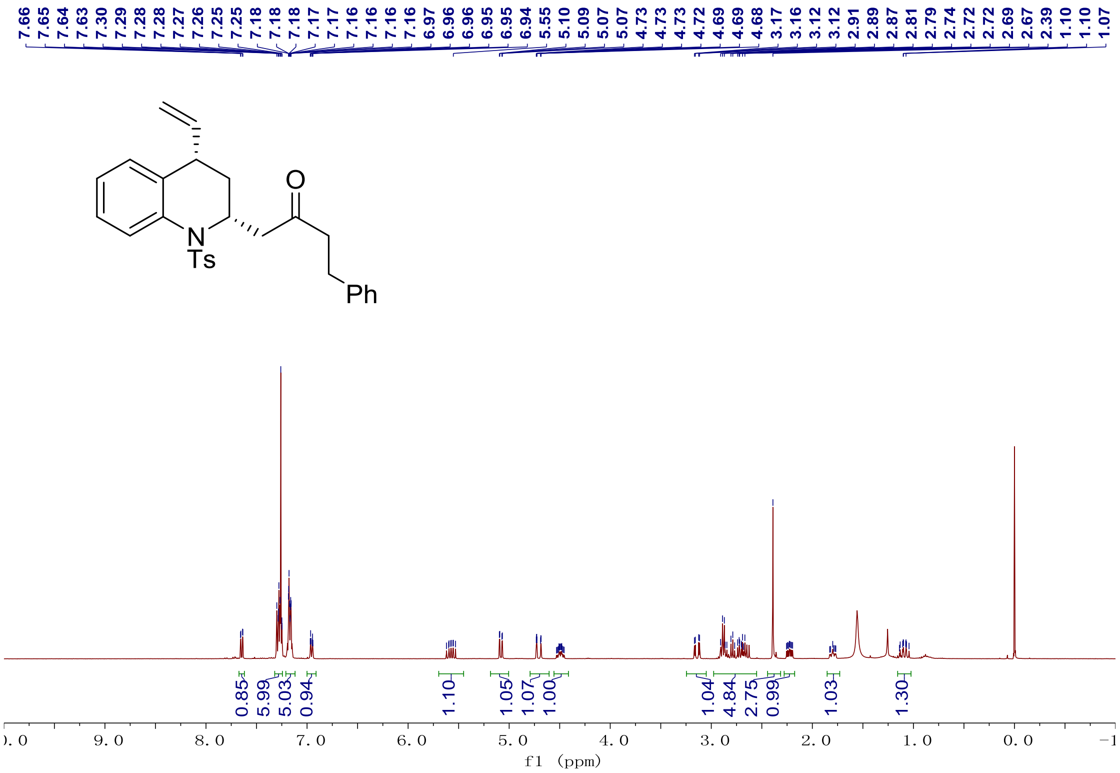

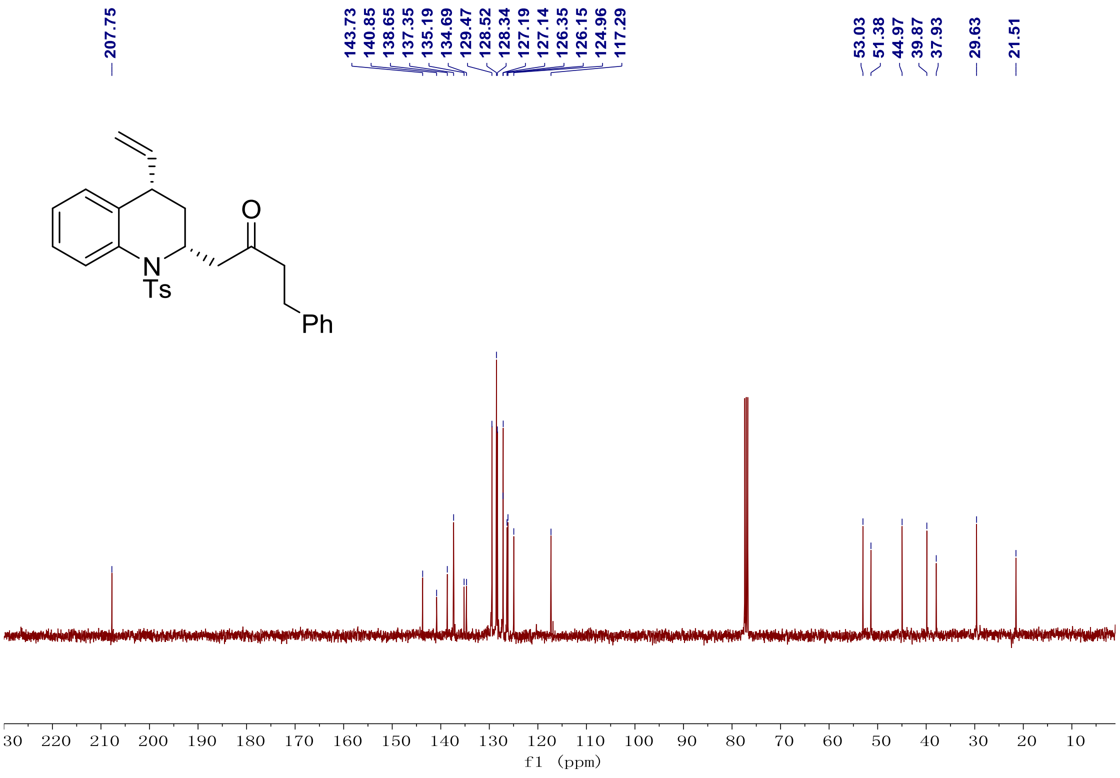

Supplementary Figure 45. ^{1}H and ^{13}C NMR of compound **(*R*,*S*)-7a**

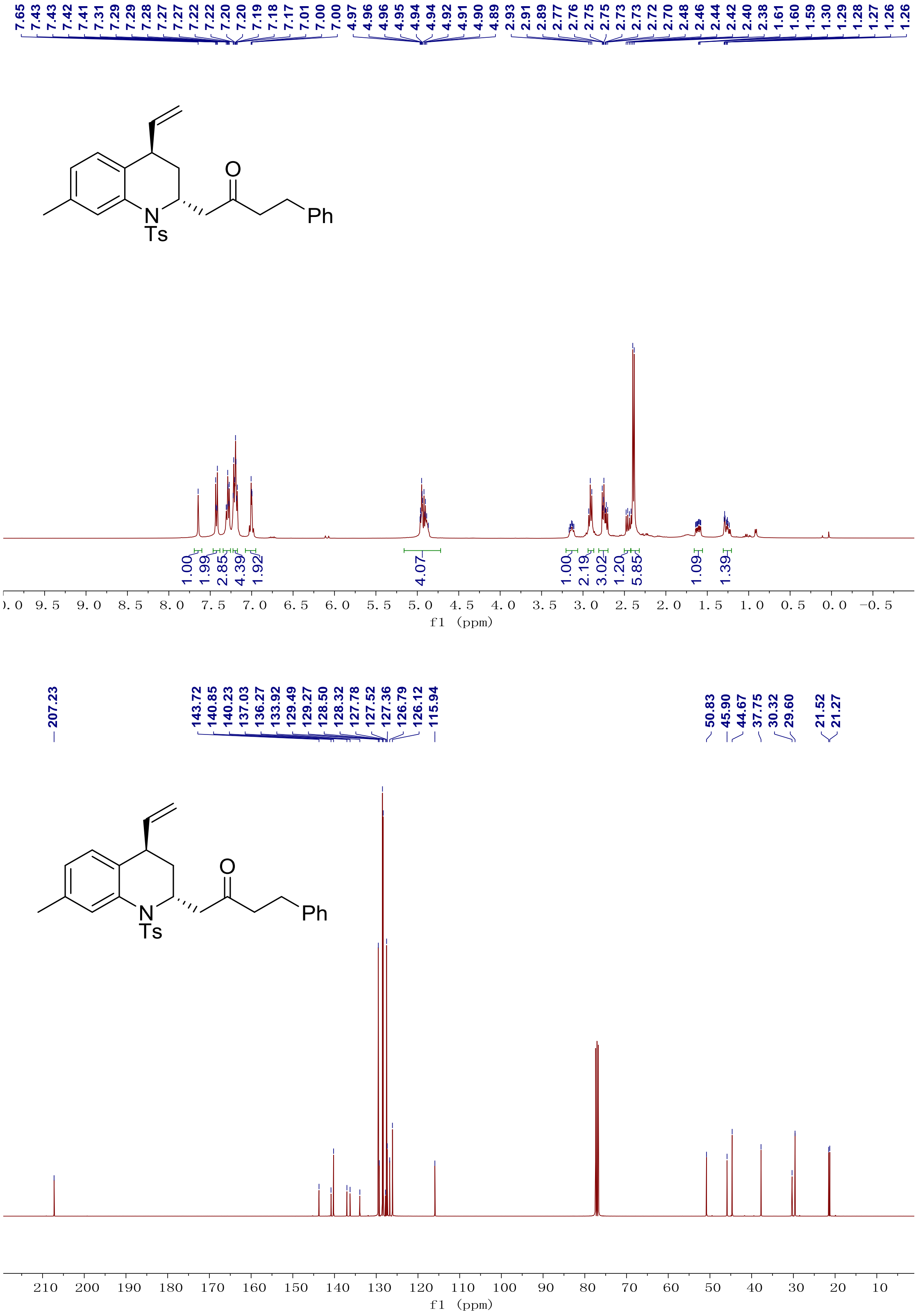

Supplementary Figure 46. ^{1}H and ^{13}C NMR of compound ***(R,R)*-7b**

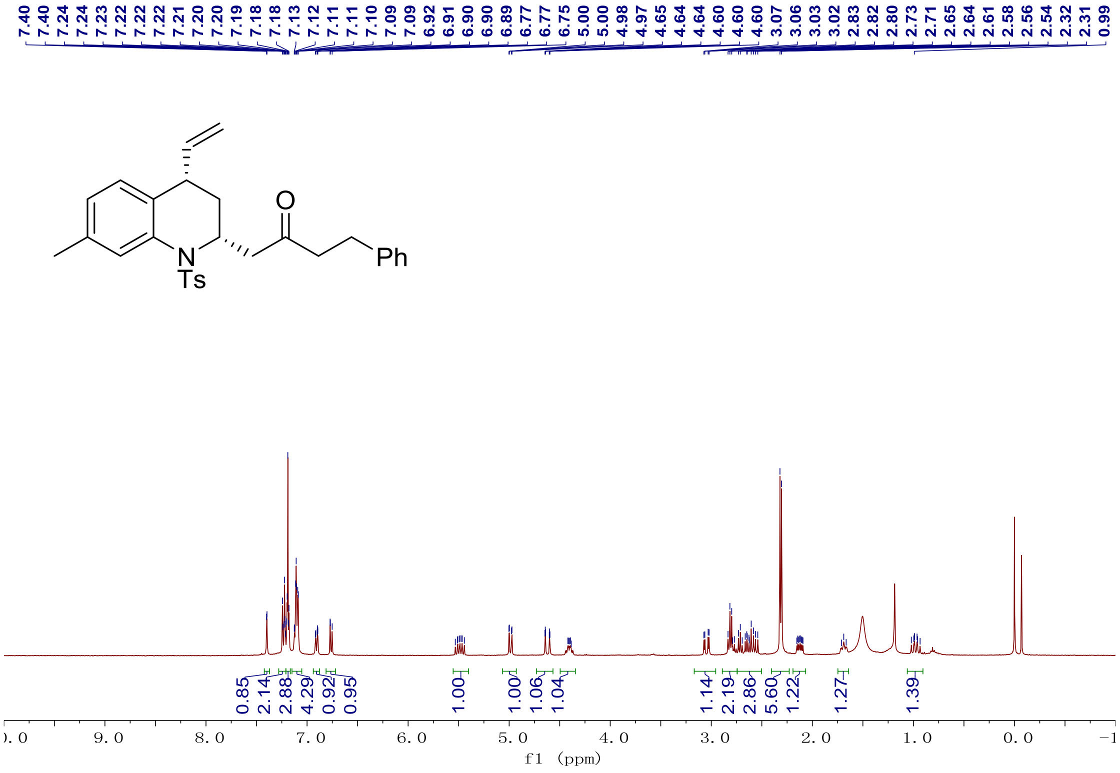

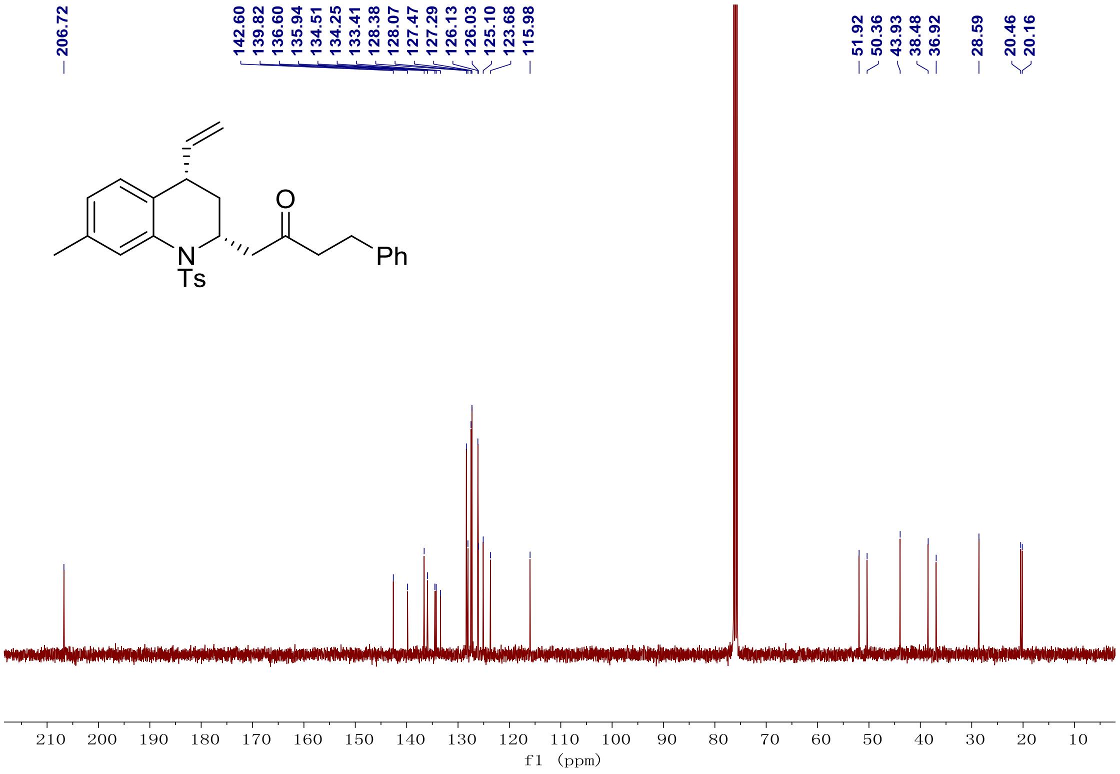

Supplementary Figure 47. ^{1}H and ^{13}C NMR of compound **(*R*,*S*)-7b**

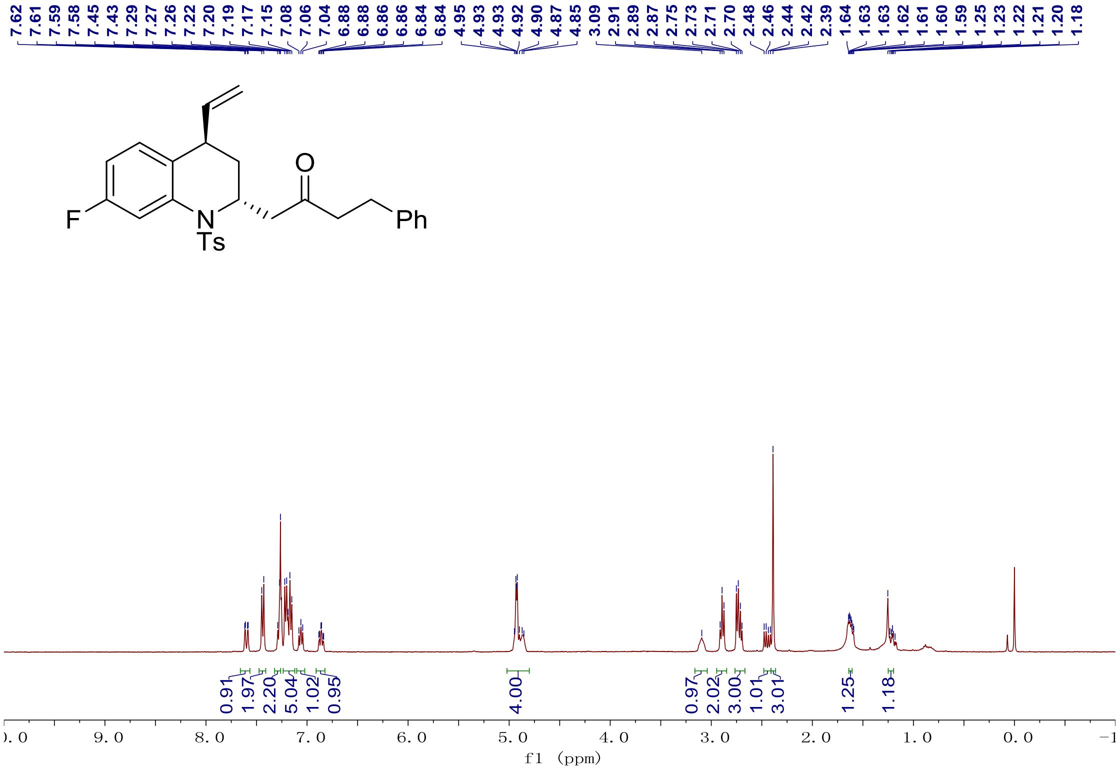
7.62 7.61 7.59 7.58 7.45 7.43 7.29 7.27 7.26 7.22 7.20 7.19 7.17 7.15 7.08 7.06 7.04 6.88 6.88 6.86 6.86 6.84 6.84 4.95 4.93 4.93 4.92 4.90 4.87 4.85 3.09 2.91 2.89 2.87 2.75 2.73 2.71 2.70 2.48 2.46 2.44 2.42 2.39 1.64 1.63 1.63 1.62 1.61 1.60 1.59 1.25 1.23 1.22 1.21 1.20 1.18
F
N
Ts
O
Ph
0.91 1.97 2.20 5.04 1.02 0.95 4.00 0.97 2.02 3.00 1.01 3.01 1.25 1.18
9.0 8.0 7.0 6.0 5.0 4.0 3.0 2.0 1.0 0.0
f1 (ppm)

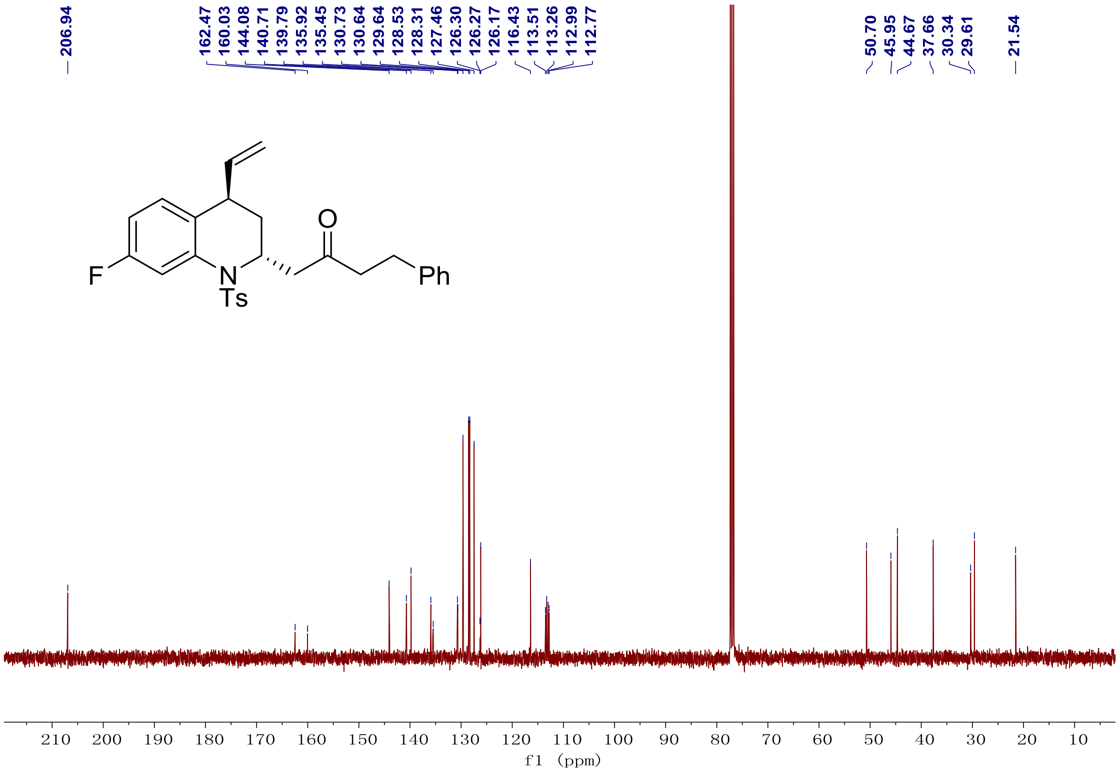
206.94 162.47 160.03 144.08 140.71 139.79 135.92 135.45 130.73 130.64 129.64 128.53 128.31 127.46 126.30 126.27 126.17 116.43 113.51 113.26 112.99 112.77 50.70 45.95 44.67 37.66 30.34 29.61 21.54
F
N
Ts
O
Ph
210 200 190 180 170 160 150 140 130 120 110 100 90 80 70 60 50 40 30 20 10
f1 (ppm)

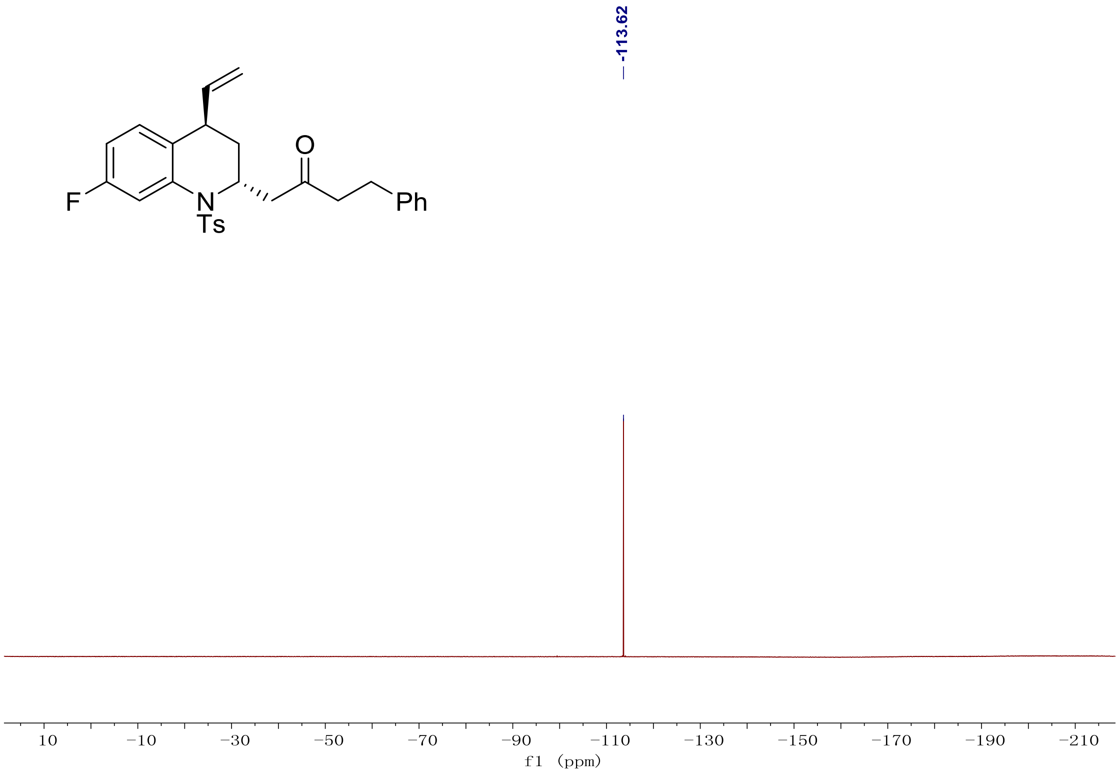

Supplementary Figure 48. ^{1}H and ^{13}C NMR ^{19}F NMR of compound ***(R,R)*-7c**

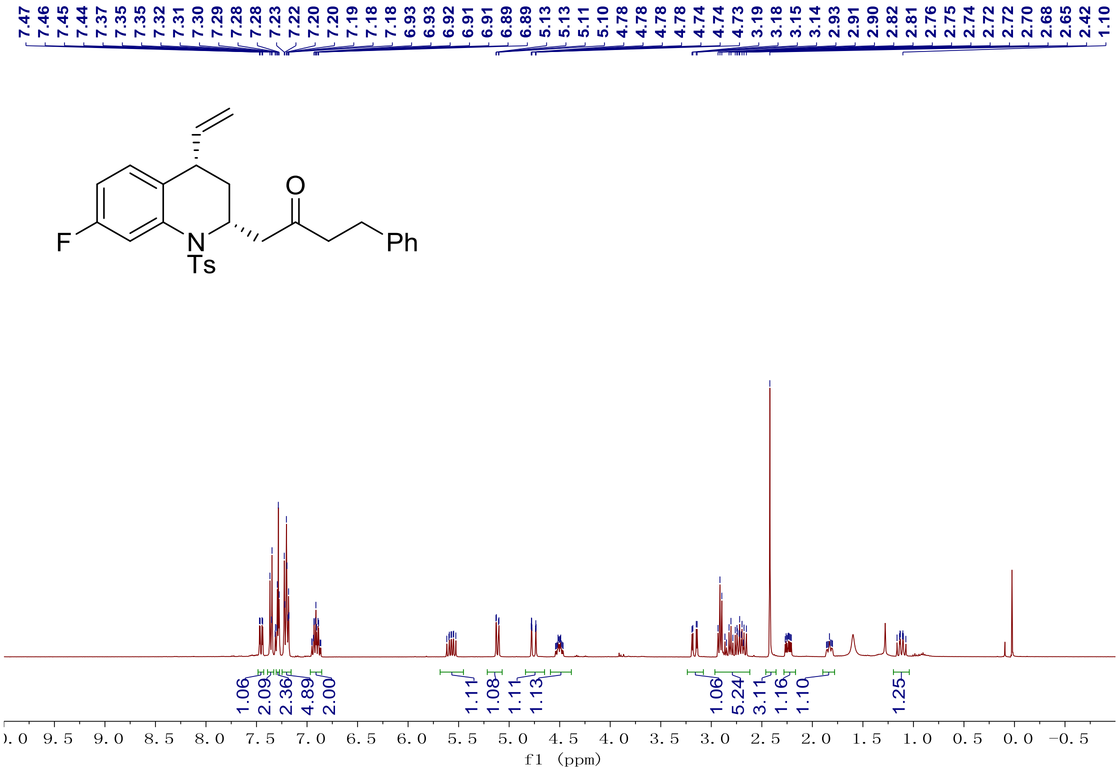

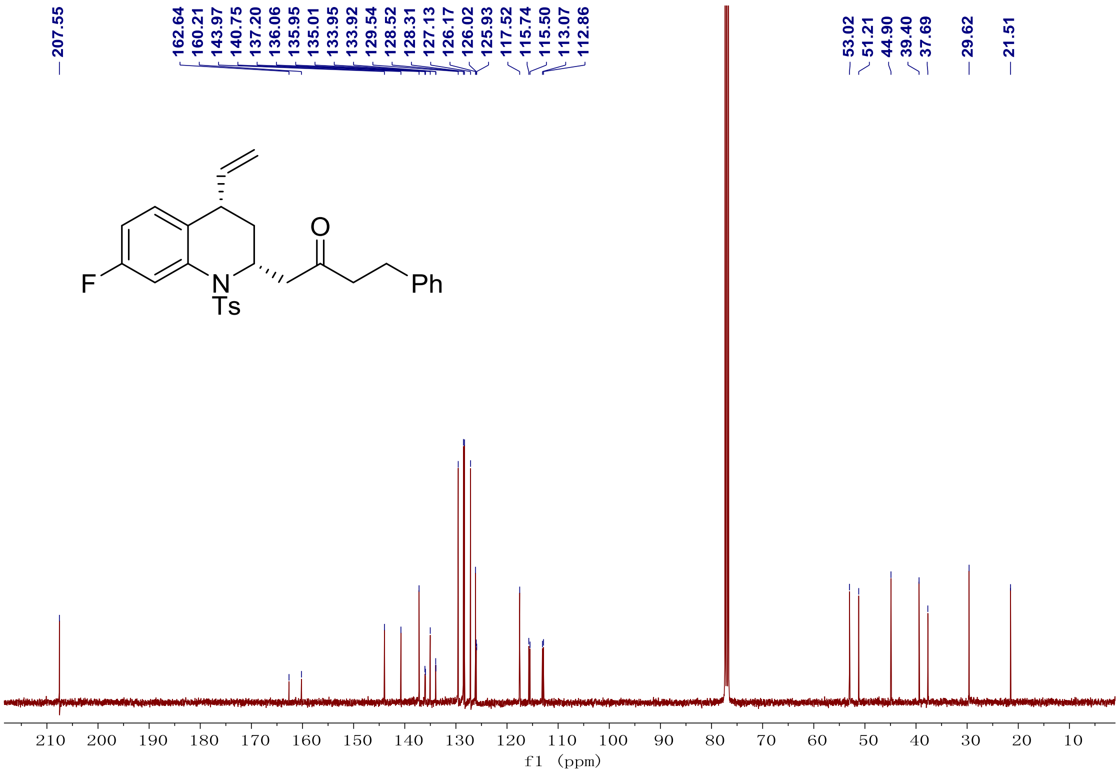

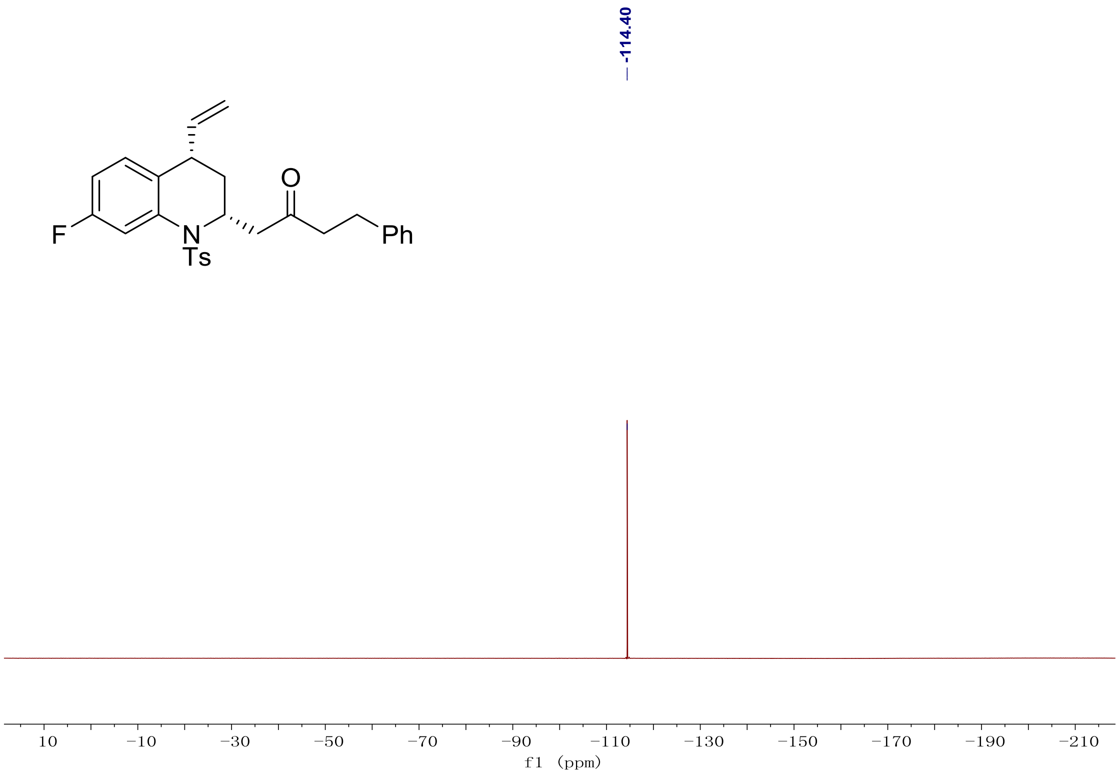

Supplementary Figure 49. ^{1}H and ^{13}C NMR ^{19}F NMR of compound **(*R*,*S*)-7c**

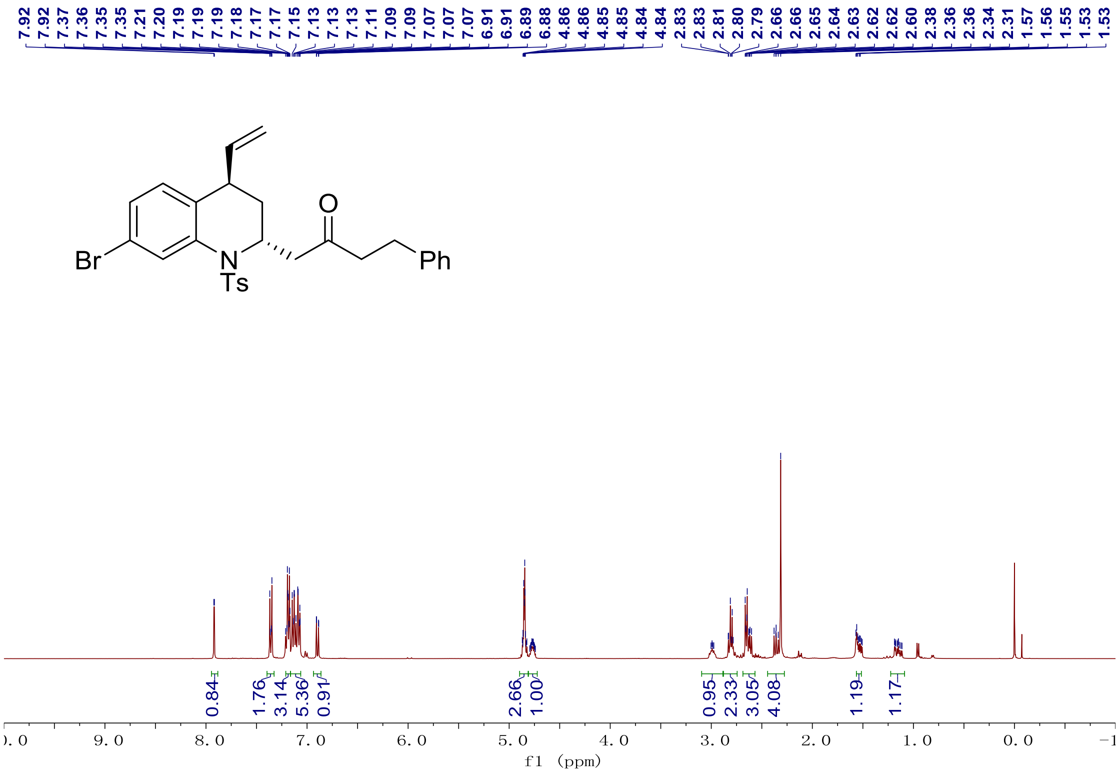

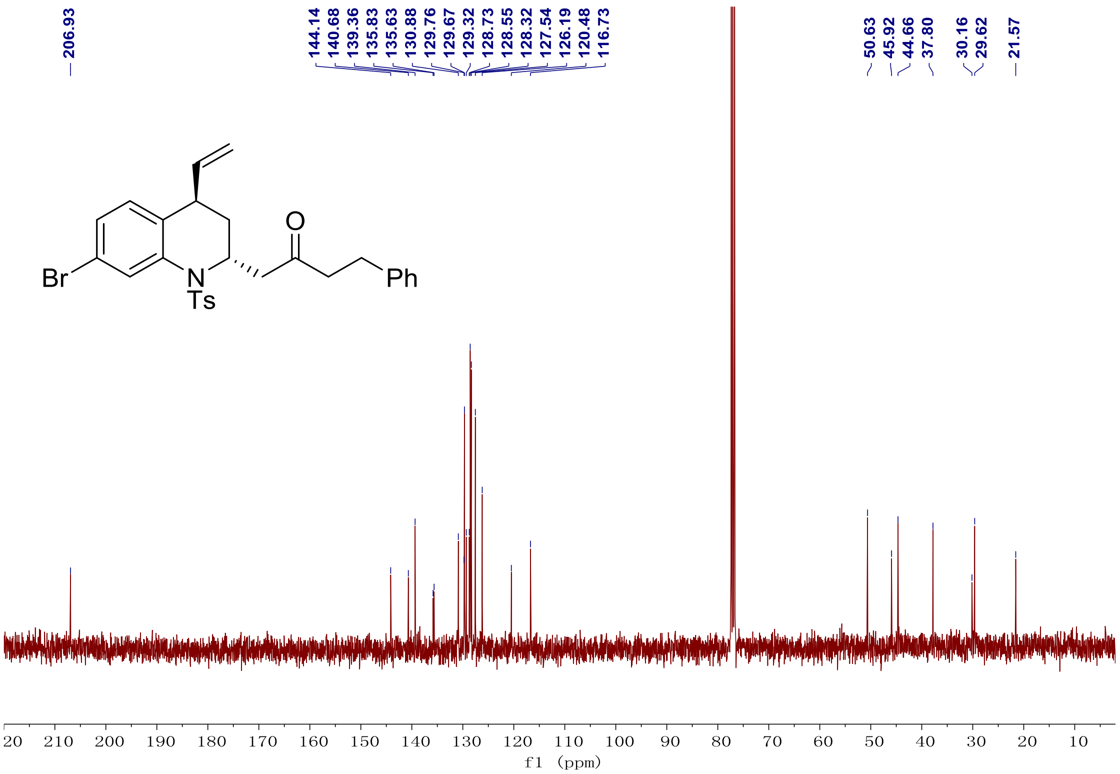

Supplementary Figure 50. ^{1}H and ^{13}C NMR of compound **(*R*,*R*)-7d**

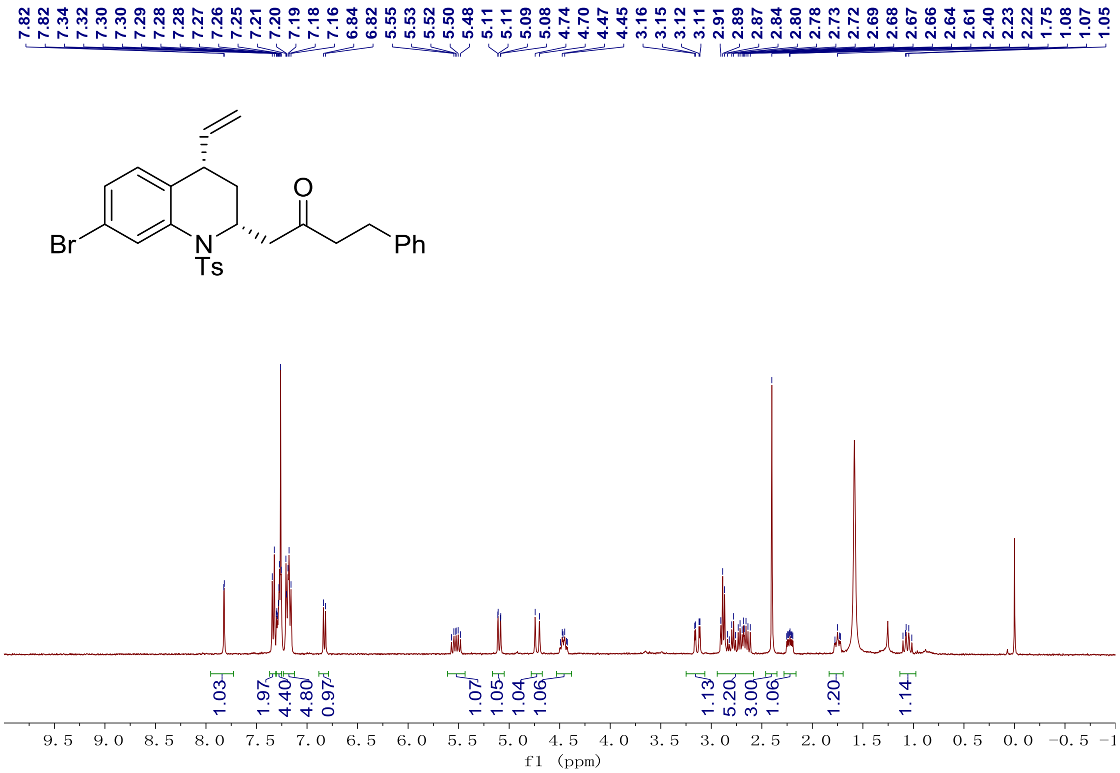

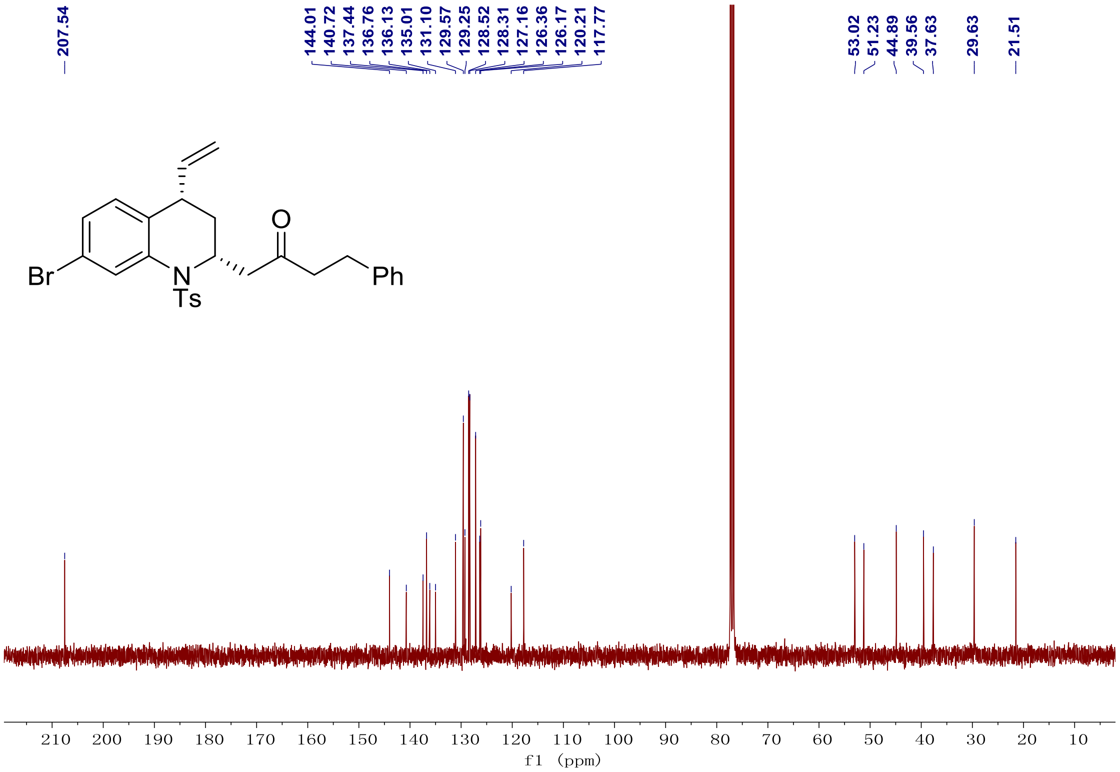

Supplementary Figure 51. ^{1}H and ^{13}C NMR of compound **(*R*,*S*)-7d**

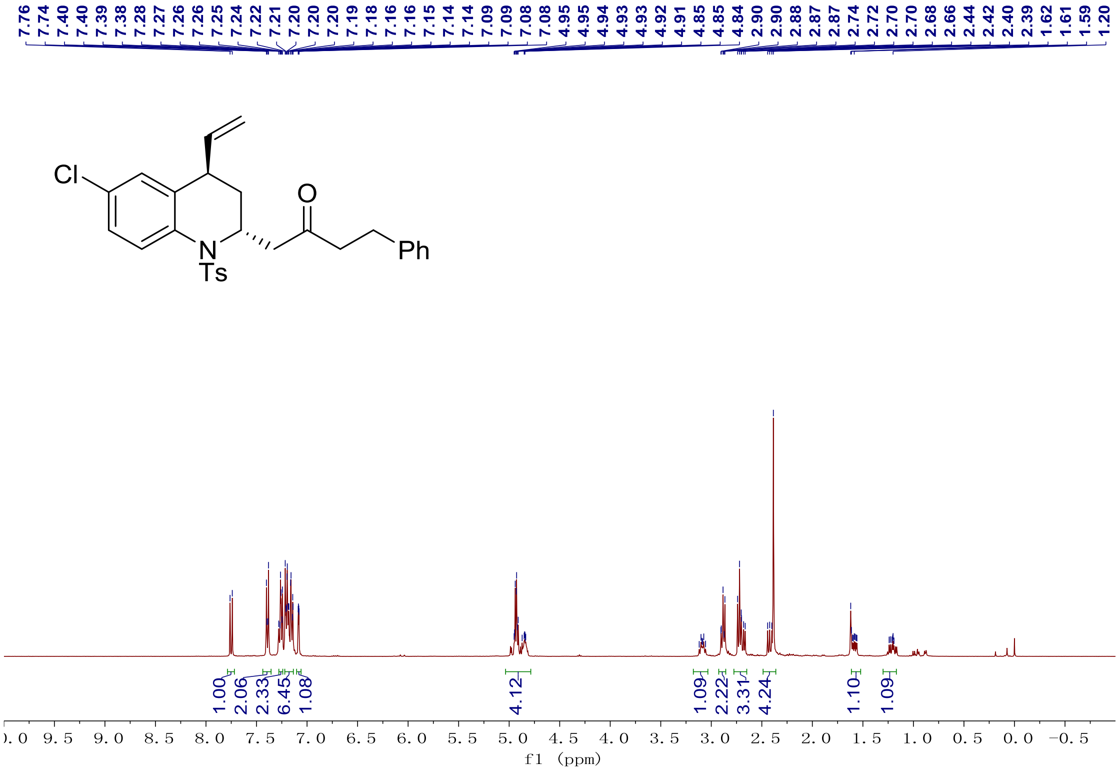

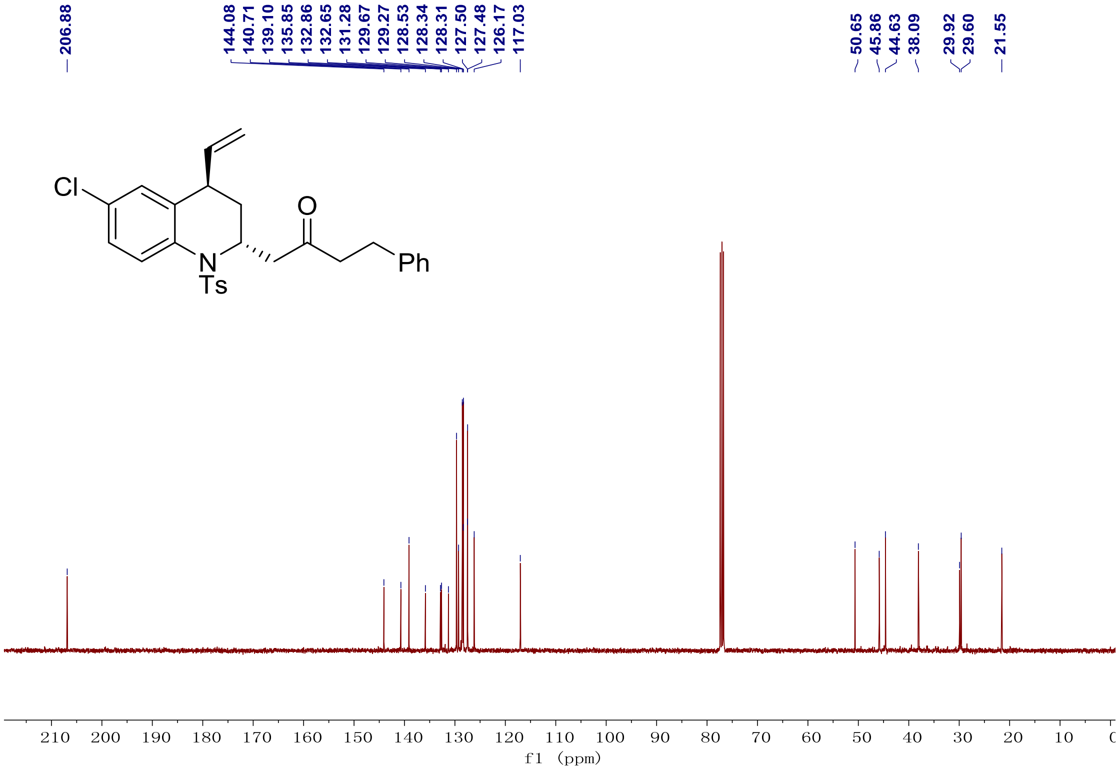

Supplementary Figure 52. ^{1}H and ^{13}C NMR of compound **(*R*,*R*)-7e**

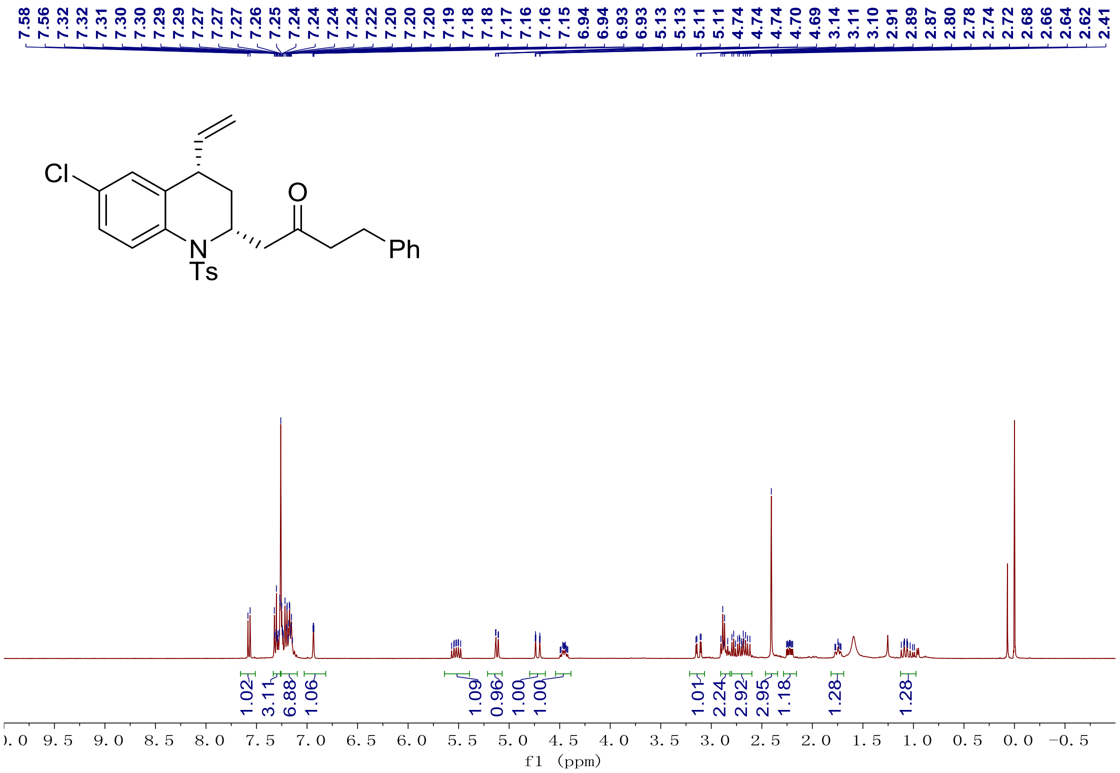

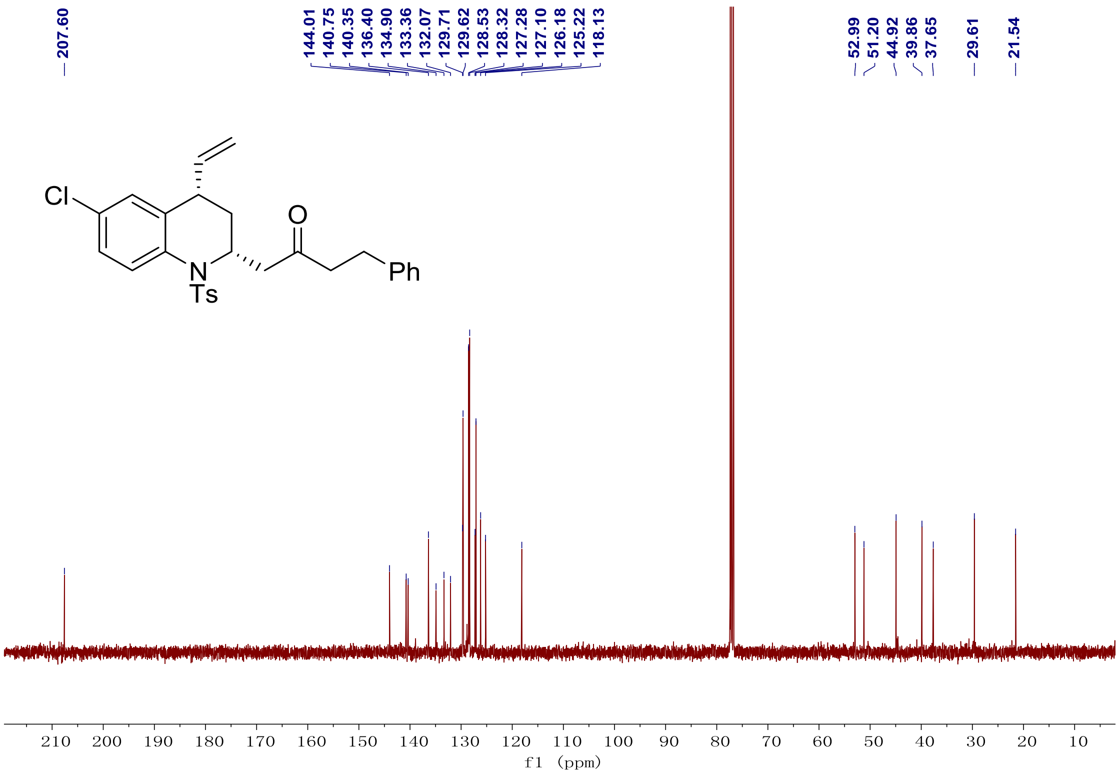

Supplementary Figure 53. ^{1}H and ^{13}C NMR of compound ***(R,S)*-7e**

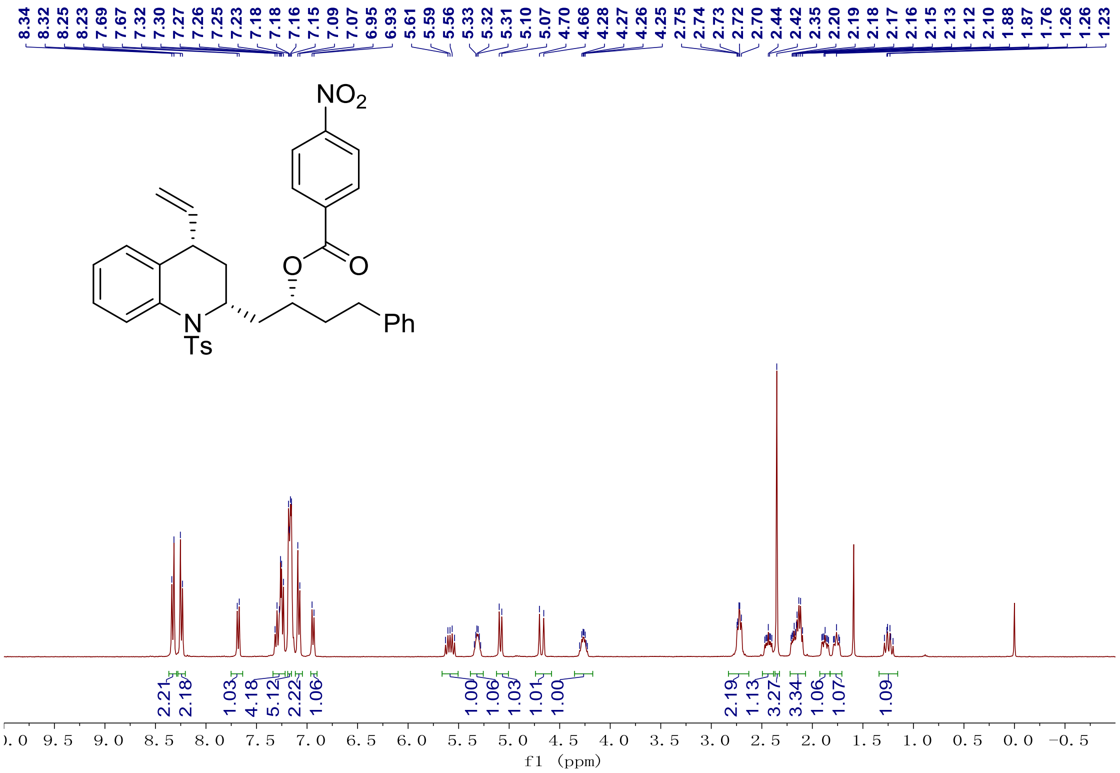

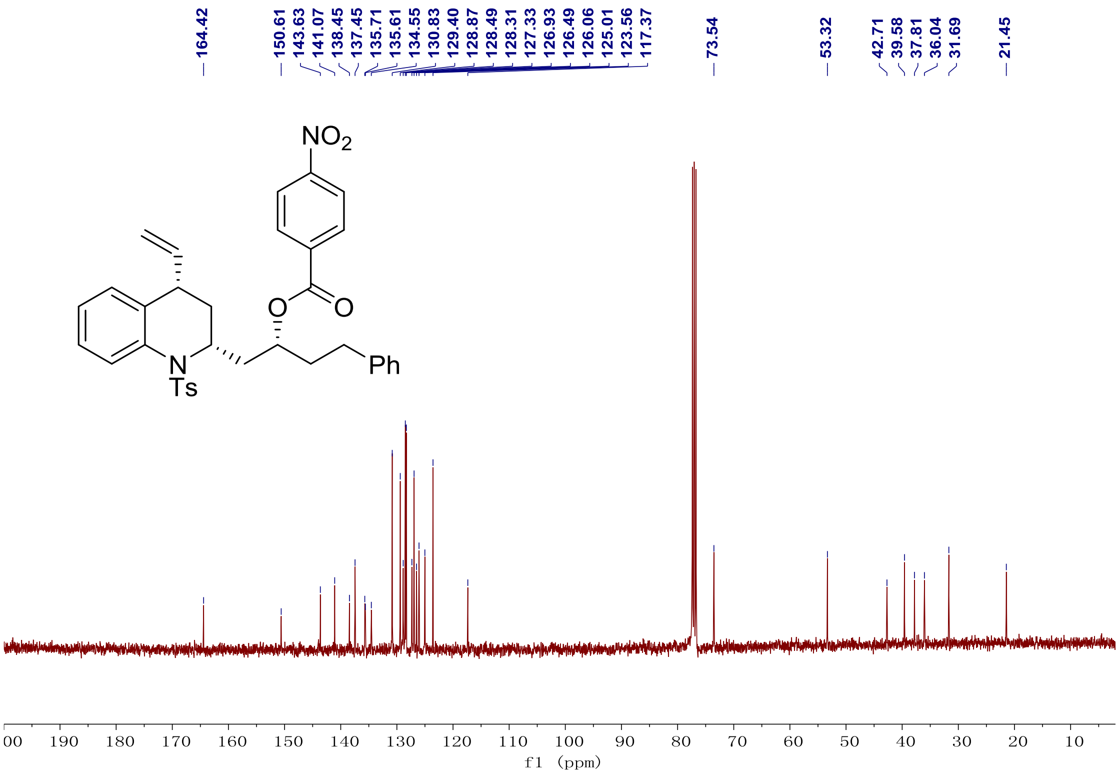

Supplementary Figure 53. ^{1}H and ^{13}C NMR of compound ***(R,S)*-8a**

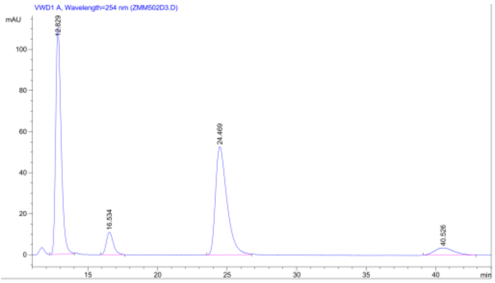

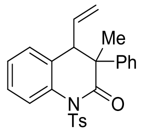

rac-**3aa**

Peak #	RetTime [min]	Type	Width [min]	Area mAU *s	Height [mAU]	Area %
1	12.829	VB	0.4170	3019.25000	110.76018	44.6792
2	16.534	BB	0.5424	385.21014	11.00316	5.7004
3	24.469	BB	0.8645	3000.66895	52.75665	44.4043
4	40.526	BB	1.1861	352.48471	3.59979	5.2161

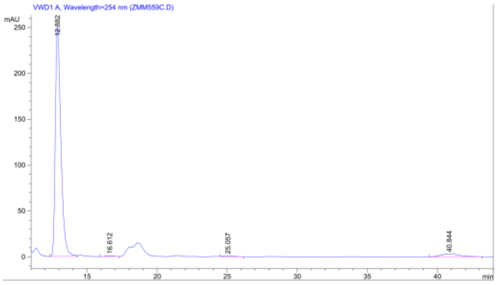

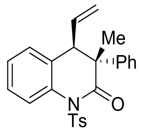

3aa

Peak #	RetTime [min]	Type	Width [min]	Area mAU *s	Height [mAU]	Area %
1	12.882	BB	0.4205	6897.28955	250.26718	94.5796
2	16.612	PV	0.5324	30.51517	7.19916e-1	0.4184
3	25.057	BP	0.6733	49.02715	9.12634e-1	0.6723
4	40.844	BB	1.1960	315.74557	3.15694	4.3297

Supplementary Figure 54. HPLC spectra for compound **3aa**

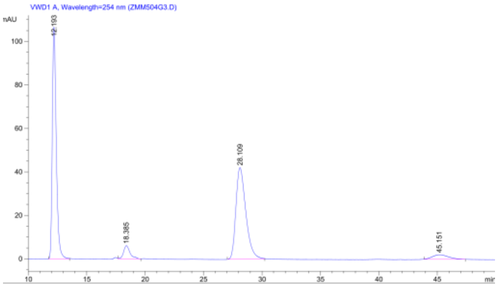

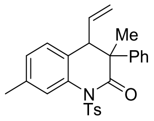

rac-**3ba**

Peak #	RetTime [min]	Type	Width [min]	Area mAU *s	Height [mAU]	Area %
1	12.193	BB	0.3600	2517.23364	106.56134	46.1001
2	18.385	VB	0.5967	240.06221	6.12906	4.3965
3	28.109	BB	0.9080	2491.75781	42.15371	45.6335
4	45.151	BB	1.1722	211.31088	2.14570	3.8699

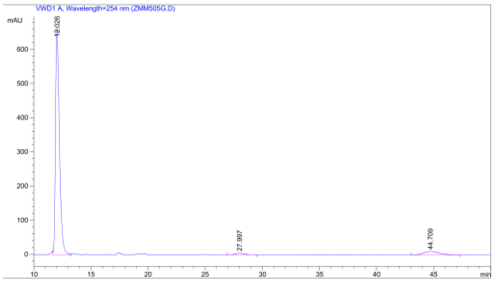

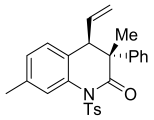

3ba

Peak #	RetTime [min]	Type	Width [min]	Area mAU *s	Height [mAU]	Area %
1	12.026	VV	0.3602	1.57094e4	664.75610	92.2014
2	27.997	BB	0.8567	296.47211	4.88400	1.7401
3	44.709	BB	1.4098	1032.25354	10.38739	6.0585

Supplementary Figure 55. HPLC spectra for compound **3ba**

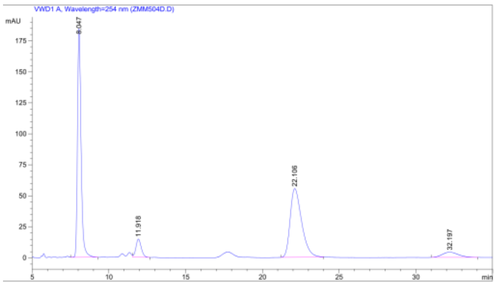

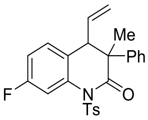

***rac*-3ca**

Peak #	RetTime [min]	Type	Width [min]	Area mAU *s	Height [mAU]	Area %
1	8.047	VB	0.2489	3017.40430	184.59630	45.8119
2	11.918	VP	0.3730	358.26083	14.78578	5.4393
3	22.106	BB	0.8043	2897.93652	55.49719	43.9981
4	32.197	BB	0.9575	312.90143	4.23806	4.7506

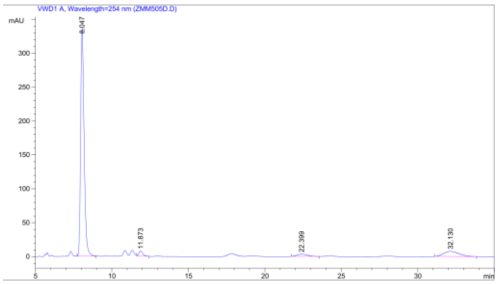

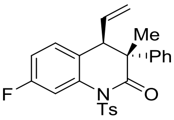

3ca

Peak #	RetTime [min]	Type	Width [min]	Area mAU *s	Height [mAU]	Area %
1	8.047	BB	0.2428	5318.38379	336.24030	85.8639
2	11.873	VP	0.2786	144.97568	7.88538	2.3406
3	22.399	BP	0.7065	174.13576	3.47730	2.8114
4	32.130	BB	1.0614	556.47229	7.72771	8.9841

Supplementary Figure 56. HPLC spectra for compound **3ca**

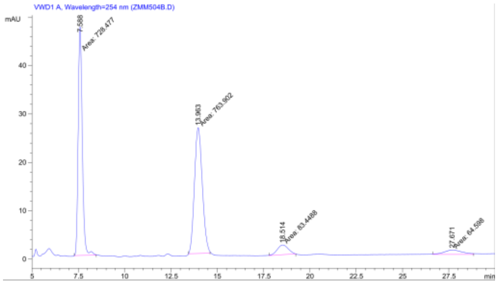

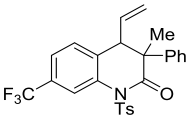

***rac*-3da**

Peak #	RetTime [min]	Type	Width [min]	Area mAU *s	Height [mAU]	Area %
1	7.588	MM	0.2569	728.47650	47.26527	44.4078
2	13.963	MM	0.4895	763.90155	26.01132	46.5673
3	18.514	MM	0.6927	83.44875	2.00768	5.0870
4	27.671	MM	1.1610	64.59799	9.27333e-1	3.9379

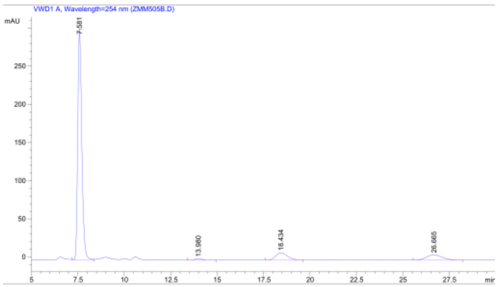

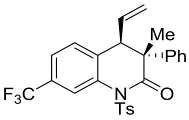

3da

Peak #	RetTime [min]	Type	Width [min]	Area mAU *s	Height [mAU]	Area %
1	7.581	VV	0.2348	4625.12305	303.18365	84.0022
2	13.980	PP	0.4137	47.21975	1.52463	0.8576
3	18.434	BB	0.6590	420.43088	9.50075	7.6359
4	26.665	BB	0.7646	413.17889	6.79603	7.5042

Supplementary Figure 57. HPLC spectra for compound **3da**

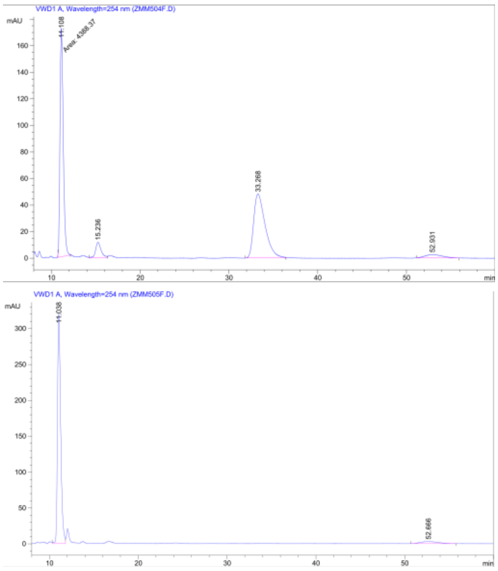

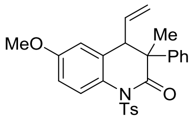

***rac*-3ea**

Peak #	RetTime [min]	Type	Width [min]	Area mAU *s	Height [mAU]	Area %
1	11.108	MM	0.4250	4388.36719	172.08061	45.7480
2	15.236	VB	0.6441	501.62622	11.60527	5.2294
3	33.268	PB	1.3712	4338.40674	48.16424	45.2271
4	52.931	BB	1.6282	364.08331	2.62756	3.7955

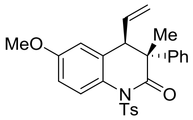

3ea

Peak #	RetTime [min]	Type	Width [min]	Area mAU *s	Height [mAU]	Area %
1	11.038	VV	0.3816	7995.24609	320.12686	95.1867
2	52.666	BB	1.6289	404.29721	2.93770	4.8133

Supplementary Figure 58. HPLC spectra for compound **3ea**

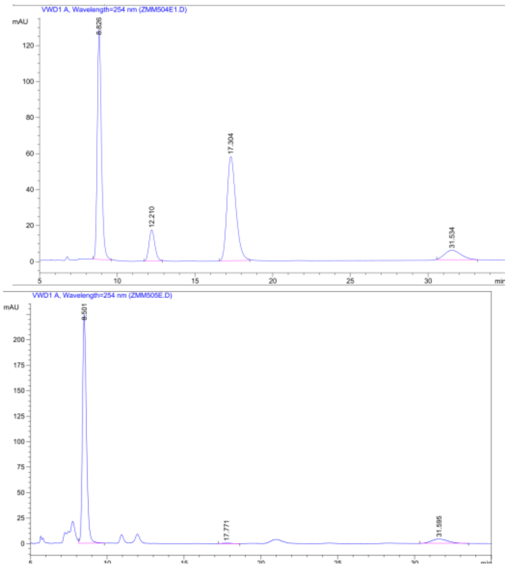

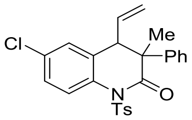

***rac*-3fa**

Peak #	RetTime [min]	Type	Width [min]	Area mAU *s	Height [mAU]	Area %
1	8.826	BB	0.2825	2362.42651	127.85316	42.8047
2	12.210	BB	0.4077	454.47931	17.34034	8.2347
3	17.304	BB	0.6164	2308.03979	57.91314	41.8193
4	31.534	BB	1.0408	394.13620	5.29144	7.1413

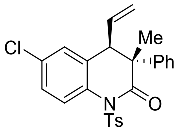

3fa

Peak #	RetTime [min]	Type	Width [min]	Area mAU *s	Height [mAU]	Area %
1	8.501	VP	0.2712	3978.37256	224.02928	91.7451
2	17.771	BP	0.4568	24.65322	6.63431e-1	0.5685
3	31.595	BB	1.0097	333.30667	4.35325	7.6864

Supplementary Figure 59. HPLC spectra for compound **3ea**

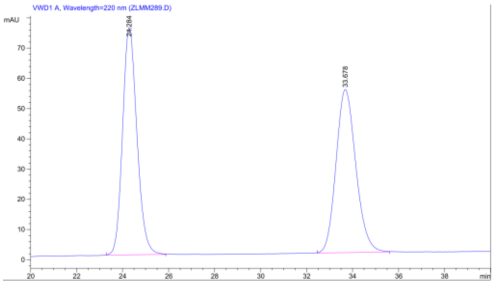

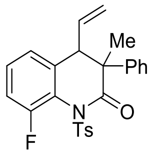

rac-**3ga**

Peak #	RetTime [min]	Type	Width [min]	Area mAU *s	Height [mAU]	Area %
1	24.284	BB	0.6669	3268.51733	75.25383	50.2925
2	33.678	BB	0.9315	3230.49194	53.95710	49.7075

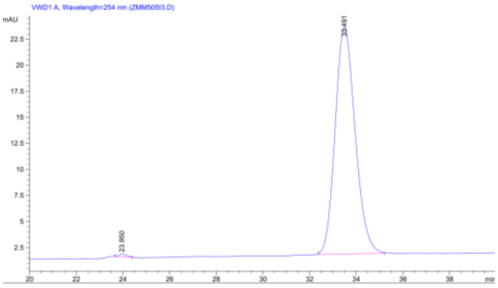

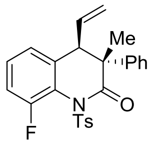

3ga

Peak #	RetTime [min]	Type	Width [min]	Area mAU *s	Height [mAU]	Area %
1	23.950	BB	0.3387	7.67444	2.76225e-1	0.5871
2	33.491	BB	0.9231	1299.46375	21.78459	99.4129

Supplementary Figure 60. HPLC spectra for compound **3ga**

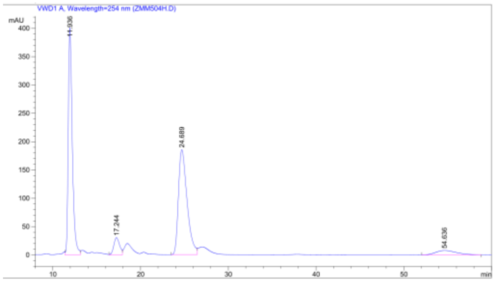

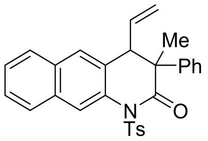

rac-**3ha**

Peak #	RetTime [min]	Type	Width [min]	Area mAU *s	Height [mAU]	Area %
1	11.936	VV	0.4843	1.28382e4	400.89816	45.3830
2	17.244	VV	0.7007	1392.86072	30.22412	4.9237
3	24.689	BV	1.0503	1.27226e4	185.57683	44.9741
4	54.636	BB	1.9988	1335.00452	7.86087	4.7192

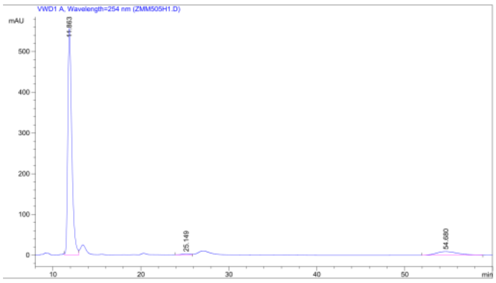

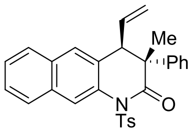

3ha

Peak #	RetTime [min]	Type	Width [min]	Area mAU *s	Height [mAU]	Area %
1	11.863	VV	0.4810	1.75333e4	556.66711	91.2276
2	25.149	BV	0.8203	170.96066	2.53954	0.8895
3	54.680	BB	2.0188	1515.02881	8.90974	7.8829

Supplementary Figure 61. HPLC spectra for compound **3ha**

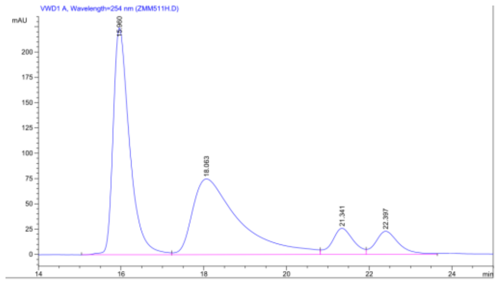

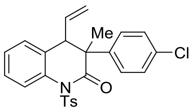

***rac*-3ab**

Peak #	RetTime [min]	Type	Width [min]	Area mAU *s	Height [mAU]	Area %
1	15.960	VV	0.4204	6170.30908	224.01115	44.4307
2	18.063	VV	1.1341	5896.81299	74.88830	42.4613
3	21.341	VV	0.5362	920.61987	25.94805	6.6291
4	22.397	VV	0.5847	899.74976	22.99176	6.4788

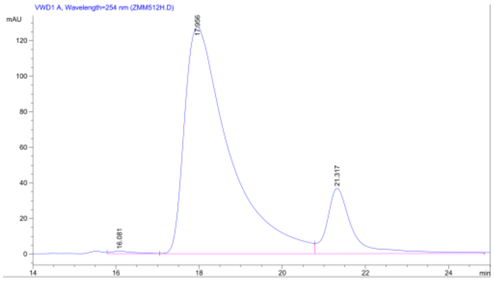

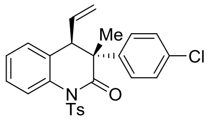

3ab

Peak #	RetTime [min]	Type	Width [min]	Area mAU *s	Height [mAU]	Area %
1	16.081	VV	0.5310	57.25276	1.50574	0.5076
2	17.956	VV	1.0864	9525.02148	127.59405	84.4458
3	21.317	VB	0.6240	1581.67493	36.77786	14.0226

Supplementary Figure 62. HPLC spectra for compound **3ab**

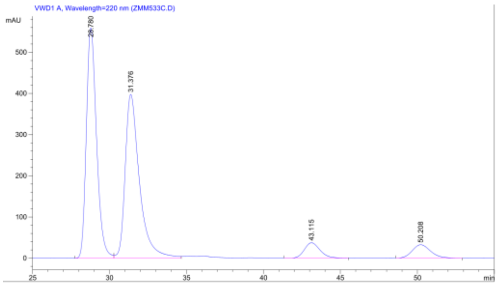

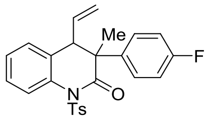

***rac*-3ac**

Peak #	RetTime [min]	Type	Width [min]	Area mAU *s	Height [mAU]	Area %
1	28.780	BV	0.7105	2.56864e4	559.31635	45.4085
2	31.376	VV	0.9598	2.55814e4	397.85489	45.2229
3	43.115	VB	1.0932	2628.51367	37.15559	4.6467
4	50.208	BB	1.2784	2671.03540	32.32900	4.7219

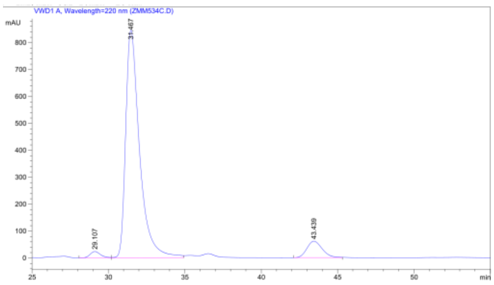

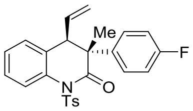

3ac

Peak #	RetTime [min]	Type	Width [min]	Area mAU *s	Height [mAU]	Area %
1	29.107	VV	0.7299	1146.68091	23.84840	1.9491
2	31.467	VB	0.9457	5.33263e4	845.26385	90.6418
3	43.439	BB	1.0931	4358.92920	61.62622	7.4091

Supplementary Figure 63. HPLC spectra for compound **3ac**

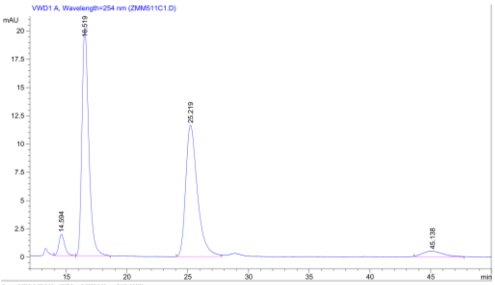

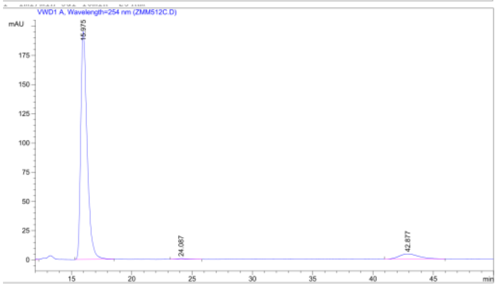

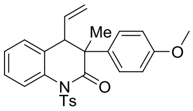

***rac*-3ad**

Peak #	RetTime [min]	Type	Width [min]	Area mAU *s	Height [mAU]	Area %
1	14.594	VV	0.5278	64.46522	1.90981	3.8055
2	16.519	VB	0.5968	789.90771	20.16554	46.6292
3	25.219	BB	1.0096	780.51923	11.63795	46.0750
4	45.138	BB	1.4264	59.12638	4.99920e-1	3.4903

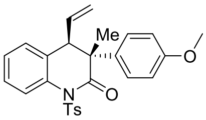

3ad

Peak #	RetTime [min]	Type	Width [min]	Area mAU *s	Height [mAU]	Area %
1	15.975	BB	0.5651	7207.80273	195.05762	93.0700
2	24.087	BB	0.8826	42.23787	6.33608e-1	0.5454
3	42.877	BB	1.6684	494.45731	4.37351	6.3846

Supplementary Figure 64. HPLC spectra for compound **3ad**

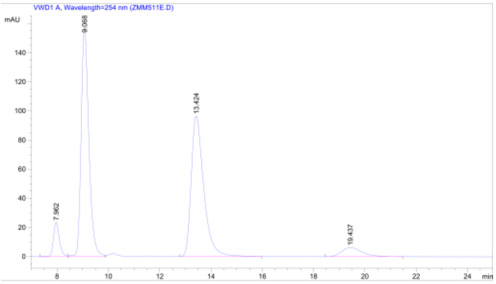

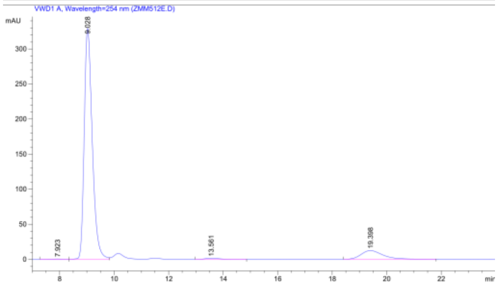

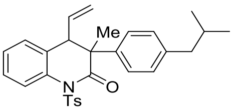

***rac*-3ae**

Peak #	RetTime [min]	Type	Width [min]	Area mAU *s	Height [mAU]	Area %
1	7.962	BV	0.2497	388.48419	23.67733	5.3152
2	9.068	VV	0.3190	3281.20874	157.33272	44.8935
3	13.424	BB	0.5192	3284.08789	96.53359	44.9329
4	19.437	BB	0.8522	355.09689	6.33283	4.8584

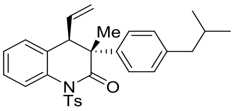

3ae

Peak #	RetTime [min]	Type	Width [min]	Area mAU *s	Height [mAU]	Area %
1	7.923	VV	0.5539	23.56730	5.75009e-1	0.3146
2	9.028	VV	0.3146	6727.27734	328.61105	89.7891
3	13.561	BB	0.5899	53.36420	1.31520	0.7123
4	19.398	BB	0.8536	688.09668	12.35536	9.1840

Supplementary Figure 65. HPLC spectra for compound **3ae**

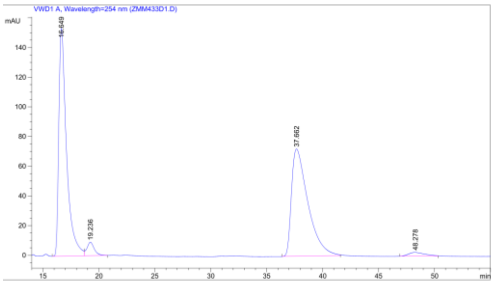

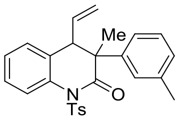

rac-**3af**

Peak #	RetTime [min]	Type	Width [min]	Area mAU *s	Height [mAU]	Area %
1	16.649	VV	0.7073	7350.55908	153.47284	47.7139
2	19.236	VP	0.6775	419.48761	9.10114	2.7230
3	37.662	PB	1.5143	7365.80029	72.32408	47.8128
4	48.278	BB	1.2498	269.65363	2.61466	1.7504

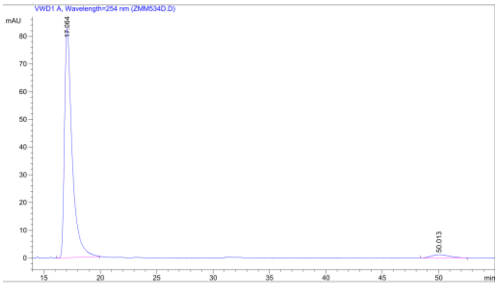

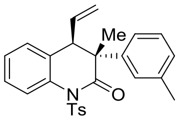

3af

Peak #	RetTime [min]	Type	Width [min]	Area mAU *s	Height [mAU]	Area %
1	17.064	VB	0.6948	3921.42432	82.85799	96.5794
2	50.013	BB	1.3431	138.88678	1.22231	3.4206

Supplementary Figure 66. HPLC spectra for compound **3af**

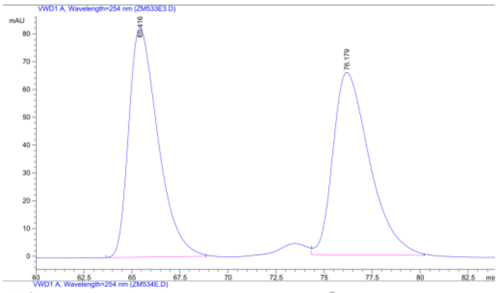

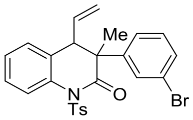

rac-**3ag**

Peak #	RetTime [min]	Type	Width [min]	Area mAU *s	Height [mAU]	Area %
1	65.416	PB	1.5823	8700.77539	82.33353	49.9939
2	76.179	VB	2.0170	8702.91016	65.70599	50.0061

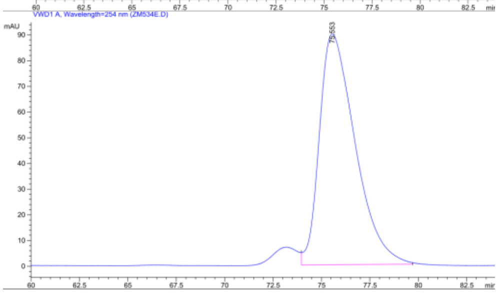

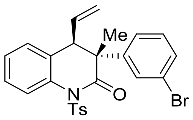

3ag

Peak #	RetTime [min]	Type	Width [min]	Area mAU *s	Height [mAU]	Area %
1	75.553	VB	1.8811	1.15236e4	89.82242	100.0000

Supplementary Figure 67. HPLC spectra for compound **3ag**

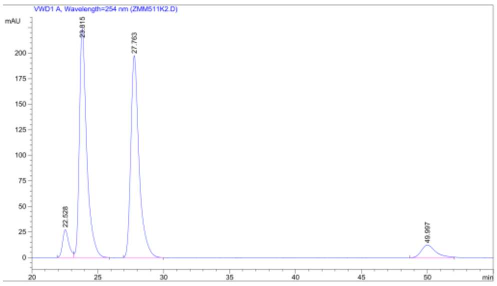

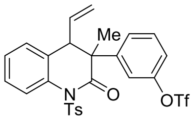

***rac*-3ah**

Peak #	RetTime [min]	Type	Width [min]	Area mAU *s	Height [mAU]	Area %
1	22.528	BV	0.5059	916.57990	27.45938	4.8028
2	23.815	VB	0.5718	8634.72949	224.13577	45.2451
3	27.763	BP	0.6519	8580.56152	197.70473	44.9613
4	49.997	BB	1.1577	952.45154	12.40644	4.9908

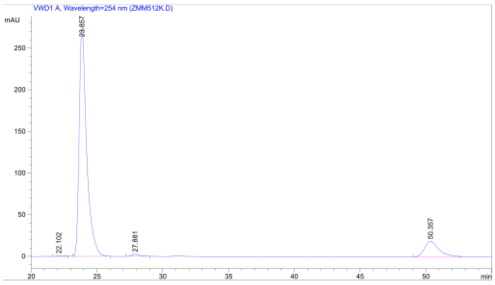

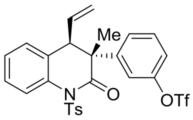

3ah

Peak #	RetTime [min]	Type	Width [min]	Area mAU *s	Height [mAU]	Area %
1	22.101	PB	0.5567	50.10389	1.23768	0.4043
2	23.856	BB	0.5833	1.07893e4	274.77734	87.0694
3	27.882	BB	0.6237	118.78265	2.76370	0.9586
4	50.357	BB	1.1453	1433.43738	18.68890	11.5677

Supplementary Figure 68. HPLC spectra for compound **3ah**

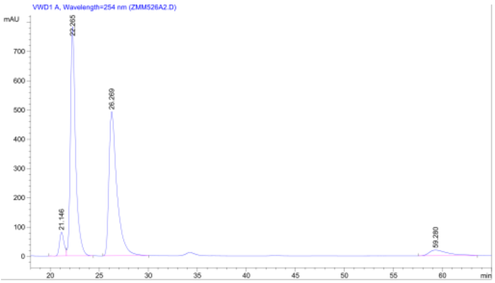

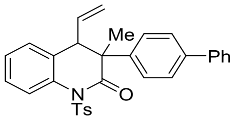

***rac*-3ai**

Peak #	RetTime [min]	Type	Width [min]	Area mAU *s	Height [mAU]	Area %
1	21.146	BV	0.4940	2639.69263	81.58481	4.2555
2	22.265	VB	0.5505	2.87464e4	783.20276	46.3429
3	26.269	BB	0.8358	2.79239e4	492.81750	45.0169
4	59.280	BB	1.9741	2719.84692	19.74302	4.3847

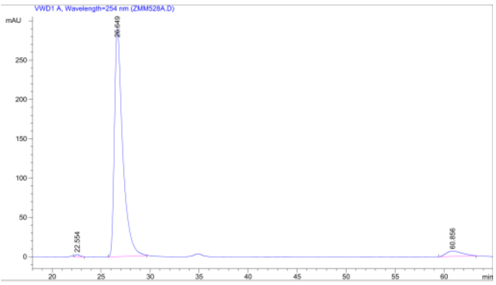

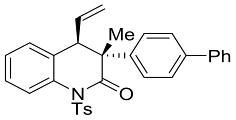

3ai

Peak #	RetTime [min]	Type	Width [min]	Area mAU *s	Height [mAU]	Area %
1	22.554	BB	0.4822	101.84451	2.94392	0.5656
2	26.649	BB	0.8626	1.70745e4	290.83722	94.8246
3	60.856	BB	1.4055	830.05676	6.99150	4.6098

Supplementary Figure 69. HPLC spectra for compound **3ai**

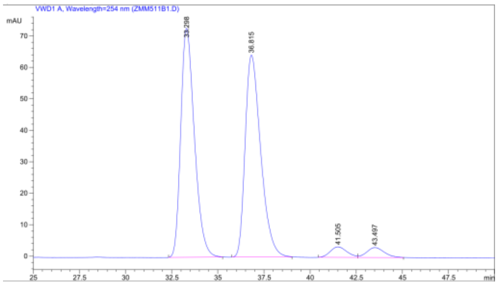

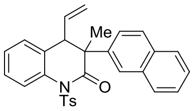

***rac*-3aj**

Peak #	RetTime [min]	Type	Width [min]	Area mAU *s	Height [mAU]	Area %
1	33.298	BB	0.7909	3755.62793	72.82802	47.2929
2	36.815	BB	0.8985	3759.90063	64.22576	47.3467
3	41.505	BV	0.8768	212.68481	3.38016	2.6782
4	43.497	VB	0.8835	213.00058	3.19116	2.6822

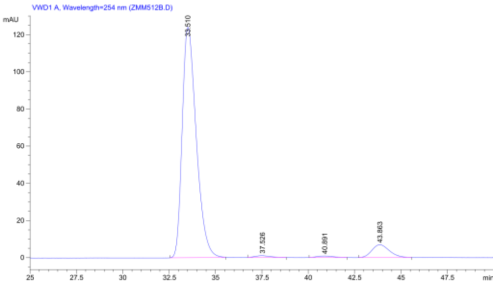

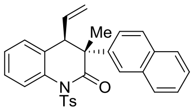

3aj

Peak #	RetTime [min]	Type	Width [min]	Area mAU *s	Height [mAU]	Area %
1	33.510	BB	0.8112	6519.43262	124.04102	92.0568
2	37.526	BB	0.6939	56.23063	9.74065e-1	0.7940
3	40.891	BB	0.7515	52.85619	8.46834e-1	0.7463
4	43.863	BB	0.9609	453.44974	6.90821	6.4029

Supplementary Figure 70. HPLC spectra for compound **3aj**

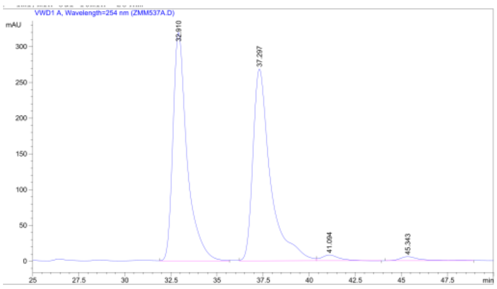

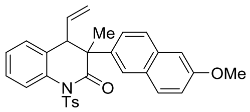

***rac*-3ak**

Peak #	RetTime [min]	Type	Width [min]	Area mAU *s	Height [mAU]	Area %
1	32.910	BB	0.7867	1.70879e4	319.68967	47.7071
2	37.297	BV	0.9699	1.77534e4	268.33832	49.5651
3	41.094	VB	0.9798	543.79852	8.11517	1.5182
4	45.343	BB	1.1004	433.26846	5.67482	1.2096

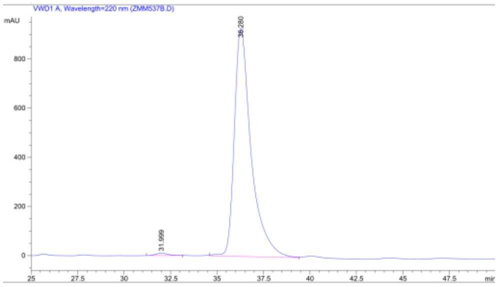

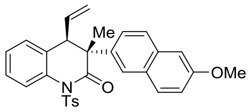

3ak

Peak #	RetTime [min]	Type	Width [min]	Area mAU *s	Height [mAU]	Area %
1	31.999	BB	0.7777	503.30444	9.73860	0.8552
2	36.280	BB	0.9310	5.83500e4	928.74622	99.1448

Supplementary Figure 71. HPLC spectra for compound **3ak**

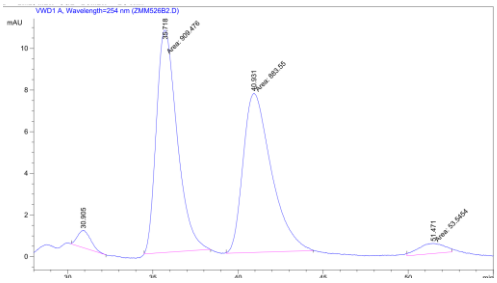

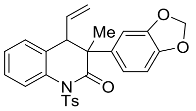

rac-3al

Peak #	RetTime [min]	Type	Width [min]	Area mAU *s	Height [mAU]	Area %
1	30.905	BP	0.6535	45.33552	8.27740e-1	2.3963
2	35.718	MM	1.4193	909.47620	10.68007	48.0719
3	40.931	MM	1.9251	883.55048	7.64957	46.7016
4	51.471	MM	1.8150	53.54545	4.91694e-1	2.8302

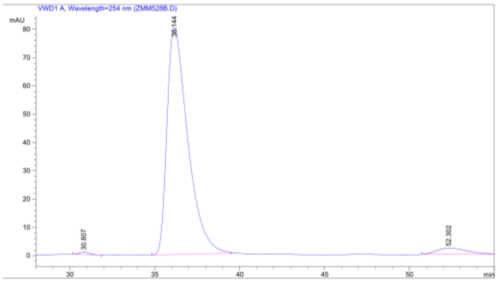

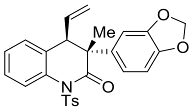

3al

Peak #	RetTime [min]	Type	Width [min]	Area mAU *s	Height [mAU]	Area %
1	30.807	BP	0.5399	37.76793	8.88021e-1	0.5275
2	36.144	BB	1.2766	6833.99951	80.20164	95.4561
3	52.302	BB	1.5275	287.54547	2.24755	4.0164

Supplementary Figure 72. HPLC spectra for compound **3al**

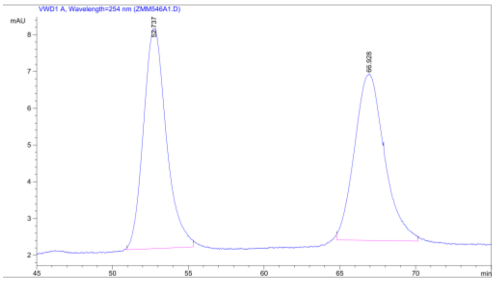

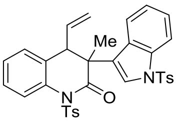

rac-3am

Peak #	RetTime [min]	Type	Width [min]	Area mAU *s	Height [mAU]	Area %
1	52.737	BB	1.5225	626.97443	5.99749	50.2350
2	66.928	BB	1.8825	621.10834	4.52054	49.7650

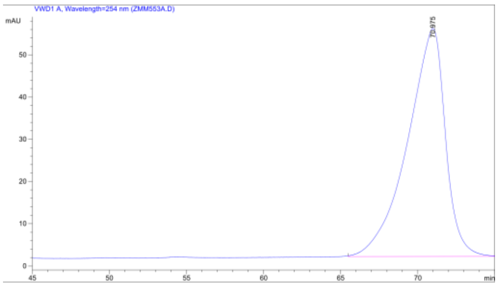

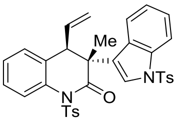

3am

Peak #	RetTime [min]	Type	Width [min]	Area mAU *s	Height [mAU]	Area %
1	70.975	BB	2.4170	9221.62891	54.01432	100.0000

Supplementary Figure 73. HPLC spectra for compound **3am**

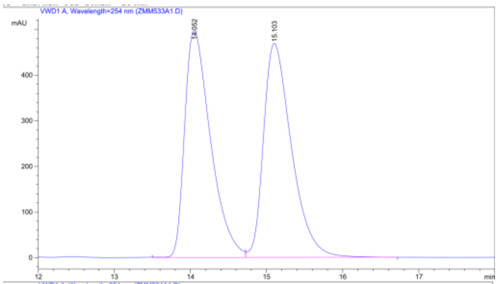

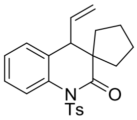

rac-3an

Peak #	RetTime [min]	Type	Width [min]	Area mAU *s	Height [mAU]	Area %
1	14.052	BV	0.3627	1.17563e4	498.10931	49.4351
2	15.103	VB	0.3926	1.20249e4	468.49344	50.5649

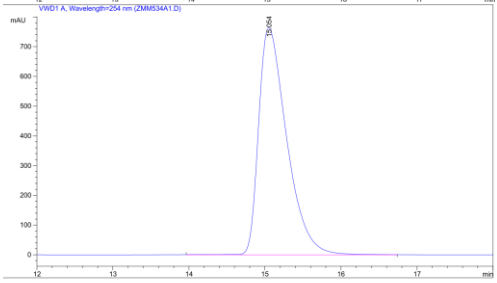

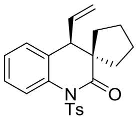

3an

Peak #	RetTime [min]	Type	Width [min]	Area mAU *s	Height [mAU]	Area %
1	15.054	BB	0.3923	1.95728e4	763.30823	100.0000

Supplementary Figure 74. HPLC spectra for compound **3an**

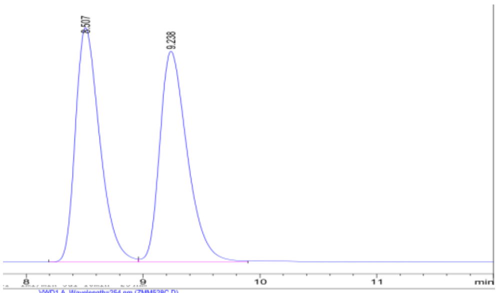

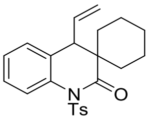

rac-3ao

Peak #	RetTime [min]	Type	Width [min]	Area mAU *s	Height [mAU]	Area %
1	8.507	BV	0.2247	9222.37891	635.17957	49.6429
2	9.238	VV	0.2523	9355.06738	571.00031	50.3571

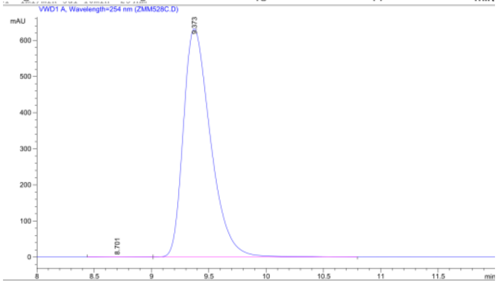

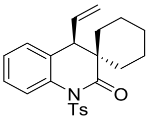

3ao

Peak #	RetTime [min]	Type	Width [min]	Area mAU *s	Height [mAU]	Area %
1	8.701	BV	0.2495	12.61167	7.14838e-1	0.1207
2	9.373	VB	0.2536	1.04330e4	632.34930	99.8793

Supplementary Figure 75. HPLC spectra for compound **3ao**

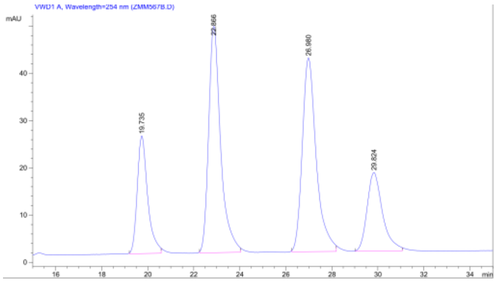

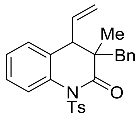

rac-**3ap**

Peak #	RetTime [min]	Type	Width [min]	Area mAU *s	Height [mAU]	Area %
1	19.735	BB	0.4786	786.55231	24.94046	15.6401
2	22.866	BB	0.5443	1735.25818	47.97415	34.5046
3	26.980	BB	0.6371	1728.73071	41.03865	34.3748
4	29.824	BB	0.7077	778.52856	16.67560	15.4806

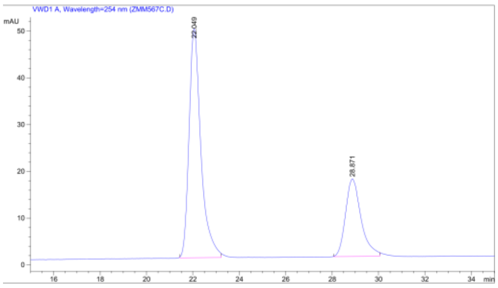

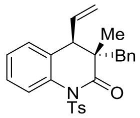

3ap

Peak #	RetTime [min]	Type	Width [min]	Area mAU *s	Height [mAU]	Area %
1	22.049	BB	0.5357	1733.95032	48.93972	69.2079
2	28.871	BB	0.7050	771.47186	16.60938	30.7921

Supplementary Figure 76. HPLC spectra for compound **3ap**

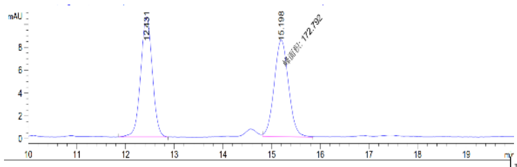

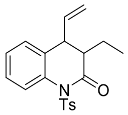

rac-**3ar**

Peak #	RetTime [min]	Type	Width [min]	Area mAU *s	Height [mAU]	Area %
1	12.431	BB	0.2585	175.11368	10.50439	50.3337
2	15.198	MM	0.3405	172.79182	8.45742	49.6663

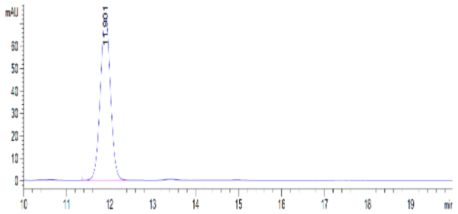

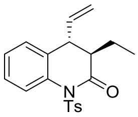

3ar

Peak #	RetTime [min]	Type	Width [min]	Area mAU *s	Height [mAU]	Area %
1	11.901	BB	0.2506	1204.91125	74.52222	100.0000

Supplementary Figure 77. HPLC spectra for compound **3ar**

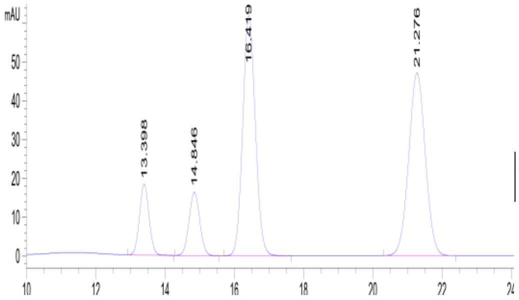

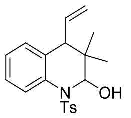

rac-**4aq**

Peak #	RetTime [min]	Type	Width [min]	Area mAU *s	Height [mAU]	Area %
1	13.398	BB	0.2992	348.63205	18.22172	9.0881
2	14.846	BB	0.3327	350.81424	16.32808	9.1450
3	16.419	BB	0.3923	1559.77979	61.83917	40.6602
4	21.276	BB	0.5250	1576.91211	47.05121	41.1068

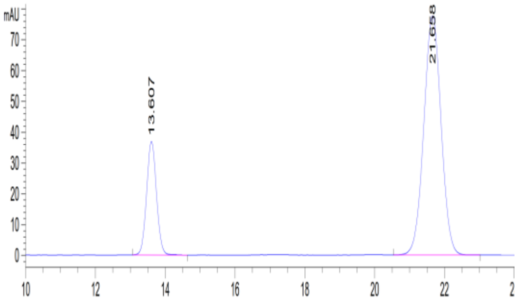

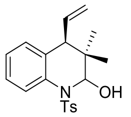

4aq

Peak #	RetTime [min]	Type	Width [min]	Area mAU *s	Height [mAU]	Area %
1	13.607	BB	0.3032	722.62897	36.79018	21.3205
2	21.658	BB	0.5314	2666.72925	77.51731	78.6795

Supplementary Figure 78. HPLC spectra for compound **4aq**

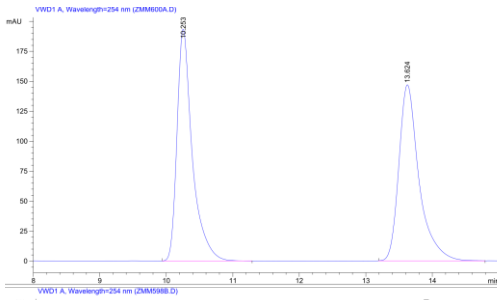

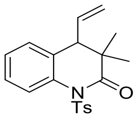

rac-**5a**

Peak #	RetTime [min]	Type	Width [min]	Area mAU *s	Height [mAU]	Area %
1	10.253	BB	0.2384	3082.97778	193.40364	49.9631
2	13.624	BB	0.3151	3087.52856	146.87784	50.0369

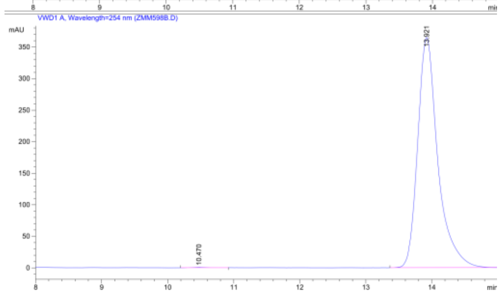

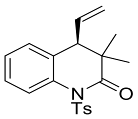

5a

Peak #	RetTime [min]	Type	Width [min]	Area mAU *s	Height [mAU]	Area %
1	10.470	PB	0.2465	7.49187	4.43704e-1	0.0967
2	13.921	BB	0.3201	7736.37695	365.04187	99.9033

Supplementary Figure 79. HPLC spectra for compound **5a**

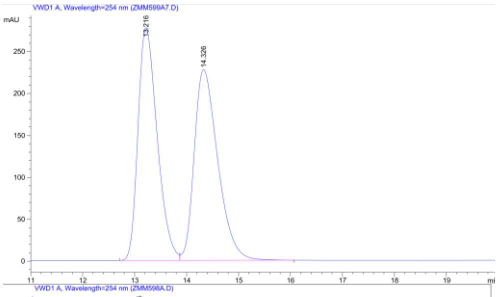

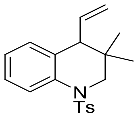

***rac*-5b**

Peak #	RetTime [min]	Type	Width [min]	Area mAU *s	Height [mAU]	Area %
1	13.215	BV	0.3954	7129.92432	277.92987	49.8219
2	14.326	VB	0.4845	7180.90479	227.64189	50.1781

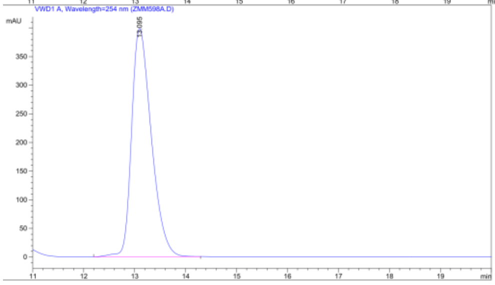

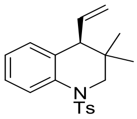

5b

Peak #	RetTime [min]	Type	Width [min]	Area mAU *s	Height [mAU]	Area %
1	13.095	BB	0.4132	1.07336e4	398.51672	100.0000

Supplementary Figure 80. HPLC spectra for compound **5b**

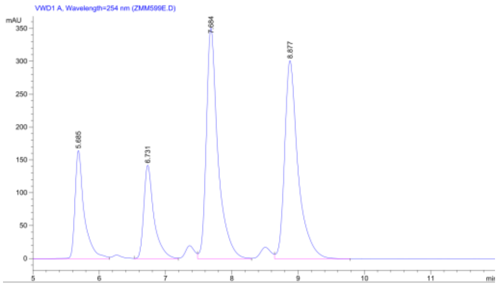

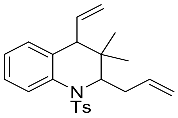

***rac*-5c**

Peak #	RetTime [min]	Type	Width [min]	Area mAU *s	Height [mAU]	Area %
1	5.685	VV	0.1400	1606.25146	164.66388	13.7887
2	6.731	VV	0.1569	1522.48975	142.25235	13.0696
3	7.684	VV	0.1801	4280.40283	351.13730	36.7446
4	8.877	VB	0.2078	4239.92383	301.47614	36.3971

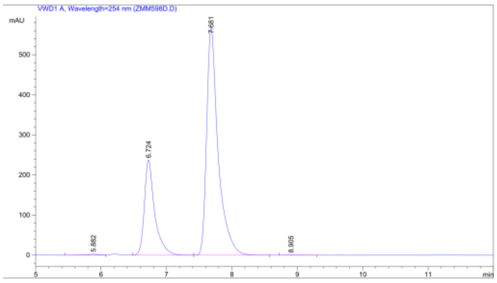

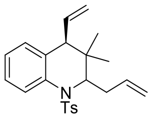

5c

Peak #	RetTime [min]	Type	Width [min]	Area mAU *s	Height [mAU]	Area %
1	5.882	BV	0.2276	40.01675	2.38781	0.4162
2	6.724	VV	0.1605	2616.56714	237.53053	27.2147
3	7.681	VB	0.1807	6941.95898	566.93958	72.2027
4	8.905	BB	0.2478	16.00248	9.41506e-1	0.1664

Supplementary Figure 81. HPLC spectra for compound **5c**

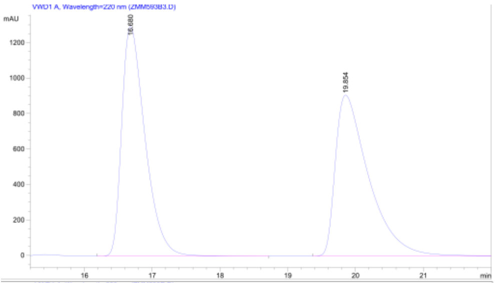

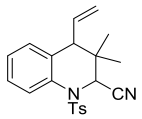

rac-**5d**

Peak #	RetTime [min]	Type	Width [min]	Area mAU *s	Height [mAU]	Area %
1	16.680	VB	0.3666	3.09860e4	1294.52051	49.8183
2	19.854	BB	0.5118	3.12121e4	907.65759	50.1817

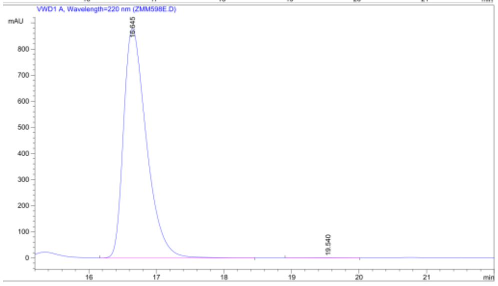

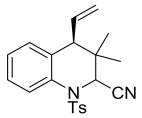

5d

Peak #	RetTime [min]	Type	Width [min]	Area mAU *s	Height [mAU]	Area %
1	16.645	VB	0.3617	2.06365e4	877.47894	99.7497
2	19.540	BV	0.5910	51.79107	1.17263	0.2503

Supplementary Figure 82. HPLC spectra for compound **5d**

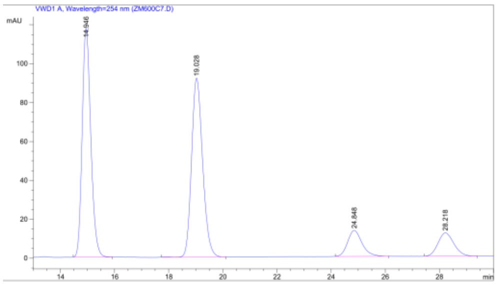

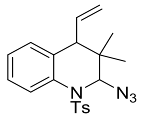

rac-**5e**

Peak #	RetTime [min]	Type	Width [min]	Area mAU *s	Height [mAU]	Area %
1	14.946	BB	0.3393	2601.82959	117.75124	41.8920
2	19.028	BP	0.4387	2610.95508	92.03516	42.0390
3	24.848	BB	0.5703	501.27478	13.40358	8.0710
4	28.218	BB	0.6384	496.73978	12.04428	7.9980

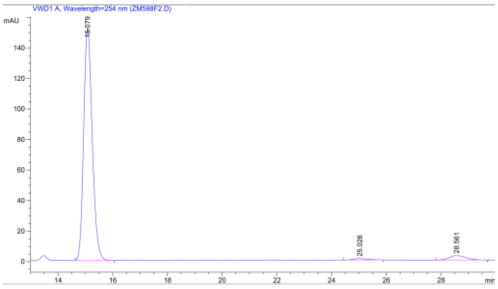

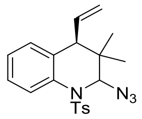

5e

Peak #	RetTime [min]	Type	Width [min]	Area mAU *s	Height [mAU]	Area %
1	15.079	BB	0.3350	3302.98511	152.01910	95.1703
2	25.026	BB	0.5411	40.42824	1.00726	1.1649
3	28.561	BP	0.6230	127.19212	2.92974	3.6648

Supplementary Figure 83. HPLC spectra for compound **5e**

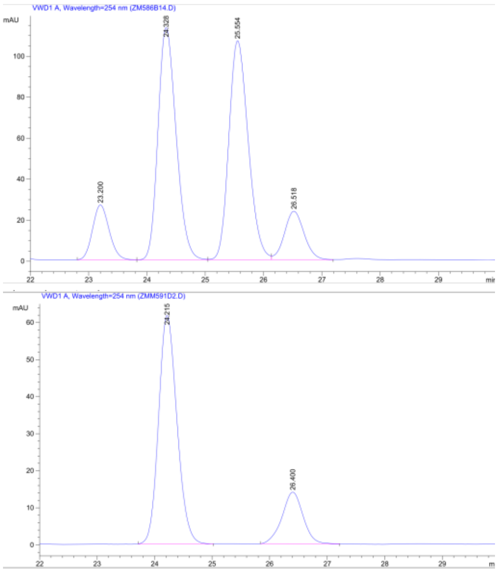

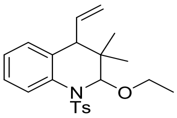

***rac*-5f**

Peak #	RetTime [min]	Type	Width [min]	Area mAU *s	Height [mAU]	Area %
1	23.200	BV	0.3118	536.37640	26.83874	8.8993
2	24.328	VV	0.3408	2459.36182	113.22040	40.8047
3	25.554	VV	0.3572	2471.79688	106.86559	41.0110
4	26.518	VV	0.3626	559.62347	23.71740	9.2850

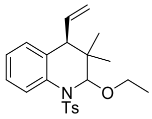

5f

Peak #	RetTime [min]	Type	Width [min]	Area mAU *s	Height [mAU]	Area %
1	24.215	BB	0.3470	1372.85242	61.68467	79.2318
2	26.400	BB	0.3936	359.85178	13.97311	20.7682

Supplementary Figure 84. HPLC spectra for compound **5f**

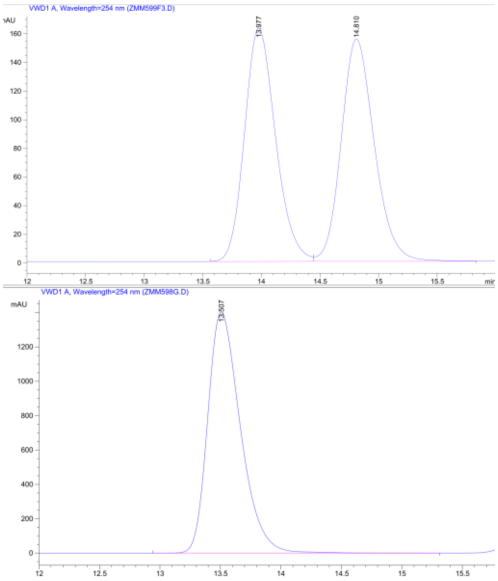

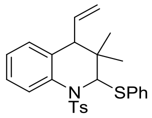

***rac*-5g**

Peak #	RetTime [min]	Type	Width [min]	Area mAU *s	Height [mAU]	Area %
1	13.977	BV	0.2850	3003.45996	162.85712	49.5913
2	14.810	VB	0.3052	3052.96704	155.23151	50.4087

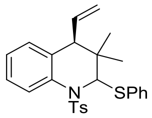

5g

Peak #	RetTime [min]	Type	Width [min]	Area mAU *s	Height [mAU]	Area %
1	13.507	BB	0.2897	2.64038e4	1401.69983	100.0000

Supplementary Figure 85. HPLC spectra for compound **5g**

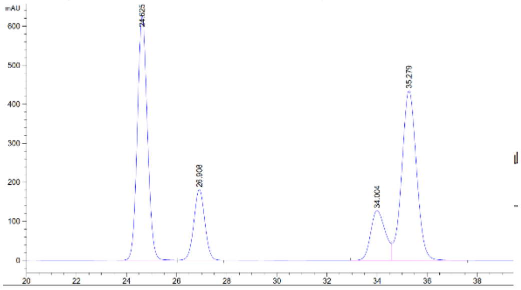

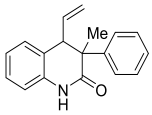

***rac*-8aa**

Peak #	RetTime [min]	Type	Width [min]	Area mAU *s	Height [mAU]	Area %
1	24.625	BB	0.4298	1.73530e4	625.15576	37.9236
2	26.908	BB	0.4668	5477.89014	181.15808	11.9715
3	34.004	BV	0.6189	5181.75098	127.84082	11.3243
4	35.279	VB	0.6279	1.77452e4	435.05685	38.7806

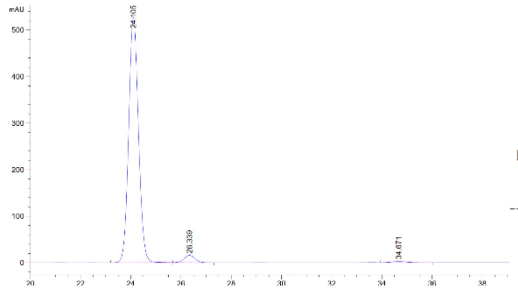

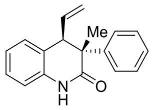

8aa

Peak #	RetTime [min]	Type	Width [min]	Area mAU *s	Height [mAU]	Area %
1	24.105	BB	0.4193	1.42373e4	526.86731	96.2075
2	26.339	BB	0.4456	459.74808	15.61179	3.1067
3	34.671	BB	0.5015	101.48010	2.47928	0.6857

Supplementary Figure 86. HPLC spectra for compound **8aa**

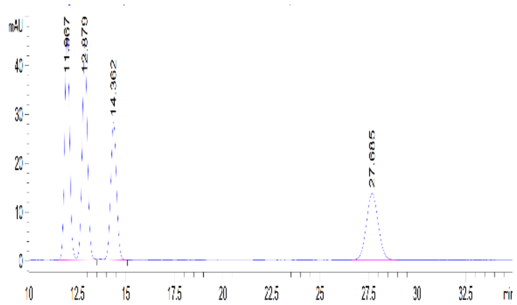

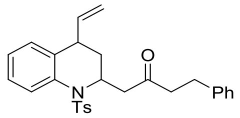

rac-**7a**

Peak #	RetTime [min]	Type	Width [min]	Area mAU *s	Height [mAU]	Area %
1	11.967	BV	0.2699	831.89996	47.58797	29.4992
2	12.879	VB	0.2955	828.43164	43.64028	29.3762
3	14.362	BB	0.3229	584.14746	28.06067	20.7139
4	27.685	BB	0.6373	575.60205	13.61421	20.4108

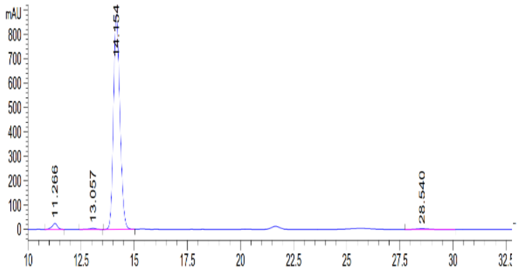

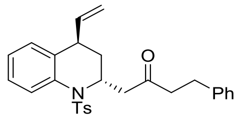

(*R*,*R*)-**7a**

Peak #	RetTime [min]	Type	Width [min]	Area mAU *s	Height [mAU]	Area %
1	11.266	BB	0.2582	428.97964	24.75271	2.2224
2	13.057	BB	0.3206	91.89863	4.11345	0.4761
3	14.154	BB	0.3268	1.86606e4	881.98322	96.6726
4	28.540	BB	0.5033	121.39806	2.90617	0.6289

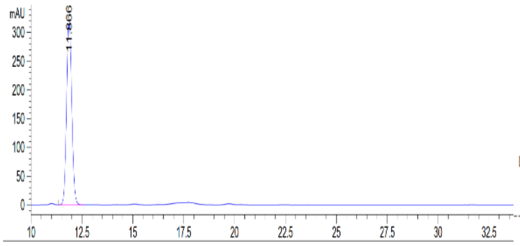

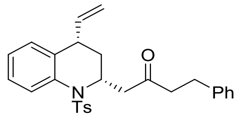

(*R*,*S*)-**7a**

Peak #	RetTime [min]	Type	Width [min]	Area mAU *s	Height [mAU]	Area %
1	11.866	BB	0.2699	5794.46191	331.54282	100.0000

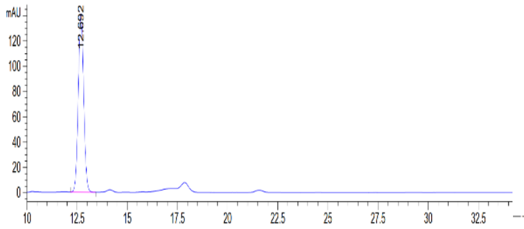

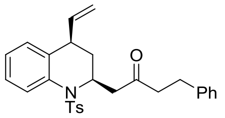

(*S*,*R*)-**7a**

Peak #	RetTime [min]	Type	Width [min]	Area mAU *s	Height [mAU]	Area %
1	12.692	BB	0.2920	2659.98413	141.03714	100.0000

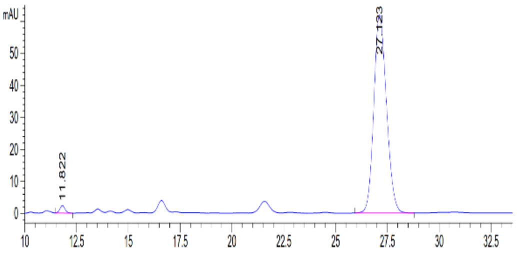

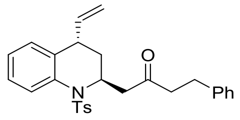

(*S*,*S*)-**7a**

Peak #	RetTime [min]	Type	Width [min]	Area mAU *s	Height [mAU]	Area %
1	11.822	BB	0.2591	38.63869	2.26405	1.4484
2	27.123	BB	0.6502	2629.06348	61.81440	98.5516

Supplementary Figure 87. HPLC spectra for compound **7a**

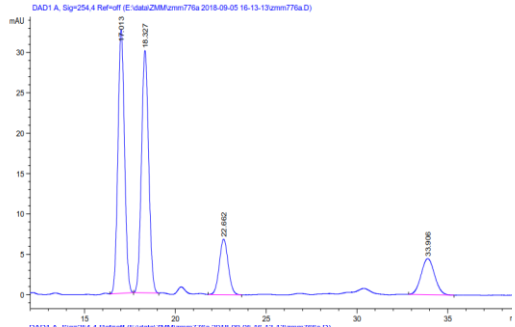

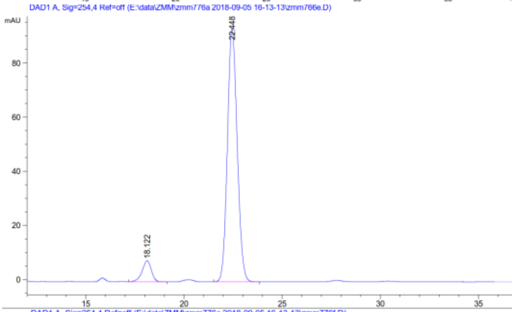

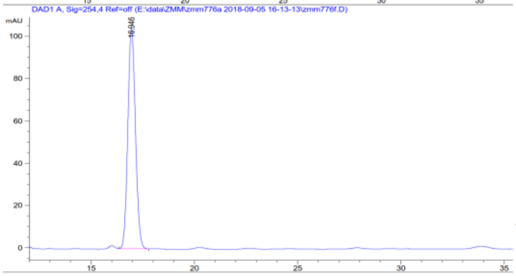

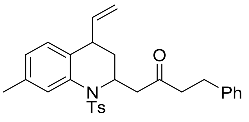

***rac*-7b**

Peak #	RetTime [min]	Type	Width [min]	Area mAU *s	Height [mAU]	Area %
1	17.013	BB	0.3950	825.88342	32.65815	38.9832
2	18.327	BB	0.4305	829.32721	30.00300	39.1458
3	22.662	BB	0.5123	233.55504	6.87587	11.0242
4	33.906	BB	0.7166	229.79640	4.47835	10.8468

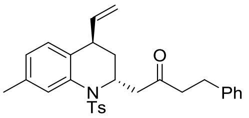

(*R*,*R*)-7b

Peak #	RetTime [min]	Type	Width [min]	Area mAU *s	Height [mAU]	Area %
1	18.122	BB	0.4813	253.00102	7.78280	7.2283
2	22.448	BB	0.5414	3247.14404	93.44183	92.7717

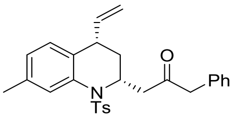

(*R*,*S*)-7b

Peak #	RetTime [min]	Type	Width [min]	Area mAU *s	Height [mAU]	Area %
1	16.946	BB	0.3982	2665.19897	104.28143	100.0000

Supplementary Figure 88. HPLC spectra for compound **7b**

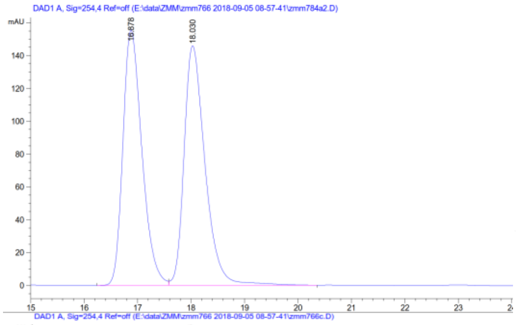

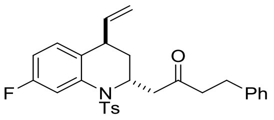

***rac*-7c**

Peak #	RetTime [min]	Type	Width [min]	Area mAU *s	Height [mAU]	Area %
1	16.878	BV	0.3810	3851.10913	155.46748	49.2178
2	18.030	VB	0.4139	3973.52075	145.83990	50.7822

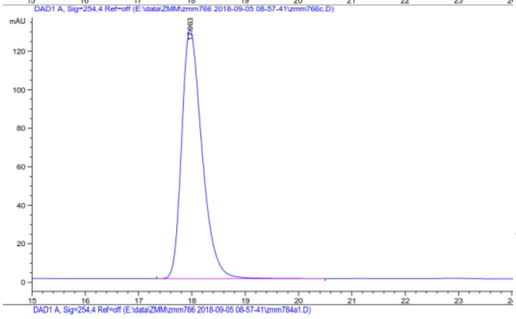

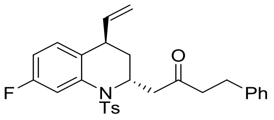

(*R*,*R*)-7c

Peak #	RetTime [min]	Type	Width [min]	Area mAU *s	Height [mAU]	Area %
1	17.963	BB	0.4054	3425.39380	129.15962	100.0000

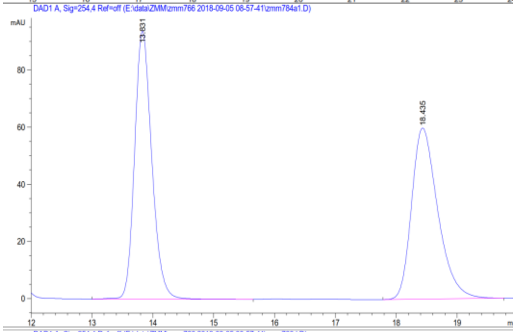

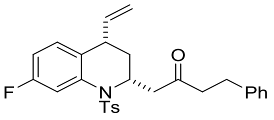

***rac*-7c**

Peak #	RetTime [min]	Type	Width [min]	Area mAU *s	Height [mAU]	Area %
1	13.831	BB	0.3013	1842.63892	93.73814	50.6959
2	18.435	BB	0.4573	1792.04944	59.85801	49.3041

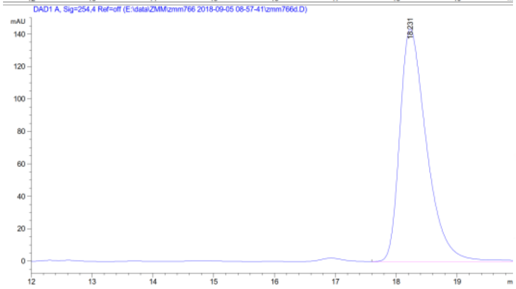

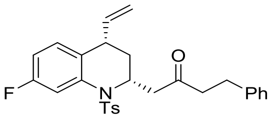

(*R*,*S*)-7c

Peak #	RetTime [min]	Type	Width [min]	Area mAU *s	Height [mAU]	Area %
1	18.231	BB	0.4653	4387.04004	143.24936	100.0000

Supplementary Figure 89. HPLC spectra for compound **7c**

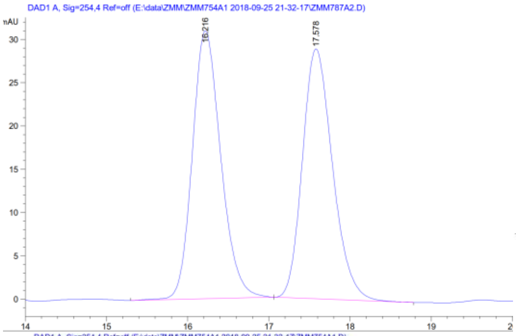

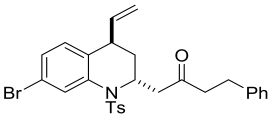

***rac*-7d**

Peak #	RetTime [min]	Type	Width [min]	Area mAU *s	Height [mAU]	Area %
1	16.216	BB	0.3721	753.71283	30.95550	50.2978
2	17.578	BB	0.3970	744.78845	28.86328	49.7022

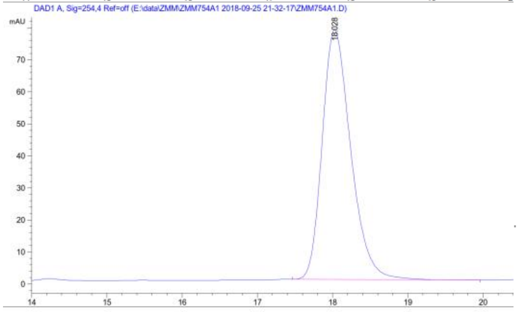

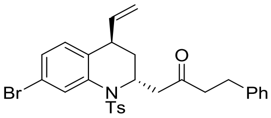

(*R*,*R*)-7d

Peak #	RetTime [min]	Type	Width [min]	Area mAU *s	Height [mAU]	Area %
1	18.028	BB	0.4057	2056.46777	77.97136	100.0000

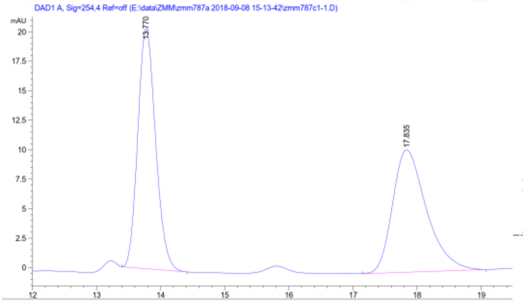

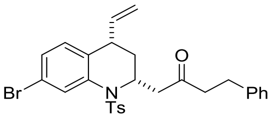

***rac*-7d**

Peak #	RetTime [min]	Type	Width [min]	Area mAU *s	Height [mAU]	Area %
1	13.770	BB	0.2994	393.40182	20.35808	50.8114
2	17.835	BB	0.5560	380.83817	10.38135	49.1886

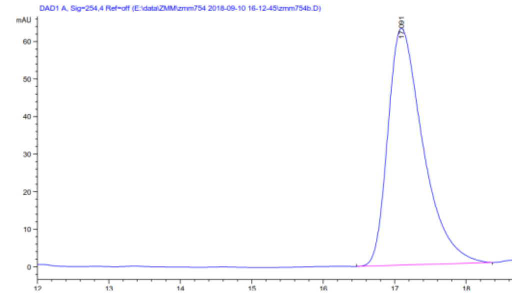

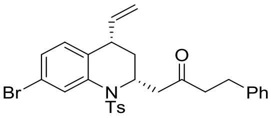

(*R*,*S*)-7d

Peak #	RetTime [min]	Type	Width [min]	Area mAU *s	Height [mAU]	Area %
1	17.091	BB	0.5138	2152.51563	63.12106	100.0000

Supplementary Figure 90. HPLC spectra for compound **7d**

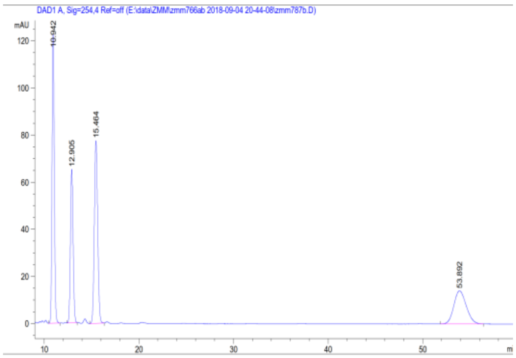

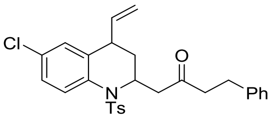

***rac*-7e**

Peak #	RetTime [min]	Type	Width [min]	Area mAU *s	Height [mAU]	Area %
1	10.942	BB	0.2571	2025.96509	122.40081	31.3644
2	12.905	BB	0.3037	1269.66418	65.05460	19.6559
3	15.464	BB	0.3775	1883.42310	77.50758	29.1576
4	53.892	BB	1.2968	1280.39685	14.05521	19.8221

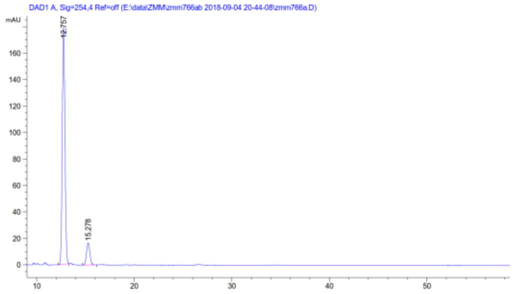

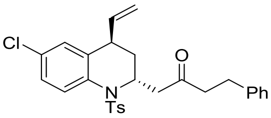

(*R*,*R*)-7e

Peak #	RetTime [min]	Type	Width [min]	Area mAU *s	Height [mAU]	Area %
1	12.757	BB	0.2982	3435.60303	178.75697	89.6637
2	15.278	BB	0.3719	396.05270	16.62525	10.3363

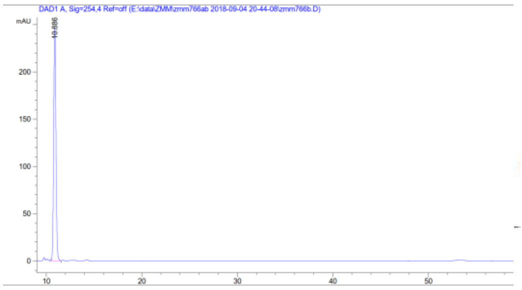

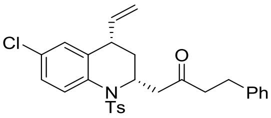

(*R*,*S*)-7e

Peak #	RetTime [min]	Type	Width [min]	Area mAU *s	Height [mAU]	Area %
1	10.886	BB	0.2554	4027.69019	245.46875	100.0000

Supplementary Figure 91. HPLC spectra for compound **7e**

Supplementary Table 1. The effect of acids for the cycloaddition of aldhyde **2a**[a]

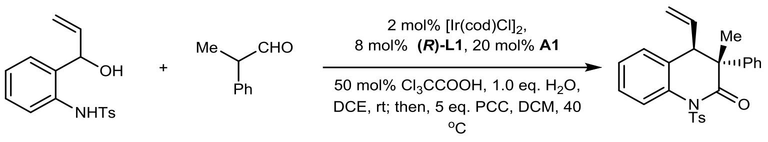

Entry	Acid	Yield(%)[b]	Dr[c]	Ee(%)[c]
1	CCl_3CO_2H	64	>19:1	99
2	CF_3CO_2H	63	>19:1	82
3	$TsOH{\cdot}H_2O$	trace	-	-
4	$(PhO)_2PO_2H$	58	>19:1	98
5	$PhCO_2H$	N.R.	-	N.R.
6	*p*-NO_2-$PhCO_2H$	N.R.	-	-
7	CH_3CO_2H	trace	-	-

[a]Standard conditions: **1a** (0.25 mmol), **2a** (0.5 mmol), $[Ir(cod)Cl]_2$ (2 mol%), chiral ligand **(*R*)-L1** (8 mol%), amine **A1** (20 mol%), acid (50 mol%) and H_2O (1.0 eq). in 1,2-dichloroethane (0.5 mL) for 48 h at room temperature; then, PCC (5.0 eq), silica gel (100 mg) and CH_2Cl_2 (5mL) were added and the mixture was stirred at 40 °C for 10 h. [b]Isolated yield. [c]Determined by chiral HPLC analysis.

Supplementary Table 2. The effect of amine catalysts for the cycloaddition of aldehyde **2a**[a]

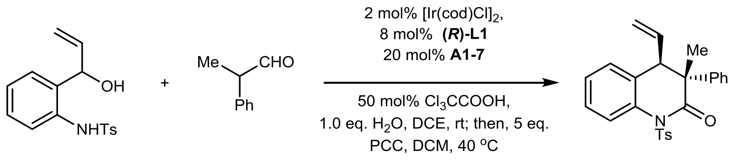

1a **2a** **3aa**

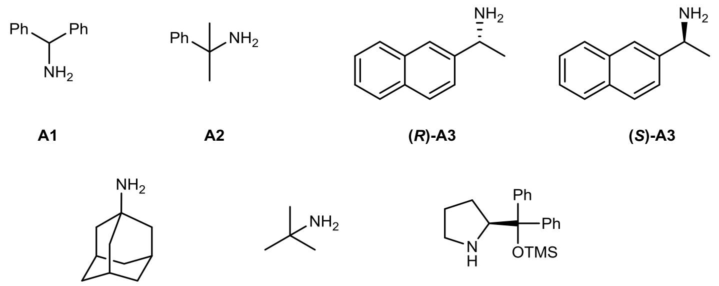

A1 **A2** **(*R*)-A3** **(*S*)-A3**

A5 **A6** **A7**

Entry	Amine	Yield(%)[b]	Dr[c]	Ee(%)[c]
1	**A1**	64	>19:1	99
2	**A2**	22	>19:1	95
3	**(*R*)-A3**	62	>19:1	93
4	**(*S*)-A3**	26	>19:1	94
5[d]	**(*R*)-A3**	28	>19:1	-95
6[d]	**(*S*)-A3**	61	>19:1	-94
7	**A5**	27	>19:1	99
8	**A6**	22	>19:1	98
9	**A7**	trace	-	-

[a]Standard conditions: **1a** (0.25 mmol), **2a** (0.5 mmol), $[Ir(cod)Cl]_2$ (2 mol%), chiral ligand (8 mol%), amine (20 mol%), trichloroacetic acid (50 mol%.) and H_2O (1.0 eq). in 1,2-dichloroethane (0.5 mL) for 48 h at room temperature; then, PCC (5.0 eq), silica gel (100 mg) and CH_2Cl_2 (5mL) were added and the mixture was stirred at 40 °C for 10 h. [b]Isolated yield. [c]Determined by chiral HPLC analysis. [d]**(*S*)-L1** was used instead of **(*R*)-L1.**

Supplementary Table 3. The effect of amine catalysts and ligands for the cycloaddition of ketone **6a**[a]

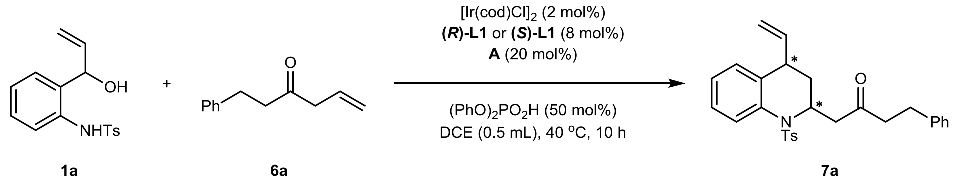

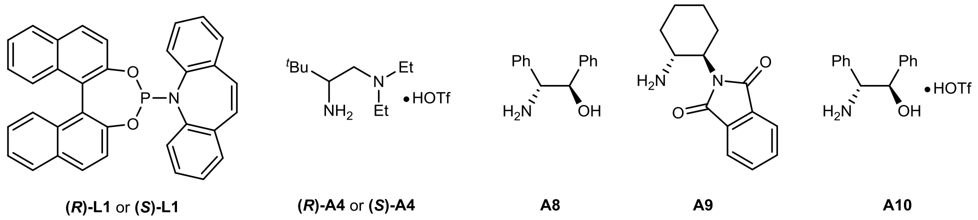

Entry	Ligand	Amine	Yield(%)[b]	Dr[c]	Ee(%)[c]
1	**(*R*)-L1**	**(*S*)-A4**	29	>20:1	>99
2	**(*S*)-L1**	**(*S*)-A4**	34	>1:20	>99
3	**(*R*)-L1**	**A8**	64	1.7:1	99
4	**(*S*)-L1**	**A8**	41	4.6:1	>99
5	**(*R*)-L1**	**A9**	N.R.	-	-
6	**(*S*)-L1**	**A9**	N.R.	-	-
7	**(*R*)-L1**	**A10**	43	4:1	>99
8	**(*S*)-L1**	**A10**	45	3:1	>99
9[d]	**(*R*)-L1**	**(*S*)-A4**	84	>20:1	>99
10[d]	**(*S*)-L1**	**(S)-A4**	81	>1:20	>99
11[d]	**(*R*)-L1**	**(*R*)-A4**	80	>1:20	>99
12[d]	**(*S*)-L1**	**(*R*)A4**	86	>20:1	>99

[a]Standard conditions: **1a** (0.25 mmol), **6a** (0.5 mmol), $[Ir(cod)Cl]_2$ (2 mol%), L (8 mol%), amine **A** (20 mol%), $(PhO)_2PO_2H$ (0.5 eq.) in 1,2-dichloroethane (0.5 mL) for 10 h at 40 °C. [b]Isolated yield. [c]Determined by chiral HPLC analysis. [d]Without $(PhO)_2PO_2H$, **6a** (1.25 mmol), DCE (0.3 mL) at 50 °C for 48 h.

Supplementary Methods

General analytical information

^{1}H NMR spectra were recorded on 400 or 600 MHz spectrophotometers. Chemical shifts (δ) are reported in ppm from the resonance of tetramethyl silane as the internal standard (TMS: 0.00 ppm). Data are reported as follows: chemical shift, multiplicity (s = singlet, d = doublet, t = triplet, q = quartet, m = multiplet), coupling constants (Hz) and integration. All NMR spectra were recorded on a Bruker spectrometer at 400 MHz (^{1}H NMR), 100 MHz (^{13}C NMR), 376 MHz (^{19}F NMR). HRMS was recorded on Waters GCT Premier ES*I*-TOF. Enantiomeric ratio (ee) values were determined by chiral HPLC with chiral OD-H, AD-H, AZ-H, IC-H, IE-H, OX-H, OJ-H, IF-H columns with hexane and *i*-PrOH as solvents. Optical rotations were measured with a polarimeter. All air- and moisture-sensitive reactions were performed under an atmosphere of Ar in fire dried glassware. The manipulations for iridium-catalyzed reactions were carried out with standard Schlenk techniques under argon. Flash column chromatography was performed using 200-300 mesh silica gel.

Preparation of Substrates and Ligands

Substrates **1** were prepared through one step from known chemcials[1] as shown below:

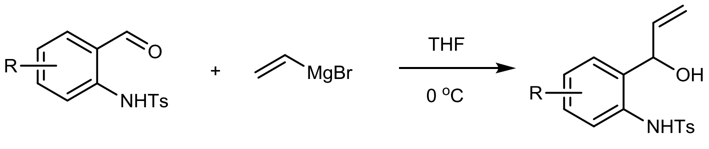

Supplementary procedure A: Vinylmagnesium bromide **B** (1.0 M THF solution, 12.5 mmol) was slowly added to the solution of aldehyde **A** (5 mmol) in dry THF (15 mL) at 0 °C under a nitrogen atmosphere. Then, the mixture was stirred at 0 °C for 30 minutes (monitored by TLC) and quenched with saturated *aq.* $NaHCO_3$ (20 mL). The aqueous layer was extracted with EtOAc and the organic phase was dried over Na_2SO_4, filtered and concentrated *in vacuo.* The crude residue was purified by column chromatography on silica gel to give the title compound **1**.

N-(2-(1-Hydroxyallyl)phenyl)-4-methylbenzenesulfonamide(1a)

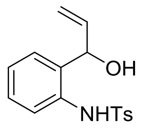

White solid, 91% yield, M.P: 63-65 °C. **^{1}H NMR** (400 MHz, $CDCl_3$) *δ* (ppm) 8.14 (s, 1H), 7.67 (d, *J* = 8.2 Hz, 2H), 7.46 (d, *J* = 8.1 Hz, 1H), 7.22 (d, *J* = 8.2 Hz, 3H), 7.10 – 7.03 (m, 2H), 5.90 – 5.82 (m, 1H), 5.23 – 5.03 (m, 3H), 2.50 (s, 1H), 2.38 (s, 3H). **^{13}C NMR** (100 MHz, $CDCl_3$) *δ* (ppm) 143.7, 137.6, 136.5, 135.6, 131.8, 129.5, 128.7, 128.2, 127.2, 124.6, 121.8, 116.1, 74.1, 21.4. HRMS (ESI): m/z [M + Na]$^+$ calcd for $C_{16}H_{17}NO_3S$: 326.0821; found: 326.0823.

N-(2-(1-Hydroxyallyl)-5-methylphenyl)-4-methylbenzenesulfonamide(1b)

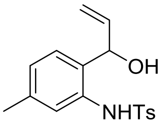

White solid, 72% yield, M.P: 105-107 °C. **^{1}H NMR** (400 MHz, $CDCl_3$) δ (ppm) 8.11 (s, 1H), 7.65 (d, J = 7.8 Hz, 2H), 7.27 (s, 1H), 7.21 (d, J = 7.9 Hz, 2H), 6.95 (d, J = 7.7 Hz, 1H), 6.84 (d, J = 7.8 Hz, 1H), 5.86-5.78 (m, 1H), 5.13 (dd, J = 27.7, 13.8 Hz, 2H), 5.02 (d, J = 5.4 Hz, 1H), 2.65 (s, 1H), 2.37 (s, 3H), 2.27 (s, 3H). **^{13}C NMR** (100 MHz, $CDCl_3$) δ (ppm) 143.7, 139.0, 137.9, 136.8, 135.7, 129.5, 128.9, 128.1, 127.3, 125.5, 122.7, 116.1, 74.0, 21.5, 21.2. HRMS (ESI): m/z [M + Na]$^+$ calcd for $C_{17}H_{19}NO_3S$: 340.0978; found: 340.0977.

N-(5-Fluoro-2-(1-hydroxyallyl)phenyl)-4-methylbenzenesulfonamide(1c)

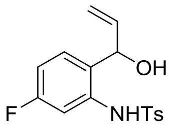

White solid, 89% yield, M.P: 78-79 °C. **^{1}H NMR** (400 MHz, $CDCl_3$) δ (ppm) 8.39 (s, 1H), 7.71 (d, J = 8.2 Hz, 2H), 7.30 – 7.24 (m, 3H), 7.02 (dd, J = 8.5, 6.2 Hz, 1H), 6.73 – 6.68 (m, 1H), 5.89-5.81 (m, 1H), 5.22 – 5.08 (m, 3H), 2.49 – 2.48 (m, 1H), 2.39 (s, 3H). **^{13}C NMR** (100 MHz, $CDCl_3$) δ (ppm) 162.6 (d, J = 246.5 Hz), 144.1, 137.6 (d, J = 10.8 Hz), 137.4, 136.4, 129.6, 129.6, 127.2, 126.3 (d, J = 3.3 Hz), 116.5, 110.7 (d, J = 21.5 Hz), 108.3 (d, J = 26.4 Hz). 74.2, 21.5. **^{19}F NMR** (376 MHz, $CDCl_3$) δ (ppm) -111.5. HRMS (ESI): m/z [M + Na]$^+$ calcd for $C_{16}H_{16}NO_3FS$: 344.0727; found: 344.0723.

N-(2-(1-Hydroxyallyl)-5-(trifluoromethyl)phenyl)-4-methylbenzenesulfonamide(1d)

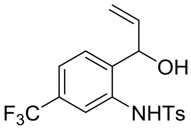

White solid, 65% yield, M.P: 66-68 °C. **^{1}H NMR** (400 MHz, $CDCl_3$) δ (ppm) 8.43 (s, 1H), 7.74 (s, 1H), 7.68 (d, J = 8.1 Hz, 2H), 7.28 – 7.23 (m, 3H), 7.19 (d, J = 8.0 Hz, 1H), 5.88-5.80 (m, 1H), 5.25 – 5.16 (m, 3H), 2.81 (d, J = 3.2 Hz, 1H), 2.38 (s, 3H). **^{13}C NMR** (100 MHz, $CDCl_3$) δ (ppm) 144.3, 136.8, 136.6, 136.1, 134.4, 129.7, 128.8, 127.3, 123.5 (q, J = 270.9 Hz) 120.9 (q, J = 120.9 Hz), 118.5, 118.0, 117.1, 74.4, 21.5. **^{19}F NMR** (376 MHz, $CDCl_3$) δ (ppm) -62.9. HRMS (ESI): m/z [M + Na]$^+$ calcd for $C_{17}H_{16}NO_3F_3S$: 394.0695; found: 394.0696.

N-(2-(1-Hydroxyallyl)-4-methoxyphenyl)-4-methylbenzenesulfonamide(1e)

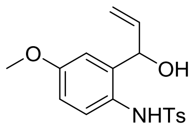

White solid, 75% yield, thick oil. **^{1}H NMR** (400 MHz, $CDCl_3$) δ (ppm) 7.61 – 7.58 (m, 2H), 7.34 (s, 1H), 7.23 (d, J = 7.9 Hz, 2H), 7.20 – 7.17 (m, 1H), 6.74 – 6.73 (m, 2H), 5.86 – 5.77 (m, 1H), 5.25 – 5.14 (m, 2H), 5.04 (s, 1H), 3.76 (s, 3H), 2.52 (d, J = 3.9 Hz, 1H), 2.40 (s, 3H). **^{13}C NMR** (100 MHz, $CDCl_3$) δ (ppm) 157.6, 143.7, 137.6, 136.6, 136.6, 129.5, 127.6, 127.3, 126.4, 116.3, 113.6, 113.6, 72.6, 55.4, 21.5. HRMS (ESI): m/z [M + Na]$^+$ calcd for $C_{17}H_{19}NO_4S$: 356.0927; found: 356.0926.

N-(4-Chloro-2-(1-hydroxyallyl)phenyl)-4-methylbenzenesulfonamide(1f)

White solid, 88% yield, M.P: 81-83 °C. **^{1}H NMR** (400 MHz, $CDCl_3$) δ (ppm) 8.19 (s, 1H), 7.64 – 7.61 (m, 2H), 7.38 – 7.35 (m, 1H), 7.23 (d, J = 7.9 Hz, 2H), 7.17 – 7.15 (m, 1H), 7.06 (s, 1H), 5.80 – 5.73 (m, 1H), 5.22 –

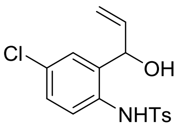

5.12 (m, 2H), 5.02 (d, *J* = 5.2 Hz, 1H), 3.01 (s, 1H), 2.38 (s, 3H). **^{13}C NMR** (100 MHz, $CDCl_3$) δ (ppm) 144.1, 136.8, 136.1, 134.2, 133.6, 130.1, 129.6, 128.7, 128.7, 128.2, 127.2, 123.4, 116.9, 73.6, 73.5, 21.5. HRMS (ESI): m/z $[M + Na]^+$ calcd for $C_{16}H_{16}NO_3SCl$: 360.0432; found: 360.0427.

***N*-(2-Fluoro-6-(1-hydroxyallyl)phenyl)-4-methylbenzenesulfonamide(1g)**

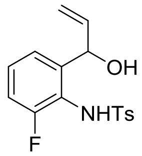

White solid, 82% yield, M.P: 112-114 °C. **^{1}H NMR** (400 MHz, $CDCl_3$) δ (ppm) 7.58 (d, *J* = 8.0 Hz, 2H), 7.30 – 7.24 (m, 4H), 6.88 – 6.83 (m, 1H), 6.51 (s, 1H), 6.09-6.01 (m, 1H), 5.79 (d, *J* = 4.5 Hz, 1H), 5.49 (d, *J* = 17.1 Hz, 1H), 5.29 (d, *J* = 10.5 Hz, 1H), 3.21 (d, *J* = 3.7 Hz, 1H), 2.43 (s, 3H). **^{13}C NMR** (100 MHz, $CDCl_3$) δ (ppm) 157.8 (d, *J* = 249.3 Hz), 143.8 (d, *J* = 70.7 Hz), 138.2, 135.9, 129.5, 129.1 (d, *J* = 8.4 Hz), 127.4, 124.5, 124.4, 121.6 (d, *J* = 13.6 Hz), 115.5, 115.0 (d, *J* = 20.8 Hz), 77.3, 77.0, 69.4, 69.4, 21.6. **^{19}F NMR** (376 MHz, CDC_{l3}) δ (ppm) -121.0. HRMS (ESI): m/z $[M + Na]^+$ calcd for $C_{16}H_{16}NO_3FS$: 344.0727; found: 344.0719.

***N*-(3-(1-Hydroxyallyl)naphthalen-2-yl)-4-methylbenzenesulfonamide(1h)**

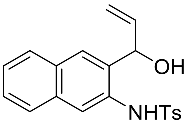

White solid, 72% yield, thick oil. **^{1}H NMR** (400 MHz, $CDCl_3$) δ (ppm) 8.43 (s, 1H), 7.90 (s, 1H), 7.72 – 7.66 (m, 4H), 7.50 (s, 1H), 7.43 – 7.37 (m, 2H), 7.13 (d, *J* = 7.9 Hz, 2H), 5.97-5.89 (m, 1H), 5.22 – 5.14 (m, 3H), 2.94 (s, 1H), 2.31 (s, 3H). **^{13}C NMR** (100 MHz, $CDCl_3$) δ (ppm) 143.8, 137.6, 136.7, 133.7, 133.3, 130.8, 130.2, 129.5, 127.7, 127.6, 127.4, 127.3, 126.8, 125.6, 118.8, 116.7, 74.6, 21.5. HRMS (ESI): m/z $[M + Na]^+$ calcd for $C_{20}H_{19}NO_3S$: 376.0978; found: 376.0976.

Substrate **2a, 2n, 2o** were commerially available, **2b-2j, 2l-2m, 2p** were prepared according to the reported procedures.[2-5] Chiral ligands **(*R*)-L1** and **(*S*)-L1** were synthesised according to the literatures.[6] Substrate **6** was prepared according to the reported procedure.[7] Substrate **2k** was prepared through two steps as shown below.

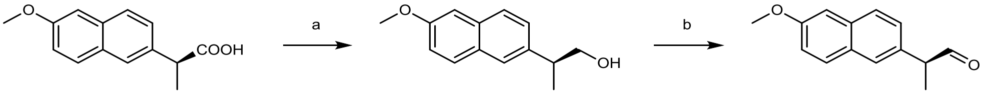

(a) $LiAlH_4$ (2.0 eq.), THF, 0 °C; (b) $PhI(OAc)_2$ (1.1 eq.), TEMPO (0.1 eq.), DCM, RT.

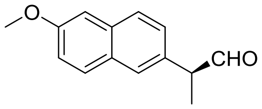

White solid, 65% yield, M.P: 70-71 °C. **^{1}H NMR** (400 MHz, $CDCl_3$) δ (ppm) 9.74 (t, *J* = 1.0 Hz, 1H), 7.73 (dd, *J* = 14.0, 8.7 Hz, 2H), 7.60 (s, 1H), 7.29 – 7.26 (m, 1H), 7.18 – 7.13 (m, 2H), 3.92 (s, 3H), 3.79 – 7.74 (m, 1H), 1.52 (d, *J* = 7.1 Hz, 3H). **^{13}C NMR** (100 MHz, $CDCl_3$) δ (ppm) 201.2, 157.8, 133.8, 132.6, 129.2, 129.1, 127.6, 127.0, 126.7, 119.3, 105.5, 55.3, 52.9, 14.6. HRMS (ESI): m/z $[M + Na]^+$ calcd for $C_{14}H_{14}O_2$: 237.0886; found: 237.0897.

General procedure for the one-pot cyclization/PCC oxidation reaction

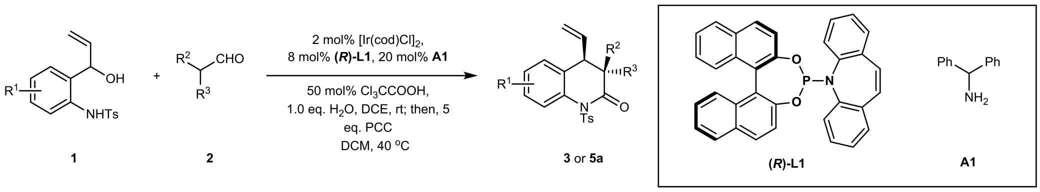

Supplementary procedure B: In a dried Schlenk tube under N_2, $[Ir(cod)Cl]_2$ (0.005 mmol, 3.35 mg) and chiral ligand **(*R*)-L1** (0.02 mmol, 10.15 mg) were mixed in 0.5 mL DCE and stirred at ambient temperature for 20 min under argon atmosphere. Then allylic alcohol **1** (0.25 mmol, 1.0 eq), aldehyde **2** (0.5 mmol, 2.0 eq), Cl_3CCOOH (0.125 mmol, 20.4 mg), amine **A1** (0.05 mmol, 9.2 mg, 8.7μL), 4.5 μL H_2O (1.0 eq.) were added to the mixture successively. The reaction mixture was stirred at ambient temperature until substrate **1** disappeared on TLC. Then the mixture was diluted with 5 mL DCM, and PCC (275 mg, 5.0 eq.) and 100 mg silica gel were added to the mixture. The mixture was stirred at 40 °C for 10 h and the final product was achieved by flash column chromatography.

General procedure for the one-pot cyclization/IBX oxidation reaction

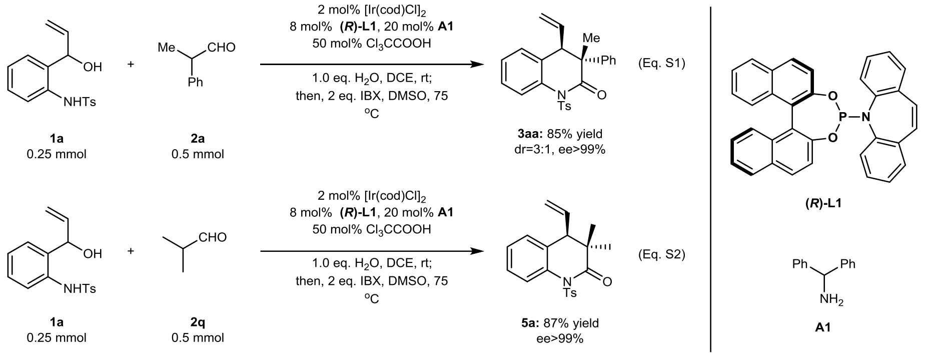

Supplementary procedure C: In a dried Schlenk tube under N_2, $[Ir(cod)Cl]_2$ (0.005 mmol, 3.35 mg) and chiral ligand **(*R*)-L1** (0.02 mmol, 10.15 mg) were mixed in 0.5 mL DCE and stirred at ambient temperature for 20 min under argon atmosphere. Then allylic alcohol **1** (0.25 mmol, 1.0 eq), aldehyde **2** (0.5 mmol, 2.0 eq), Cl_3CCOOH (0.125 mmol, 20.4 mg), amine **A1** (0.05 mmol, 9.2 mg, 8.7μL), 4.5 μL H_2O (1.0 eq.) were added to the mixture successively. The reaction mixture was stirred at ambient temperature until substrate **1** disappeared on TLC. After removing the solvent under reduced pressure, 4 mL DMSO and 2.0 eq IBX were added and the mixture was stirred at 75 °C for 12 h. Then water (10 mL) was added and the aqueous phase was extracted with ethyl acetate (4×5 mL). The combined organic layers were dried over Na_2SO_4, filtered and concentrated in vacuo. Finaly, the residue was purified by flash silica gel chromatography to get the title compound.

Supplementary procedure for the synthesis of 5b

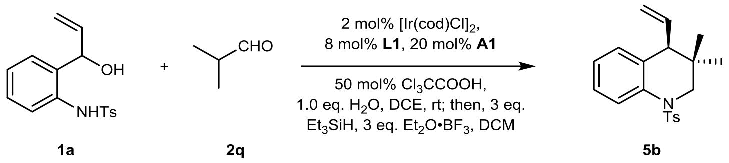

Supplementary procedure D: After the first step (as **Supplementary procedure B**), the mixture was diluted with 3 mL DCM under N_2, cooled to 0 °C in ice bath; then Et_3SiH (3.0 eq., 87.2 mg, 120μL) and $BF_3{\cdot}Et_2O$ (3.0 eq., 106.4 mg, 93 μL) were added successively. The mixture was stirred at 0 °C until the hemiaminal was disappeared determined by TLC. The residue was directly purified by flash silica gel chromatography to afford the title compound as a white solid.

Procedure for the synthesis of 5c

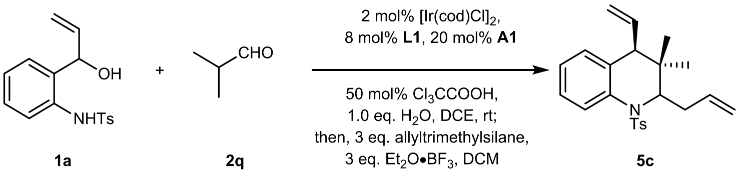

Supplementary procedure E: After the first step (as **Supplementary procedure B**), the mixture was diluted with 3 mL DCM under N_2, cooled to 0 °C in ice bath; then allyltrimethylsilane (3.0 eq. 85.7 mg, 119 μL) and $BF_3{\cdot}Et_2O$ (3.0 eq., 106.4 mg, 93 μL) were added successively. The mixture was stirred at 0 °C until the hemiaminal was disappeared determined by TLC. The residue was directly purified by flash silica gel chromatography to afford the title compound as a white solid.

Procedure for the synthesis of 5d

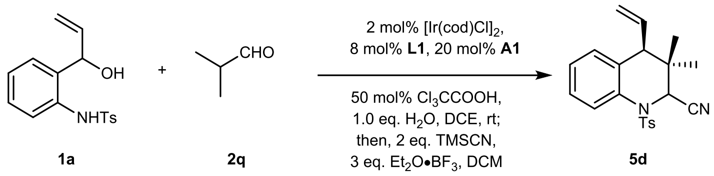

Supplementary procedure F: After the first step (as **Supplementary procedure B**), the mixture was diluted with 3 mL DCM under N_2, cooled to 0 °C in ice bath; then nucleophiles (2.0 eq., 50 mg, 63 μL) and $BF_3{\cdot}Et_2O$ (3.0 eq., 106.4 mg, 93 μL) were added successively. The mixture was stirred at 0 °C until the hemiaminal was disappeared determined by TLC. The residue was directly purified by flash chromatography to afford the title compound as a white solid.

Procedure for the synthesis of 5e

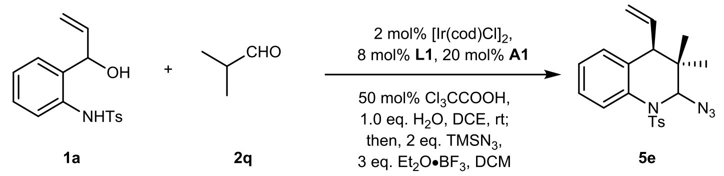

Supplementary procedure G: After the first step (as **Supplementary procedure B**), the mixture was diluted with 3 mL DCM under N_2, cooled to 0 °C in ice bath; then $TMSN_3$ (2.0 eq., 58 mg, 68 μL) and $BF_3{\cdot}Et_2O$ (3.0 eq., 106.4 mg, 93 μL) were added successively. The mixture was stirred at 0 °C until the hemiaminal was disappeared determined by TLC. The residue was directly purified by flash silica gel chromatography to afford the title compound as a white solid.

Procedure for the synthesis of 5f

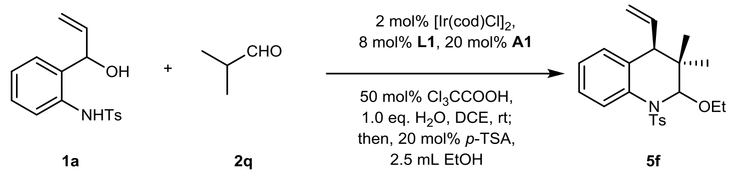

Supplementary procedure H: After the first step (as **Supplementary procedure B**), the solvent was removed *in vacuo* and *p*-TSA (20 mol%, 9.5 mg) and 2.5 mL EtOH was added under N_2. The mixture was stirred at 30 °C until the hemiaminal was disappeared determined by TLC. The residue was concetrated and purified by flash silica gel chromatography to afford the title compound as a white solid.

Procedure for the synthesis of 5g

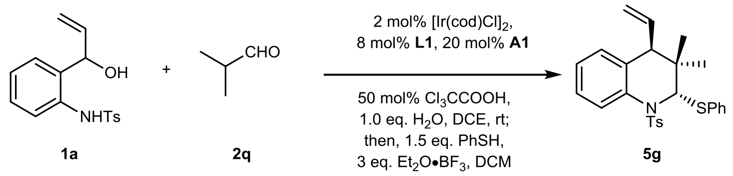

Supplementary procedure I: After the first step (as **Supplementary procedure B**), the mixture was diluted with 3 mL DCM under N_2, cooled to 0 °C in ice bath; then PhSH (1.5 eq., 41 mg, 38 μL) and $BF_3{\cdot}Et_2O$ (3.0 eq., 106.4 mg, 93 μL) were added successively. The mixture was stirred at 0 °C until the hemiaminal was disappeared determined by TLC. The residue was directly purified by flash silica gel chromatography to afford the title compound as a white solid.

Procedure for the synthesis of 5h and 5i

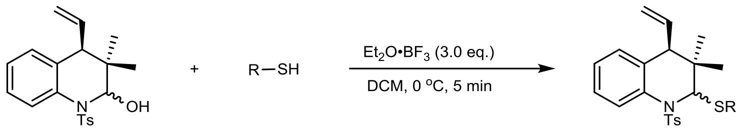

0.1 mmol　　0.15 mmol　　**5h** (R = Bn): 87% yield, 6:1 dr
5i (R = *t*-Bu): 83% yield, >20:1 dr

Supplementary procedure J: the mixture was diluted with 3 mL DCM under N_2, cooled to 0 °C in ice bath; then BnSH or ^{t}BuSH (1.5 eq.) and $BF_3{\cdot}Et_2O$ (3.0 eq.) were added successively. The mixture was stirred at 0 °C until the hemiaminal was disappeared determined by TLC. The residue was directly purified by flash silica gel chromatography to afford the title compound as colorless oil.

Procedure for the removal of N-Ts group

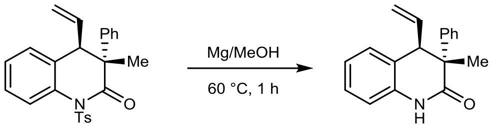

3aa: 0.1 mmol
99% ee, >20:1 dr

8aa: 91% yield
99% ee, >20:1 dr

Supplementary procedure K: Under argon atmosphere, a flame-dried 10 ml Schlenk tube was charged with charged 4aa (41.7 mg, 0.1 mmol, 99% ee, >20:1 d.r.), Mg (240 mg, 10 mmol, 200-300 mesh), and anhydrous MeOH (5 mL). The resulting solution was heated at 60 °C for 1 h. Then saturated NH_4Cl (3 mL) was added to the reaction mixture to quench excess magnesium powder. The aqueous phase was extracted with ethyl acetate (4×5 mL). The combined organic layers were dried over Na_2SO_4, filtered and concentrated in vacuo. Then the residue was purified by flash silica gel chromatography (Petrol ether/EtOAc = 5/1) to afford the product **8aa** in 91% yield (24 mg, 99% ee, >20:1 d.r.).

Procedure for the synthesis of 7

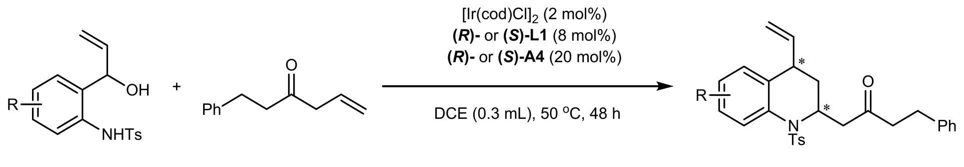

1　　6a　　7

Supplementary procedure L: In a dried Schlenk tube under N_2, $[Ir(cod)Cl]_2$ (0.005 mmol, 3.35 mg) and chiral ligand **(*R*)-L** or **(*S*)-L1** (0.02 mmol, 10.15 mg) were mixed in 0.3 mL DCE and stirred at ambient temperature for 20 min under argon atmosphere. Then allylic alcohol **1** (0.25 mmol, 1.0 eq), ketone **2** (0.5 mmol, 2.0 eq) and chiral amine catalyst **(*S*)-A4** or **(*R*)-A4** (0.05 mmol, 16.1 mg) were added to the mixture successively. After 8 h, the other 3.0 eq. ketone **2** was added in portions. Stirring at 50 °C for 48 h. The residue was directly purified by flash silica gel chromatography to afford the title compound.

Procedure for the synthesis of 3ar

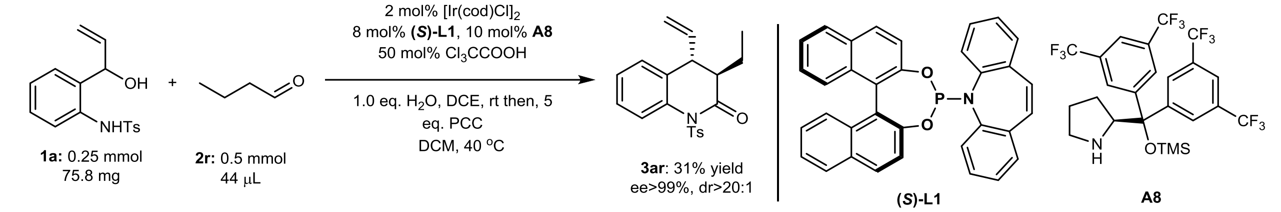

Supplementary procedure M: In a dried Schlenk tube under N_2, $[Ir(cod)Cl]_2$ (0.005 mmol, 3.35 mg) and chiral ligand **(*S*)-L1** (0.02 mmol, 10.15 mg) were mixed in 0.5 mL DCE and stirred at ambient temperature for 20 min under argon atmosphere. Then allylic alcohol **1a** (0.25 mmol, 1.0 eq), aldehyde **2r** (0.5 mmol, 2.0 eq), Cl_3CCOOH (0.125 mmol, 20.4 mg), chiral amine catalyst **A8** (0.025 mmol, 14.9 mg), 4.5 μL H_2O (1.0 eq.) were added to the mixture successively. The reaction mixture was stirred at ambient temperature until substrate **1a** disappeared on TLC. Then the mixture was diluted with 5 mL DCM, and PCC (275 mg, 5.0 eq.) and 100 mg silica gel were added to the mixture. The mixture was stirred at 40 °C for 10 h and the final product **3ar** was achieved by flash column chromatography.

(3*R*,4*R*)-3-Methyl-3-phenyl-1-tosyl-4-vinyl-3,4-dihydroquinolin-2(1H)-one (3aa)

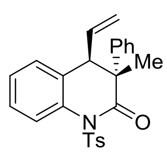

White solid, 64% yield. $[\alpha]_D^{25}$ = -21.7 (*c* = 1.00 in $CHCl_3$); ee = 99%, dr > 95:5, determined by HPLC analysis (Chiralpak OD column, hexane/*i*-PrOH, 90:10 v/v, flow rate 1 mL/min, λ = 254 nm, 25 °C), t_R (major) = 9.60 min, t_R (minor) = 19.22 min; **^{1}H NMR** (400 MHz, $CDCl_3$) *δ* (ppm) 7.99 (d, *J* = 8.3 Hz, 2H), 7.57 (d, *J* = 8.2 Hz, 1H), 7.37 (d, *J* = 8.1 Hz, 2H), 7.16 – 7.00 (m, 6H), 6.96 (m, 2H), 5.69 (m, 1H), 5.30 – 5.04 (m, 2H), 3.79 (d, *J* = 8.1 Hz, 1H), 2.48 (s, 3H), 1.39 (s, 3H); **^{13}C NMR** (100 MHz, $CDCl_3$) *δ* (ppm) 173.6, 145.0, 141.0, 136.5, 134.2, 134.1, 131.5, 129.3, 129.1, 128.4, 128.0, 127.1, 126.8, 126.1, 125.3, 123.2, 119.4, 53.4, 52.4, 24.7, 21.7; HRMS (ESI) for: $C_{25}H_{23}NO_3S$ $[M+H]^+$: calcd 418.1480, found 418.1471.

(3*R*,4*R*)-3,7-Dimethyl-3-phenyl-1-tosyl-4-vinyl-3,4-dihydroquinolin-2(1H)-one (3ba)

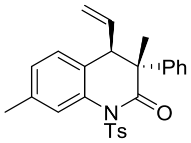

White solid, 59% yield; $[\alpha]_D^{25}$ = -33.6 (*c* = 1.00 in $CHCl_3$); ee = 96%, d.r. = 94:6, determined by HPLC analysis (Chiralpak OD column, hexane/*i*-PrOH, 90:10 v/v, flow rate 1 mL/min, λ = 254 nm, 25 °C), t_R (major) = 28.00 min, t_R (minor) = 12.03 min; **^{1}H NMR** (400 MHz, $CDCl_3$) *δ* (ppm) 7.98 (d, *J* = 8.4 Hz, 2H), 7.41 – 7.36 (m, 3H), 7.07 (m, 3H), 6.99 – 6.91 (m, 3H), 6.84 (d, *J* = 7.9 Hz, 1H), 5.71 – 5.62 (m, 1H), 5.23 – 5.06 (m, 2H), 3.76 (d, *J* = 8.1 Hz, 1H), 2.48 (s, 3H), 2.26 (s, 3H), 1.36 (s, 3H). **^{13}C NMR** (100 MHz, $CDCl_3$) *δ* (ppm) 173.8, 145.0, 141.2, 137.0, 136.6, 134.5, 133.9, 129.3, 129.2,

128.5, 127.7, 127.0, 126.8, 125.4, 123.8, 119.2, 117.2, 53.4, 52.0, 24.8, 21.8, 21.4. HRMS (ESI) for: $C_{26}H_{25}NO_3S$ $[M+H]^+$: calcd 432.1628, found 432.1636.

(3*R*,4*R*)-7-Fluoro-3-methyl-3-phenyl-1-tosyl-4-vinyl-3,4-dihydroquinolin-2(1H)-one (3ca)

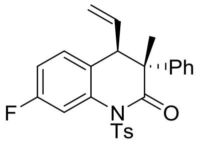

White solid, 59% yield; $[\alpha]_D^{25}$ = -4.0 (*c* = 1.00 in $CHCl_3$); ee = 94%, d.r. = 89:11, determined by HPLC analysis (Chiralpak OD column, hexane/*i*-PrOH, 90:10 v/v, flow rate 1 mL/min, λ = 254 nm, 25 °C), t_R (major) = 8.05 min, t_R (minor) = 22.40 min; **^{1}H NMR** (400 MHz, $CDCl_3$) *δ* (ppm) 7.98 (d, *J* = 8.4 Hz, 2H), 7.38 (m, 3H), 7.12 – 7.05 (m, 3H), 7.01 (dd, *J* = 8.4, 6.1 Hz, 1H), 6.98 – 6.91 (m, 2H), 6.75 (m, 1H), 5.70-5.62 (m, 1H), 5.21 – 5.09 (m, 2H), 3.79 (d, *J* = 7.9 Hz, 1H), 2.49 (s, 3H), 1.38 (s, 3H); **^{13}C NMR** (100 MHz, $CDCl_3$) *δ* (ppm) 173.2, 161.0 (d, *J* = 245.1 Hz), 145.3, 140.7, 136.2, 135.0 (d, *J* = 10.8 Hz), 133.9, 129.4, 129.1, 128.9 (d, *J* = 9.6 Hz), 128.6, 127.2, 127.0, 125.2, 119.6, 113.0 (d, *J* = 21.6 Hz), 111.0 (d, *J* = 27.1 Hz), 53.3, 51.7, 24.7, 21.8. **^{19}F NMR** (376 MHz, $CDCl_3$) *δ* (ppm) -112.9. HRMS (ESI) for: $C_{25}H_{22}FNO_3S$ $[M+H]^+$: calcd 436.1377, found 436.1382.

(3*R*,4*R*)-3-Methyl-3-phenyl-1-tosyl-7-(trifluoromethyl)-4-vinyl-3,4-dihydroquinolin-2(1H)-one (3da)

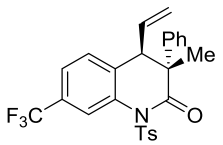

White solid, 66% yield; $[\alpha]_D^{25}$ = -24.8 (*c* = 1.00 in $CHCl_3$); ee = 98%, d.r. = 85:15, determined by HPLC analysis (Chiralpak OD column, hexane/*i*-PrOH, 90:10 v/v, flow rate 1 mL/min, λ = 254 nm, 25 °C), t_R (major) = 7.58 min, t_R (minor) = 13.98 min; **^{1}H NMR** (400 MHz, $CDCl_3$) *δ* (ppm) 7.99 (d, *J* = 8.1 Hz, 2H), 7.86 (s, 1H), 7.39 (d, *J* = 8.1 Hz, 2H), 7.29 (m, 1H), 7.18 (d, *J* = 7.9 Hz, 1H), 7.12 – 7.01 (m, 3H), 6.97 – 6.92 (m, 2H), 5.66 (m, 1H), 5.32 – 5.05 (m, 2H), 3.86 (d, *J* = 8.0 Hz, 1H), 2.49 (s, 3H), 1.40 (s, 3H); **^{13}C NMR** (100 MHz, $CDCl_3$) *δ* (ppm) 172.9, 145.4, 140.3, 136.1, 135.3, 134.6, 133.3 , 129.7, 129.4, 129.2, 128.7, 128.4, 127.2, 125.1, 124.9, 122.8 (q, *J* = 3.7 Hz), 122.2 , 120.2 (d, *J* = 4.4 Hz), 53.2, 52.3, 24.6, 21.7; **^{19}F NMR** (377 MHz, $CDCl_3$) *δ* (ppm) -62.9; HRMS (ESI) for: $C_{26}H_{22}NO_3F_3S$ $[M+H]^+$: calcd 486.1350, found 486.1345.

(3*R*,4*R*)-6-Methoxy-3-methyl-3-phenyl-1-tosyl-4-vinyl-3,4-dihydroquinolin-2(1H)-one (3ea)

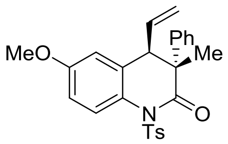

White solid, 61% yield; $[\alpha]_D^{25}$ = -37.0 (*c* = 1.00 in $CHCl_3$); ee = 99%, >95:5 d.r., determined by HPLC analysis (Chiralpak OD column, hexane/*i*-PrOH, 90:10 v/v, flow rate 1 mL/min, λ = 254 nm, 25 °C), t_R (major) = 11.04 min; **^{1}H NMR** (400 MHz, $CDCl_3$) *δ* (ppm) 7.98 – 7.96 (m, 2H), 7.48 (d, *J* = 9.0 Hz, 1H), 7.36 (d, *J* = 8.0 Hz, 2H), 7.11 – 6.97 (m, 5H), 6.66 – 6.59 (m, 2H), 5.64 – 5.55 (m, 1H), 5.26 – 5.09 (m, 2H), 3.75 – 3.72 (m, 4H), 2.47 (s, 3H), 1.36 (s, 3H); **^{13}C NMR** (100 MHz, $CDCl_3$) *δ* (ppm) 173.5, 157.5, 144.9, 141.0, 136.5, 134.1, 133.1, 129.2, 129.1, 128.4, 127.3, 126.8,

125.3, 124.5, 119.4, 113.6, 111.8, 55.4, 53.4, 52.7, 24.7, 21.7; HRMS (ESI) for: $C_{26}H_{25}NO_4S$ [M+H]$^+$: calcd 448.1586, found 448.1577.

(3*R*,4*R*)-6-Chloro-3-methyl-3-phenyl-1-tosyl-4-vinyl-3,4-dihydroquinolin-2(1H)-one (3fa)

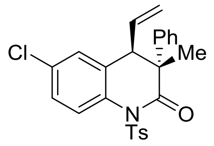

White solid, 60% yield; $[\alpha]_D^{25}$ = -32.3 (*c* = 1.00 in $CHCl_3$); ee = 99%, d.r. = 92:8, determined by HPLC analysis (Chiralpak OD column, hexane/*i*-PrOH, 90:10 v/v, flow rate 1 mL/min, λ = 254 nm, 25 ºC), t_R (major) = 8.50 min, t_R (minor) = 17.78 min; **^{1}H NMR** (400 MHz, $CDCl_3$) *δ* (ppm) 7.96 (d, *J* = 7.9 Hz, 2H), 7.52 (d, *J* = 8.7 Hz, 1H), 7.38 (d, *J* = 8.0 Hz, 2H), 7.17 – 7.01 (m, 5H), 6.96 (d, *J* = 7.5 Hz, 2H), 5.72 – 5.48 (m, 1H), 5.16 (m, 2H), 3.76 (d, *J* = 8.1 Hz, 1H), 2.48 (s, 3H), 1.37 (s, 3H); **^{13}C NMR** (100 MHz, $CDCl_3$) *δ* (ppm) 173.1, 145.3, 140.5, 136.1, 133.4, 133.3, 132.7, 131.6, 129.3, 129.2, 128.6, 127.7, 127.2, 127.1, 125.2, 124.5, 120.0, 53.2, 52.1, 24.6, 21.8; HRMS (ESI) for: $C_{25}H_{22}NO_3SCl$ [M+H]$^+$: calcd 452.1088, found 452.1082.

(3*R*,4*R*)-8-Fluoro-3-methyl-3-phenyl-1-tosyl-4-vinyl-3,4-dihydroquinolin-2(1H)-one (3ga)

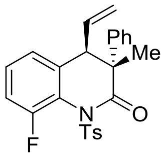

White solid, 73% yield; $[\alpha]_D^{25}$ = 16.1 (*c* = 1.00 in $CHCl_3$); ee = 99%, >95:5 d.r., determined by HPLC analysis (Chiralpak IC column, hexane/*i*-PrOH, 90:10 v/v, flow rate 1 mL/min, λ = 254 nm, 25 ºC), t_R (major) = 33.49 min, t_R (minor) = 23.95 min; **^{1}H NMR** (400 MHz, $CDCl_3$) *δ* (ppm) 8.23 (d, *J* = 8.1 Hz, 2H), 7.40 (d, *J* = 8.1 Hz, 2H), 7.04 (m, 4H), 6.95 – 6.90 (m, 2H), 6.87 (d, *J* = 7.8 Hz, 2H), 6.07 (m, 1H), 5.49 – 5.15 (m, 2H), 3.87 (d, *J* = 8.2 Hz, 1H), 2.49 (s, 3H), 1.50 (s, 3H); **^{13}C NMR** (100 MHz, $CDCl_3$) *δ* (ppm) 174.4 , 156.3 , 153.8 , 145.1 , 140.9 , 136.5 , 135.9 , 129.6 (d, *J* = 3.7 Hz), 129.2 , 128.5 , 127.9 (d, *J* = 8.3 Hz), 126.9 , 125.0 , 123.3 , 122.4 (d, *J* = 11.6 Hz), 120.2 , 115.1 (d, *J* = 21.2 Hz), 54.8 , 53.3 , 25.3 , 21.7 ; **^{19}F NMR** (377 MHz, $CDCl_3$) *δ* (ppm) -112.9; HRMS (ESI) for: $C_{25}H_{22}NO_3FS$ [M+H]$^+$: calcd 436.1386, found 436.1377.

(3*R*,4*R*)-3-Methyl-3-phenyl-1-tosyl-4-vinyl-3,4-dihydrobenzo[g]quinolin-2(1H)-one (3ha)

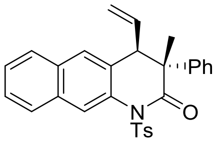

White solid, 63% yield; $[\alpha]_D^{25}$ = -106.8 (c = 1.00 in $CHCl_3$); ee = 98%, d.r. = 92:8, determined by HPLC analysis (Chiralpak OD column, hexane/*i*-PrOH, 90:10 v/v, flow rate 1 mL/min, λ = 254 nm, 25 ºC), t_R (major) = 11.86 min, t_R (minor) = 25.15 min; **^{1}H NMR** (400 MHz, $CDCl_3$) *δ* (ppm) 8.05 – 7.99 (m, 3H), 7.79 – 7.67 (m, 2H), 7.53 (s, 1H), 7.41 – 7.36 (m, 4H), 7.03 – 7.01 (m, 4H), 6.97 – 6.95 (m, 1H), 5.76-5.66 (m, 1H), 5.31 – 5.09 (m, 2H), 4.01 (d, *J* = 7.9 Hz, 1H), 2.48 (s, 3H), 1.42 (s, 3H). **^{13}C NMR** (100 MHz, $CDCl_3$) δ (ppm) 173.5, 145.1, 140.8, 136.4, 134.4, 132.1, 131.6,

131.2, 130.5, 129.3, 128.5, 128.3, 126. 9, 126.6, 126.2, 126.2, 125.4, 121.3, 119.6, 53.4, 52.5, 29.7, 21.8. HRMS (ESI) for: $C_{29}H_{25}NO_3SBr$ $[M+H]^+$: calcd 468.1628, found 468.1622.

(3*R*,4*R*)-3-(4-Chlorophenyl)-3-methyl-1-tosyl-4-vinyl-3,4-dihydroquinolin-2(1H)-one (3ab)

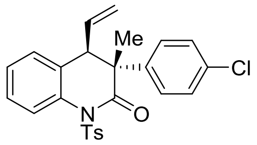

White solid, 61% yield; $[\alpha]_D^{25}$ = -8.9 (*c* = 1.00 in $CHCl_3$); ee>99%, d.r. = 88:12, determined by HPLC analysis (Chiralpak IF column, hexane/*i*-PrOH, 90:10 v/v, flow rate 1 mL/min, λ = 254 nm, 25 °C), t_R (major) = 17.96 min, t_R (minor) = 16.08 min; **^{1}H NMR** (400 MHz, $CDCl_3$) δ (ppm) 7.98 (d, *J* = 8.4 Hz, 2H), 7.58 (d, *J* = 8.2 Hz, 1H), 7.37 (d, *J* = 8.1 Hz, 2H), 7.19 – 7.14 (m, 1H), 7. 08 – 7.02 (m, 4H), 6.89 (d, *J* = 8.7 Hz, 2H), 6.70 – 5.61 (m, 1H), 5.21 – 5.12 (m, 2H), 3.75 (d, *J* = 8.1 Hz, 1H), 2.48 (s, 3H), 1.35 (s, 3H); **^{13}C NMR** (100 MHz, $CDCl_3$) δ (ppm) 173.1, 145.2, 139.6, 136.3, 134.0, 133.8, 132.6, 131.2, 129.3, 129.1, 128.6, 127.9, 127.4, 126.8, 126.3, 123.2, 119.7, 53.0, 52.2, 24.6, 21.7; HRMS (ESI) for: $C_{25}H_{22}NO_3SCl$ $[M+H]^+$: calcd 452.1081, found 452.1082.

(3*R*,4*R*)-3-(4-Fluorophenyl)-3-methyl-1-tosyl-4-vinyl-3,4-dihydroquinolin-2(1H)-one (3ac)

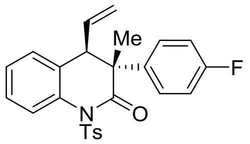

White solid, 55% yield; $[\alpha]_D^{25}$ = -14.3 (*c* = 1.00 in $CHCl_3$); ee = 96%, d.r. = 92:8, determined by HPLC analysis (Chiralpak AZ column, hexane/*i*-PrOH, 90:10 v/v, flow rate 1 mL/min, λ = 254 nm, 25 °C), t_R (major) = 31.47 min, t_R (minor) = 29.11 min; **^{1}H NMR** (400 MHz, $CDCl_3$) δ (ppm) 7.98 (d, *J* = 8.5 Hz, 2H), 7.58 (d, *J* = 8.1 Hz, 1H), 7.37 (d, *J* = 8.1 Hz, 2H), 7.16 (m, 1H), 7.10 – 7.03 (m, 2H), 6.98 – 6.88 (m, 2H), 6.80 – 6.72 (m, 2H), 5.65 (m, 1H), 5.25 – 5.05 (m, 2H), 3.74 (d, *J* = 8.1 Hz, 1H), 2.48 (s, 3H), 1.36 (s, 3H); **^{13}C NMR** (100 MHz, $CDCl_3$) δ (ppm) 173.4, 162.5, 160.1, 145.1, 136.8 (d, *J* = 3.5 Hz), 136.4, 134.1, 133.9, 131.3, 129.2 (d, *J* = 13.2 Hz), 127.9, 127.3, 127.0 (d, *J* = 8.1 Hz), 126.3, 123.2, 119.6, 115.3 (d, *J* = 21.3 Hz), 52.9, 52.5, 24.6, 21.7; **^{19}F NMR** (377 MHz, $CDCl_3$) δ (ppm) -115.6; HRMS (ESI) for: $C_{25}H_{22}NO_3FS$ $[M+Na]^+$: calcd 458.1204, found 458.1197.

(3*R*,4*R*)-3-(4-Methoxyphenyl)-3-methyl-1-tosyl-4-vinyl-3,4-dihydroquinolin-2(1H)-one (3ad)

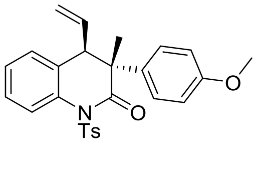

White solid, 71% yield; $[\alpha]_D^{25}$ = -15.2 (*c* = 1.00 in $CHCl_3$); ee = 99%, d.r. = 94:6, determined by HPLC analysis (Chiralpak OD column, hexane/*i*-PrOH, 90:10 v/v, flow rate 1 mL/min, λ = 254 nm, 25 °C), t_R (major) = 15.98 min, t_R (minor) = 24.09 min; **^{1}H NMR** (400 MHz, $CDCl_3$) δ (ppm) 7.98 (d, *J* = 8.3 Hz, 2H), 7.58 (d, *J* = 8.2 Hz, 1H), 7.37 (d, *J* = 8.1 Hz, 2H), 7.18 – 7.10 (m, 1H), 7.10 – 7.01 (m, 2H), 6.87 (d, *J* = 8.9 Hz, 2H), 6.60 (d, *J* = 8.9 Hz, 2H), 5.71-5.62 (m, 1H), 5.22 – 5.07 (m, 2H), 3.75 (d, *J* = 8.1 Hz, 1H), 3.66 (s, 3H), 2.48 (s, 3H), 1.35 (s, 3H). **^{13}C NMR** (100 MHz, $CDCl_3$) δ (ppm) 173.8, 158.1, 145.0, 136.6, 134.4, 134.1, 132.9, 131.6, 129.3, 129.2, 128.0, 127.2, 126.5, 126.2,

123.2, 119.4, 113.8, 55.1, 52.7, 52.5, 24.9, 21.8. HRMS (ESI) for: $C_{26}H_{25}NO_4S$ [M+H]$^+$: calcd 448.1577, found 448.1574.

(3*R*,4*R*)-3-(4-Isobutylphenyl)-3-methyl-1-tosyl-4-vinyl-3,4-dihydroquinolin-2(1H)-one (3ae)

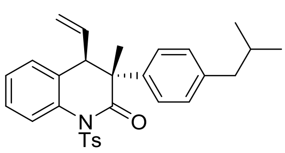

White solid, 62% yield; $[\alpha]_D^{25}$ = -7.5 (*c* = 1.00 in $CHCl_3$); ee = 99%, d.r. = 91:9, determined by HPLC analysis (Chiralpak OD column, hexane/*i*-PrOH, 90:10 v/v, flow rate 1 mL/min, λ = 254 nm, 25 °C), t_R (major) = 9.03 min, t_R (minor) = 13.56 min; **^{1}H NMR** (400 MHz, $CDCl_3$) *δ* (ppm) 7.99 (d, *J* = 8.0 Hz, 2H), 7.56 (d, *J* = 8.2 Hz, 1H), 7.37 (d, *J* = 8.0 Hz, 2H), 7.15 – 7.08 (m, 1H), 7.07 – 6.99 (m, 2H), 6.83 (s, 4H), 5.68 (dt, *J* = 17.4, 9.1 Hz, 1H), 5.30 – 5.09 (m, 2H), 3.76 (d, *J* = 8.2 Hz, 1H), 2.48 (s, 3H), 2.27 (d, *J* = 7.2 Hz, 2H), 1.76 – 1.62 (m, 1H), 1.38 (s, 3H), 0.75 (dd, *J* = 6.8, 2.5 Hz, 6H). **^{13}C NMR** (100 MHz, $CDCl_3$) *δ* (ppm) 137.8, 145.0, 140.2, 138.1, 136.5, 134.3, 134.1, 131.6, 129.3, 129.1, 129.0, 128.0, 127.0, 126.0, 125.0, 123.2, 119.3, 53.1, 52.6, 44.7, 30.0, 24.6, 22.2, 22.1, 21.7. HRMS (ESI) for: $C_{29}H_{31}NO_3S$ [M+H]$^+$: calcd 474.2097, found 474.2095.

(3*R*,4*R*)-3-Methyl-3-(m-tolyl)-1-tosyl-4-vinyl-3,4-dihydroquinolin-2(1H)-one (3af)

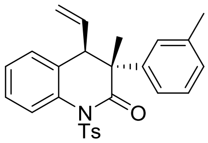

White solid, 73% yield; $[\alpha]_D^{25}$ = -20.6 (*c* = 1.00 in $CHCl_3$); ee = 99%, d.r.>95:5, determined by HPLC analysis (Chiralpak OD column, hexane/*i*-PrOH, 95:5 v/v, flow rate 1 mL/min, λ = 254 nm, 25 °C), t_R (major) = 17.06 min; **^{1}H NMR** (400 MHz, $CDCl_3$) *δ* (ppm) 7.99 (d, *J* = 8.0 Hz, 2H), 7.59 (d, *J* = 8.1 Hz, 1H), 7.37 (d, *J* = 8.1 Hz, 2H), 7.15 – 7.11 (m, 1H), 7.07 – 7.04 (m, 2H), 6.97 – 6.93 (m, 1H), 6.83 (d, *J* = 8.1 Hz, 1H), 6.74 (d, *J* = 10.2 Hz, 2H), 5.73 – 5.64 (m, 1H), 5.21 – 5.11 (m, 2H), 3.79 (d, *J* = 8.1 Hz, 1H), 2.48 (s, 3H), 2.14 (s, 3H), 1.37 (s, 3H). **^{13}C NMR** (100 MHz, $CDCl_3$) *δ* (ppm) 173.7, 145.0, 140.8, 137.9, 136.5, 134. 2, 134.1, 131.5, 129.3, 129.0, 128.2, 128.0, 127.6, 127.1, 126.1, 126.0, 123.1, 122.4, 119.4, 53.2, 52.3, 24.7, 21.7, 21.4. HRMS (ESI) for: $C_{26}H_{25}NO_3S$ [M+Na]$^+$: calcd 454.1447, found 454.1445.

(3*R*,4*R*)-3-(3-Bromophenyl)-3-methyl-1-tosyl-4-vinyl-3,4-dihydroquinolin-2(1H)-one (3ag)

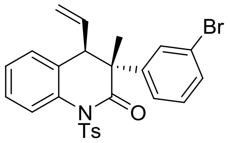

White solid, 63% yield; $[\alpha]_D^{25}$ = -7.6 (*c* = 1.00 in $CHCl_3$); ee = 99%, d.r.>95:5, determined by HPLC analysis (Chiralpak OZ column, hexane/*i*-PrOH, 95:5 v/v, flow rate 1 mL/min, λ = 254 nm, 25 °C), t_R (major) = 33.42 min; **^{1}H NMR** (400 MHz, $CDCl_3$) *δ* (ppm) 7.98 (d, *J* = 8.1 Hz, 2H), 7.61 (d, *J* = 8.2 Hz, 1H), 7.39 (d, *J* = 8.1 Hz, 2H), 7.21 – 7.13 (m, 2H), 7.09 (d, *J* = 3.1 Hz, 2H), 7.04 (d, *J* = 1.8 Hz, 1H), 6.94 (d, *J* = 7.7 Hz, 1H), 6.91–6.85 (m, 1H), 5.82–5.59 (m, 1H), 5.26 – 5.12 (m, 2H), 3.74 (d, *J* = 8.2 Hz, 1H), 2.48 (s, 3H), 1.37 (s, 3H). **^{13}C NMR** (100 MHz, $CDCl_3$) *δ* (ppm) 172.9, 145.2,

143.3, 136.3, 133.9, 133.5, 131.0, 130.1, 130.0, 129.4, 129.0, 128.6, 127.9, 127.4, 126.4, 124.1, 123.2, 122.5, 119.9, 53.2, 52.2, 24.3, 21.8. HRMS (ESI) for: $C_{25}H_{22}BrNO_3S$ [M+Na]$^+$: calcd 518.0396, found 518.0403.

3-((3*R*,4*R*)-3-Methyl-2-oxo-1-tosyl-4-vinyl-1,2,3,4-tetrahydroquinolin-3-yl)phenyl trifluoromethanesulfonate (3ah)

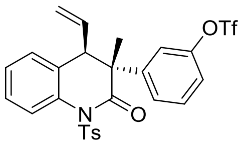

White solid, 68% yield; $[\alpha]_D^{25}$ = -3.0 (*c* = 1.00 in $CHCl_3$); ee = 98%, d.r. = 88:12, determined by HPLC analysis (Chiralpak OD column, hexane/*i*-PrOH, 95:5 v/v, flow rate 1 mL/min, λ = 254 nm, 25 °C), t_R (major) = 23.88 min, t_R (minor) = 27.88 min; **^{1}H NMR** (400 MHz, $CDCl_3$) *δ* (ppm) 8.05 (d, *J* = 8.1 Hz, 2H), 7.62 (d, *J* = 8.2 Hz, 1H), 7.43 (d, *J* = 8.1 Hz, 2H), 7.25 – 7.17 (m, 2H), 7.12 (s, 2H), 7.06 (d, *J* = 8.1 Hz, 1H), 7.00 (dd, *J* = 8.2, 2.3 Hz, 1H), 6.84 (s, 1H), 5.80 – 5.71 (m, 1H), 5.31 – 5.16 (m, 2H), 3.79 (d, *J* = 8.1 Hz, 1H), 2.53 (s, 3H), 1.44 (s, 3H). **^{13}C NMR** (100 MHz, $CDCl_3$) *δ* (ppm) 172.6, 149.4, 145.3, 144.3, 136.2, 133.8, 133.2, 130.8, 130.3, 129.4, 129.0, 127.9, 127.5, 126.5, 125.5, 123.3, 120.3, 119.7, 118.7, 53.5, 52.3, 24.0, 21.7. **^{19}F NMR** (376 MHz, $CDCl_3$) δ -72.7. HRMS (ESI) for: $C_{26}H_{22}F_3NO_6S_2$ [M+H]$^+$: calcd 566.0913, found 566.0915.

(3*R*,4*R*)-3-([1,1'-Biphenyl]-4-yl)-3-methyl-1-tosyl-4-vinyl-3,4-dihydroquinolin-2(1H)-one (3ai)

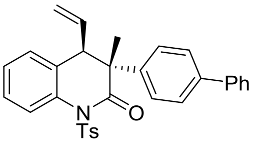

White solid, 75% yield; $[\alpha]_D^{25}$ = 23.1 (*c* = 1.00 in $CHCl_3$); ee = 99%, d.r. = 94:6, determined by HPLC analysis (Chiralpak OD column, hexane/*i*-PrOH, 90:10 v/v, flow rate 1 mL/min, λ = 254 nm, 25 °C), t_R (major) = 26.65 min, t_R (minor) = 27.88 min; **^{1}H NMR** (400 MHz, $CDCl_3$) *δ* (ppm) 8.00 (d, *J* = 8.1 Hz, 2H), 7.60 (d, *J* = 8.1 Hz, 1H), 7.44 (d, *J* = 7.5 Hz, 2H), 7.39 – 7.34 (m, 4H), 7.31 (d, *J* = 8.3 Hz, 3H), 7.16 – 7.09 (m, 2H), 7.04 (t, *J* = 8.5 Hz, 3H), 5.74 – 5.66 (m, 1H), 5.27 – 5.10 (m, 2H), 3.83 (d, *J* = 8.1 Hz, 1H), 2.47 (s, 3H), 1.42 (s, 3H). **^{13}C NMR** (100 MHz, $CDCl_3$) *δ* (ppm) 173.5, 145.0, 140.0, 140.0, 139.4, 136.4, 134.1, 134.0, 131.4, 129.3, 129.1, 128.6, 128.0, 127.3, 127.2, 127.0, 126.8, 126.2, 125.7, 123.2, 119.5, 53.1, 52.3, 24.7, 21.7. HRMS (ESI) for: $C_{31}H_{27}NO_3S$ [M+H]$^+$: calcd 494.1784, found 494.1776.

(3*R*,4*R*)-3-Methyl-3-(naphthalen-2-yl)-1-tosyl-4-vinyl-3,4-dihydroquinolin-2(1H)-one (3aj)

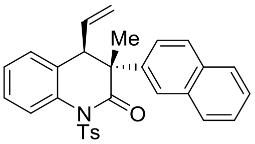

White solid, 75% yield; $[\alpha]_D^{25}$ = 25.3 (*c* = 1.00 in $CHCl_3$); ee = 98%, d.r. = 94:6, determined by HPLC analysis (Chiralpak IE column, hexane/*i*-PrOH, 90:10 v/v, flow rate 1 mL/min, λ = 254 nm, 25 °C), t_R (major) = 33.51 min, t_R (minor) = 37.53 min; **^{1}H NMR** (400 MHz, $CDCl_3$) *δ* (ppm) 8.00 (d, *J* = 8.0 Hz, 2H), 7.87 – 7.65 (m, 4H), 7.51 – 7.10 (m, 9H), 5.80 (s, 1H), 5.08 – 4.93 (m, 2H), 3.65 (s, 1H), 2.45 (s, 3H), 1.63 (s, 3H); **^{13}C NMR** (100 MHz, $CDCl_3$) *δ* (ppm) 174.4, 144.9, 136.7, 135.8, 135.4, 135.2, 134.5, 131.7, 131.1, 129.4, 129.1, 129.0, 128.8, 127.9, 127.4, 127.2, 126.2,

126.2, 125.0, 124.8, 124.4, 122.3, 119.5, 54.4, 52.9, 26.4, 21.7; HRMS (ESI) for: $C_{29}H_{25}NO_3S$ $[M+Na]^+$: calcd 490.1450, found 490.1447.

(3R,4R)-3-(6-Methoxynaphthalen-2-yl)-3-methyl-1-tosyl-4-vinyl-3,4-dihydroquinolin-2(1H)-one (3ak)

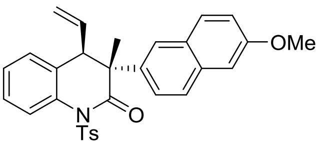

White solid, 71% yield; $[\alpha]_D^{25}$ = 50.0 (*c* = 1.00 in $CHCl_3$); ee = 98%, d.r.>95:5, determined by HPLC analysis (Chiralpak OD column, hexane/*i*-PrOH, 90:10 v/v, flow rate 1 mL/min, λ = 254 nm, 25 °C), t_R (major) = 36.28 min, t_R (minor) = 32.00 min; **^{1}H NMR** (400 MHz, $CDCl_3$) *δ* (ppm) 8.01 (d, *J* = 8.4 Hz, 2H), 7.56 (dd, *J* = 8.1, 1.2 Hz, 1H), 7.47 (dd, *J* = 8.8, 5.9 Hz, 2H), 7.43 – 7.36 (m, 2H), 7.15 – 7.07 (m, 2H), 7.07 – 7.02 (m, 2H), 7.01-6.97 (m, 1H), 6.95 (d, *J* = 2.5 Hz, 1H), 5.77 – 5.68 (m, 1H), 5.24 – 5.13 (m, 2H), 3.92 (d, *J* = 8.2 Hz, 1H), 3.83 (s, 3H), 2.50 (s, 3H), 1.44 (s, 3H). **^{13}C NMR** *δ* (ppm) 173.7, 157.7, 145.0, 136.5, 135.9, 134.2, 134.1, 133.1, 131.4, 129.3, 129.2, 129.1, 128.4, 127.9, 127.2, 127.1, 126.1, 124.2, 123.7, 123.1, 119.5, 118.9, 105.2, 55.2, 53.3, 52.2, 24.6, 21.8. HRMS (ESI) for: $C_{30}H_{27}NO_4S$ $[M+Na]^+$: calcd 520.1553, found 520.1561.

(3*R*,4*R*)-3-(Benzo[d][1,3]dioxol-5-yl)-3-methyl-1-tosyl-4-vinyl-3,4-dihydroquinolin-2(1H)-one (3al)

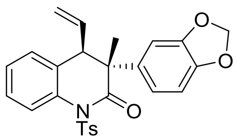

White solid, 57% yield; $[\alpha]_D^{25}$ = 1.7 (*c* = 1.00 in $CHCl_3$); ee>99%, d.r. = 95:5, determined by HPLC analysis (Chiralpak OD column, hexane/*i*-PrOH, 95:5 v/v, flow rate 1 mL/min, λ = 254 nm, 25 °C), t_R (major) = 36.14 min; **^{1}H NMR** (400 MHz, $CDCl_3$) *δ* (ppm) 7.98 (d, *J* = 8.0 Hz, 2H), 7.60 (d, *J* = 8.2 Hz, 1H), 7.37 (d, *J* = 8.0 Hz, 2H), 7.21 – 7.14 (m, 1H), 7.08 (d, *J* = 4.5 Hz, 2H), 6.58 – 6.44 (m, 2H), 6.44 – 6.36 (m, 1H), 5.83 (s, 2H), 5.62 (m, 1H), 5.26 – 5.02 (m, 2H), 3.70 (d, *J* = 8.2 Hz, 1H), 2.48 (s, 3H), 1.34 (s, 3H). **^{13}C NMR** (100 MHz, $CDCl_3$) *δ* (ppm) δ 173.5, 147.7, 146.2, 145.1, 136.4, 134.8, 134.1, 134.0, 131.4, 129.3, 129.1, 127.9, 127.2, 126.2, 123.2, 119.5, 118.8, 108.0, 105.9, 101.0, 53.0, 52.6, 24.8, 21.7. HRMS (ESI) for: $C_{26}H_{23}NO_5S$ $[M+H]^+$: calcd 462.1370, found 462.1359.

(3*R*,4*R*)-3-Methyl-1-tosyl-3-(1-tosyl-1H-indol-3-yl)-4-vinyl-3,4-dihydroquinolin-2(1H)-one (3am)

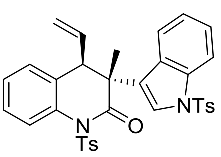

White solid, 56% yield; $[\alpha]_D^{25}$ = 40.7 (*c* = 1.00 in $CHCl_3$); ee>99%, d.r.>95:5, determined by HPLC analysis (Chiralpak OD column, hexane/*i*-PrOH, 95:5 v/v, flow rate 1 mL/min, λ = 254 nm, 25 °C), t_R (major) = 70.98 min; **^{1}H NMR** (400 MHz, $CDCl_3$) *δ* (ppm) 7.95 (d, *J* = 8.0 Hz, 2H), 7.77 (d, *J* = 8.3 Hz, 1H), 7.61 (d, *J* = 7.9 Hz, 1H), 7.35 (t, *J* = 7.9 Hz, 4H), 7.28 – 7.26 (m, 2H), 7.24 – 7.11 (m, 5H), 7.05 – 7.01 (m, 2H), 5.63 – 5.54 (m, 1H), 5.04 – 5.00 (m, 2H), 3.96 (d, *J* = 8.5 Hz, 1H), 2.48 (s, 3H), 2.35 (s, 3H), 1.44 (s, 3H). **^{13}C NMR** (100 MHz, $CDCl_3$) *δ* (ppm) 172.1, 145.1, 144.8, 136.3, 135.0, 134.9, 134.2, 133.0, 131.3, 129.8, 129.4, 129.2, 128.1, 127.7, 127.1, 126.6, 126.4, 124.6, 124.2, 123.2, 123.1,

121.4, 120.6, 120.0, 113.5, 50.8, 48.4, 21.7, 21.6, 20.9. HRMS (ESI) for: $C_{34}H_{30}N_2O_5S_2$ $[M+Na]^+$: calcd 633.1488, found 633.1495.

(*R*)-1'-Tosyl-4'-vinyl-1',4'-dihydro-2'H-spiro[cyclopentane-1,3'-quinolin]-2'-one (3an)

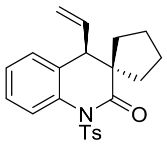

White solid, 64% yield; $[\alpha]_D^{25}$ = -169.1 (*c* = 1.00 in $CHCl_3$); ee>99%, determined by HPLC analysis (Chiralpak OD column, hexane/*i*-PrOH, 90:10 v/v, flow rate 1 mL/min, λ = 254 nm, 25 °C), t_R (major) = 15.05 min; **^{1}H NMR** (400 MHz, $CDCl_3$) *δ* (ppm) 7.92 (d, *J* = 8.1 Hz, 2H), 7.73 (d, *J* = 8.2 Hz, 1H), 7.37 – 7.29 (m, 3H), 7.24 – 7.14 (m, 2H), 5.64-5.56 (m, 1H), 5.09 – 4.98 (m, 2H), 3.11 (d, *J* = 7.3 Hz, 1H), 2.45 (s, 3H), 2.14 (dd, *J* = 13.4, 6.7 Hz, 1H), 1.81 – 1.70 (m, 1H), 1.56 (s, 6H), 1.43 (dd, *J* = 13.7, 7.4 Hz, 1H), 1.25 (d, *J* = 6.3 Hz, 1H). **^{13}C NMR** (100 MHz, $CDCl_3$) *δ* (ppm) 175.2, 144.8, 136.5, 135.3, 134. 6, 131.7, 129.1, 128.1, 127.2, 126.1, 123.3, 118.5, 55.0, 52.5, 36.1, 32.7, 25.7, 25.2, 21.7. HRMS (ESI) for: $C_{22}H_{23}NO_3S$ $[M+Na]^+$: calcd 404.1291, found 404.1284.

(*R*)-1'-Tosyl-4'-vinyl-1',4'-dihydro-2'H-spiro[cyclohexane-1,3'-quinolin]-2'-one (3ao)

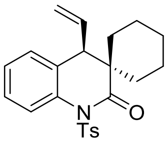

White solid, 74% yield; $[\alpha]_D^{25}$ = -167.6 (*c* = 1.00 in $CHCl_3$); ee>99%, determined by HPLC analysis (Chiralpak OD column, hexane/*i*-PrOH, 90:10 v/v, flow rate 1 mL/min, λ = 254 nm, 25 °C), t_R (major) = 9.37 min, t_R (minor) = 8.70 min; **^{1}H NMR** (400 MHz, $CDCl_3$) *δ* (ppm) 7.94 (d, *J* = 8.1 Hz, 2H), 7.74 (d, *J* = 8.2 Hz, 1H), 7.32 (d, *J* = 8.2 Hz, 3H), 7.24 – 7.15 (m, 2H), 5.58-5.49 (m, 1H), 5.14 – 5.01 (m, 2H), 3.42 (d, *J* = 8.4 Hz, 1H), 2.44 (s, 3H), 1.91-1.84 (m, 1H), 1.78 – 1.63 (m, 1H), 1.48 – 1.22 (m, 8H), 1.14 – 1.03 (m, 1H). **^{13}C NMR** (100 MHz, $CDCl_3$) *δ* (ppm) 175.0, 144.8, 136.6, 134.6, 134.2, 130.7, 129.2, 129.0, 128.2, 127.3, 126.4, 123.1, 118.3, 49.0, 46.8, 31.0, 29.8, 25.4, 21.6, 21.0, 20.9. HRMS (ESI) for: $C_{23}H_{25}NO_3S$ $[M+H]^+$: calcd 396.1628, found 396.1623.

(3*S*,4*R*)-3-Benzyl-3-methyl-1-tosyl-4-vinyl-3,4-dihydroquinolin-2(1H)-one(3ap)

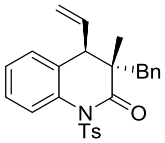

White solid, 77% yield; $[\alpha]_D^{25}$ = -70.3 (*c* = 0.40 in $CHCl_3$); ee>99%/>99%, d.r. = 69:31, determined by HPLC analysis (Chiralpak AD column, hexane/*i*-PrOH, 95:5 v/v, flow rate 1 mL/min, λ = 254 nm, 25 °C), diastereomer A: t_R (major) = 22.05 min, diastereomer B: t_R (major) = 28.87 min; **^{1}H NMR** (400 MHz, $CDCl_3$) *δ* (ppm) 1H NMR (400 MHz, $CDCl_3$) δ (ppm) 7.98 (d, *J* = 8.1 Hz, 3H, major+minor), 7.83 (d, *J* = 8.2 Hz, 1H, major), 7.73 (d, *J* = 8.2 Hz, 1H, minor), 7.42-7.38 (m, 4H, minor), 7.34-7.28 (m, 4H, major), 7.21 (dd, *J* = 7.6, 1.7 Hz, 4H, minor), 7.17 – 7.12 (m, 4H, major), 6.98 (s, 2H, major), 6.83 – 6.77 (m, 2H, minor), 5.90-5.81 (m, 1H, minor), 5.65-5.56 (m, 1H, major), 5.38 – 5.21 (m, 2H, minor), 5.10 – 4.96 (m, 2H, major), 3.25 (d, *J* = 8.5 Hz, 1H, minor), 3.13 (d, *J* = 8.3 Hz, 1H, major), 2.54 – 2.40 (m, 5H, major+minor), 1.06 (s, 3H, major), 0.86 (s, 1H, minor). **^{13}C NMR** (100 MHz, $CDCl_3$) *δ* (ppm) 174.2,

144.9, 136.4, 135.3, 134.5, 134.3, 130.6, 130.3, 129.2, 129.1, 128.6, 127.9, 127.5, 126.7, 126.4, 123.0, 118.8, 50.6, 48.4, 41.4, 21.7, 20.0. HRMS (ESI) for: $C_{26}H_{25}NO_3S$ $[M+H]^+$: calcd 432.1628, found 432.1640.

(3*R*,4*R*)-3-ethyl-1-tosyl-4-vinyl-3,4-dihydroquinolin-2(1H)-one (3ar)

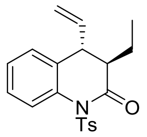

Colorless oil, 31% yield; $[\alpha]_D^{25}$ = 121.40 (*c* = 0.50 in $CHCl_3$); ee>99%, d.r. >20:1, determined by HPLC analysis (Chiralpak AD column, hexane/*i*-PrOH, 90:10 v/v, flow rate 1 mL/min, λ = 254 nm, 25 °C), diastereomer A: t_R (major) = 19.90 min; **^{1}H NMR** (400 MHz, $CDCl_3$) *δ* 7.91 (d, *J* = 8.4 Hz, 2H), 7.73 (dd, *J* = 8.2, 1.1 Hz, 1H), 7.41 – 7.14 (m, 5H), 5.67 (ddd, *J* = 16.8, 10.3, 6.2 Hz, 1H), 5.16 – 4.91 (m, 2H), 3.41 – 3.27 (m, 1H), 2.62 – 2.37 (m, 4H), 1.42-1.33 (m, 1H), 0.81 (t, *J* = 7.4 Hz, 3H). **^{13}C NMR** (100 MHz, $CDCl_3$) δ 172.2, 145.0, 136.8, 136.3, 134.4, 129.9, 129.4, 129.1, 128.2, 127.4, 126.3, 123.5, 117.9, 50.3, 44.3, 22.0, 21.7, 10.9. HRMS (ESI) for: $C_{20}H_{21}NO_3S$ $[M+Na]^+$: calcd 378.1134, found 378.1134.

(4*R*)-3,3-dimethyl-1-tosyl-4-vinyl-1,2,3,4-tetrahydroquinolin-2-ol (4aq)

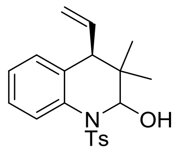

White solid, 91% yield, $[\alpha]_D^{25}$ = -78.77 (*c* = 1.0 in $CHCl_3$); ee>99%, d.r. =5:1, determined by HPLC analysis (Chiralpak AD column, hexane/*i*-PrOH, 90:10 v/v, flow rate 1 mL/min, λ = 254 nm, 25 °C), diastereomer A: t_R (major) = 21.66 min, t_R (minor) = 13.61 min; **^{1}H NMR** (400 MHz, Chloroform-d) δ 7.85 (d, *J* = 8.0 Hz, 2H, major), 7.71 (d, *J* = 8.2 Hz, 2H, minor), 7.53 (dd, *J* = 11.9, 8.3 Hz, 1H, major+minor), 7.31 (d, *J* = 7.5 Hz, 2H, major), 7.25 (d, *J* = 7.9 Hz, 2H, minor), 7.18 (d, *J* = 7.7 Hz, 1H), 7.13 (d, *J* = 7.6 Hz, 1H), 7.01 (t, *J* = 7.4 Hz, 1H), 5.98 – 5.78 (m, 1H, minor), 5.59 (s, 1H, major+minor), 5.55-5.46 (m, 1H, major), 5.30 – 5.13 (m, 2H, major+minor), 4.98 – 4.81 (m, 1H, minor), 3.40 (d, *J* = 9.3 Hz, 1H), 2.44 (s, 3H, major+minor), 1.13 (s, 3H, major), 1.03 (s, 3H, minor), 0.94 (s, 3H, major), 0.74 (s, 3H, minor). **^{13}C NMR** (101 MHz, CDCl3) δ 144.1, 135.7, 135.1, 129.9, 129.6, 129.4, 127.4, 127.0, 127.0, 123.2, 119.8, 118.9, 85.3, 48.40, 36.5, 24.3, 21.6, 21.2. **HRMS** (ESI) for: $C_{20}H_{23}NO_3S$ $[M+Na]^+$: calcd 380.1291, found 380.1294.

(*R*)-3,3-Dimethyl-1-tosyl-4-vinyl-3,4-dihydroquinolin-2(1H)-one (5a)

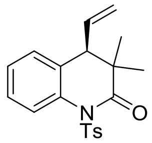

White solid, 88% yield; $[\alpha]_D^{25}$ = -119.9 (c = 1.00 in $CHCl_3$); ee>99%, determined by HPLC analysis (Chiralpak OD column, hexane/ *i*-PrOH, 90:10 v/v, flow rate 1 mL/min, λ = 254 nm, 25 °C), t_R (major) = 13.92 min, t_R (minor) = 10.47 min; **^{1}H NMR** (400 MHz, $CDCl_3$) *δ* (ppm) 7.97 (d, *J* = 8.4 Hz, 2H), 7.69 (d, *J* = 8.2 Hz, 1H), 7.36 – 7.31 (m, 3H), 7.28 – 7.14 (m, 2H), 5.73-5.64 (m, 1H), 5.25 – 5.07 (m, 2H), 3.12 (d, *J* = 8.6 Hz, 1H), 2.45 (s, 3H), 1.03 (s, 3H), 0.94 (s, 3H); **^{13}C NMR** (100 MHz, $CDCl_3$) *δ* (ppm) 175.5, 144.9, 136.6, 134.2, 134.1, 131.2, 129.3, 128.9, 127.7, 127.2, 126.3, 122.9, 119.3, 52.8, 44.1, 24.2, 21.7, 21.3. HRMS (ESI) for: $C_{20}H_{21}NO_3S$ $[M+Na]^+$: calcd 378.1134, found 378.1134.

(*S*)-1'-Tosyl-4'-vinyl-1',4'-dihydro-2'H-spiro[cyclohexane-1,3'-quinoline (5b)

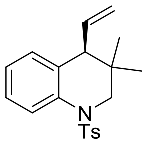

White solid, 87% yield; $[\alpha]_D^{25}$ = -78.2 (c = 1.00 in $CHCl_3$); ee>99%, determined by HPLC analysis (Chiralpak AS column, hexane/*i*-PrOH, 90:10 v/v, flow rate 1 mL/min, λ = 254 nm, 25 °C), t_R (major) = 13.10 min; **^{1}H NMR** (400 MHz, $CDCl_3$) *δ* (ppm) 7.71 (s, 1H), 7.69 (s, 1H), 7.65 (d, *J* = 8.5 Hz, 1H), 7.28 – 7.24 (m, 2H), 7.10 (t, *J* = 7.8 Hz, 1H), 7.07 – 7.03 (m, 1H), 6.96 (t, J = 7.4 Hz, 1H), 5.56 (dt, *J* = 17.1 Hz, 9.5 Hz, 1H), 5.11 (dd, *J* = 10.0 Hz, 1.9 Hz, 1H), 4.89 (dd, *J* = 17.0 Hz, 1.9 Hz, 1H), 3.72 (d, *J* = 12.1 Hz, 1H), 3.49 (d, *J* = 12.0 Hz, 1H), 2.92 (d, *J* = 9.0 Hz, 1H), 2.39 (s, 3H), 1.00 (s, 3H), 0.96 (s, 3H); **^{13}C NMR** (100 MHz, $CDCl_3$) *δ* (ppm) 143.6, 137.6, 137.0, 135.8, 130.2, 129.6, 128.6, 126.9, 126.9, 123.2, 119.6, 118.3, 56.0, 53.6, 32.3, 26.4, 22.4, 21.5. HRMS (ESI) for: $C_{20}H_{23}NO_2S$ $[M+Na]^+$: calcd 364.1342, found 364.1342.

(4*S*)-2-Allyl-3,3-dimethyl-1-tosyl-4-vinyl-1,2,3,4-tetrahydroquinoline (5c)

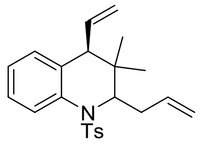

White solid, 91% yield; $[\alpha]_D^{25}$ = -55.1 (c = 1.00 in $CHCl_3$); d.r. = 72:27, ee>99%, determined by HPLC analysis (Chiralpak OD column, hexane/*i*-PrOH, 90:10 v/v, flow rate 1 mL/min, λ = 254 nm, 25 °C), diastereomer A: t_R (major) = 7.68 min, t_R (minor) = 8.91 min; diastereomer B: t_R (major) = 6.72 min, t_R (minor) = 5.88 min; **^{1}H NMR** (400 MHz, $CDCl_3$) δ (ppm) 7.85 (d, *J* = 8.4 Hz, 2H, major+minor), 7.69 (dd, *J* = 8.3, 1.2 Hz, 1H, major), 7.62 (dd, *J* = 8.0, 1.3 Hz, 1H, minor), 7.32 (d, *J* = 8.1 Hz, 2H, minor), 7.26 – 7.21 (m, 2H, major), 7.18-7.14 (m, 2H, major), 7.13 – 7.09 (m, 2H, minor), 7.00-6.96 (m, 1H, major), 6.88 (d, *J* = 7.6 Hz, 1H, minor), 6.11 – 5.97 (m, 1H, minor), 5.97 – 5.82 (m, 1H, major), 5.78 – 5.67 (m, 1H, minor), 5.65 – 5.54 (m, 1H, major), 5.36 – 5.16 (m, 2H, major), 5.15 – 5.12 (m, 2H, minor), 5.11 – 5.06 (m, 2H, minor), 5.05 – 4.92 (m, 2H, minor), 4.51 – 4.38 (m, 1H, major), 3.96 (t, *J* = 6.6 Hz, 1H, minor), 3.17 (d, *J* = 9.3 Hz, 1H, major), 2.48-2.42 (m, 1H, major+minor), 2.38 (s, 4H, major+minor), 2.17 – 1.98 (m, 1H, major), 1.61 (d, *J* = 9.9 Hz, 1H, minor), 1.07 (s, 3H, major), 0.91 (s, 3H, minor), 0.88 (s, 3H, major), 0.44 (s, 3H, minor). **^{13}C NMR** (100 MHz, $CDCl_3$) *δ* (ppm) 143.4, 136.8, 135.8, 134.4, 130.1, 129.2, 128.1, 127.2, 126.8, 123.3, 120.6, 119.5, 117.5, 65.5, 50.2, 34.7, 33.5, 26.0, 23.9, 21.5. HRMS (ESI) for: $C_{23}H_{27}NO_2S$ $[M+Na]^+$: calcd 404.1655, found 404.1654.

(4*S*)-3,3-Dimethyl-1-tosyl-4-vinyl-1,2,3,4-tetrahydroquinoline-2-carbonitrile (5d)

White solid, 90% yield, $[\alpha]_D^{25}$ = -62.6 (c = 1.00 in $CHCl_3$); ee>99%, determined by HPLC analysis (Chiralpak IF column, hexane/*i*-PrOH, 95:5 v/v, flow rate 1 mL/min, λ = 254 nm, 25 °C), t_R (major) = 14.64 min; **^{1}H NMR**

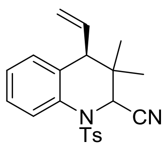

(400 MHz, $CDCl_3$) δ (ppm) 77.93 – 7.84 (m, 21H), 7.39 (d, J = 8.1 Hz, 2H), 7.28 – 7.17 (m, 2H), 7.06 (m, 2H), 5.68 – 5.54 (m, 1H), 5.44 (dd, J = 10.0 Hz, 1.9 Hz, 1H), 5.37 (s, 1H), 5.33 (dd, J = 16.9 Hz, 1.9 Hz, 1H), 3.50 (d, J = 9.2 Hz, 1H), 2.45 (s, 3H), 1.29 (s, 3H), 1.10 (s, 3H); **^{13}C NMR** (100 MHz, $CDCl_3$) δ (ppm) 144.6, 137.6, 135.1, 133.2, 130.4, 130.3, 127.3, 126.8, 126.4, 124.2, 121.3, 119.1, 116.6, 56.6, 50.5, 34.6, 25.0, 21.6, 19.9; HRMS (ESI) for: $C_{21}H_{22}N_2O_2S$ $[M+Na]^+$: calcd 389.1294, found 389.1294.

(4*R*)-2-Azido-3,3-dimethyl-1-tosyl-4-vinyl-1,2,3,4-tetrahydroquinoline (5e)

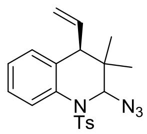

White solid, 84% yield, $[\alpha]_D^{25}$ = -252.2 (c = 1.00 in $CHCl_3$); ee>99%, determined by HPLC analysis (Chiralpak IC column, hexane/*i*-PrOH, 95:5 v/v, flow rate 1 mL/min, λ = 254 nm, 25 °C), t_R (major) = 14.95 min; **^{1}H NMR** (400 MHz, Acetone-*d*6) δ (ppm) 7.98 (dd, J = 8.3, 1.6 Hz, 2H), 7.73 (d, J = 8.3 Hz, 1H, major), 7.69 (d, J = 8.1 Hz, 1H, minor), 7.56 (d, J = 8.3, 2H, minor), 7.47 (d, J = 8.0 Hz, 2H, major), 7.37 (d, J = 7.9 Hz, 2H, minor), 7.32 (t, J = 7.8 Hz, 2H, minor)7.17 (t, J = 8.6 Hz, 2H, major), 7.02 (t, J = 7.5 Hz, 1H, major), 6.97 (d, J = 7.8 Hz, 1H, minor), 5.97 (s , 1H, major), 5.53 (s, 1H, minor),5.86 (m, 1H, minor), 5.71 – 5.57 (m, 1H, major), 5.45 – 5.27 (m, 2H, major), 5.21 – 5.13 (m, 2H, minor), 4.83 – 4.72 (m, 2H, minor), 3.37 (d, J = 9.5 Hz, 1H), 2.42 (s, 3H), 1.18 (s, 3H, major), 0.97 (s, 3H, monor), 0.83 (s, 3H, major), 0.63 (s, 3H, minor). **^{13}C NMR** (100 MHz, Acetone-*d*6) δ (ppm) 144.9, 136.8, 135.7, 133.4, 129.9, 129.6, 127.8, 127.1, 126.9, 123.4, 120.3, 118.5, 80.0, 48.1, 35.8, 24.2, 20.6, 19.8. HRMS (ESI) for: $C_{21}H_{22}N_2O_2S$ $[M+Na]^+$: calcd 405.1356, found 405.1363.

(4*R*)-2-Ethoxy-3,3-dimethyl-1-tosyl-4-vinyl-1,2,3,4-tetrahydroquinoline(5f)

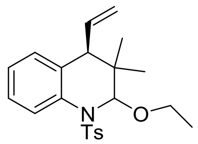

White solid, 77% yield, $[\alpha]_D^{25}$ = -111.2 (c = 1.00 in $CHCl_3$); ee>99%, d.r. = 80:20, determined by HPLC analysis (Chiralpak IE column*2, hexane/*i*-PrOH, 95:5 v/v, flow rate 1 mL/min, λ = 254 nm, 25 °C), diastereomer A: t_R (major) = 24.22 min; diastereomer B: t_R (major) = 26.40 min; **^{1}H NMR** (400 MHz, $CDCl_3$) δ (ppm) 7.85 (d, J = 8.2 Hz, 2H, major), 7.81 (d, J = 8.3 Hz, 2H, minor), 7.68 – 7.64 (m, 1H, major), 7.50-7.46 (m, 1H, minor), 7.28 – 7.23 (m, 2H, major), 7.21 (d, J = 8.1 Hz, 2H, minor), 7.17 (dt, J = 7.7, 1.4 Hz, 1H, major+minor), 7.10-7.05 (m, 1H, major), 7.03 (s, 1H, minor), 6.97-6.93 (m, 1H, major), 6.04-6.95 (m, 1H, minor), 5.70 – 5.53 (m, 1H, major), 5.41 (s, 1H, major), 5.36 (dd, J = 10.1, 2.2 Hz, 1H, major), 5.31 (s, 1H, minor), 5.25 (dd, J = 17.0, 2.2 Hz, 1H, major), 4.97 (dd, J = 9.9, 2.2 Hz, 1H, minor), 4.85 (dd, J = 17.0, 2.2 Hz, 1H, minor), 3.96 – 3.78 (m, 1H, major+minor), 3.75 – 3.57 (m, 1H, major+minor), 3.49 (d, J = 9.8 Hz, 1H, major), 2.52 (d, J = 10.0 Hz, 1H, minor), 2.38 (s, 4H, major+minor), 1.17 (t, J = 7.0 Hz, 4H, major+minor), 1.13 (s, 3H, major), 0.92 (s, 1H, minor), 0.90 (s, 1H, minor), 0.76 (s, 3H,

major); **^{13}C NMR** (100 MHz, $CDCl_3$) δ (ppm) 143.8, 136.9, 136.2, 133.6, 129.8, 129.4, 127.9, 127.8, 126.5, 122.9, 120.2, 119.0, 91.5, 64.7, 48.2, 35.5, 24.9, 21.5, 20.7, 14.9; HRMS (ESI) for: $C_{22}H_{27}NO_3S$ $[M+Na]^+$: calcd 408.1604, found 408.1604.

(4*R*)-3,3-Dimethyl-2-(phenylthio)-1-tosyl-4-vinyl-1,2,3,4-tetrahydroquinoline (5g)

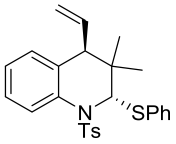

White solid, 77% yield, $[\alpha]_D^{25}$ = -370.6 (c = 1.00 in $CHCl_3$); ee>99%, d.r.>95:5, determined by HPLC analysis (Chiralpak OD column, hexane/*i*-PrOH, 90:10 v/v, flow rate 1 mL/min, λ = 254 nm, 25 °C), t_R (major) = 13.51 min; **^{1}H NMR** (400 MHz, $CDCl_3$) δ (ppm) 7.72 (d, *J* = 8.1, 2H), 7.56 – 7.49 (m, 2H), 7.36 – 7.27 (m, 3H), 7.24 (d, *J* = 8.3, 2H), 7.17 (d, *J* = 8.1, 2H), 7.07-7.02 (m, 2H), 5.98 (s, 1H), 5.74 – 5.60 (m, 1H), 5.46 – 5.22 (m, 2H), 3.61 (d, *J* = 9.4, 1H), 2.37 (s, 3H), 1.25 (s, 3H), 1.06 (s, 3H); **^{13}C NMR** (100 MHz, $CDCl_3$) δ (ppm) 143.6, 138.4, 136.2, 134.5, 134.0, 132.6, 130.2, 129.7, 129.2, 127.8, 127.6, 127.3, 126.9, 123.7, 120.4, 119.9, 76.4, 49.8, 37.7, 26.2, 22.3, 21.5; HRMS (ESI) for: $C_{26}H_{27}NO_2S_2$ $[M+Na]^+$: calcd 472.1375, found 472.1375.

(4*R*)-2-(benzylthio)-3,3-dimethyl-1-tosyl-4-vinyl-1,2,3,4-tetrahydroquinoline (5h)

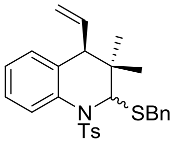

Colorless oil, 87% yield, d.r.=6:1. **^{1}H NMR** (400 MHz, $CDCl_3$) δ 7.93 (d, *J* = 8.1 Hz, 2H, major), 7.65 (d, *J* = 8.0 Hz, 2H, minor), 7.55 – 7.39 (m, 1H), 7.34 (d, *J* = 7.3 Hz, 2H), 7.31-7.22 (m, 6H, major+minor), 7.18 (d, *J* = 7.6 Hz, 1H), 7.08 (t, *J* = 7.8 Hz, 1H, major), 6.98 (t, *J* = 7.4 Hz, 1H, minor). 5.81-5.74 (m, 1H, minor), 5.71 – 5.55 (m, 2H, major), 5.34 (dd, *J* = 10.0, 2.0 Hz, 1H), 5.29 – 5.19 (m, 1H), 5.08 (d, J = 10.2 Hz, 1H, minor), 4.56 (d, *J* = 16.9 Hz, 1H, minor), 4.21 (d, *J* = 12.7 Hz, 1H, minor), 4.00 (d, *J* = 12.8 Hz, 1H, major), 3.90 (d, *J* = 12.8 Hz, 1H, major), 3.40 (d, *J* = 9.3 Hz, 1H), 2.41 (s, 3H), 1.92 (d, *J* = 9.7 Hz, 1H, minor), 0.98 (s, 3H, major), 0.88 (s, 3H, major+minor), 0.63 (s, 3H, minor). **^{13}C NMR** (100 MHz, $CDCl_3$) δ 143.9, 137.9, 137.7, 136.2, 133.9, 130.2, 129.7, 129.3, 128.5, 127.5, 127.2, 126.9, 123.5, 120.3, 119.9, 119.1, 72.6, 50.0, 36.8, 36.7, 25.9, 22.5, 21.6. ATR-IR: 3147, 2359, 1651, 1400 cm^{-1} HRMS (ESI) for: $C_{27}H_{29}NO_2S_2$ $[M+Na]^+$: calcd 486.1532, found 486.1534.

(2*S*,4*R*)-2-(tert-butylthio)-3,3-dimethyl-1-tosyl-4-vinyl-1,2,3,4-tetrahydroquinoline (5i)

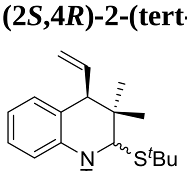

Colorless oil, 83% yield, d.r.>19:1. **^{1}H NMR** (400 MHz, $CDCl_3$) δ 8.03 – 7.93 (m, 2H), 7.32 (d, *J* = 7.9 Hz, 3H), 7.26 – 7.21 (m, 1H), 7.11-7.01 (m, 2H), 5.76 – 5.54 (m, 2H), 5.48 – 5.25 (m, 2H), 3.55 (d, *J* = 9.4 Hz, 1H), 2.45 (s, 3H), 1.45 (s, 9H), 1.27 (s, 3H), 1.05 (s, 3H). **^{13}C NMR** (100 MHz, $CDCl_3$) δ 143.6, 138.4, 136.4, 134.4, 130.3, 129.5, 128.2, 127.3, 126.7, 123.7, 121.0, 120.2, 70.0, 49.7, 45.1, 37.2, 32.2, 26.4, 22.6, 21.6. ATR-IR: 3420, 3148, 1614, 1400 cm^{-1}HRMS (ESI) for: $C_{24}H_{31}NO_2S_2$ $[M+Na]^+$: calcd 452.1688, found 452.1682.

(3*R*,4*R*)-3-methyl-3-phenyl-4-vinyl-3,4-dihydroquinolin-2(1H)-one (8aa)

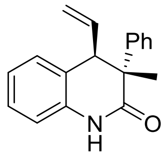

Colorless oil, 91% yield, $[\alpha]_D^{25}$ = -71.43 (c = 1.00 in $CHCl_3$); ee>99%, d.r.>95:5, determined by HPLC analysis (Chiralpak AD column, hexane/*i*-PrOH, 90:10 v/v, flow rate 0.4 mL/min, λ = 254 nm, 25 °C). t_R (major) = 24.11 min. **^{1}H NMR** (400 MHz, $CDCl_3$) *δ* (ppm) 8.67 (s, 1H), 7.35 – 7.29 (m, 2H), 7.18 (t, *J* = 7.6 Hz, 2H), 7.14 – 7.03 (m, 3H), 6.91 (m, 1H), 6.69 (d, *J* = 7.8 Hz, 1H), 5.93 (m, 1H), 5.21 – 4.99 (m, 2H), 3.94 (d, *J* = 8.8 Hz, 1H), 1.54 (s, 3H); **^{13}C NMR** (100 MHz, $CDCl_3$) *δ* (ppm) 174.1, 142.2, 135.7, 135.4, 128.3, 128.1, 127.6, 126.8, 126.5, 126.1, 123.4, 117.8, 115.2, 52.7, 49.5, 23.6; HRMS (ESI) for: $C_{18}H_{17}NO$ $[M+H]^+$: calcd 264.1378, found 264.1383.

4-Phenyl-1-((2*R*,4*R*)-1-tosyl-4-vinyl-1,2,3,4-tetrahydroquinolin-2-yl)butan-2-one ((*R,R*)-7a）

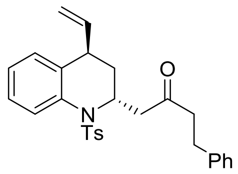

Colorless oil, 84% yield, $[\alpha]_D^{25}$ = -58.10 (c = 1.00 in $CHCl_3$); ee>99%, d.r.>95:5, determined by HPLC analysis (Chiralpak AD column, hexane/*i*-PrOH, 90:10 v/v, flow rate 1 mL/min, λ = 254 nm, 25 °C), t_R (major) = 14.25 min; **^{1}H NMR** (400 MHz, $CDCl_3$) *δ* 7.80 (d, *J* = 8.2 Hz, 1H), 7.41 – 7.36 (m, 2H), 7.30 – 7.23 (m, 3H), 7.21 – 7.07 (m, 7H), 4.99 – 4.79 (m, 4H), 3.22 – 3.09 (m, 1H), 2.89 (t, *J* = 7.6 Hz, 2H), 2.76-2.71 (m, 3H), 2.47-2.39 (m, 1H), 2.37 (s, 3H), 1.64-1.57 (m, 1H), 1.32-1.24 (m, 1H). **^{13}C NMR** (100 MHz, $CDCl_3$) *δ* 206.1, 142.7, 139.8, 138.9, 135.2, 133.2, 129.9, 128.5, 128.4, 127.5, 127.3, 126.5, 126.2, 126.0, 125.1, 124.7, 115.2, 49.7, 45.0, 43.6, 37.1, 29.4, 28.6, 20.5; HRMS (ESI) for: $C_{28}H_{29}NO_3S$ $[M+H]^+$: calcd 460.1941, found 460.1937.

4-Phenyl-1-((2*R*,4*S*)-1-tosyl-4-vinyl-1,2,3,4-tetrahydroquinolin-2-yl)butan-2-one ((*R,S*)-7a）

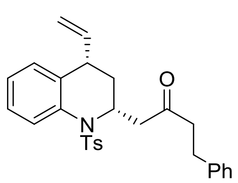

Clorless oil, 81% yield, $[\alpha]_D^{25}$ = 43.93 (c = 1.00 in $CHCl_3$); ee>99%, d.r.>95:5, determined by HPLC analysis (Chiralpak AD column, hexane/*i*-PrOH, 90:10 v/v, flow rate 1 mL/min, λ = 254 nm, 25 °C), t_R (major) = 12.01 min; **^{1}H NMR** (400 MHz, $CDCl_3$) δ 7.65 (dd, *J* = 8.0, 1.3 Hz, 1H), 7.32 – 7.24 (m, 6H), 7.18-7.16 (m, 5H), 6.97-6.94 (m, 1H), 5.62-5.53 (m, 1H), 5.08 (dd, *J* = 10.1, 1.7 Hz, 1H), 4.77 – 4.67 (m, 1H), 4.53-4.46 (m, 1H), 3.14 (dd, *J* = 16.9, 3.7 Hz, 1H), 2.96 – 2.60 (m, 5H), 2.39 (s, 3H), 2.26-2.20 (m, 1H), 1.85 – 1.75 (m, 1H), 1.14-1.04 (m, 1H). **^{13}C NMR** (100 MHz, $CDCl_3$) δ 207.8, 143.7, 140.8, 138.6, 137.4, 135.2, 134.7, 129.5, 128.5, 128.3, 127.2, 127.1, 126.4, 126.2, 125.0, 117.3, 53.0, 51.4, 45.0, 39.9, 37.9, 29.6, 21.5; HRMS (ESI) for: $C_{28}H_{29}NO_3S$ $[M+H]^+$: calcd 460.1941, found 472.1942.

4-Phenyl-1-((2*S*,4*R*)-1-tosyl-4-vinyl-1,2,3,4-tetrahydroquinolin-2-yl)butan-2-one ((*S*,*R*)-7a)

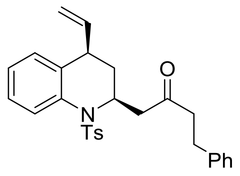

Colorless oil, 80% yield, $[\alpha]_D^{25}$ = -42.20 (c = 1.00 in $CHCl_3$); ee>99%, d.r.>95:5, determined by HPLC analysis (Chiralpak AD column, hexane/*i*-PrOH, 90:10 v/v, flow rate 1 mL/min, λ = 254 nm, 25 °C), t_R (major) = 14.25 min; HRMS (ESI) for: $C_{28}H_{29}NO_3S$ $[M+H]^+$: calcd 460.1941, found 460.1939.

4-Phenyl-1-((2*S*,4*S*)-1-tosyl-4-vinyl-1,2,3,4-tetrahydroquinolin-2-yl)butan-2-one ((*S*,*S*)-7a)

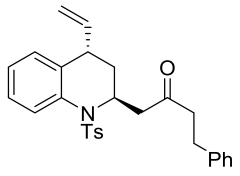

Colorless oil, 86% yield, $[\alpha]_D^{25}$ = 56.32 (c = 1.00 in $CHCl_3$); ee>99%, d.r.>95:5, determined by HPLC analysis (Chiralpak AD column, hexane/*i*-PrOH, 90:10 v/v, flow rate 1 mL/min, λ = 254 nm, 25 °C), t_R (major) = 14.25 min; HRMS (ESI) for: $C_{28}H_{29}NO_3S$ $[M+H]^+$: calcd 460.1941, found 460.1943.

1-((2*R*,4*R*)-7-Methyl-1-tosyl-4-vinyl-1,2,3,4-tetrahydroquinolin-2-yl)-4-phenylbutan-2-one ((*R*,*R*)-7b)

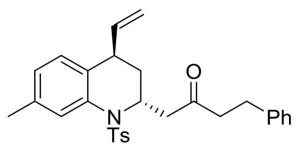

Clorless oil, 57% yield, $[\alpha]_D^{25}$ = -120.80 (c = 1.00 in $CHCl_3$); ee>99%, d.r. = 13:1, determined by HPLC analysis (Chiralpak AD column, hexane/*i*-PrOH, 95:5 v/v, flow rate 1 mL/min, λ = 254 nm, 25 °C), t_R (major) = 22.45 min; **^{1}H NMR** (400 MHz, $CDCl_3$) δ 7.65 (s, 1H), 7.42 (d, *J* = 8.3 Hz, 2H), 7.29 (dd, *J* = 8.3, 6.4 Hz, 3H), 7.23 – 7.16 (m, 4H), 7.03 – 6.97 (m, 2H), 5.00 – 4.81 (m, 4H), 3.17-3.11 (m, 1H), 2.93-2.89 (m, 2H), 2.79 – 2.67 (m, 3H), 2.48-2.42 (m, 1H), 2.40 (s, 3H), 2.38 (s, 3H), 1.64–1.58 (m, 1H), 1.30 – 1.23 (m, 1H). **^{13}C NMR** (100 MHz, $CDCl_3$) δ 207.2, 143.7, 140.8, 140.2, 137.0, 136.3, 133.9, 129.5, 129.27, 128.5, 128.3, 127.8, 127.5, 127.4, 126.8, 126.1, 115.9, 50.8, 45.9, 44.7, 37.8, 30.3, 29.6, 21.5, 21.3; HRMS (ESI) for: $C_{29}H_{31}NO_3S$ $[M+Na]^+$: calcd 516.1371, found 516.1369.

1-((2*R*,4*S*)-7-Methyl-1-tosyl-4-vinyl-1,2,3,4-tetrahydroquinolin-2-yl)-4-phenylbutan-2-one ((*R*,*S*)-7b)

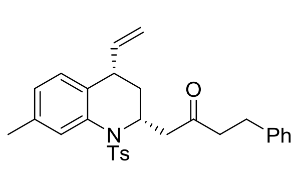

Clorless oil, 53% yield, $[\alpha]_D^{25}$ = -16.13 (c = 1.00 in $CHCl_3$); ee>99%, d.r.>95:5, determined by HPLC analysis (Chiralpak AD column, hexane/*i*-PrOH, 95:15 v/v, flow rate 1 mL/min, λ = 254 nm, 25 °C), t_R (major) = 16.95 min; **^{1}H NMR** (400 MHz, $CDCl_3$) δ 7.40 (d, *J* = 1.8 Hz, 1H), 7.25 – 7.21 (m, 2H), 7.20 – 7.17 (m, 3H), 7.13-7.09 (m, 4H), 6.93 – 6.88 (m, 1H), 6.78 – 6.74 (m, 1H), 5.53-5.44 (m, 1H), 4.99 (dd, *J* = 10.1, 1.8 Hz, 1H), 4.65-4.60 (m, 1H), 4.42-4.39 (m, 1H), 3.05 (dd, *J* = 16.8, 3.7 Hz, 1H), 2.86 – 2.75 (m, 2H), 2.72 – 2.50 (m, 3H), 2.32 (s, 3H), 2.31 (s, 3H), 2.15-2.09 (m, 1H), 1.71-1.67 (m, 1H), 1.04 – 0.91 (m, 1H). **^{13}C NMR** (100 MHz, $CDCl_3$) δ 206.7, 142.6, 139.8, 136.6, 135.9, 134.5, 134.2, 133.4, 128.4, 128.1, 127.5, 127.3, 126.1, 126.0, 125.1, 123.7, 116.0, 51.9,

50.4, 43.9, 38.5, 36.9, 28.6, 20.5, 20.2; HRMS (ESI) for: $C_{29}H_{31}NO_3S$ $[M+Na]^+$: calcd 516.1371, found 516.1371..

1-((2*R*,4*R*)-7-Fluoro-1-tosyl-4-vinyl-1,2,3,4-tetrahydroquinolin-2-yl)-4-phenylbutan-2-one ((*R*,*R*)-7c)

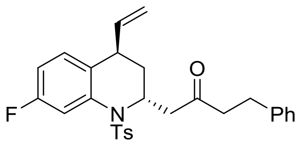

Clorless oil, 63% yield, $[\alpha]_D^{25}$ = -53.93 (c = 1.00 in $CHCl_3$); ee>99%, d.r.>95:5, determined by HPLC analysis (Chiralpak IF column, hexane/*i*-PrOH, 90:10 v/v, flow rate 1 mL/min, λ = 254 nm, 25 °C), t_R (major) = 17.96 min; **^{1}H NMR** (400 MHz, $CDCl_3$) δ 7.60 (dd, J = 10.9, 2.6 Hz, 1H), 7.44 (d, *J* = 8.1 Hz, 2H), 7.27 (d, *J* = 3.5 Hz, 2H), 7.19 (dd, *J* = 20.3, 7.6 Hz, 5H), 7.09 – 7.03 (m, 1H), 6.86 (td, *J* = 8.2, 2.6 Hz, 1H), 4.99 – 4.82 (m, 4H), 3.09 (s, 1H), 2.89 (t, *J* = 7.6 Hz, 2H), 2.72 (dd, *J* = 15.3, 7.0 Hz, 3H), 2.45 (dd, *J* = 16.6, 8.1 Hz, 1H), 2.39 (s, 3H), 1.71 – 1.56 (m, 3H), 1.28 – 1.11 (m, 2H). **^{13}C NMR** (100 MHz, $CDCl_3$) δ 206.9, 161.2 (d, *J* = 244 Hz), 160.0, 144.1, 140.7, 139.8, 135.9, 135.4, 130.7, 130.7 (d, *J* = 9 Hz), 129.6, 128.5, 128.3, 127.5, 126.3 (d, *J* = 3 Hz), 126.2, 116.4, 113.3 (d, *J* = 25 Hz), 112.9 (d, *J* = 22 Hz), 50.7, 46.0, 44.7, 37.7, 30.3, 29.6, 21.5. **^{19}F NMR** (377 MHz, $CDCl_3$) δ (ppm) -113.6; HRMS (ESI) for: $C_{28}H_{28}FNO_3S$ $[M+Na]^+$: calcd 500.1666, found 500.1666.

1-((2*R*,4*S*)-7-Fluoro-1-tosyl-4-vinyl-1,2,3,4-tetrahydroquinolin-2-yl)-4-phenylbutan-2-one ((*R*,*S*)-7c)

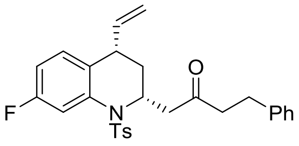

Clorless oil, 45% yield, $[\alpha]_D^{25}$ = 6.77 (c = 1.00 in $CHCl_3$); ee>99%, d.r.>95:5, determined by HPLC analysis (Chiralpak IF column, hexane/*i*-PrOH, 90:10 v/v, flow rate 1 mL/min, λ = 254 nm, 25 °C), t_R (major) = 18.23 min; **^{1}H NMR** (400 MHz, $CDCl_3$) δ 7.45 (dd, *J* = 10.0, 2.5 Hz, 1H), 7.36 (d, *J* = 8.3 Hz, 2H), 7.32 – 7.27 (m, 2H), 7.23-7.18 (m, 5H), 6.98 – 6.85 (m, 2H), 5.62-5.53 (m, 1H), 5.12 (dd, *J* = 10.2, 1.6 Hz, 1H), 4.80 – 4.73 (m, 1H), 4.58 – 4.43 (m, 1H), 3.17 (dd, *J* = 17.1, 3.5 Hz, 1H), 2.99 – 2.62 (m, 5H), 2.42 (s, 3H), 2.27-2.21 (m, 1H), 1.86-1.80 (m, 1H), 1.16-1.07 (m, 1H). **^{13}C NMR** (100 MHz, $CDCl_3$) δ 207.6, 161.4 (d, *J* = 243 Hz), 144.0, 140.8, 137.2, 136.0 (d, *J* = 11 Hz), 135.01, 133.9 (d, *J* = 3 Hz), 129.5, 128.5, 128.3, 127.1, 126.2, 126.0 (d, *J* = 7 Hz), 117.5, 115.6 (d, *J* = 24 Hz), 113.0 (d, *J* = 21 Hz), 53.0, 51.2, 44.9, 39.4, 37.7, 29.6, 21.5. **^{19}F NMR** (377 MHz, $CDCl_3$) δ (ppm) -114.4; HRMS (ESI) for: $C_{28}H_{28}FNO_3S$ $[M+Na]^+$: calcd 500.1666, found 500.1666.

1-((2*R*,4*R*)-7-Bromo-1-tosyl-4-vinyl-1,2,3,4-tetrahydroquinolin-2-yl)-4-phenylbutan-2-one ((*R*,*R*)-7d)

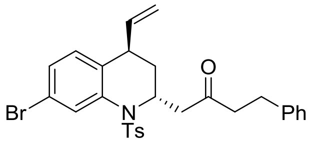

Clorless oil, 81% yield, $[\alpha]_D^{25}$ = -84.93 (c = 1.00 in $CHCl_3$); ee>99%, d.r.>95:5, determined by HPLC analysis (Chiralpak IF column, hexane/*i*-PrOH, 90:10 v/v, flow rate 1 mL/min, λ = 254 nm, 25 °C), t_R (major) = 18.03 min; **^{1}H NMR** (400 MHz, $CDCl_3$) δ 7.92 (d, *J* = 2.0 Hz, 1H), 7.36 (d, *J* = 8.4 Hz, 2H), 7.21-7.17 (m, 3H), 7.15 – 7.07 (m, 5H), 6.90 (dd, *J* = 8.4, 0.9 Hz, 1H), 4.88 – 4.82 (m, 3H), 4.80-4.74 (m, 1H), 2.99 (dd, *J* = 6.8, 4.0 Hz, 1H), 2.88 – 2.76 (m,

2H), 2.66 – 2.60 (m, 3H), 2.36 (dd, *J* = 8.9, 7.7 Hz, 1H), 2.31 (s, 3H), 1.57-1,51 (m, 1H), 1.21 – 1.08 (m, 1H). **^{13}C NMR** (100 MHz, $CDCl_3$) δ 206.9, 144.1, 140.7, 139.4, 135.8, 135.6, 130.9, 129.8, 129.7, 129.3, 128.7, 128.6, 128.3, 127.5, 126.2, 120.5, 116.7, 50.6, 45.9, 44.7, 37.8, 30.2, 29.6, 21.6; HRMS (ESI) for: $C_{28}H_{28}BrNO_3S$ $[M+Na]^+$: calcd 560.0865, found 560.0857.

1-((2*R*,4*S*)-7-Bromo-1-tosyl-4-vinyl-1,2,3,4-tetrahydroquinolin-2-yl)-4-phenylbutan-2-one ((*R*,*S*)-7d)

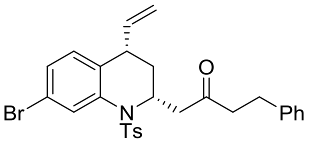

Clorless oil, 74% yield, $[\alpha]_D^{25}$ = -34.60 (c = 1.00 in $CHCl_3$); ee>99%, d.r.>95:5, determined by HPLC analysis (Chiralpak IF column, hexane/*i*-PrOH, 90:10 v/v, flow rate 1 mL/min, λ = 254 nm, 25 °C), t_R (major) = 17.09 min; **^{1}H NMR** (400 MHz, $CDCl_3$) δ 7.82 (d, *J* = 2.1 Hz, 1H), 7.33 (d, 2H), 7.31 – 7.24 (m, 4H), 7.23 – 7.12 (m, 5H), 6.83 (d, *J* = 8.2 Hz, 1H), 5.57-5.48 (m, 1H), 5.10 (dd, *J* = 10.1, 1.6 Hz, 1H), 4.72 (d, *J* = 17.1 Hz, 1H), 4.45 (dd, *J* = 13.5, 6.7 Hz, 1H), 3.14 (dd, *J* = 17.1, 3.5 Hz, 1H), 2.99 – 2.57 (m, 5H), 2.40 (s, 3H), 2.25-2.19 (m, 1H), 1.83 – 1.69 (m, 1H), 1.21 – 0.94 (m, 1H). **^{13}C NMR** (100 MHz, $CDCl_3$) δ 207.5, 144.0, 140.7, 137.4, 136.8, 136.1, 135.0, 131.1, 129.6, 129.3, 128.5, 128.3, 127.2, 126.4, 126.2, 120.2, 117.8, 53.02 51.2, 44.9, 39.6, 37.6, 29.6, 21.5; HRMS (ESI) for: $C_{28}H_{28}BrNO_3S$ $[M+Na]^+$: calcd 560.0865, found 560.0860.

1-((2*R*,4*R*)-6-Chloro-1-tosyl-4-vinyl-1,2,3,4-tetrahydroquinolin-2-yl)-4-phenylbutan-2-one ((*R*,*R*)-7e)

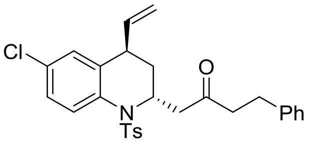

Clorless oil, 66% yield, $[\alpha]_D^{25}$ = -81.87 (c = 1.00 in $CHCl_3$); ee>99%, d.r. = 9:1, determined by HPLC analysis (Chiralpak AD column, hexane/*i*-PrOH, 90:10 v/v, flow rate 1 mL/min, λ = 254 nm, 25 °C), t_R (major) = 12.76 min; **^{1}H NMR** (400 MHz, $CDCl_3$) δ 7.75 (d, *J* = 8.8 Hz, 1H), 7.39 (d, *J* = 8.3 Hz, 2H), 7.30 – 7.23 (m, 2H), 7.23 – 7.13 (m, 6H), 7.08 (dd, *J* = 2.5, 0.9 Hz, 1H), 5.01 – 4.88 (m, 3H), 4.88 – 4.79 (m, 1H), 3.09 (dt, *J* = 11.0, 7.1 Hz, 1H), 2.88 (t, *J* = 7.2 Hz, 2H), 2.80 – 2.64 (m, 3H), 2.45 – 2.40 (m, 1H), 2.39 (s, 3H), 1.62 – 1.53 (m, 1H), 1.26 – 1.15 (m, 1H). **^{13}C NMR** (100 MHz, $CDCl_3$) δ 206.9, 144.1, 140.7, 139.1, 135.8, 132.9, 132.6, 131.3, 129.7, 129.3, 128.5, 128.3, 128.3, 127.5, 127.5, 126.2, 117.0, 50.6, 45.9, 44.6, 38.1, 29.9, 29.6, 21.6; HRMS (ESI) for: $C_{28}H_{28}ClNO_3S$ $[M+Na]^+$: calcd 516.1371, found 516.1369.

1-((2*R*,4*S*)-6-Chloro-1-tosyl-4-vinyl-1,2,3,4-tetrahydroquinolin-2-yl)-4-phenylbutan-2-one ((*R*,*S*)-7e)

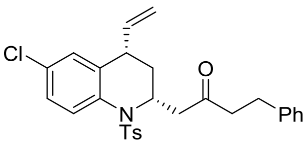

Clorless oil, 59% yield, $[\alpha]_D^{25}$ = -4.93 (c = 1.00 in $CHCl_3$); ee>99%, d.r.>95:5, determined by HPLC analysis (Chiralpak AD column, hexane/*i*-PrOH, 90:10 v/v, flow rate 1 mL/min, λ = 254 nm, 25 °C), t_R (major) = 10.89 min; **^{1}H NMR** (400 MHz, $CDCl_3$) δ 7.57 (d, *J* = 8.5 Hz, 1H), 7.35 – 7.28 (m, 3H), 7.27 – 7.10 (m, 7H), 6.94 (dd, *J* = 2.5, 1.1 Hz, 1H), 5.57-5.48 (m, 1H), 5.12 (dd, *J* = 10.1, 1.6 Hz, 1H), 4.72 (dt, *J* = 17.0, 1.2 Hz, 1H), 4.52 – 4.38 (m, 1H),

3.13 (dd, J = 17.0, 3.5 Hz, 1H), 2.96 – 2.81 (m, 2H), 2.80 – 2.61 (m, 3H), 2.41 (s, 3H), 2.25-2.19 (m, 1H), 1.78-1.72 (m, 1H), 1.17 – 0.98 (m, 1H). **^{13}C NMR** (100 MHz, $CDCl_3$) δ 207.6, 144.0, 140.8, 140.4, 136.4, 134.9, 133.4, 132.1, 129.7, 129.6, 128.5, 128.3, 127.3, 127.1, 126.2, 125.2, 118.1, 53.0, 51.2, 44.9, 39.9, 37.6, 29.6, 21.5; HRMS (ESI) for: $C_{28}H_{28}ClNO_3S$ $[M+Na]^+$: calcd 516.1371, found 516.1371.

(*R*)-4-Phenyl-1-((2*R*,4*S*)-1-tosyl-4-vinyl-1,2,3,4-tetrahydroquinolin-2-yl)butan-2-yl 4-nitrobenzoate (*R*,*S*)-8a

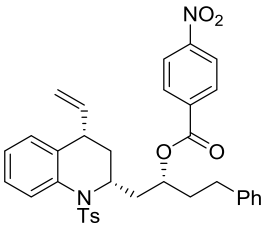

White solid, 73% yield, $[\alpha]_D^{25}$ = 46.17 (c = 1.00 in $CHCl_3$). **^{1}H NMR** (400 MHz, $CDCl_3$) δ 8.33 (d, J = 8.5 Hz, 2H), 8.24 (d, J = 8.5 Hz, 2H), 7.68 (d, J = 8.0 Hz, 1H), 7.34 – 7.22 (m, 4H), 7.19 – 7.11 (m, 5H), 7.08 (d, J = 8.0 Hz, 2H), 6.94 (d, J = 7.6 Hz, 1H), 5.59 (dt, J = 18.0, 9.4 Hz, 1H), 5.34-5.28 (m, 1H), 5.09 (d, J = 10.2 Hz, 1H), 4.68 (d, J = 17.0 Hz, 1H), 4.34 – 4.20 (m, 1H), 2.73 (dt, J = 9.6, 4.5 Hz, 2H), 2.47-2.40 (m, 1H), 2.35 (s, 3H), 2.22 – 2.04 (m, 3H), 1.91-1.84 (m, 1H), 1.81 – 1.68 (m, 1H), 1.24 (td, J = 12.4, 9.5 Hz, 1H). **^{13}C NMR** (100 MHz, $CDCl_3$) δ 164.4, 150.6, 143.6, 141.1, 138.4, 137.4, 135.7, 135.6, 134.6, 130.8, 129.4, 128.9, 128.5, 128.3, 127.3, 126.9, 126.5, 126.1, 125.0, 123.6, 117.4, 73.5, 53.3, 42.7, 39.6, 37.8, 36.0, 31.7, 21.4. HRMS (ESI) for: $C_{28}H_{28}ClNO_3S$ $[M+K]^+$: calcd 649.1769, found 649.1764.

Supplementary Note 1

Determination of the Absolute Configurlations of Products

The absolute configuration of products **3** were determined to be (3*R*,4*R*) by comparing with the chiral HPLC spectra of our previous work (*J. Am. Chem. Soc.* **2017**, *139*, 14707−14713). The absolute configuration of chiral product **5g** was determined to be (2*S*,4*R*) through the X-ray single crystal diffraction analysis **(CCDC 1821791)**.

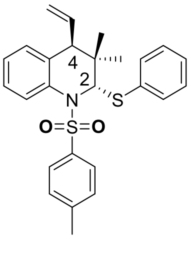

5g

CCDC 1821791

≡

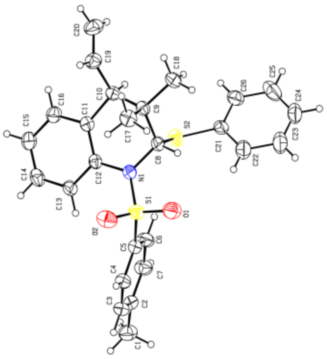

Given the fact that product **7a** is an oil at room temperature, it was converted to a solid product **8a** through a two-step operation. The absolute configuration of **8a** was established through the X-ray single crystal diffraction analysis **(CCDC 1869868)** and thus the structure of compound **7a** was

determined to be (2*R*,4*S*). The configurations of other stereoisomers were proposed based on this result together with the chiral HPLC and previous literatures (Carreira, E. M. *Science* **2013**, *340*, 1065-1068; *J. Am. Chem. Soc.* **2014**, *136*, 3020-3023).

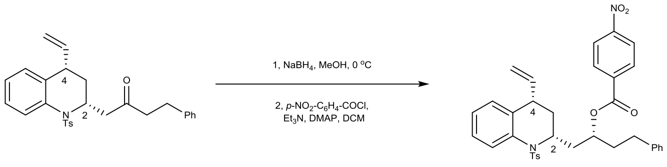

***(R,S)*-7a** ***(R,S)*-8a**: yield=73%

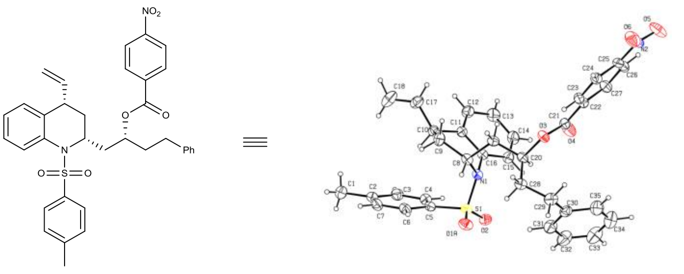

(*R*,*S*)-8a CCDC 1869868

Supplementary procedure N for the synthesis of 8a: To a suspension of $NaBH_4$ (82 mg, 0.18 mmol) in methanol (10 mL) was added **(*R*,*S*)-7a** (82 mg,0.18 mmol) at 0 °C, the mixture was stired for 20 min. Then the rection mixture was quenchend with saturated *aq.* NH_4Cl (10 mL). The aqueous layer was extracted with DCM and the organic phase was dried over Na_2SO_4, filtered and concentrated *in vacuo.* The crude residue was used directly for the next step. The crude acohol was disolved in 4 mL DCM, Et_3N (3.0 eq., 0.54 mmol, 55 mg), DMAP (0.1 eq., 0.018 mmol, 2 mg) were added to the mixture at 0 °C. Then 4-nitrobenzoyl chloride (3.0 eq., 0.54 mmol, 100 mg) was added, and the mixture was stired at 0 °C for 0.5 h. Then the rection mixture was quenchend with saturated *aq.* NH_4Cl (10 mL). The aqueous layer was extracted with DCM and the organic phase was dried over Na_2SO_4, filtered and concentrated *in vacuo.* The final product was achieved by flash column chromatography as white solid in 73% yield.

Supplementary Discussion 1

Preliminary Mechanism Study with HRMS

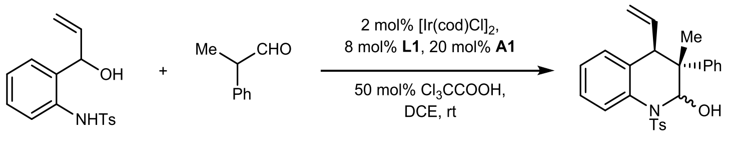

1a **2a** **4aa**

Under standard reaction conditions, the key intermidiates **V** and **VI** (**4aa**) were detected by HRMS after 12 h, which was in correspondence with the proposed mechanism to generate hemiaminal **4aa** in

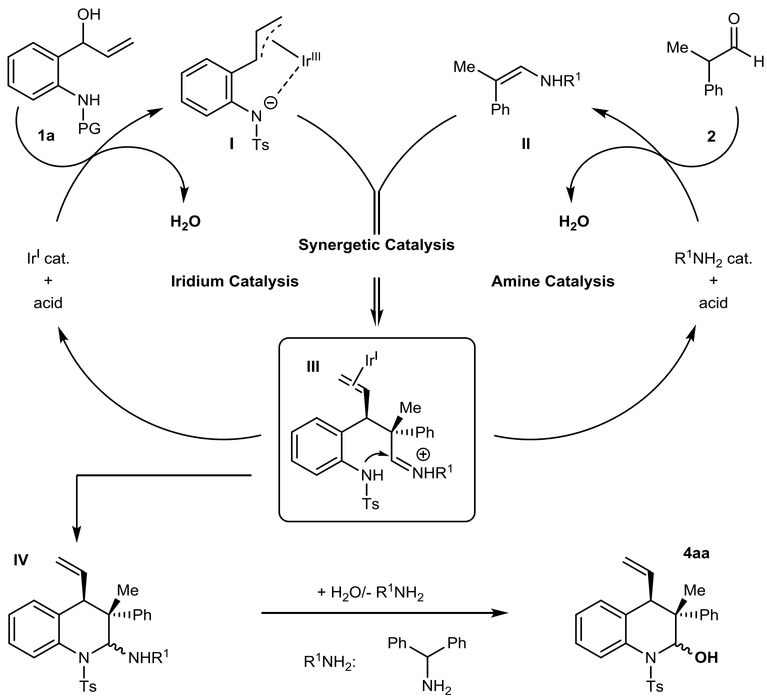

Supplementary Figure 92. The proposed mechanism

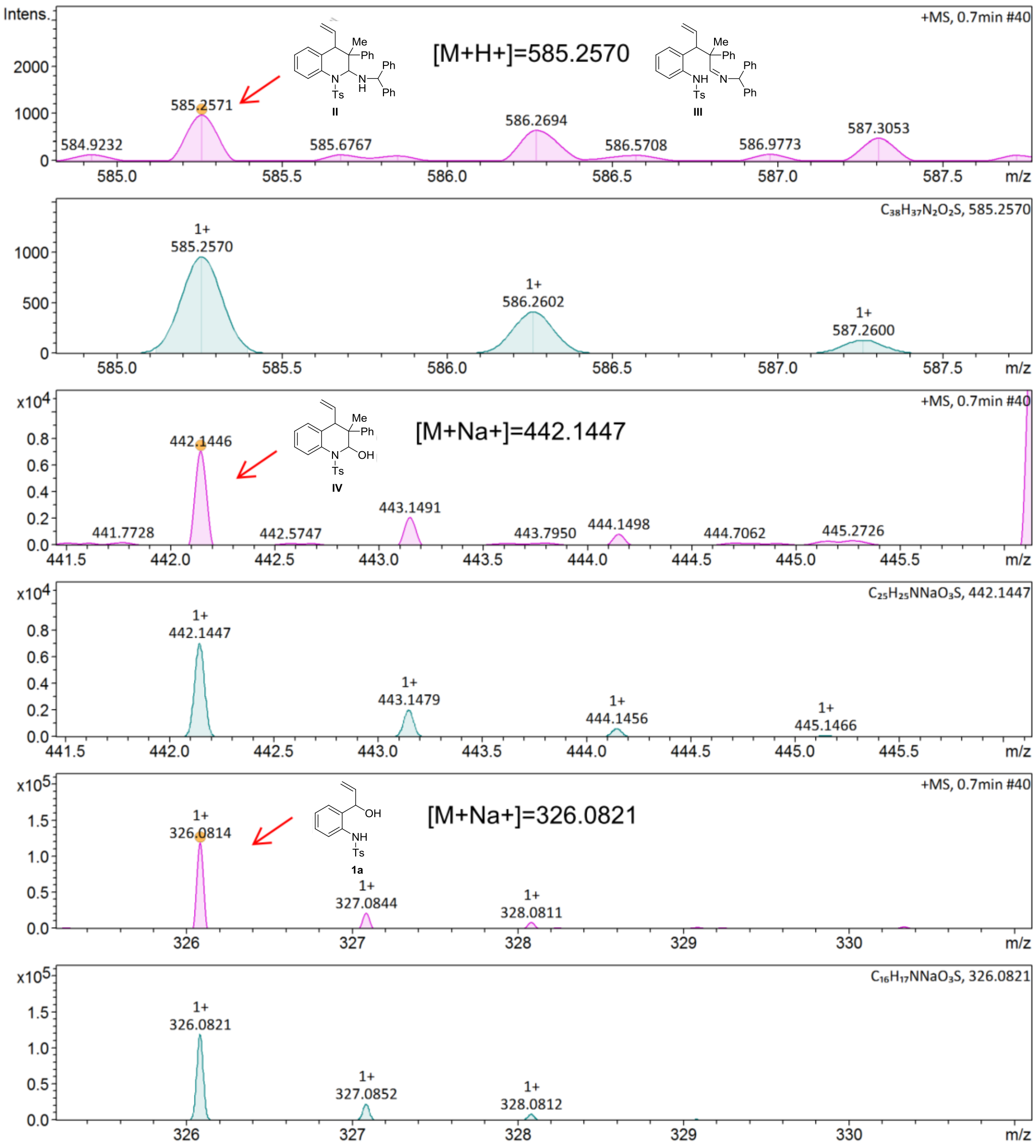

Supplementary Figure 93. The copies of HRMS of reaction mixture (12 h)

Supplementary Discussion 2

Proposed Stereo-Control Models for Asymmetric Cycloadditions

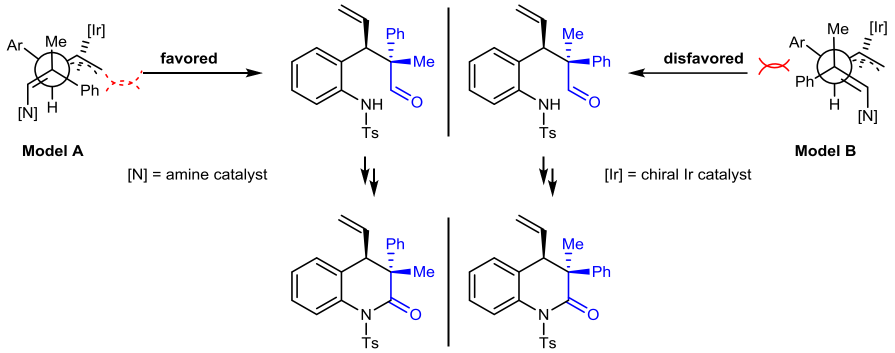

Supplementary Figure 94. Proposed stereochemical models for aldehyde cycloadditions

According to Sunoj's detailed computational study[8] and Carreira's experimental mechanistic study[9,10] on the Ir-catalyzed allylic alkylation of carbonyls, the chiral π-allyl-Ir complex controls the enantioselectivity of the stereocenter on the allyl part (as shown in **Supplementary Figure 94**, Model A). The *Si*- or *Re*-face attack of enamine nucleophile onto the π-allyl-Ir complex leads to two different diasteroisomers. Based on these information, Model A and B was depicted with the formulas Newman projection. It was proposed that the different steric repulsion between enamine and π-allyl-Ir complex resulted in diasteroselectivity.

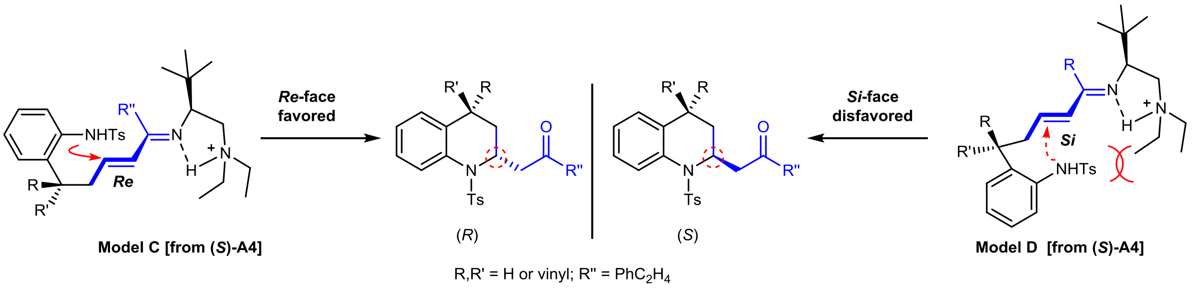

Supplementary Figure 95. Proposed stereochemical model for the cycloadditions of β,γ-unsaturated ketones

For the formal cycloaddition with β,γ-unsaturated ketone, there are two sequential processes including an Ir-catalyzed asymmetric intermolecular allylation of dienamine intermediate and a primary amine-catalyzed asymmetric intramolecular aza-Michael addition. The enantioselectivity of allylation steps was determined by the chiral Ir catalyst according to the work of Sunoj[8] and Carreira.[9,10] The stereo-control of the second step was proposed according to Luo's mode[11] which was used to rationally illustrate the enantio-induction of asymmetric Michael additions using the same

chiral primary amine catalyst. It was believed that, the allylic stereocenter was far from the β-position of conjugated iminium, thus not affecting the intramolecular asymmetric Michael addition step.

Supplementary References

(1) Wang, Q., Li, T.-R., Lu, L.-Q., Li, M.-M., Zhang, K. & Xiao, W.-J. Catalytic asymmetric [4+1] annulation of sulfur ylides with copper-allenylidene intermediates. *J. Am. Chem. Soc.* **138**, 8360-8363 (2016).

(2) Witten, M. R. & Jacobsen, E. N. A Simple primary amine catalyst for enantioselective α-hydroxylations and α-fluorinations of branched aldehydes. *Org. Lett.* **17**, 2772-2775 (2015)**.**

(3) Hoffmann, S., Nicoletti, M. & List, B. Catalytic asymmetric reductive amination of aldehydes via dynamic kinetic resolution. *J. Am. Chem. Soc.* **128**, 13074-13075 (2006).

(4) Cruz, F. A. & Dong, V. M. Stereodivergent coupling of aldehydes and alkynes via synergistic catalysis using Rh and Jacobsen's amine. *J. Am. Chem. Soc.***139**, 1029-1032 (2017).

(5) Franzoni, I., Guénéeb, L. & Mazet, C. Access to congested quaternary centers by Pd-catalyzed intermolecular γ-arylation of unactivated α,β-unsaturated aldehydes *Chem. Sci.* **4**, 2619-2624 (2013).

(6) Hamilton, J. Y., Sarlah, D. & Carreira, E. M. Iridium-catalyzed enantioselective allylic vinylation with potassium alkenyltrifluoroborates. *Org. Synth.* **92**, 1-12 (2015).

(7) Zhan, G., He, Q., Yuan, X. & Chen, Y.-C. Asymmetric direct vinylogous Michael additions of allyl alkyl ketones to maleimides through dienamine catalysis. *Org. Lett*. **16**, 6000-6003 (2014).

(8) Bhaskararao, B. & Sunoj, R. B. Origin of stereodivergence in cooperative asymmetric catalysis with simultaneous involvement of two chiral catalysts. *J. Am. Chem. Soc.* **137**, 15712-15722 (2015).

(9) Rössler, S. L., Krautwald, S. & Carreira, E. M. Study of intermediates in iridium–(phosphoramidite,olefin)-catalyzed enantioselective allylic substitution. *J. Am. Chem. Soc.* **139**, 3603-3606 (2017).

(10) Schafroth, M. A., Rummelt, S. M., Sarlah, D. & Carreira, E. M. Enantioselective iridium-catalyzed allylic cyclizations. *Org. Lett.* **19**, 3235-3238 (2017).

(11) Zhu, L., Zhang, L. & Luo, S. Catalytic asymmetric β-c–h functionalizations of ketones via enamine oxidation. *Org. Lett.* **20**, 1672-1675 (2018).
